# Supplementary figures and images for: Collagen imaging reveals synergistic effects of sutures and host-donor misalignment on topographical irregularities in penetrating keratoplasty (part 2 of 2)
Source: PLoS One. 2024 Aug 8;19(8):e0308204. doi: 10.1371/journal.pone.0308204 (PMC11309498; doi:10.1371/journal.pone.0308204)

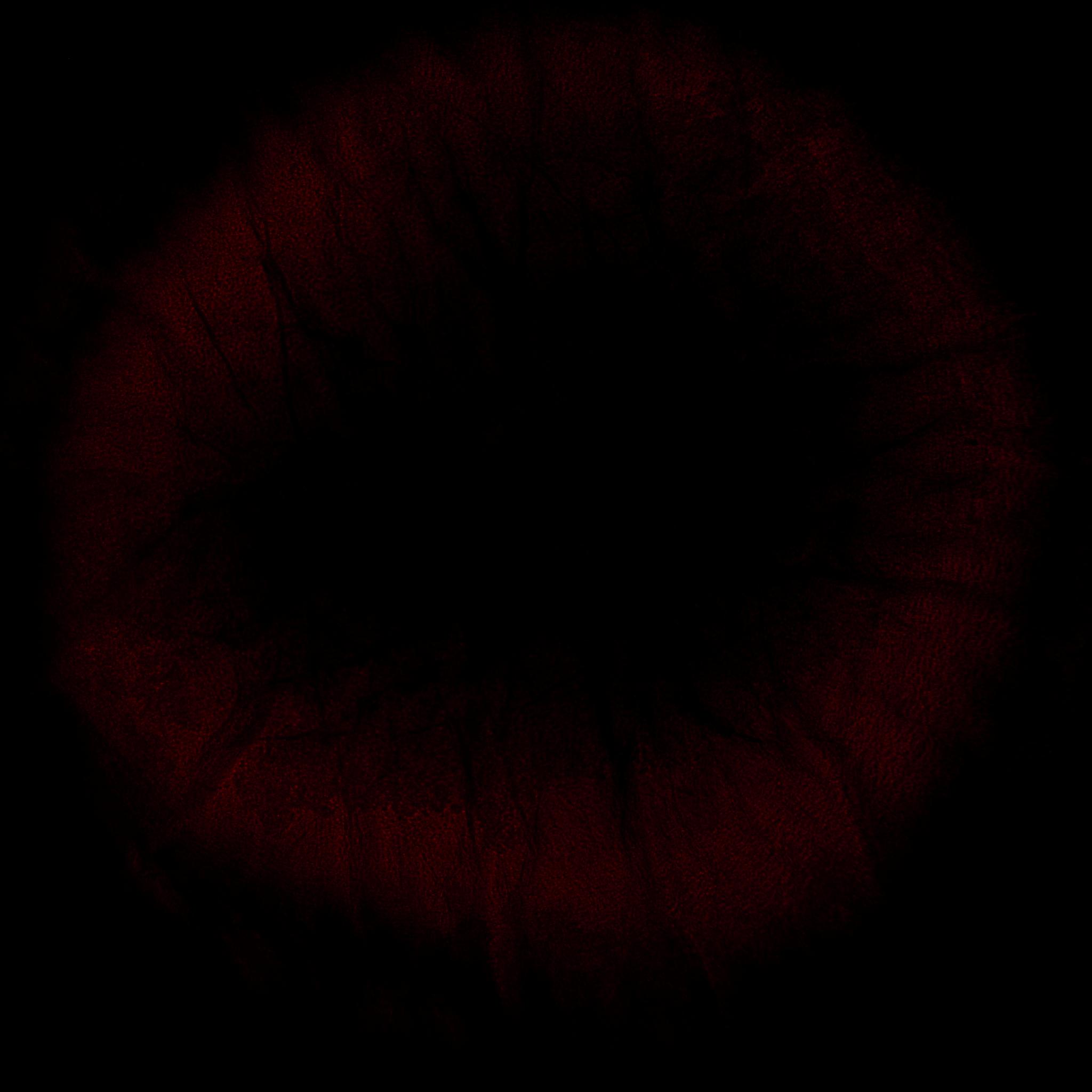

Supplement: S1 File — (ZIP) [file pone.0308204.s001.zip › S1 file. Birefringence Images/B-PK/0 degee/2349OD/IW6.jpg]

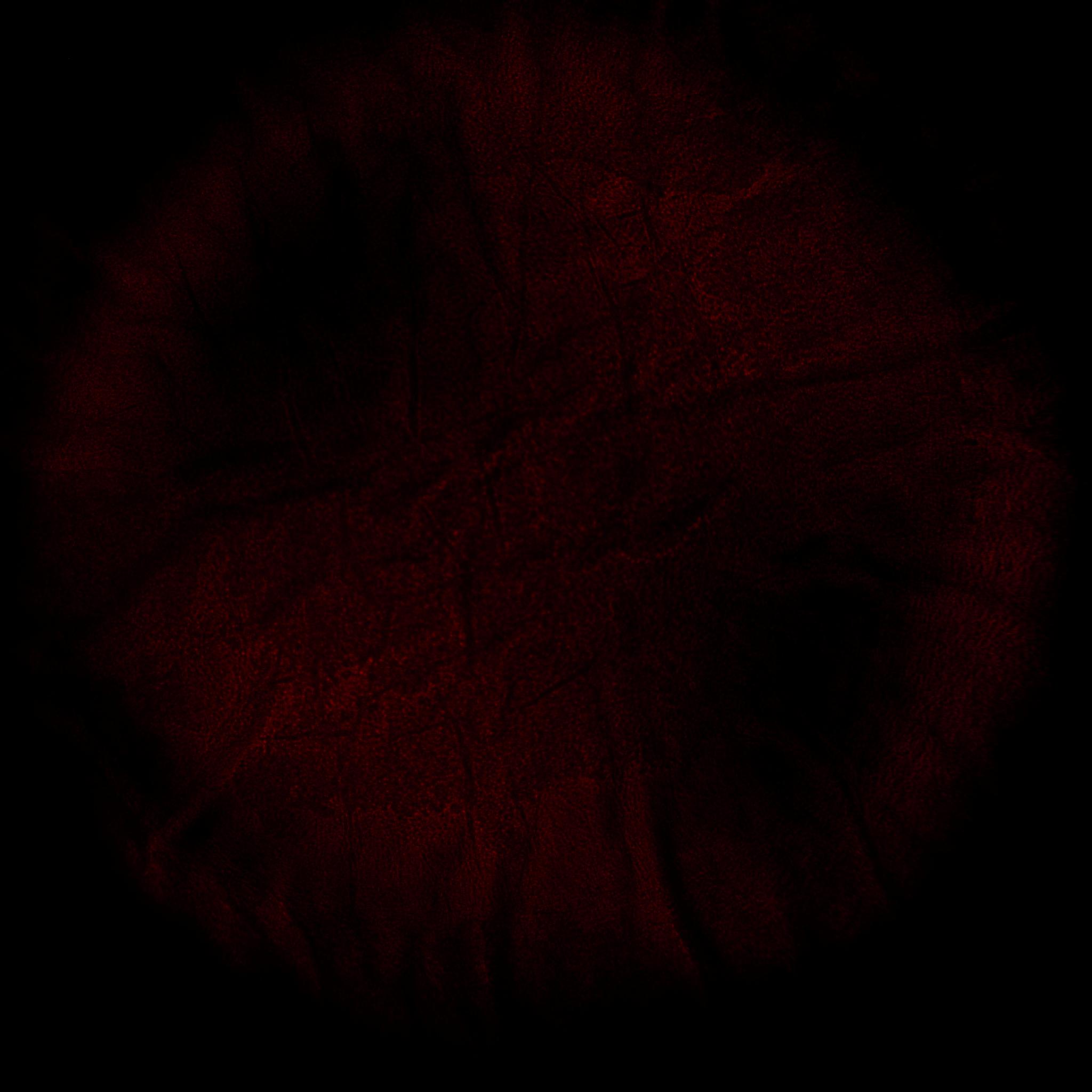

Supplement: S1 File — (ZIP) [file pone.0308204.s001.zip › S1 file. Birefringence Images/B-PK/0 degee/2349OD/IW7.jpg]

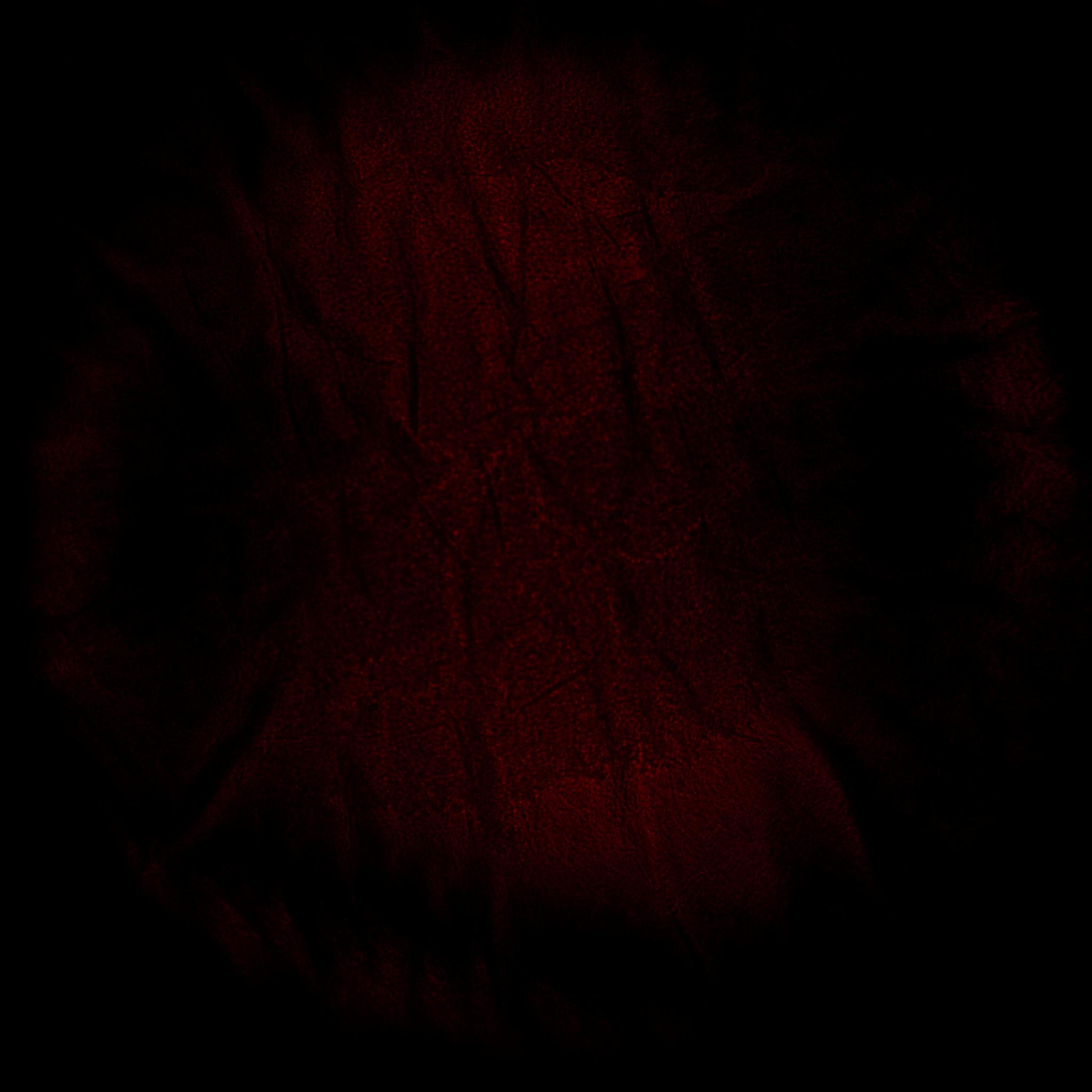

Supplement: S1 File — (ZIP) [file pone.0308204.s001.zip › S1 file. Birefringence Images/B-PK/0 degee/2349OD/IW8.jpg]

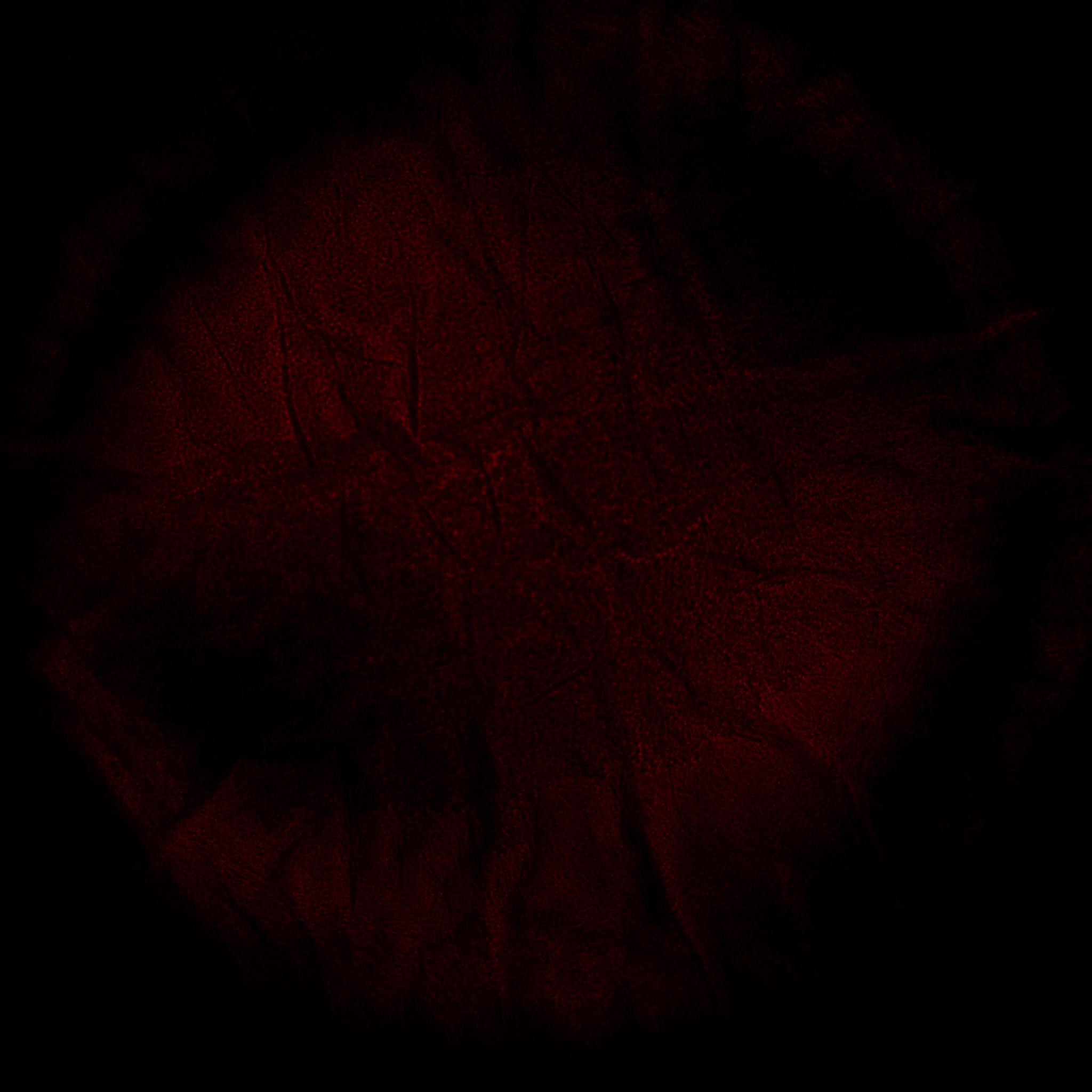

Supplement: S1 File — (ZIP) [file pone.0308204.s001.zip › S1 file. Birefringence Images/B-PK/0 degee/2349OD/IW9.jpg]

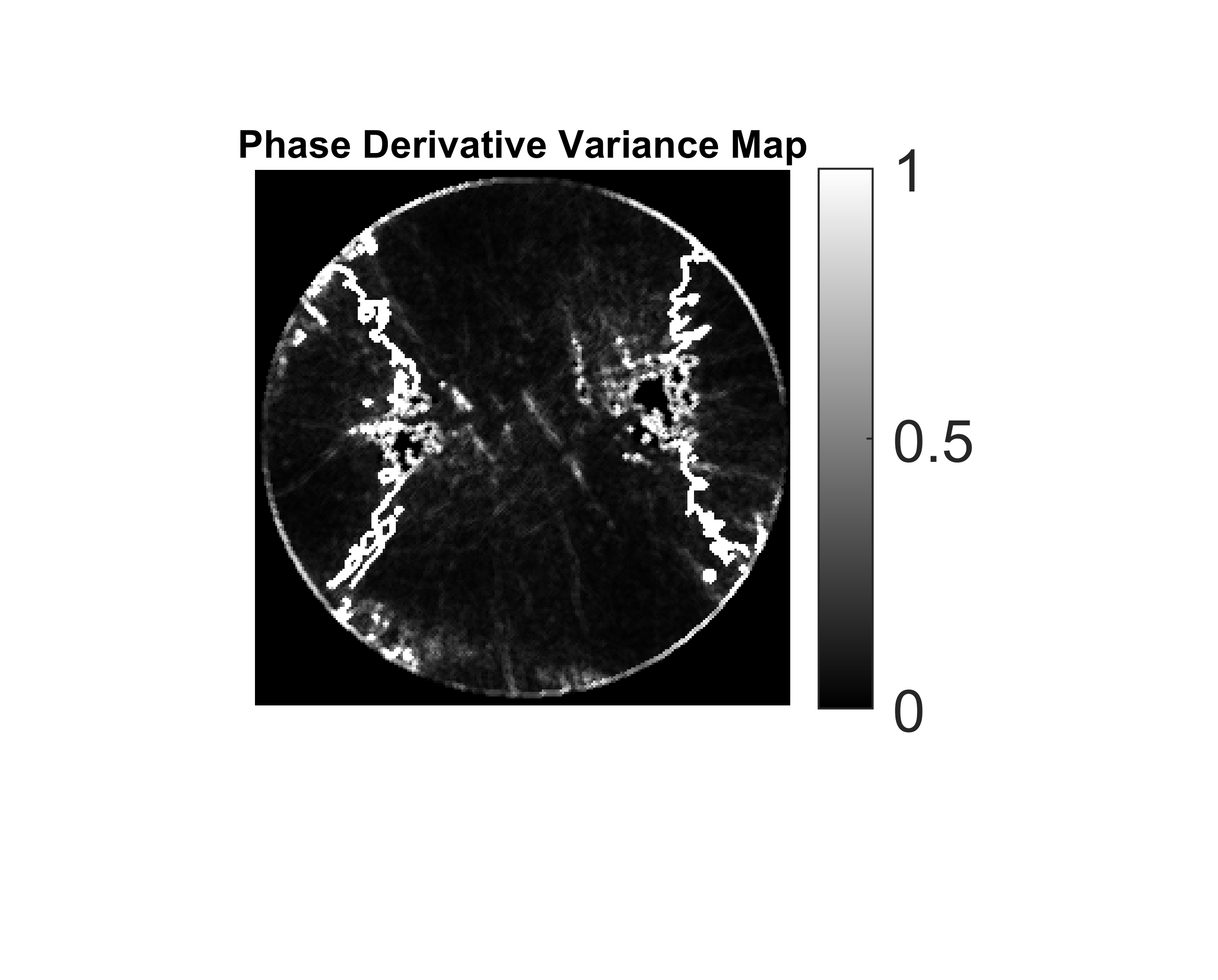

Supplement: S1 File — (ZIP) [file pone.0308204.s001.zip › S1 file. Birefringence Images/B-PK/0 degee/2349OD/PDV.tif]

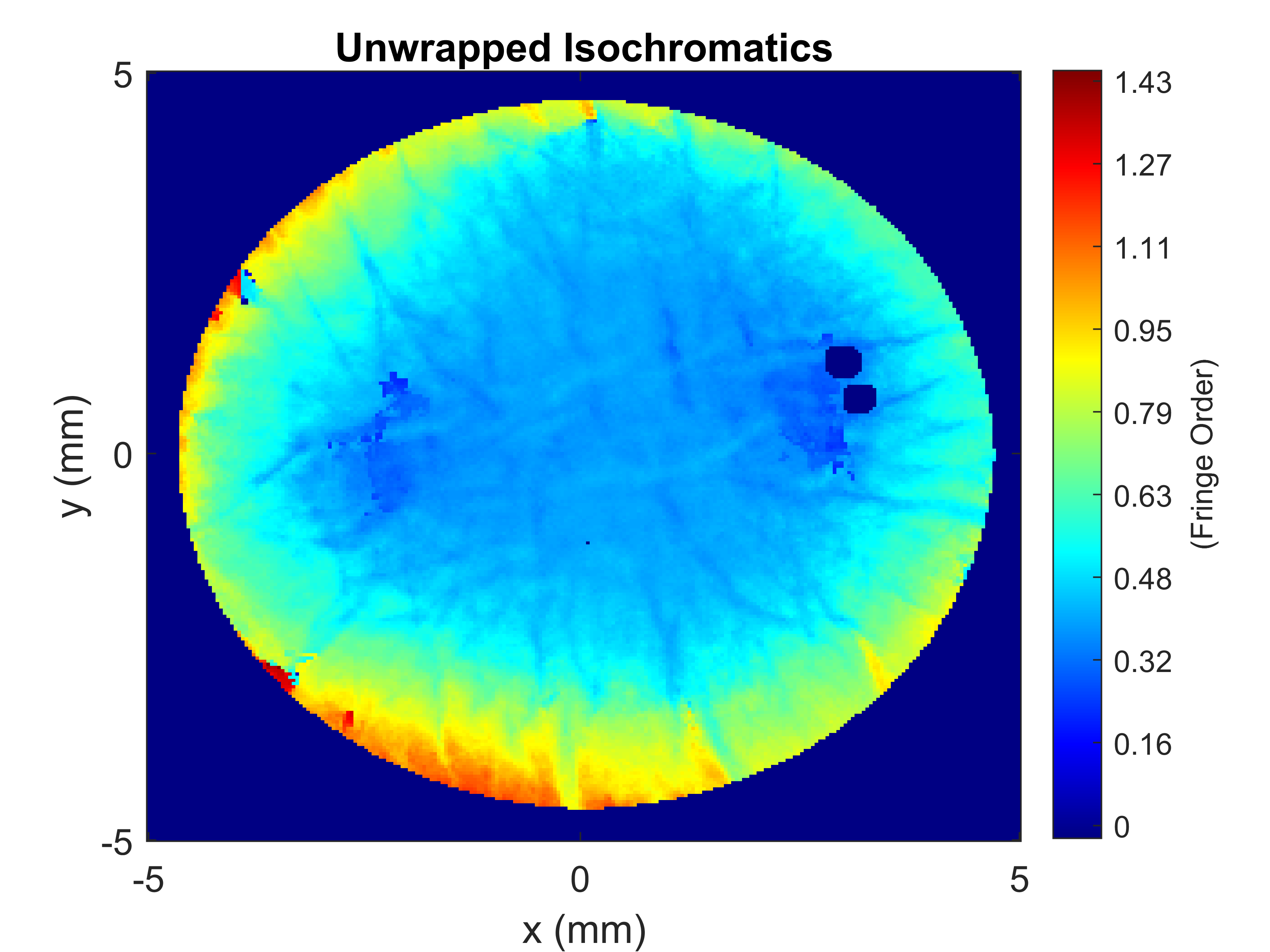

Supplement: S1 File — (ZIP) [file pone.0308204.s001.zip › S1 file. Birefringence Images/B-PK/0 degee/2349OD/unwappedISOCHcolo.tif]

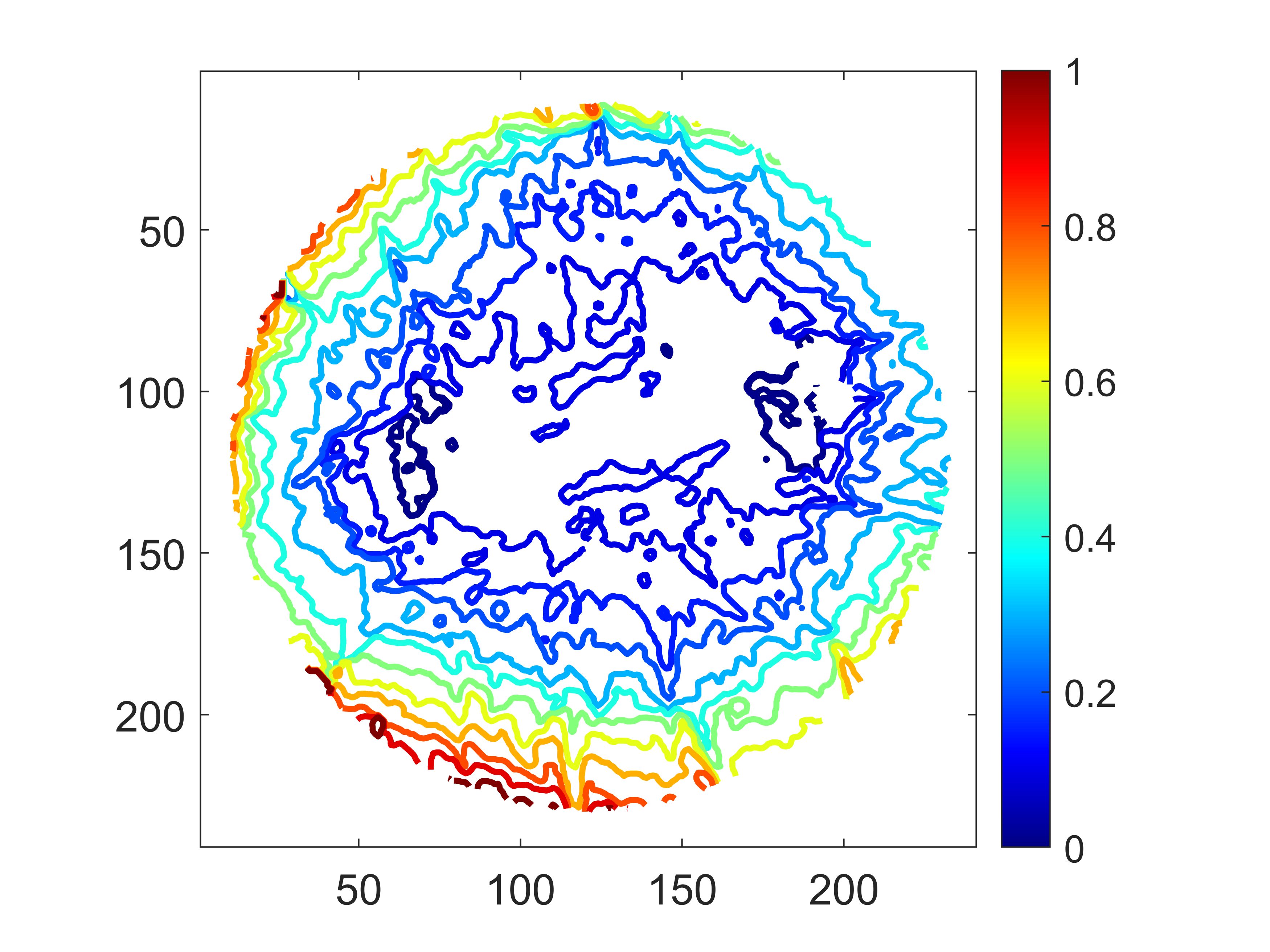

Supplement: S1 File — (ZIP) [file pone.0308204.s001.zip › S1 file. Birefringence Images/B-PK/0 degee/2349OD/unwappedISOCHconou.tif]

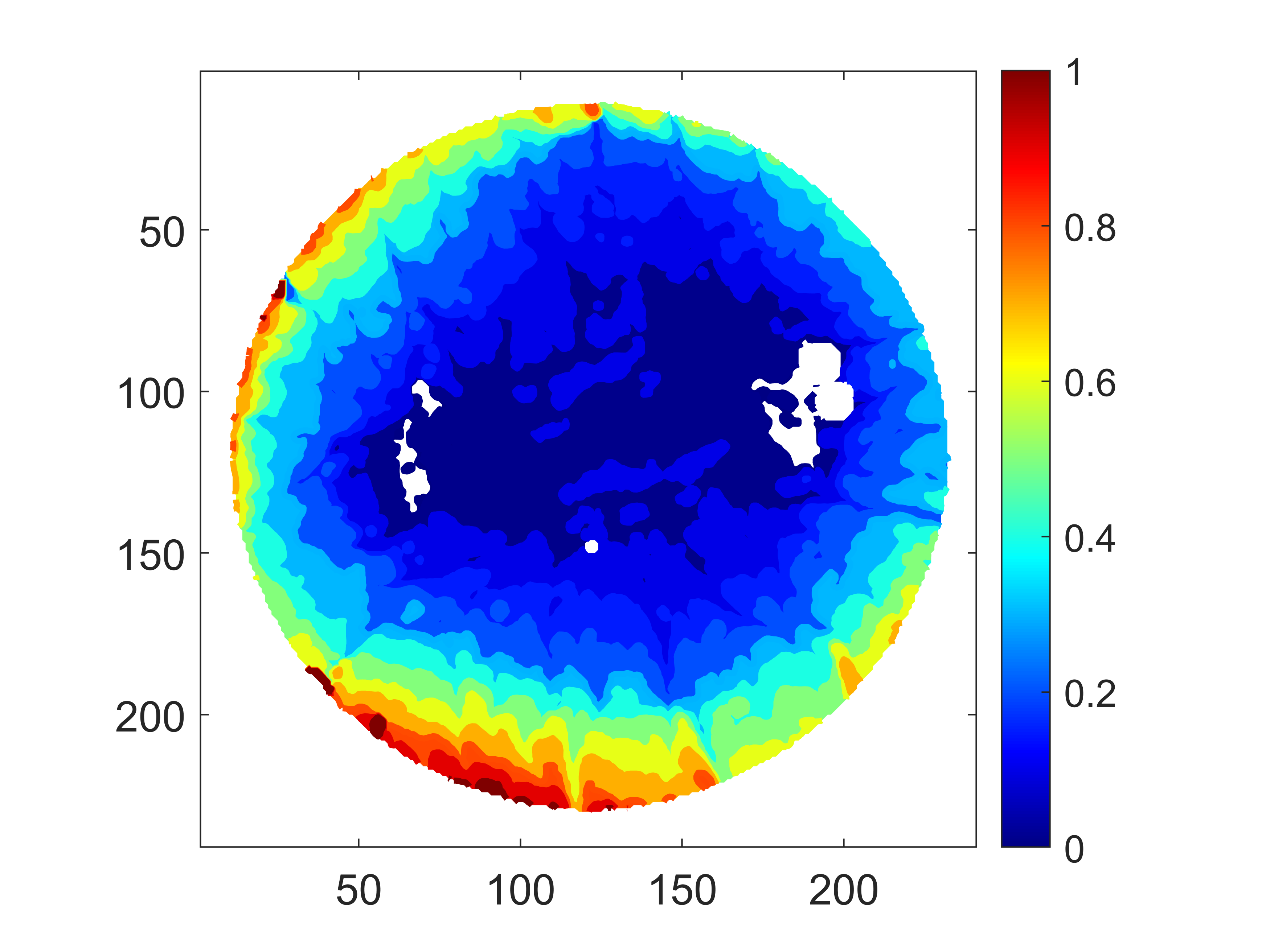

Supplement: S1 File — (ZIP) [file pone.0308204.s001.zip › S1 file. Birefringence Images/B-PK/0 degee/2349OD/unwappedISOCHfill.tif]

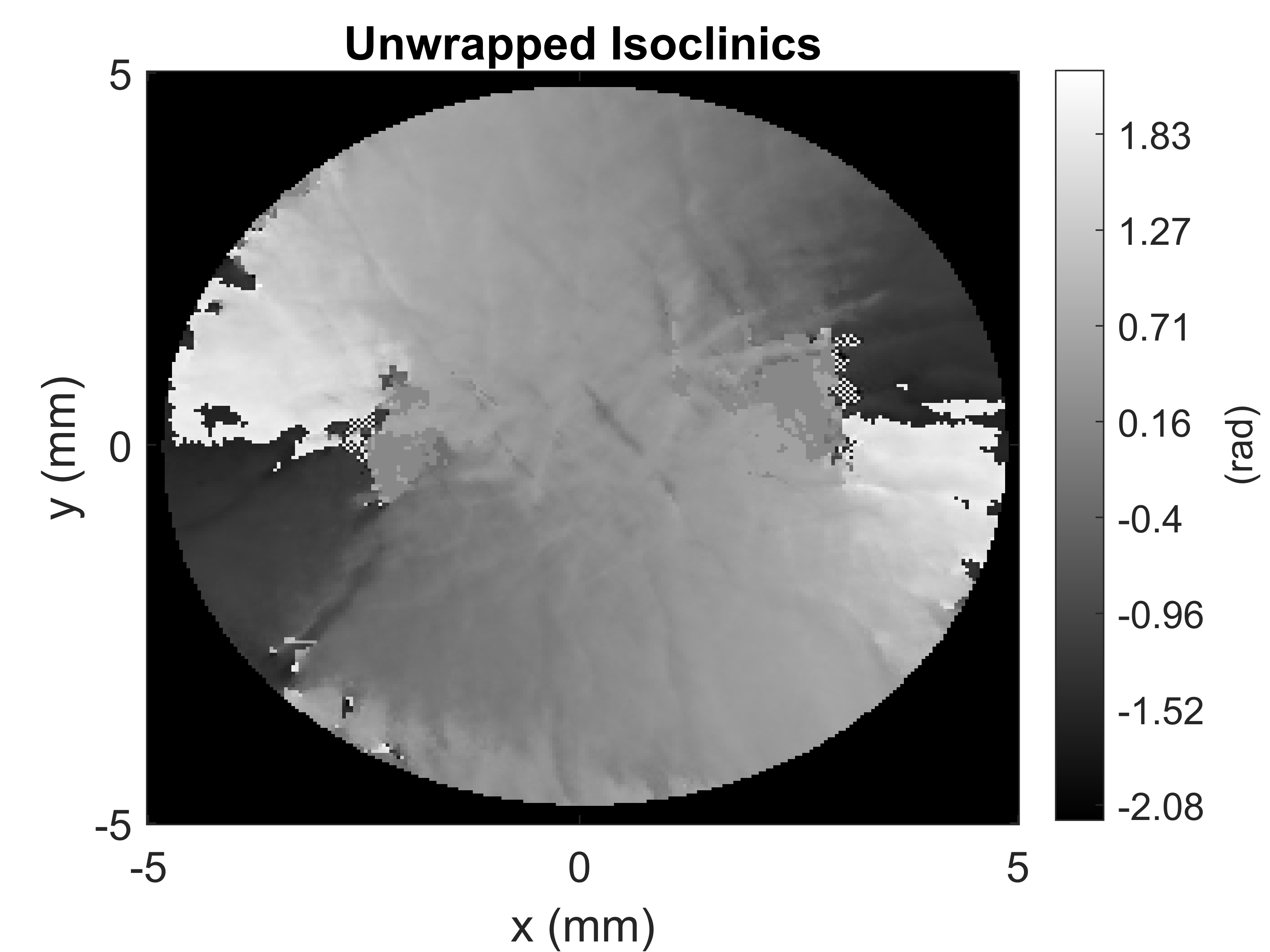

Supplement: S1 File — (ZIP) [file pone.0308204.s001.zip › S1 file. Birefringence Images/B-PK/0 degee/2349OD/unwppedISO.tif]

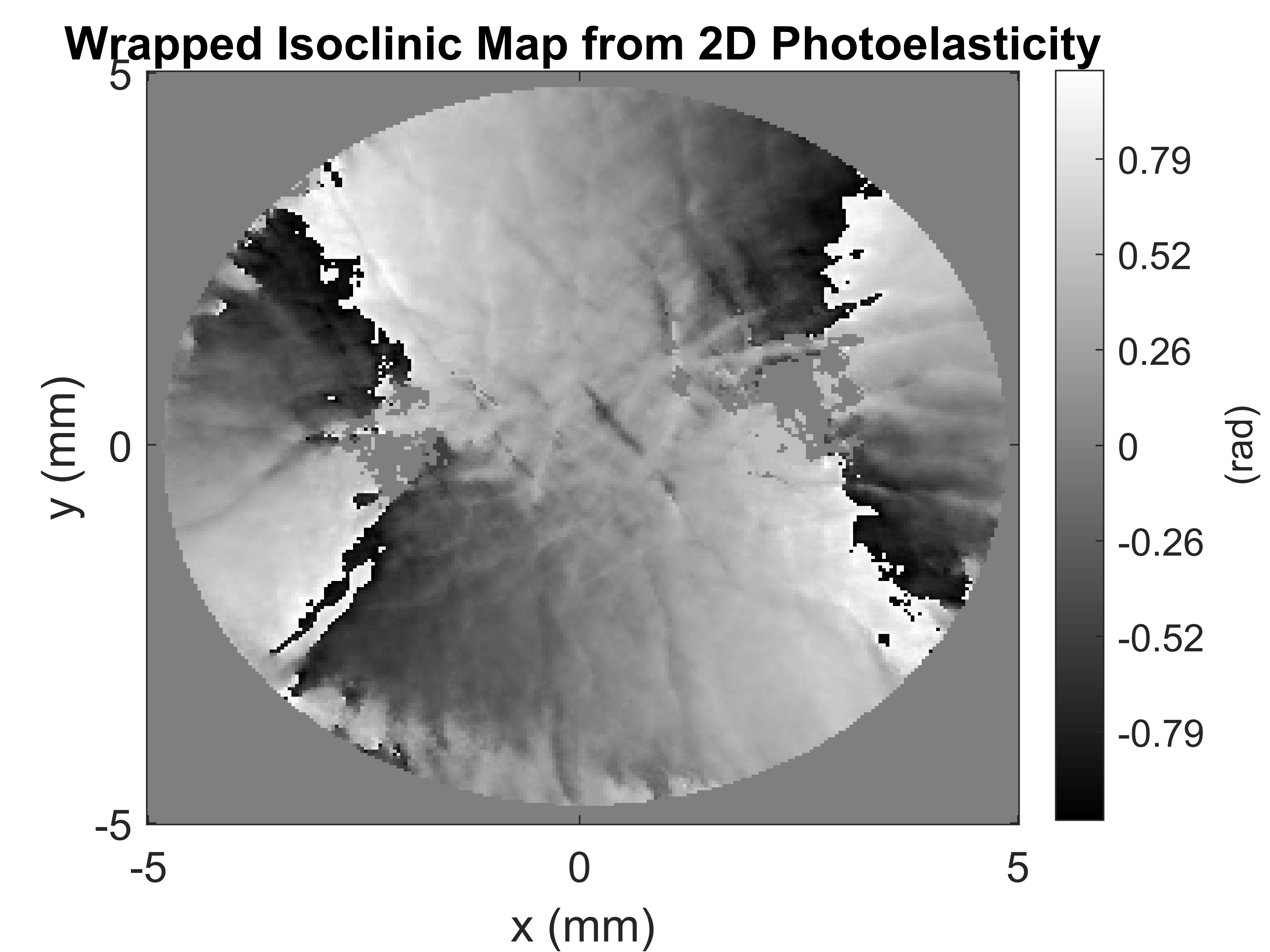

Supplement: S1 File — (ZIP) [file pone.0308204.s001.zip › S1 file. Birefringence Images/B-PK/0 degee/2349OD/wappedISO.tif]

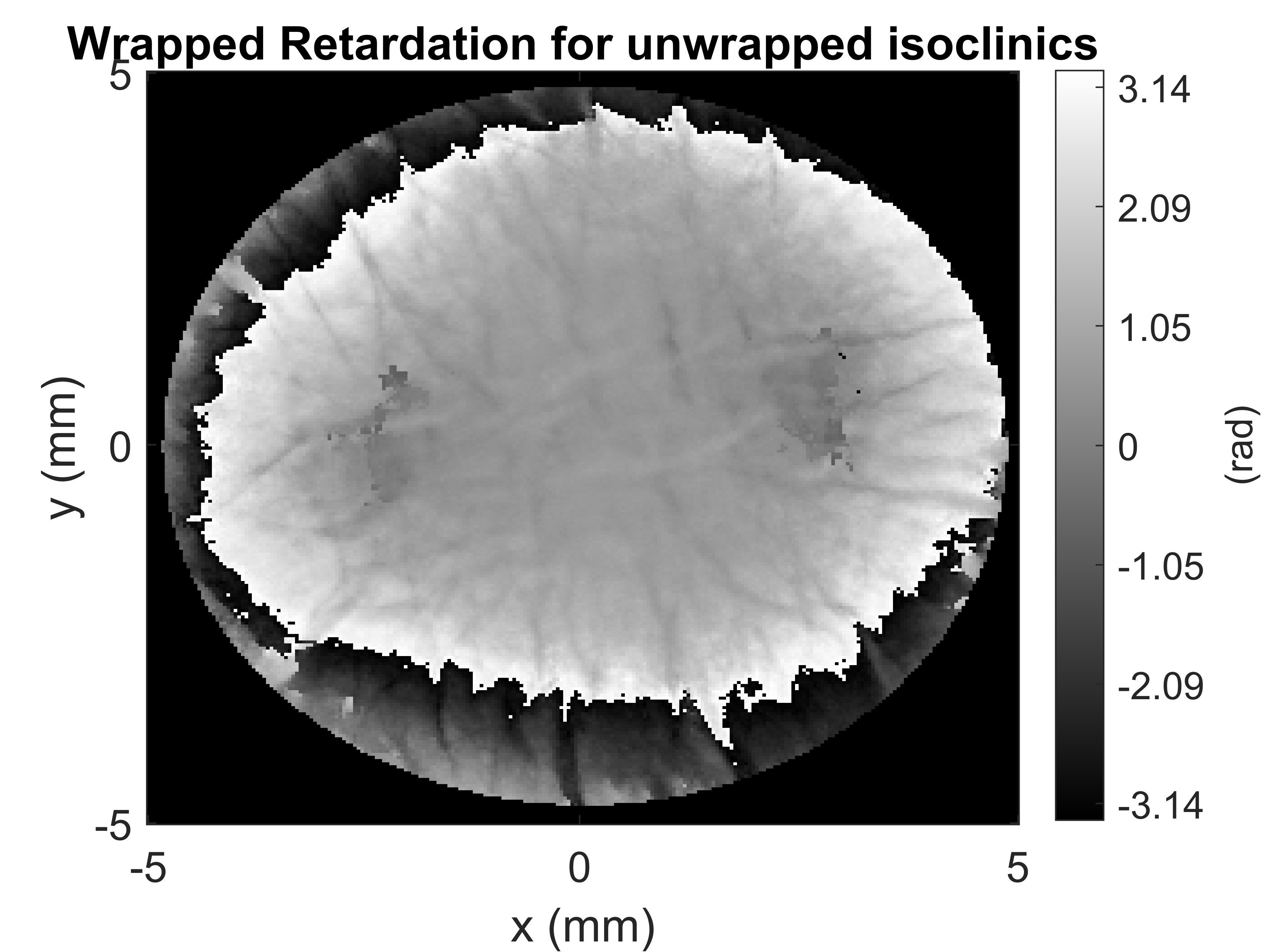

Supplement: S1 File — (ZIP) [file pone.0308204.s001.zip › S1 file. Birefringence Images/B-PK/0 degee/2349OD/wappedISOCHunwappedISO.tif]

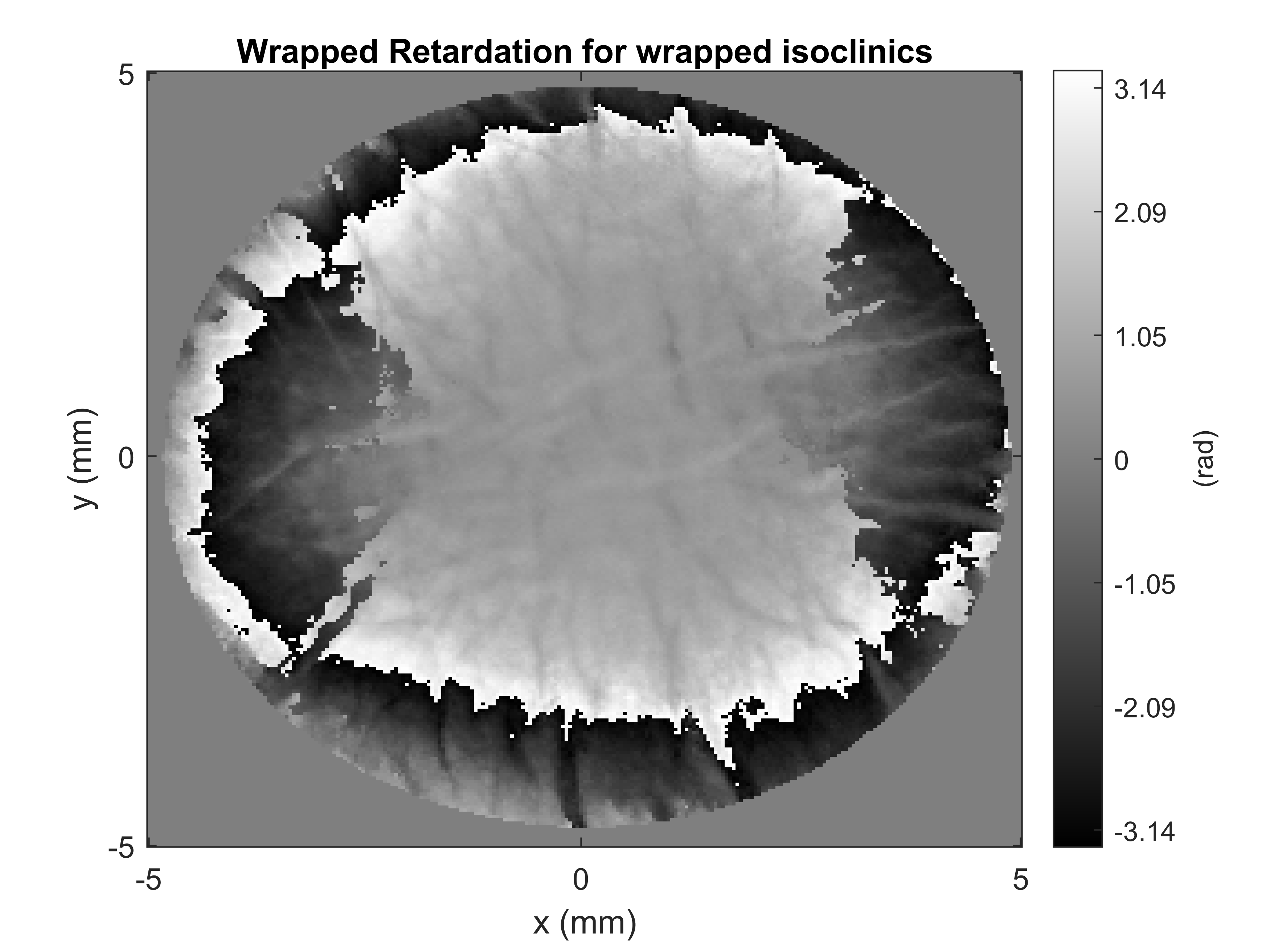

Supplement: S1 File — (ZIP) [file pone.0308204.s001.zip › S1 file. Birefringence Images/B-PK/0 degee/2349OD/wappedISOwappedISOCH.tif]

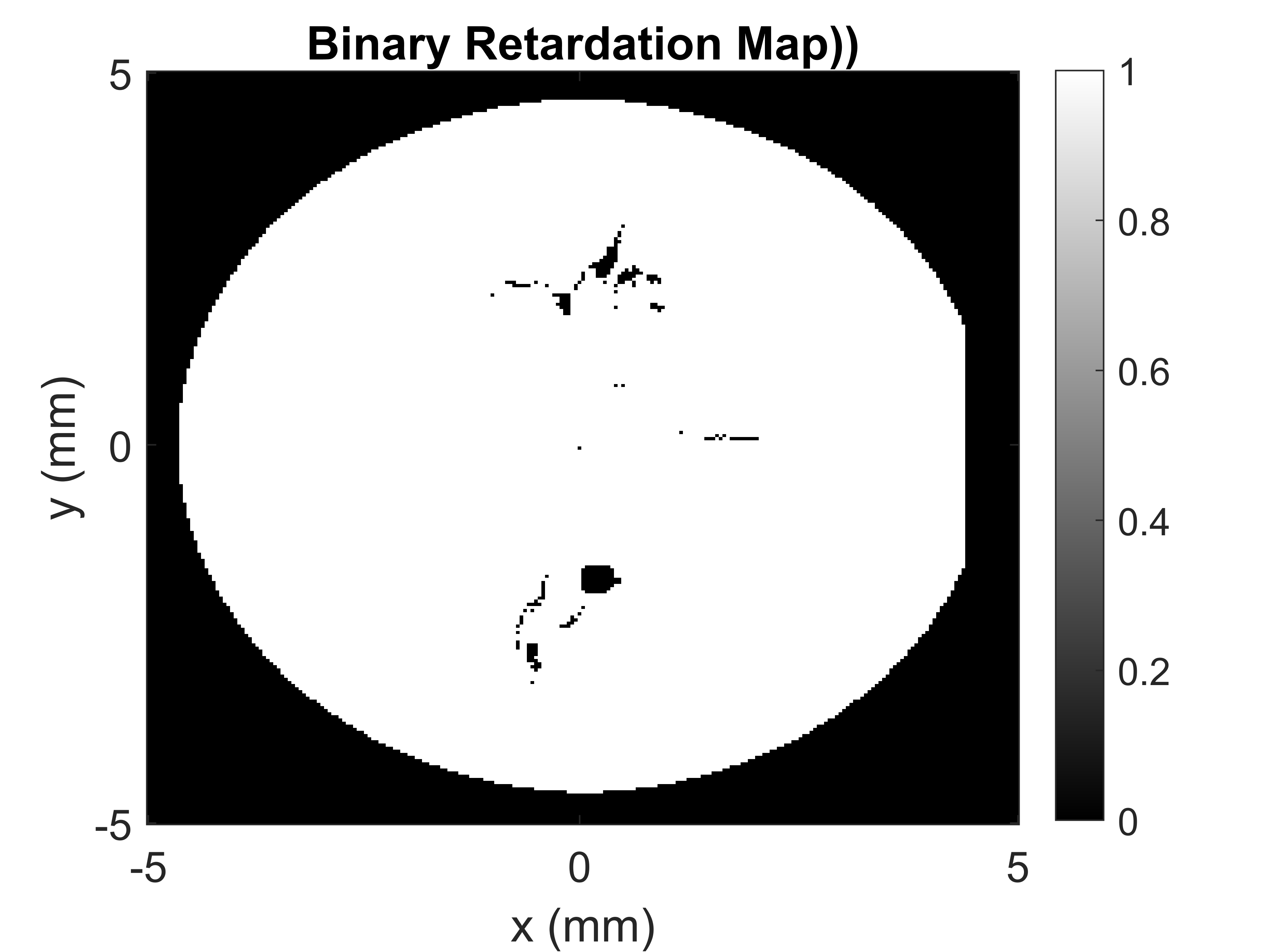

Supplement: S1 File — (ZIP) [file pone.0308204.s001.zip › S1 file. Birefringence Images/B-PK/30 degee/2391OD/ISOOPIC.tif]

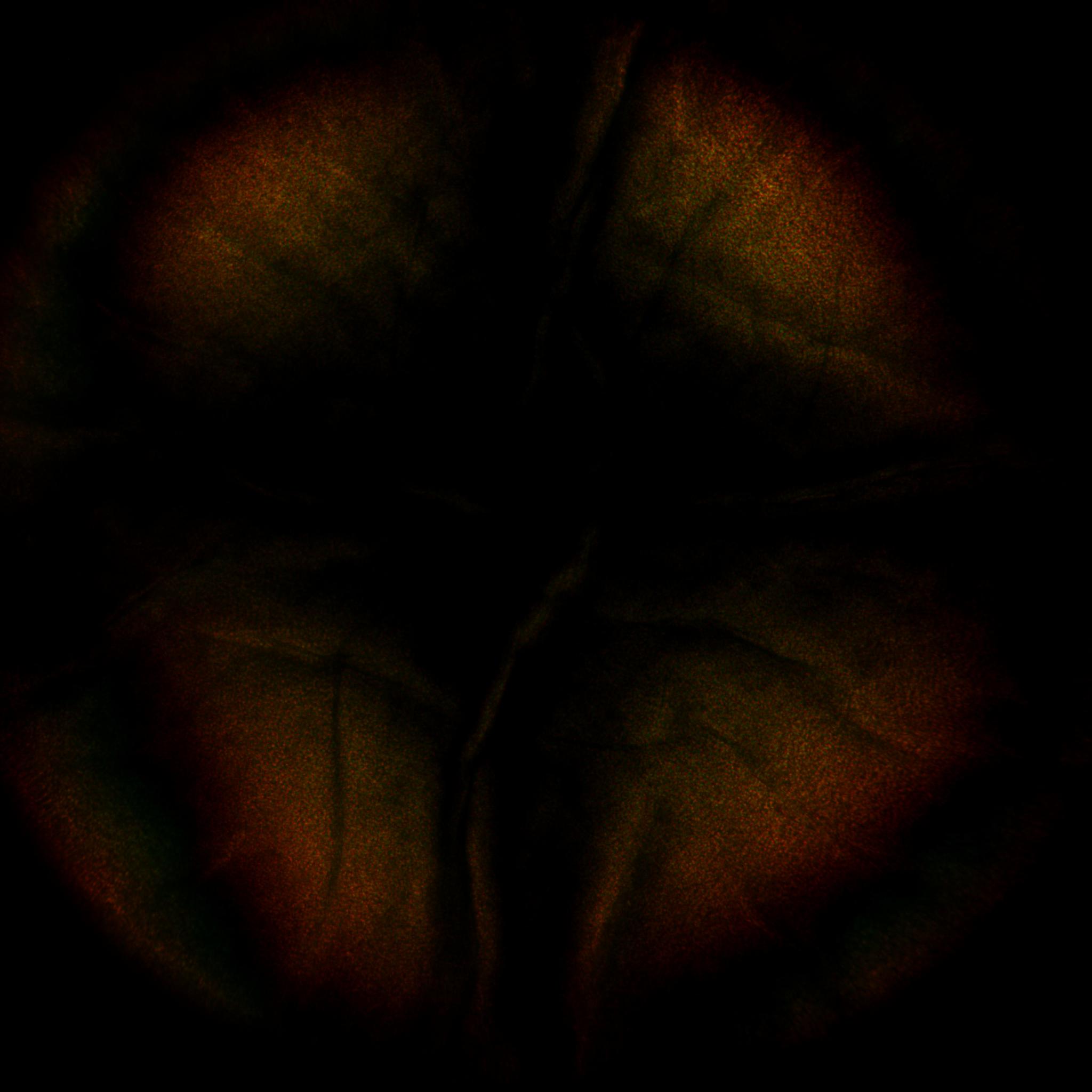

Supplement: S1 File — (ZIP) [file pone.0308204.s001.zip › S1 file. Birefringence Images/B-PK/30 degee/2391OD/IW1.jpg]

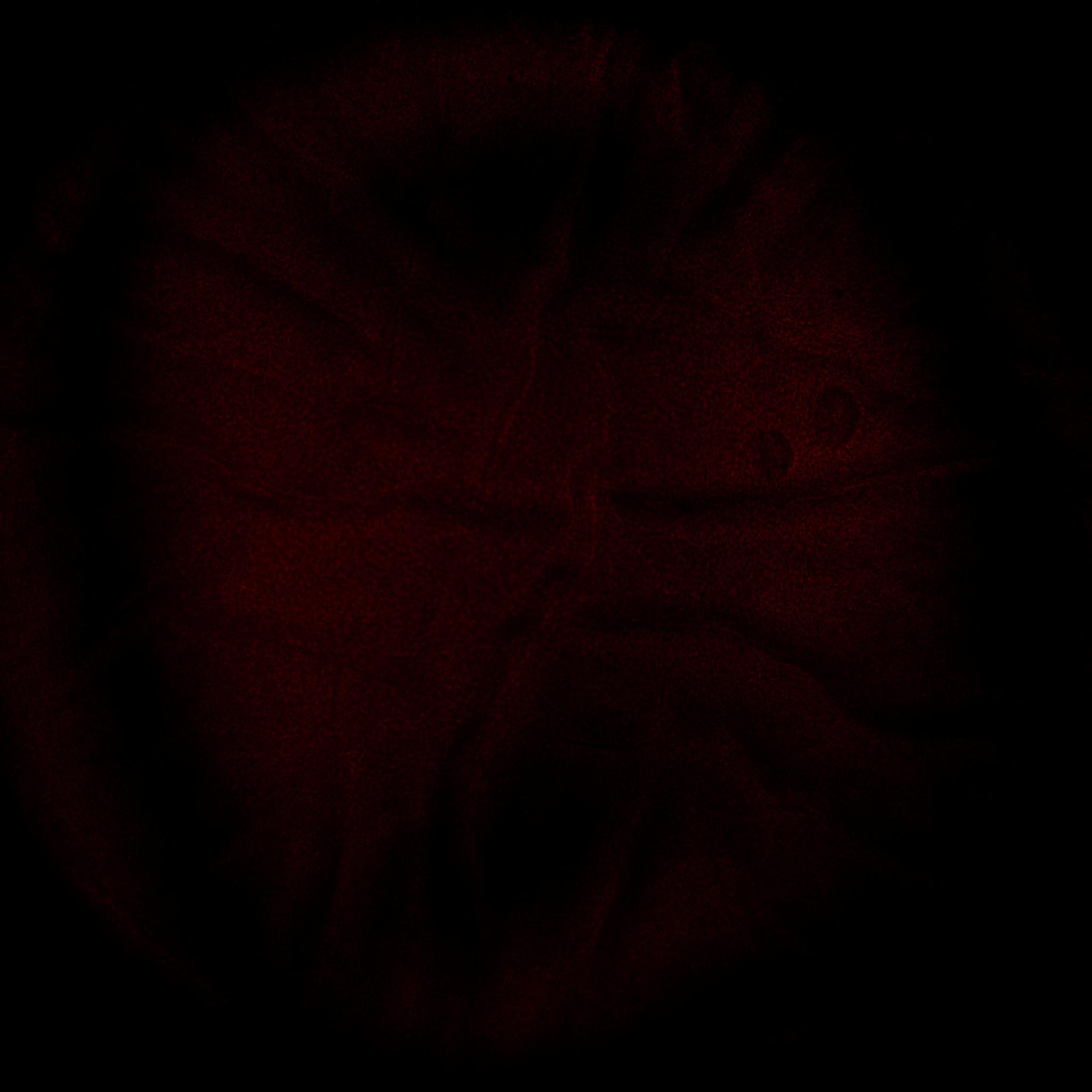

Supplement: S1 File — (ZIP) [file pone.0308204.s001.zip › S1 file. Birefringence Images/B-PK/30 degee/2391OD/IW10.jpg]

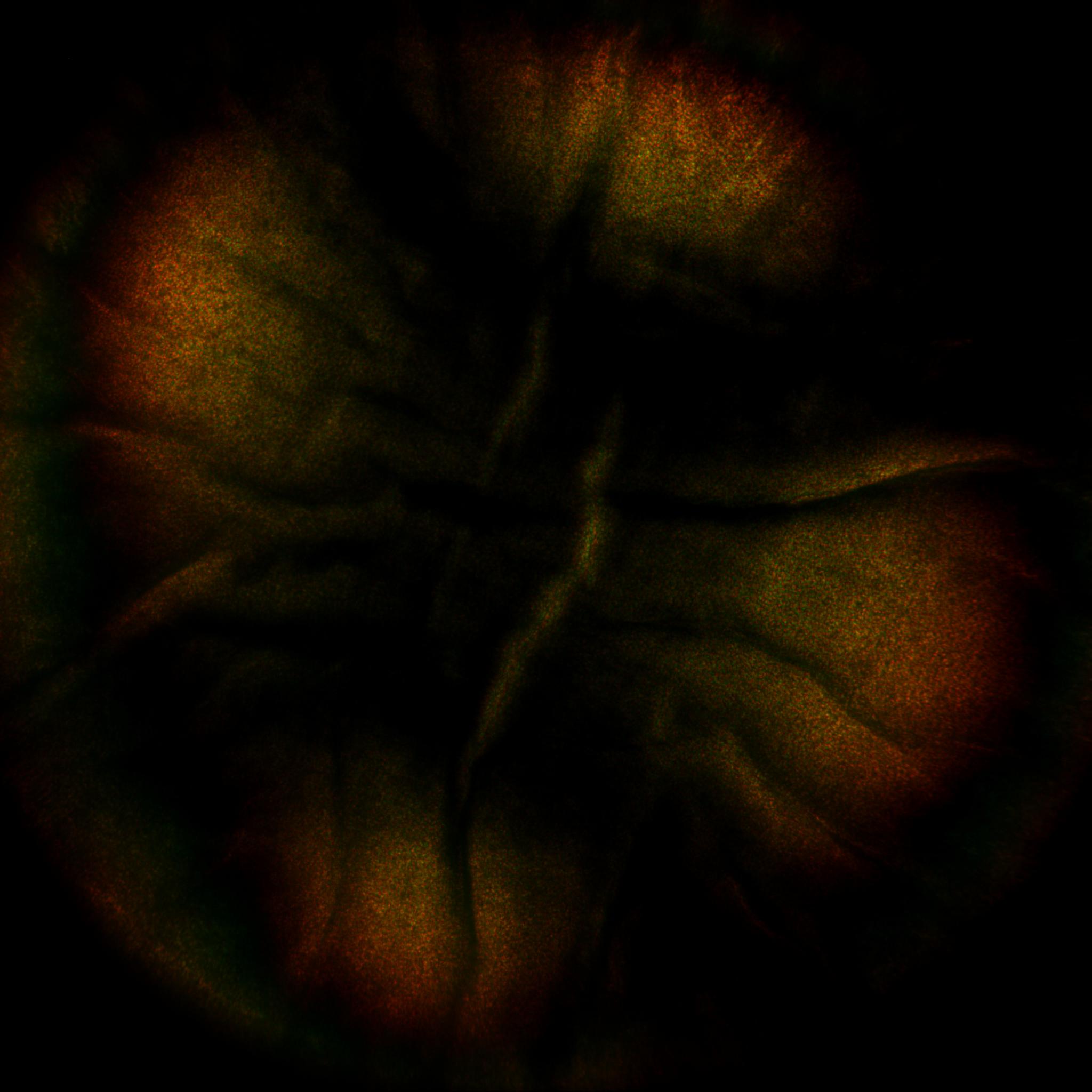

Supplement: S1 File — (ZIP) [file pone.0308204.s001.zip › S1 file. Birefringence Images/B-PK/30 degee/2391OD/IW2.jpg]

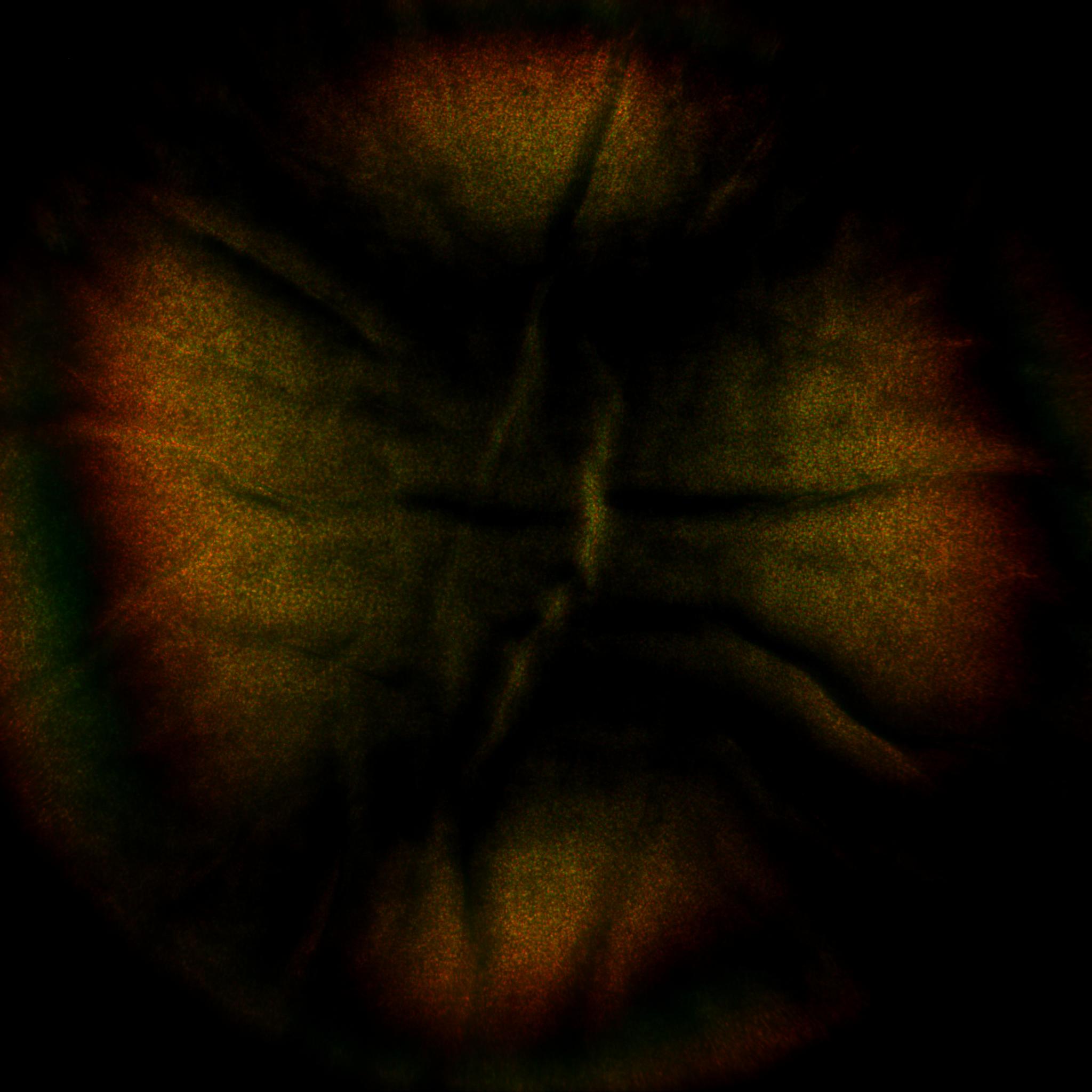

Supplement: S1 File — (ZIP) [file pone.0308204.s001.zip › S1 file. Birefringence Images/B-PK/30 degee/2391OD/IW3.jpg]

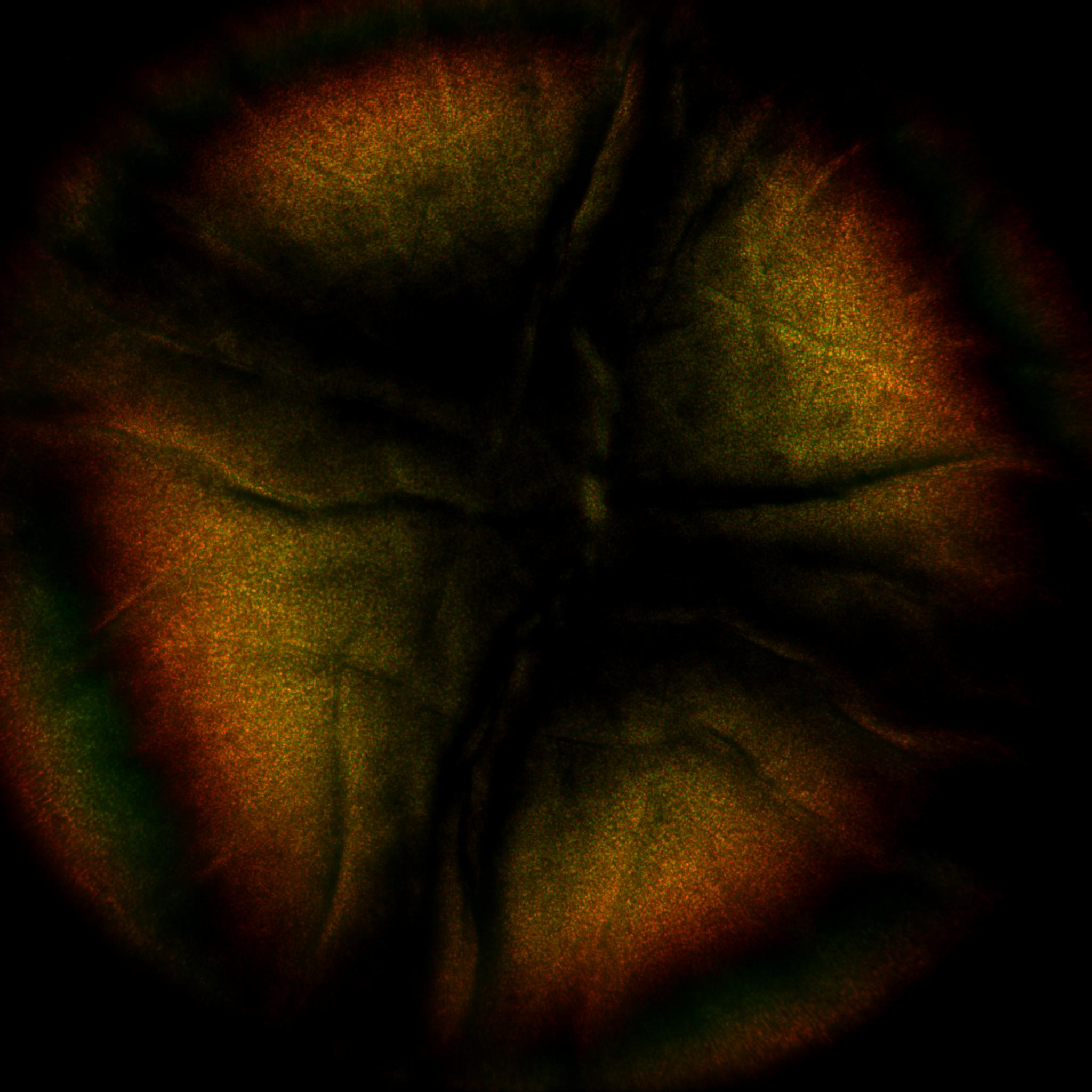

Supplement: S1 File — (ZIP) [file pone.0308204.s001.zip › S1 file. Birefringence Images/B-PK/30 degee/2391OD/IW4.jpg]

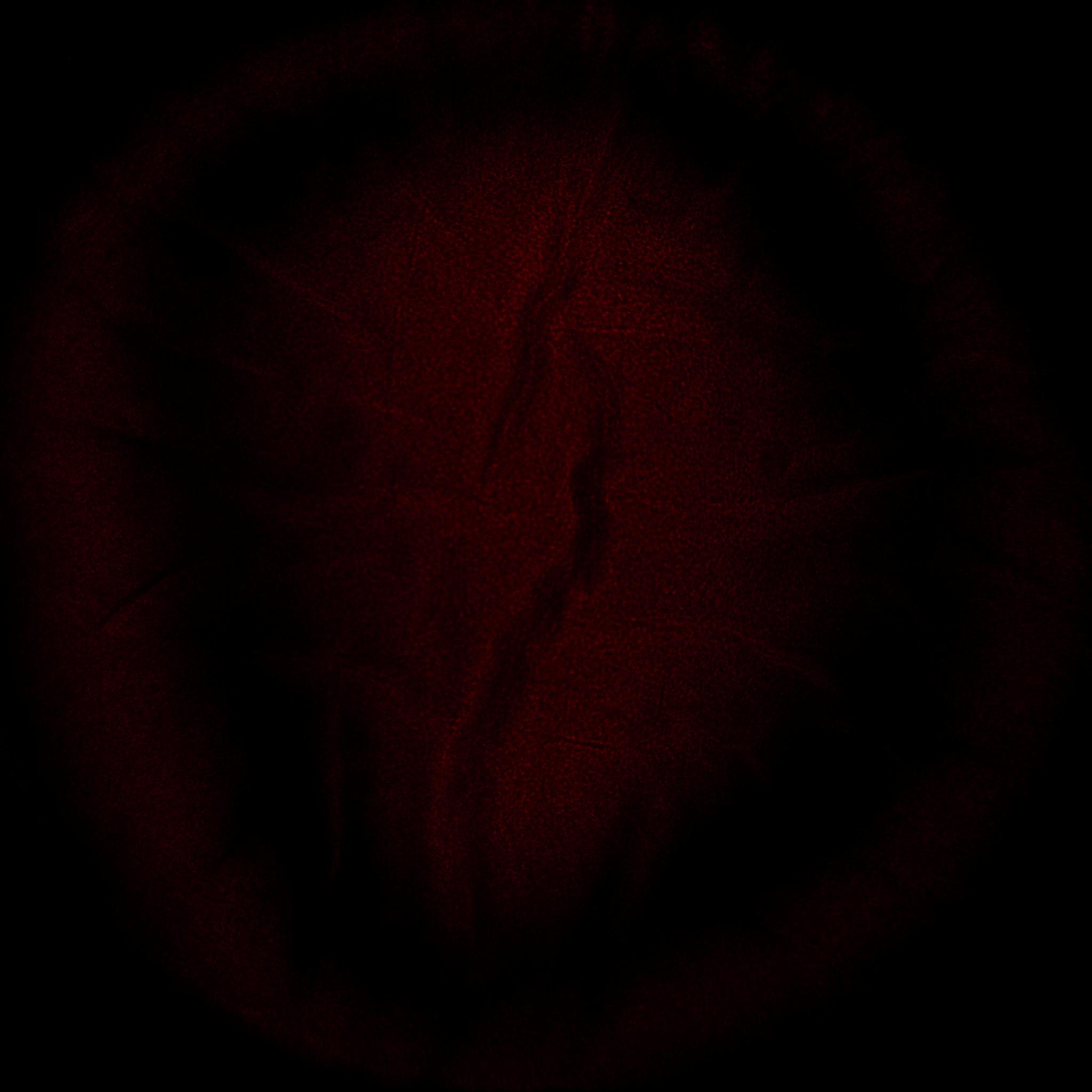

Supplement: S1 File — (ZIP) [file pone.0308204.s001.zip › S1 file. Birefringence Images/B-PK/30 degee/2391OD/IW5.jpg]

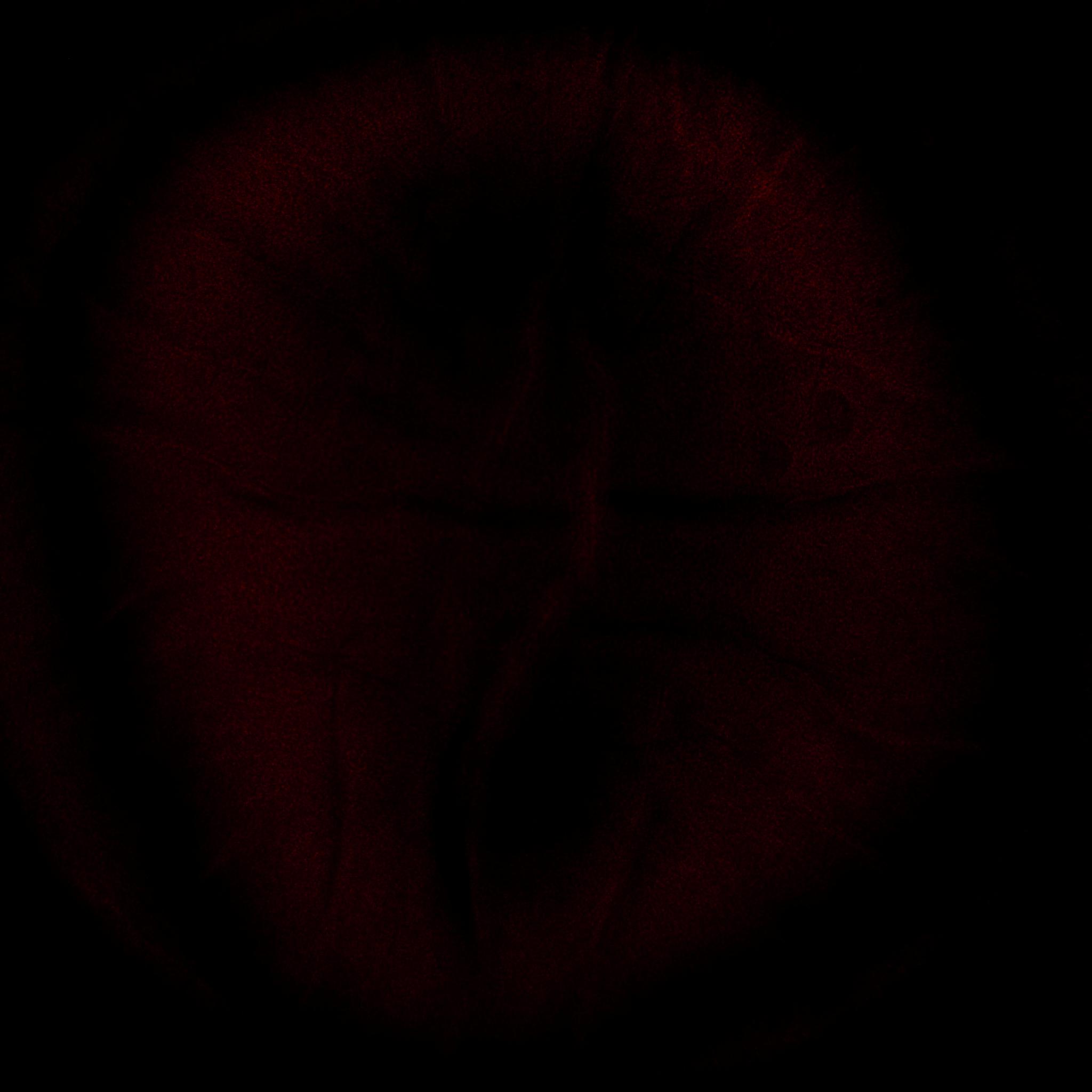

Supplement: S1 File — (ZIP) [file pone.0308204.s001.zip › S1 file. Birefringence Images/B-PK/30 degee/2391OD/IW6.jpg]

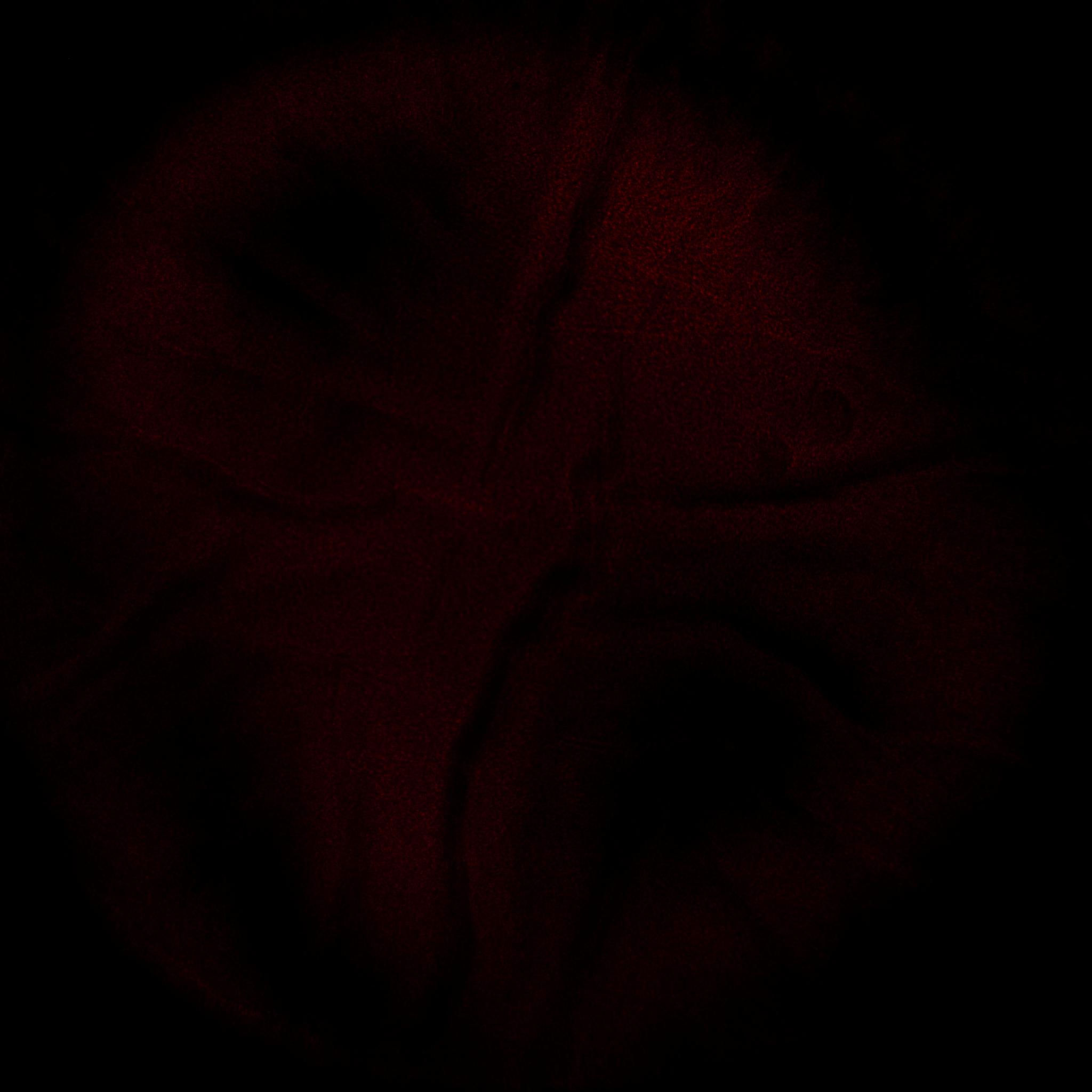

Supplement: S1 File — (ZIP) [file pone.0308204.s001.zip › S1 file. Birefringence Images/B-PK/30 degee/2391OD/IW7.jpg]

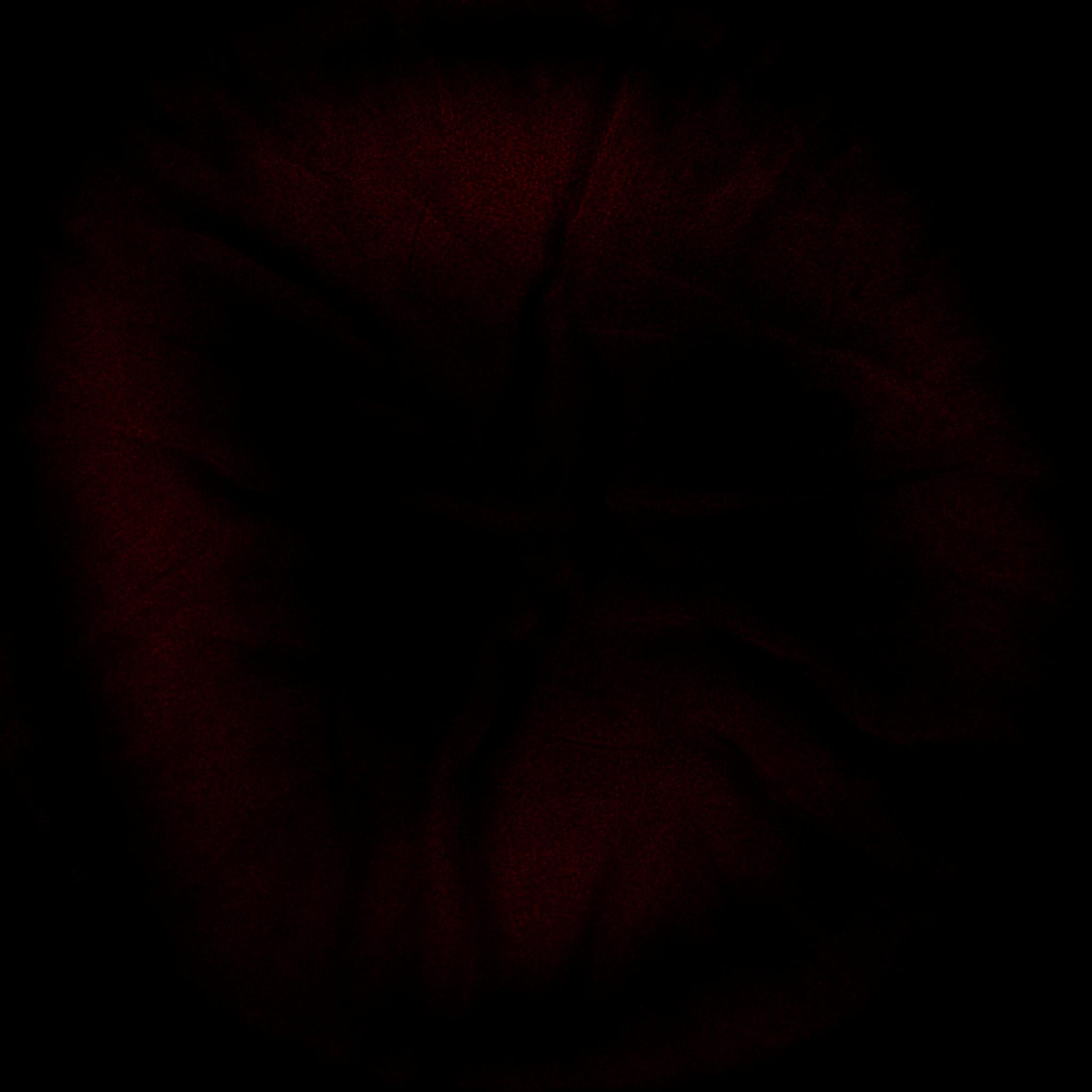

Supplement: S1 File — (ZIP) [file pone.0308204.s001.zip › S1 file. Birefringence Images/B-PK/30 degee/2391OD/IW8.jpg]

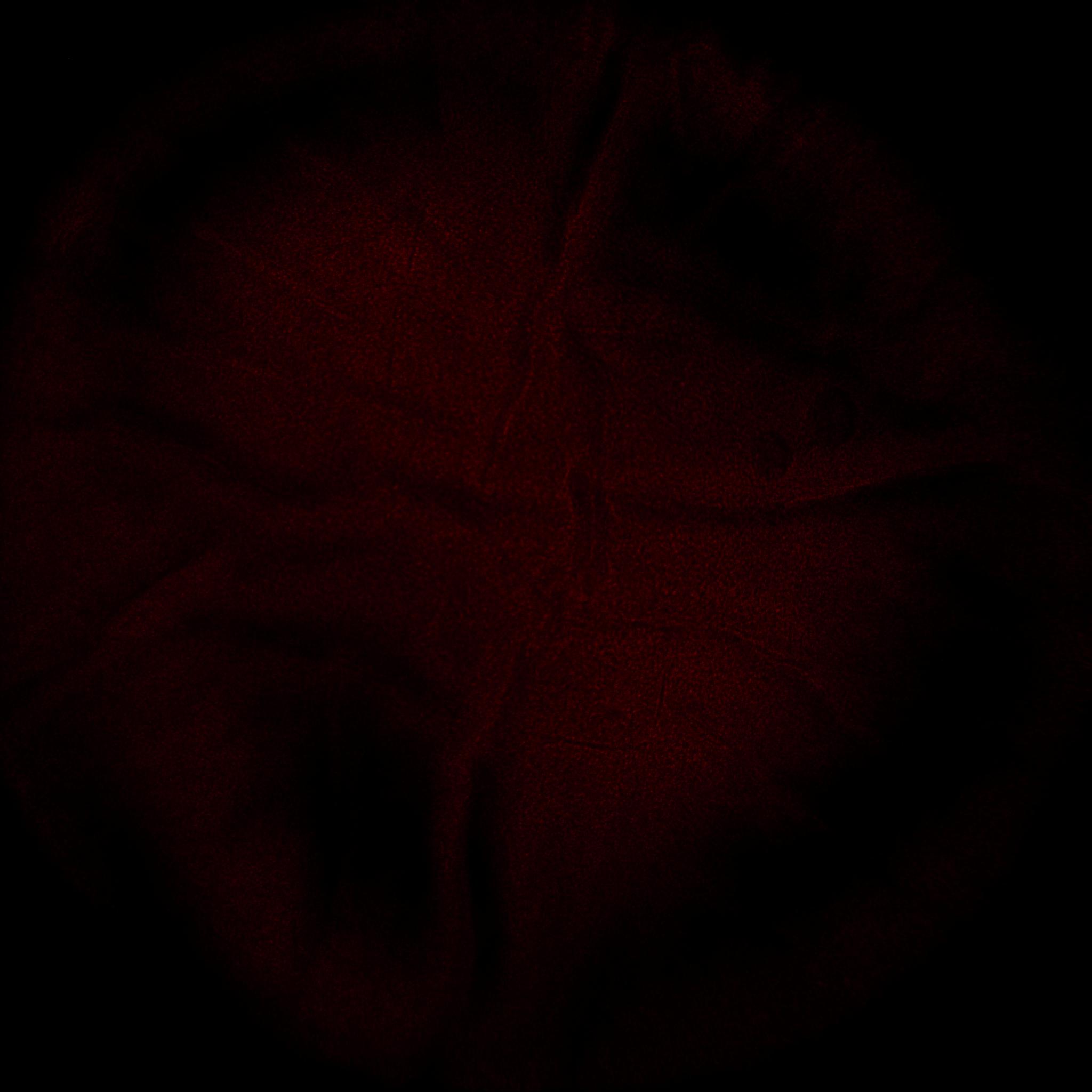

Supplement: S1 File — (ZIP) [file pone.0308204.s001.zip › S1 file. Birefringence Images/B-PK/30 degee/2391OD/IW9.jpg]

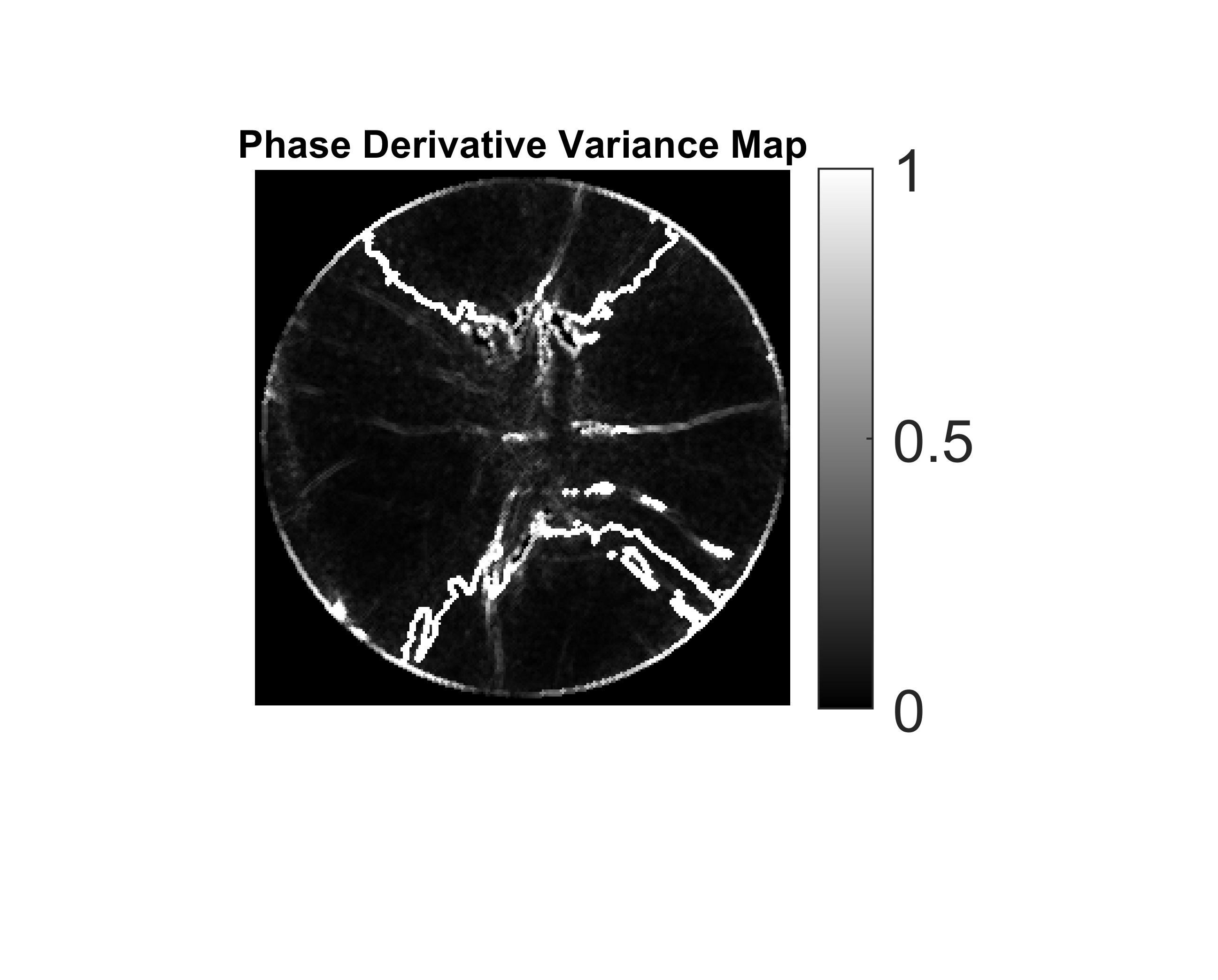

Supplement: S1 File — (ZIP) [file pone.0308204.s001.zip › S1 file. Birefringence Images/B-PK/30 degee/2391OD/PDV.tif]

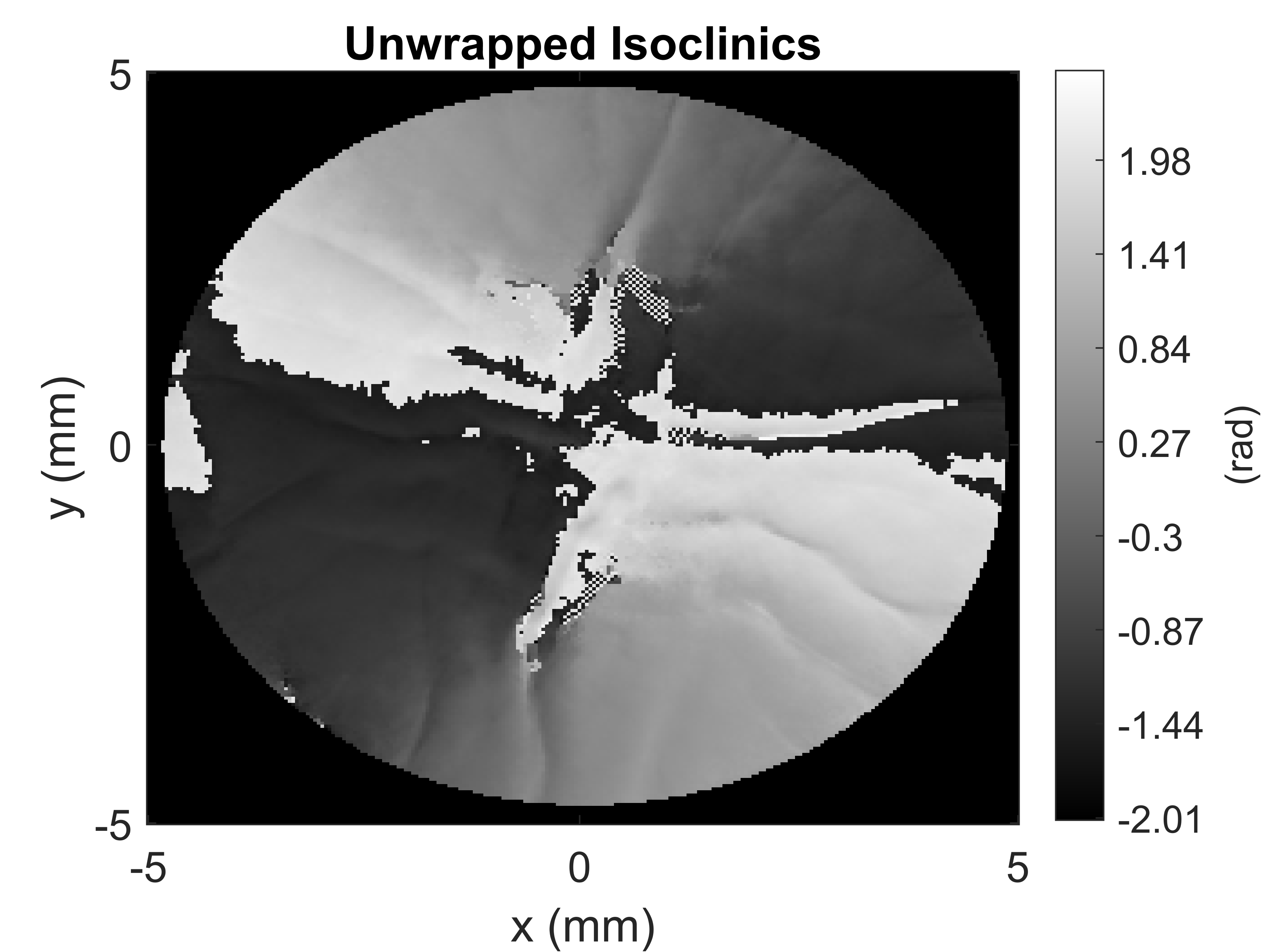

Supplement: S1 File — (ZIP) [file pone.0308204.s001.zip › S1 file. Birefringence Images/B-PK/30 degee/2391OD/unwppedISO.tif]

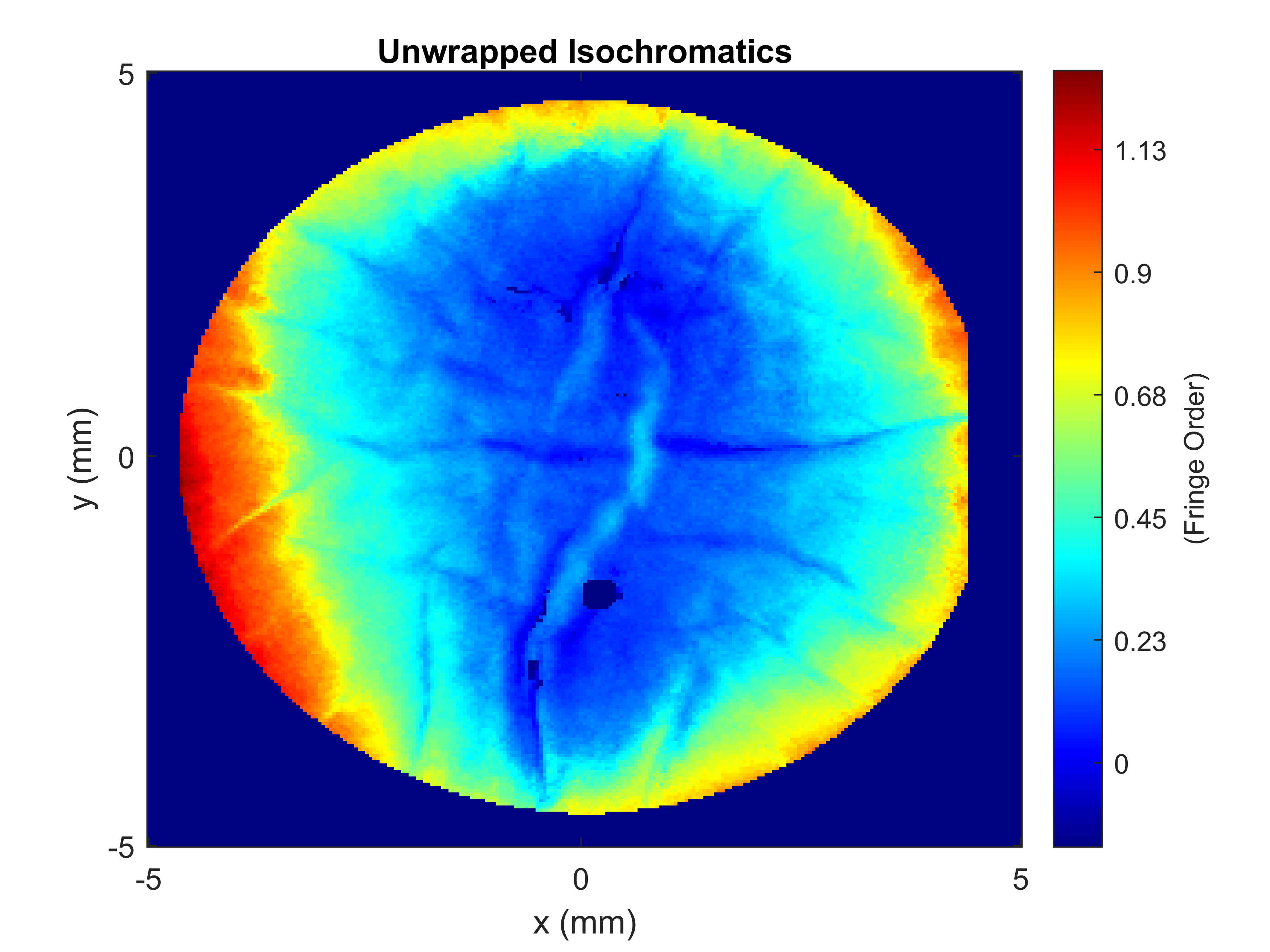

Supplement: S1 File — (ZIP) [file pone.0308204.s001.zip › S1 file. Birefringence Images/B-PK/30 degee/2391OD/unwppedISOCHcolo.tif]

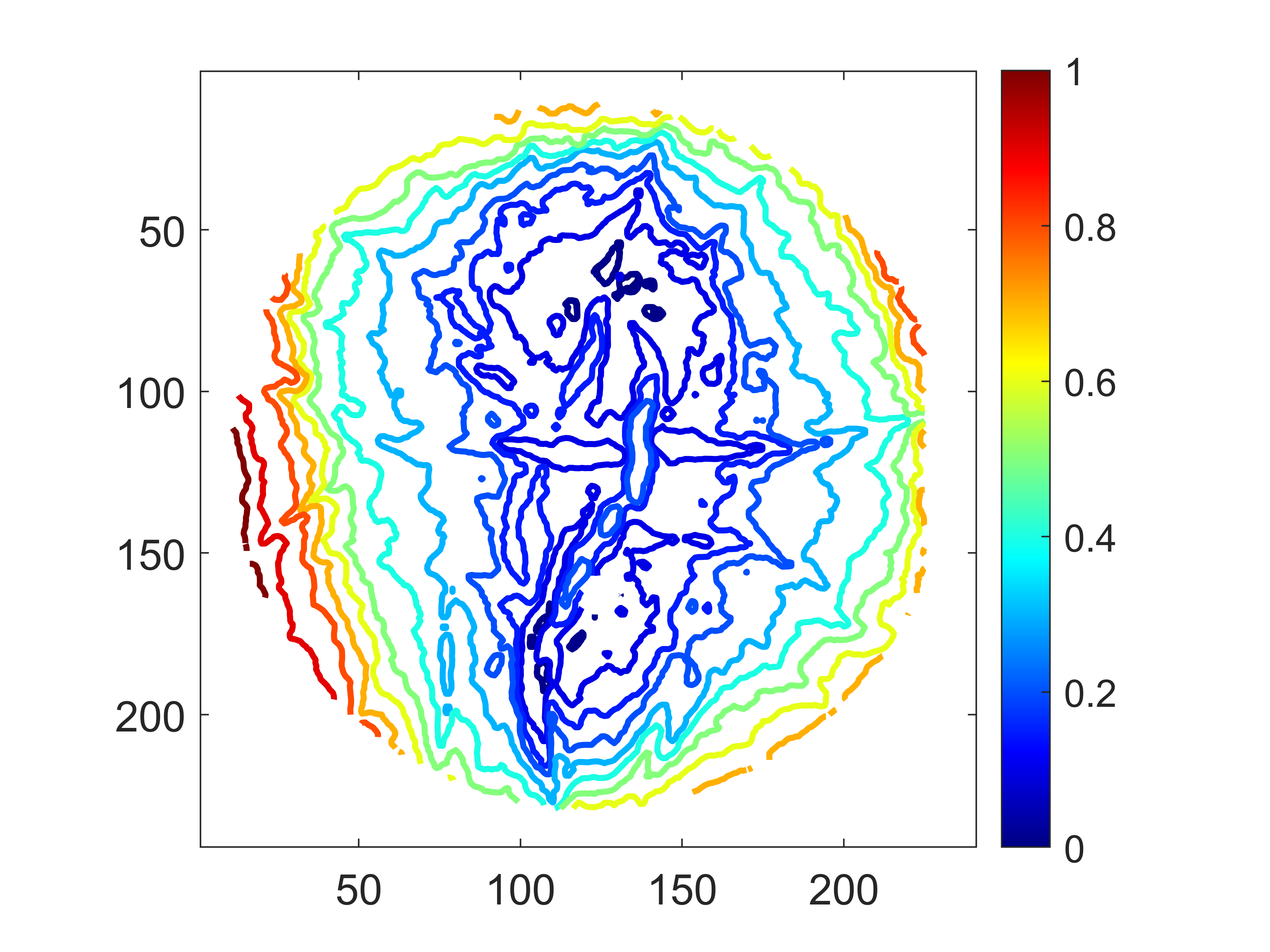

Supplement: S1 File — (ZIP) [file pone.0308204.s001.zip › S1 file. Birefringence Images/B-PK/30 degee/2391OD/unwppedISOCHconou.tif]

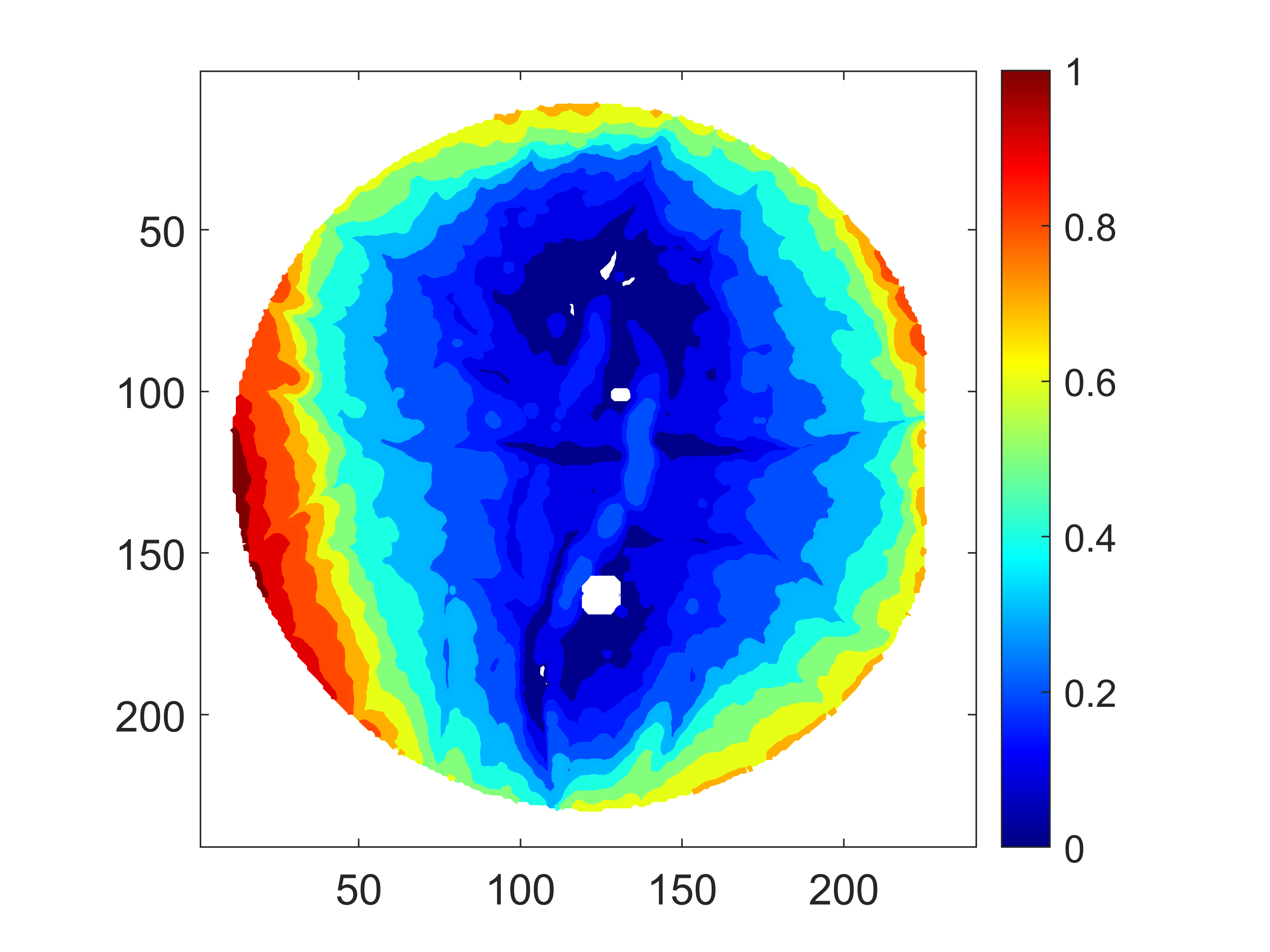

Supplement: S1 File — (ZIP) [file pone.0308204.s001.zip › S1 file. Birefringence Images/B-PK/30 degee/2391OD/unwppedISOCHfill.tif]

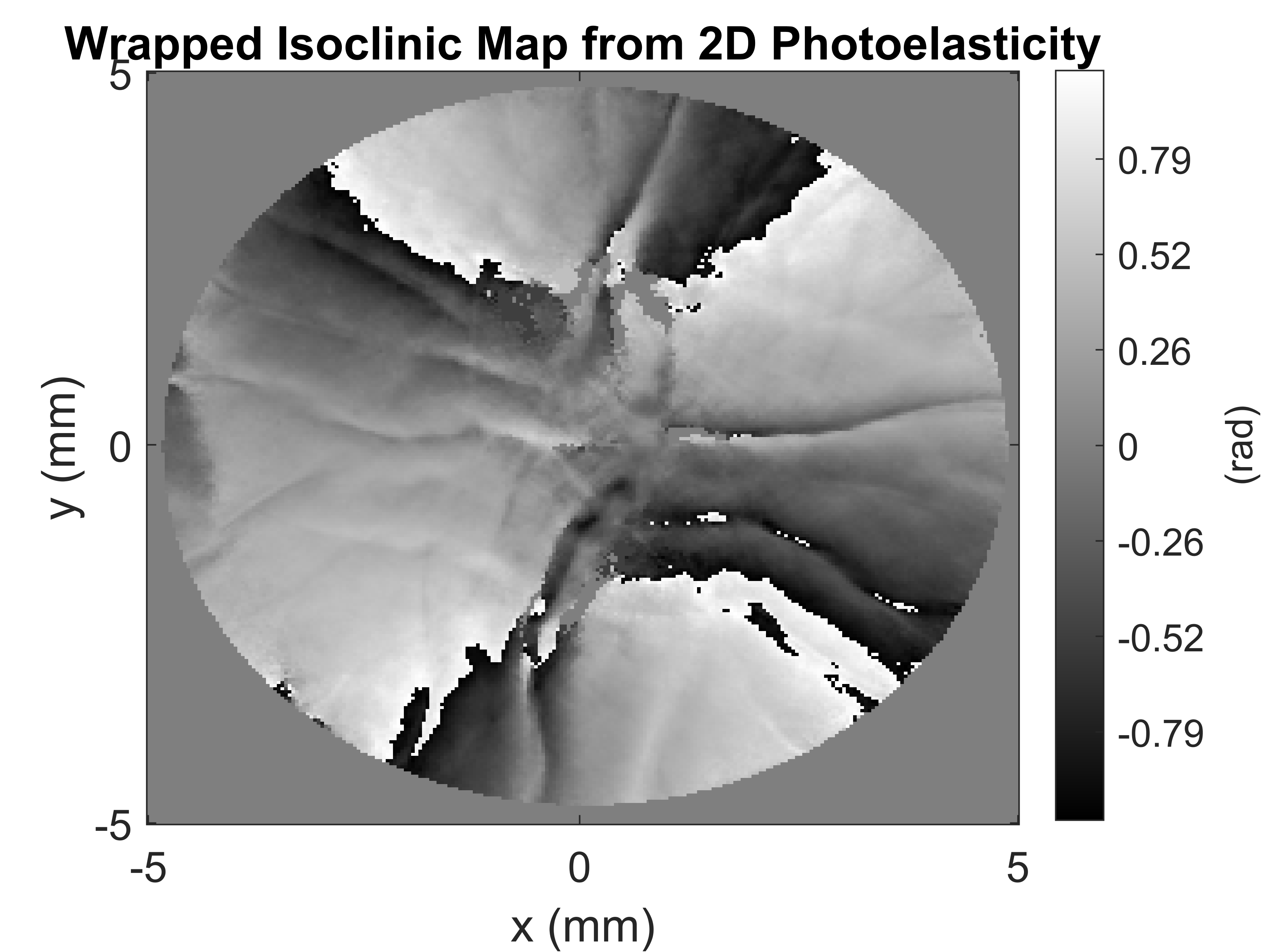

Supplement: S1 File — (ZIP) [file pone.0308204.s001.zip › S1 file. Birefringence Images/B-PK/30 degee/2391OD/wappedISO.tif]

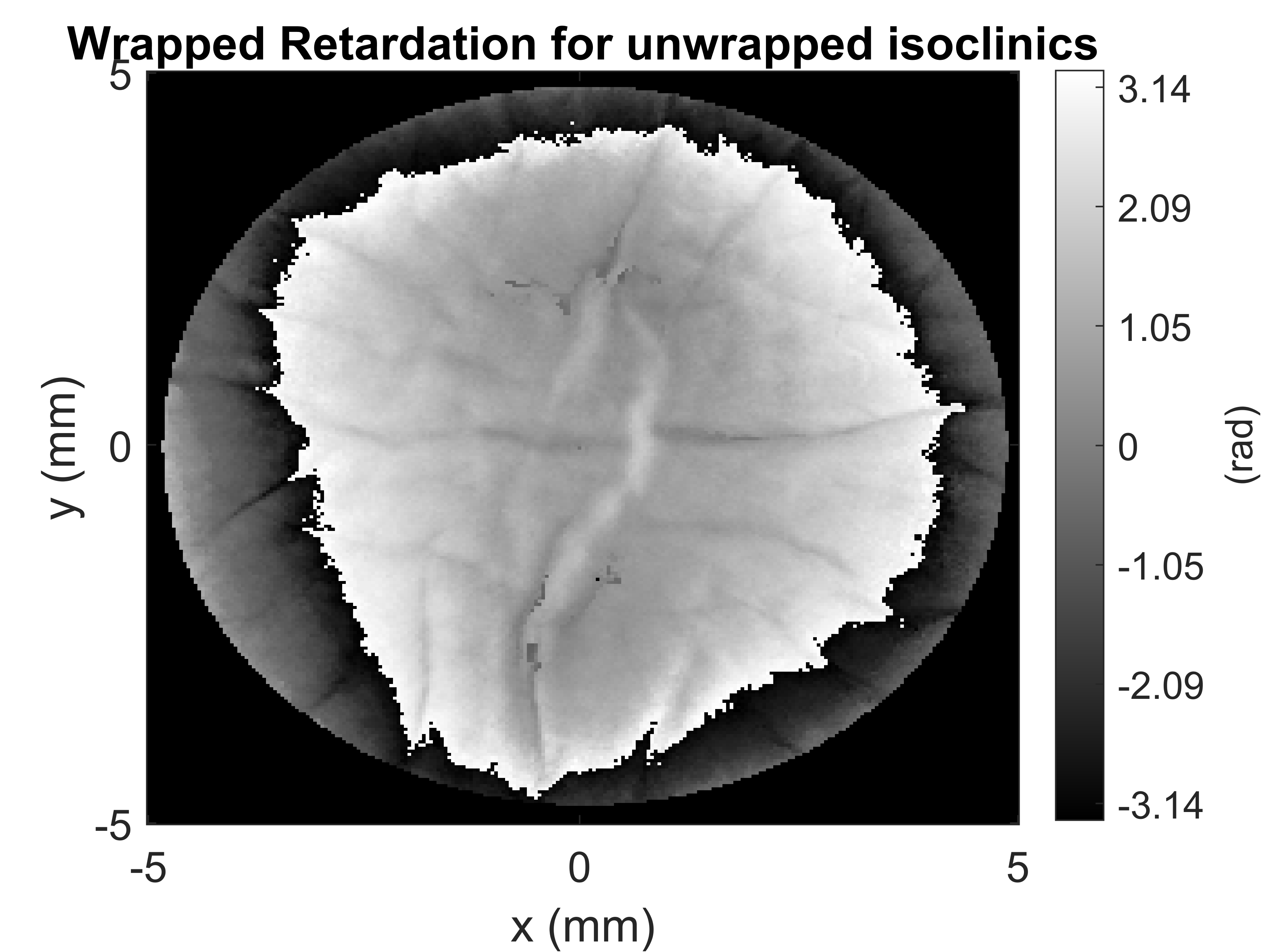

Supplement: S1 File — (ZIP) [file pone.0308204.s001.zip › S1 file. Birefringence Images/B-PK/30 degee/2391OD/wppedISOCHunwppedISO.tif]

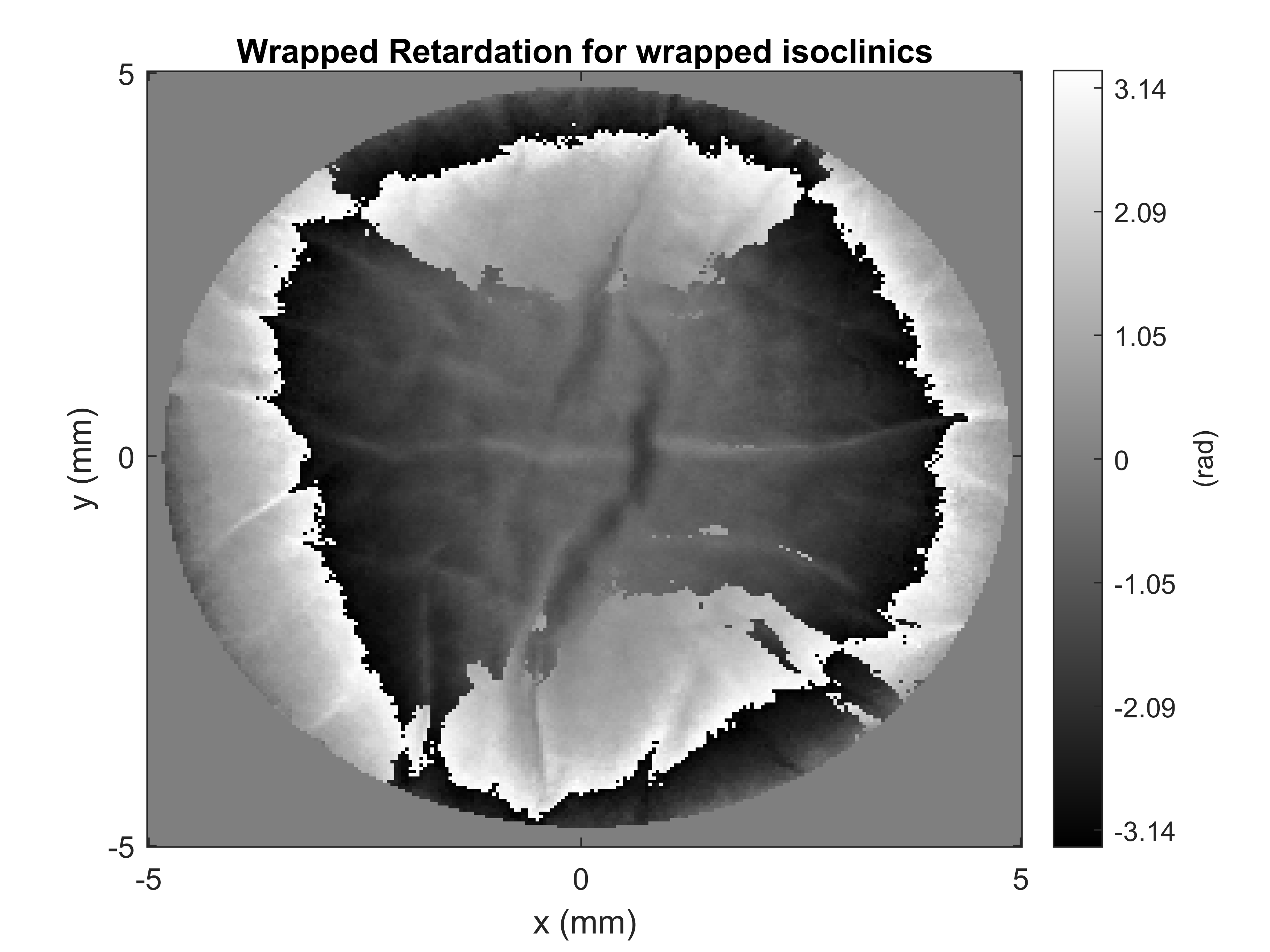

Supplement: S1 File — (ZIP) [file pone.0308204.s001.zip › S1 file. Birefringence Images/B-PK/30 degee/2391OD/wppedISOwppedISOCH.tif]

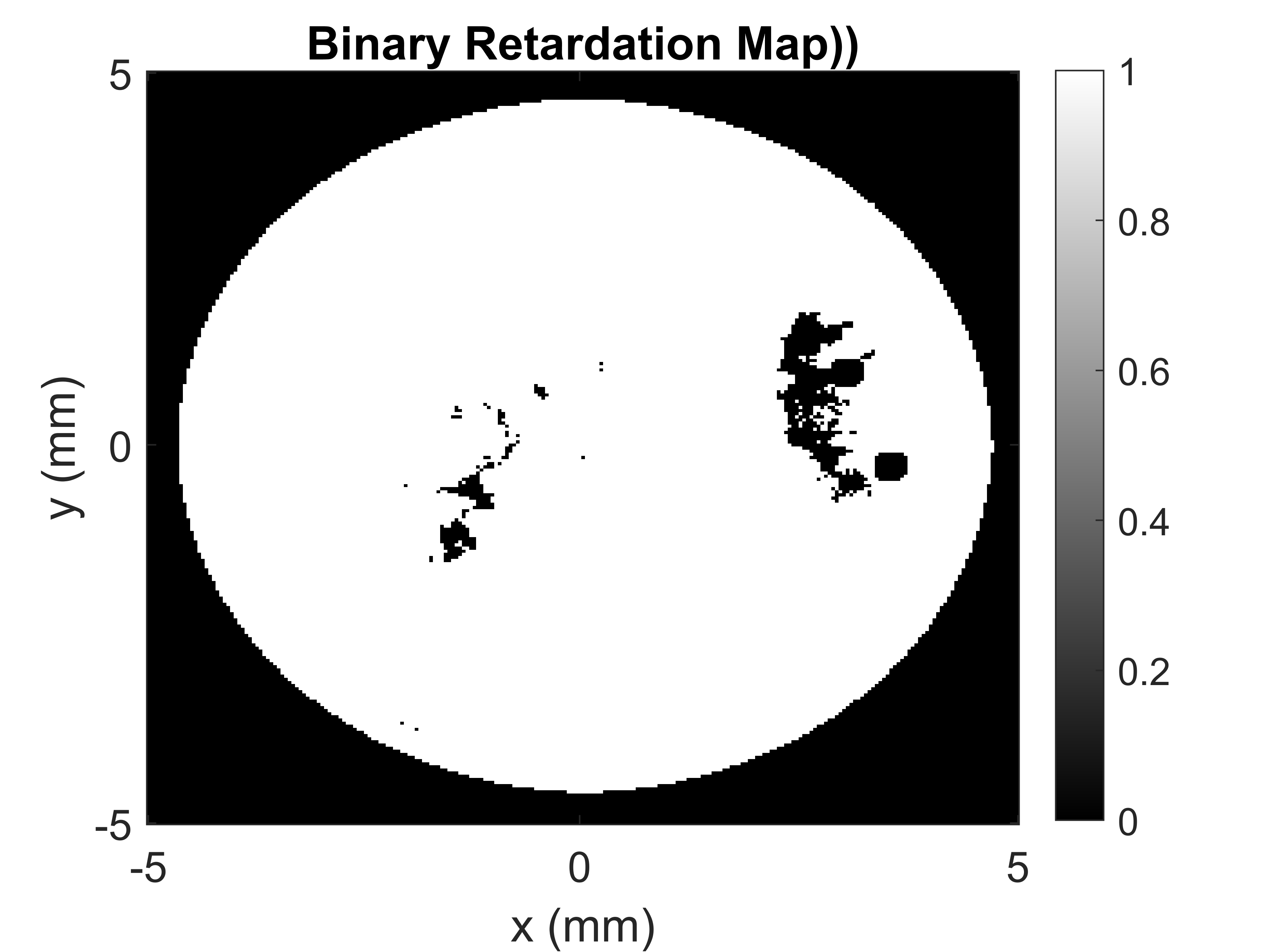

Supplement: S1 File — (ZIP) [file pone.0308204.s001.zip › S1 file. Birefringence Images/B-PK/45 degee/2751OD/isoopicpoins.tif]

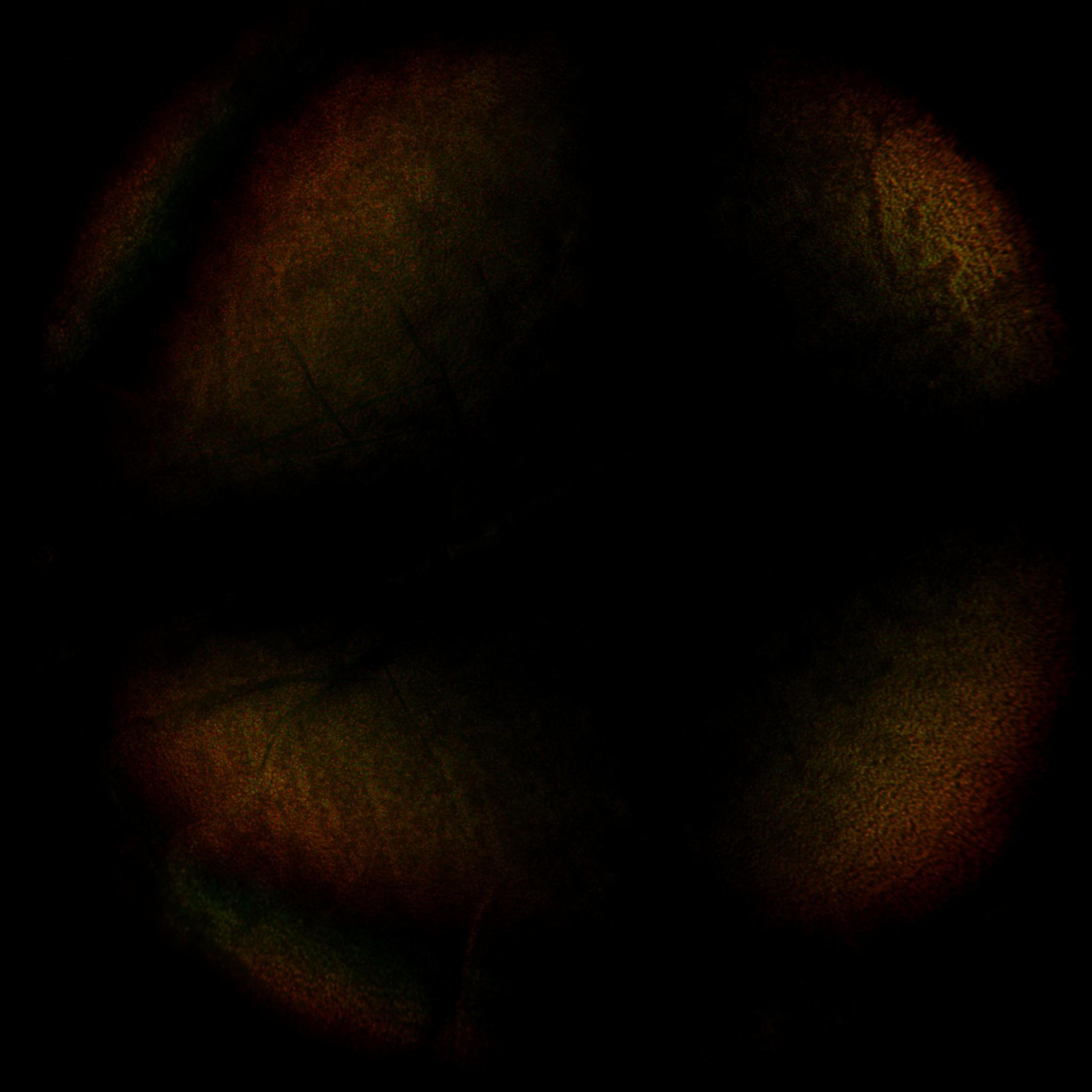

Supplement: S1 File — (ZIP) [file pone.0308204.s001.zip › S1 file. Birefringence Images/B-PK/45 degee/2751OD/IW1.jpg]

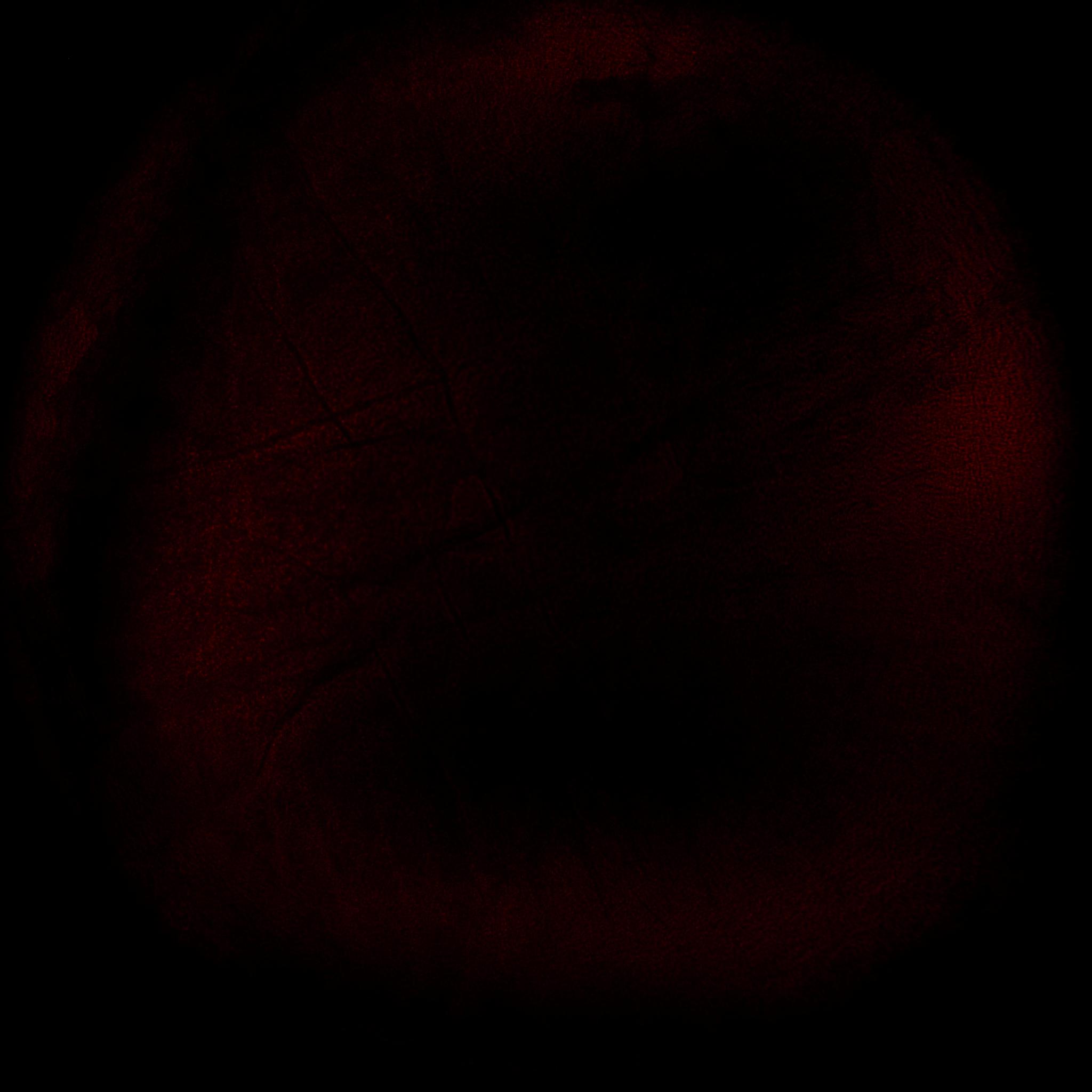

Supplement: S1 File — (ZIP) [file pone.0308204.s001.zip › S1 file. Birefringence Images/B-PK/45 degee/2751OD/IW10.jpg]

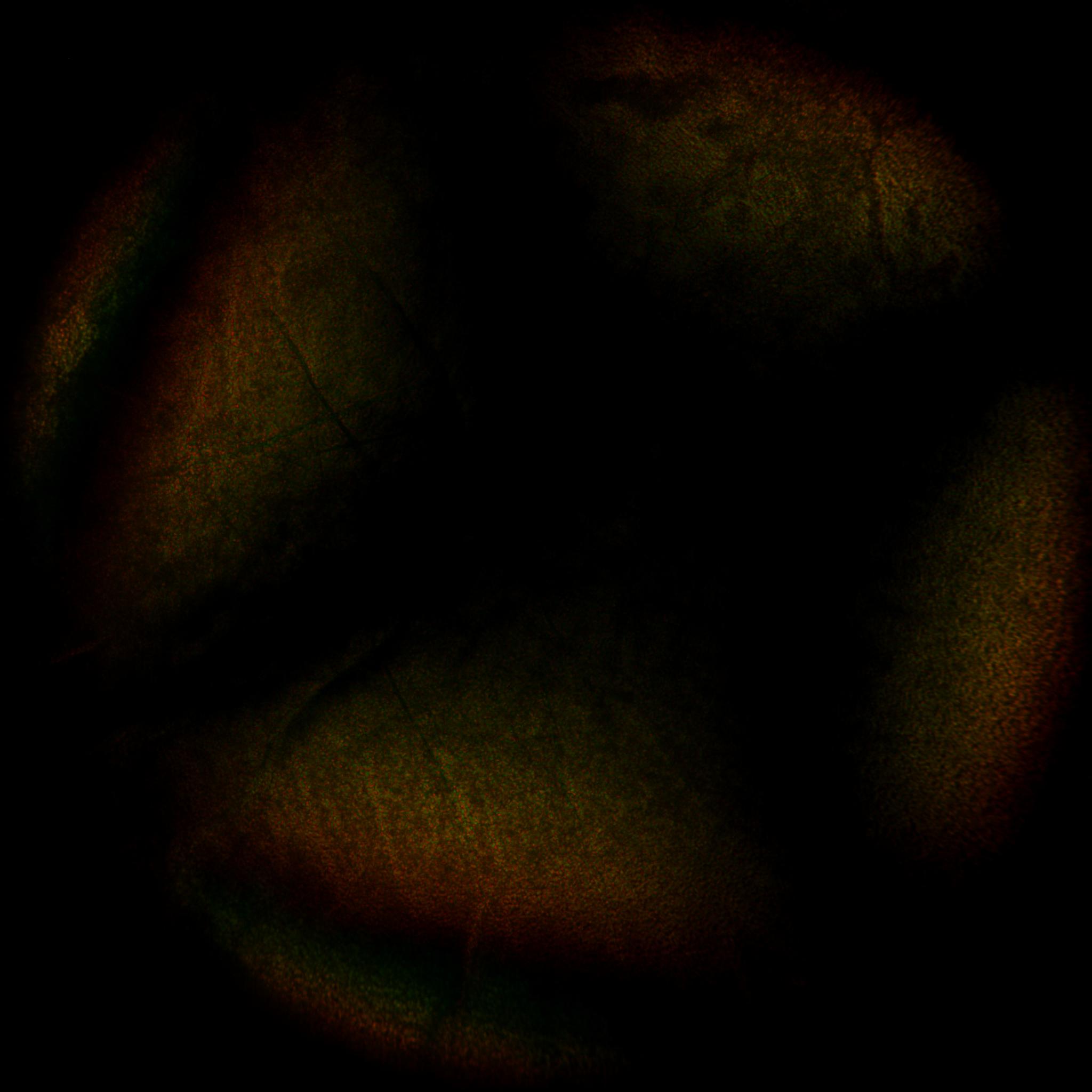

Supplement: S1 File — (ZIP) [file pone.0308204.s001.zip › S1 file. Birefringence Images/B-PK/45 degee/2751OD/IW2.jpg]

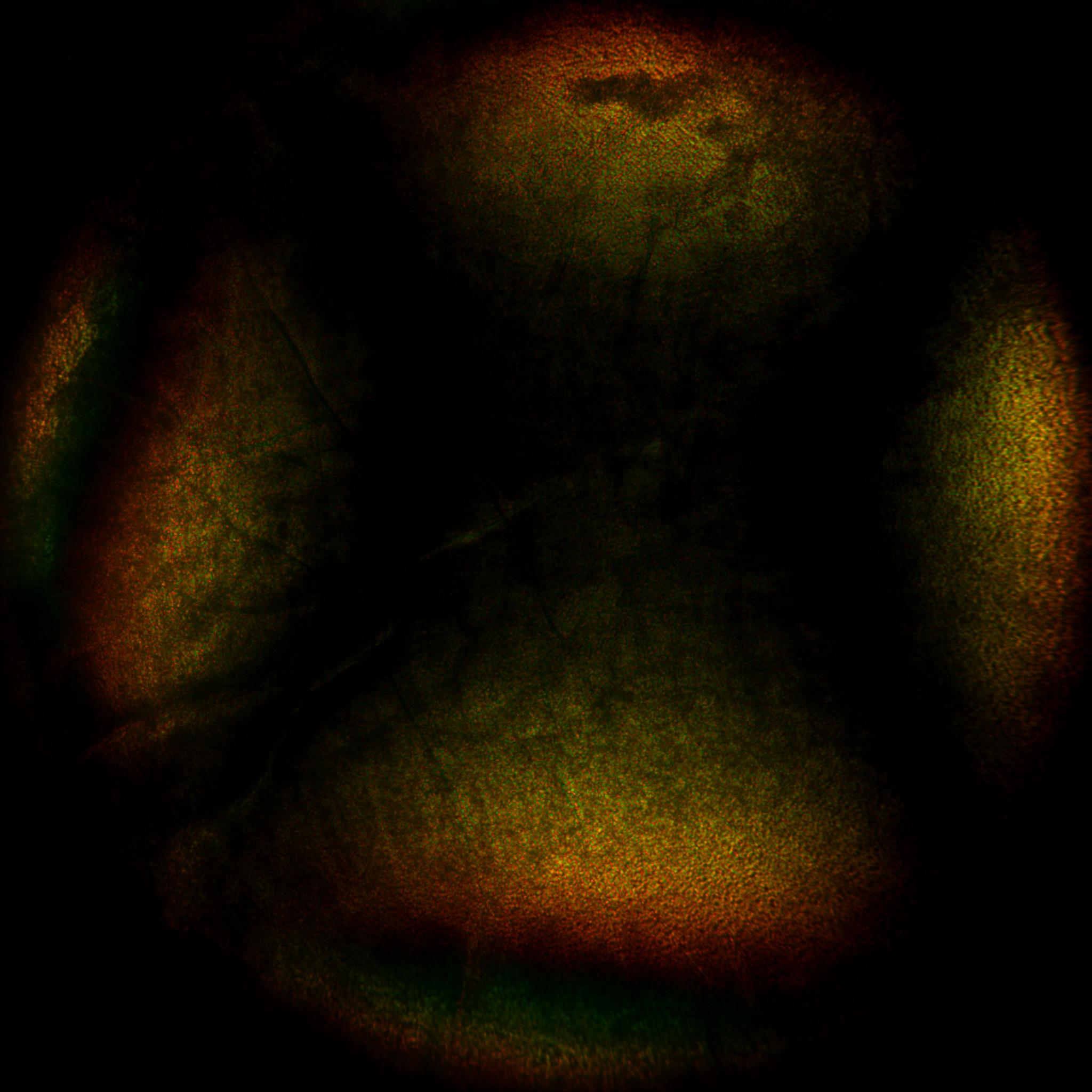

Supplement: S1 File — (ZIP) [file pone.0308204.s001.zip › S1 file. Birefringence Images/B-PK/45 degee/2751OD/IW3.jpg]

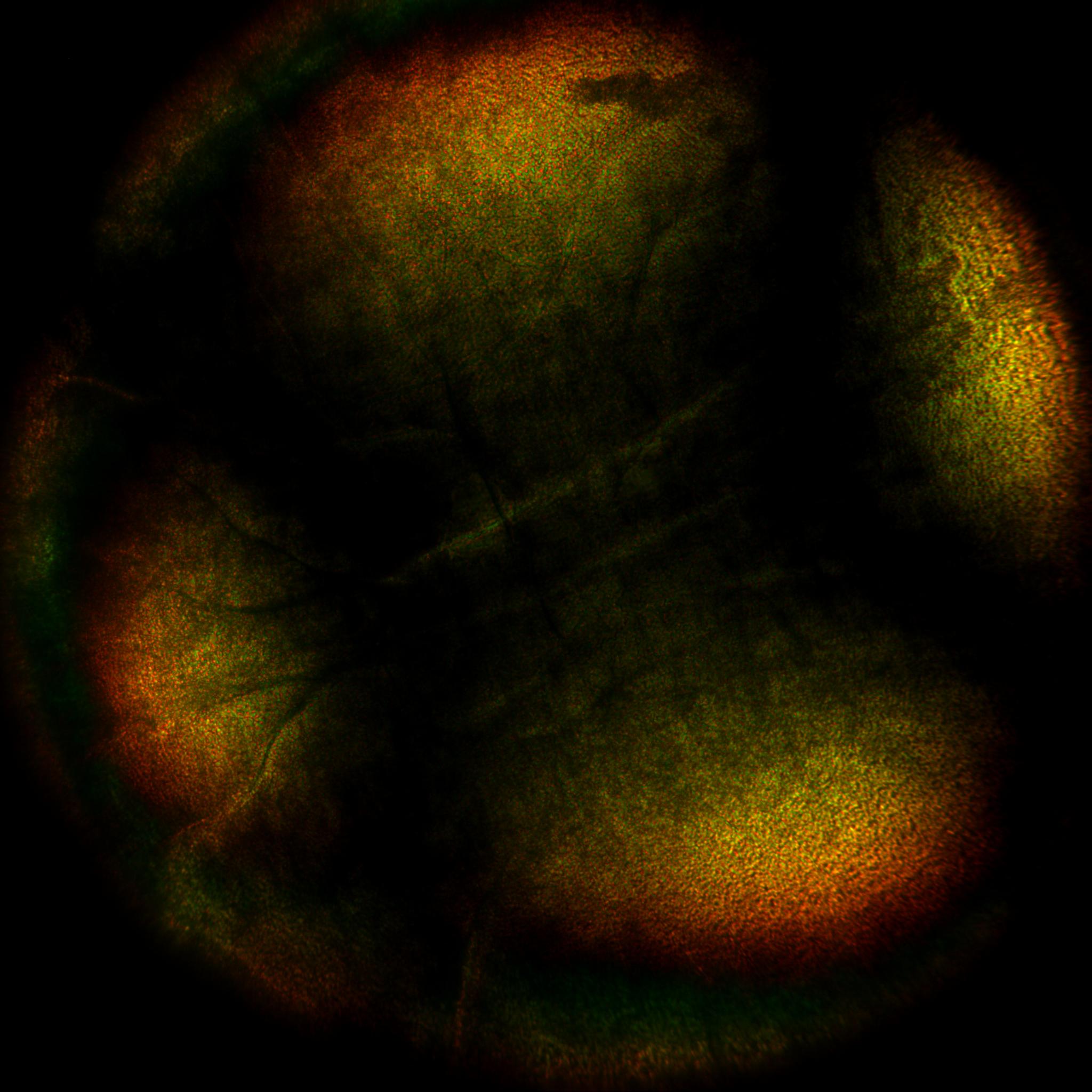

Supplement: S1 File — (ZIP) [file pone.0308204.s001.zip › S1 file. Birefringence Images/B-PK/45 degee/2751OD/IW4.jpg]

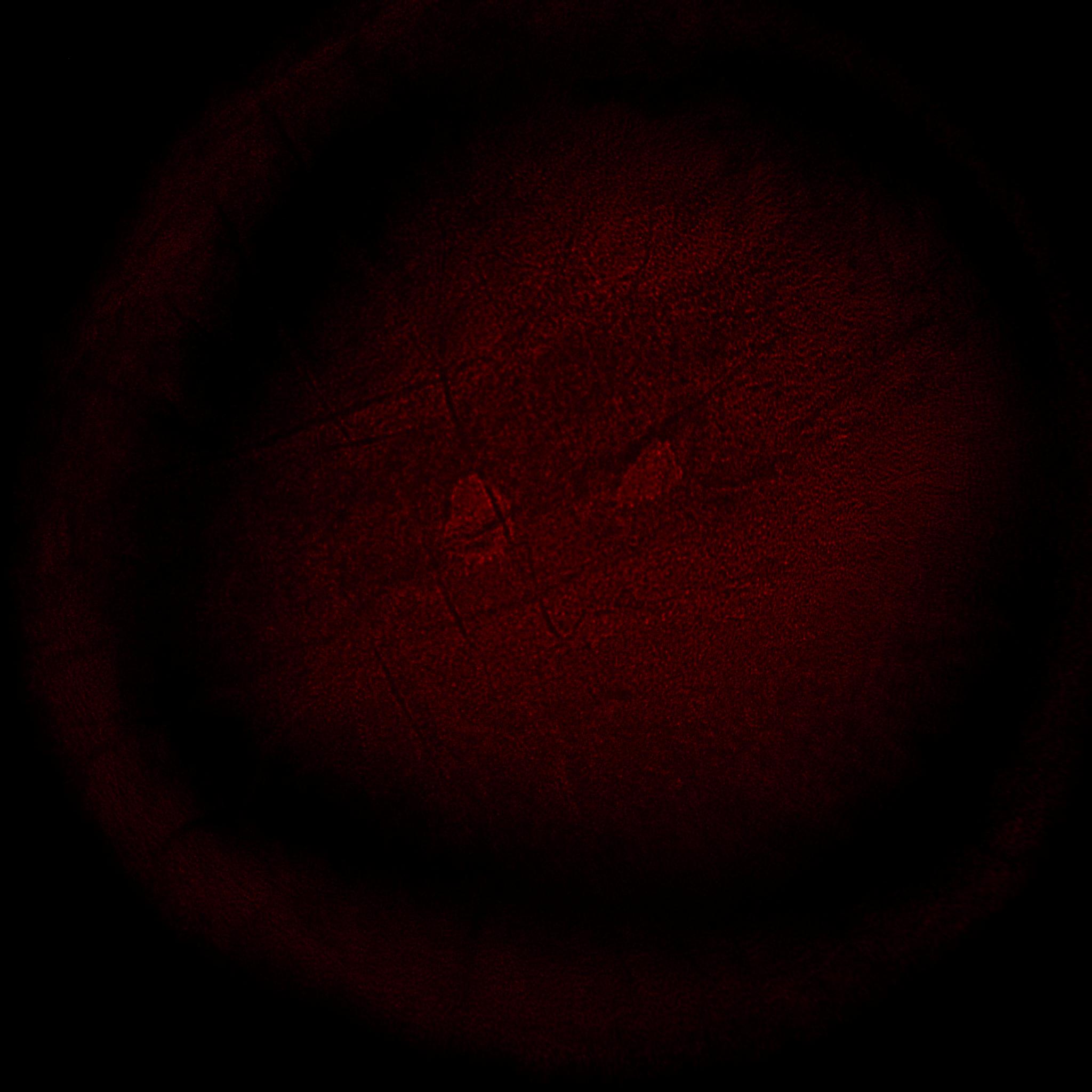

Supplement: S1 File — (ZIP) [file pone.0308204.s001.zip › S1 file. Birefringence Images/B-PK/45 degee/2751OD/IW5.jpg]

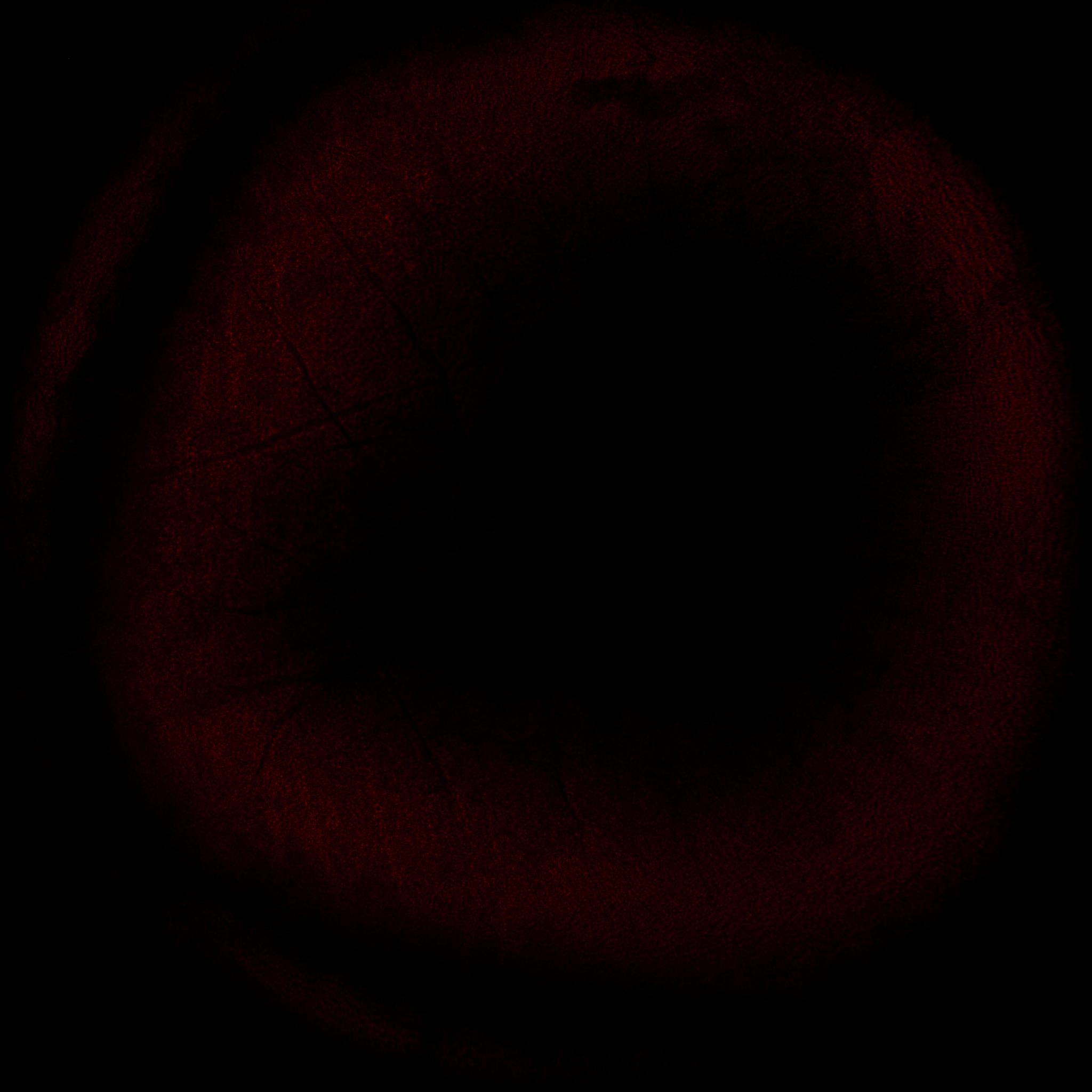

Supplement: S1 File — (ZIP) [file pone.0308204.s001.zip › S1 file. Birefringence Images/B-PK/45 degee/2751OD/IW6.jpg]

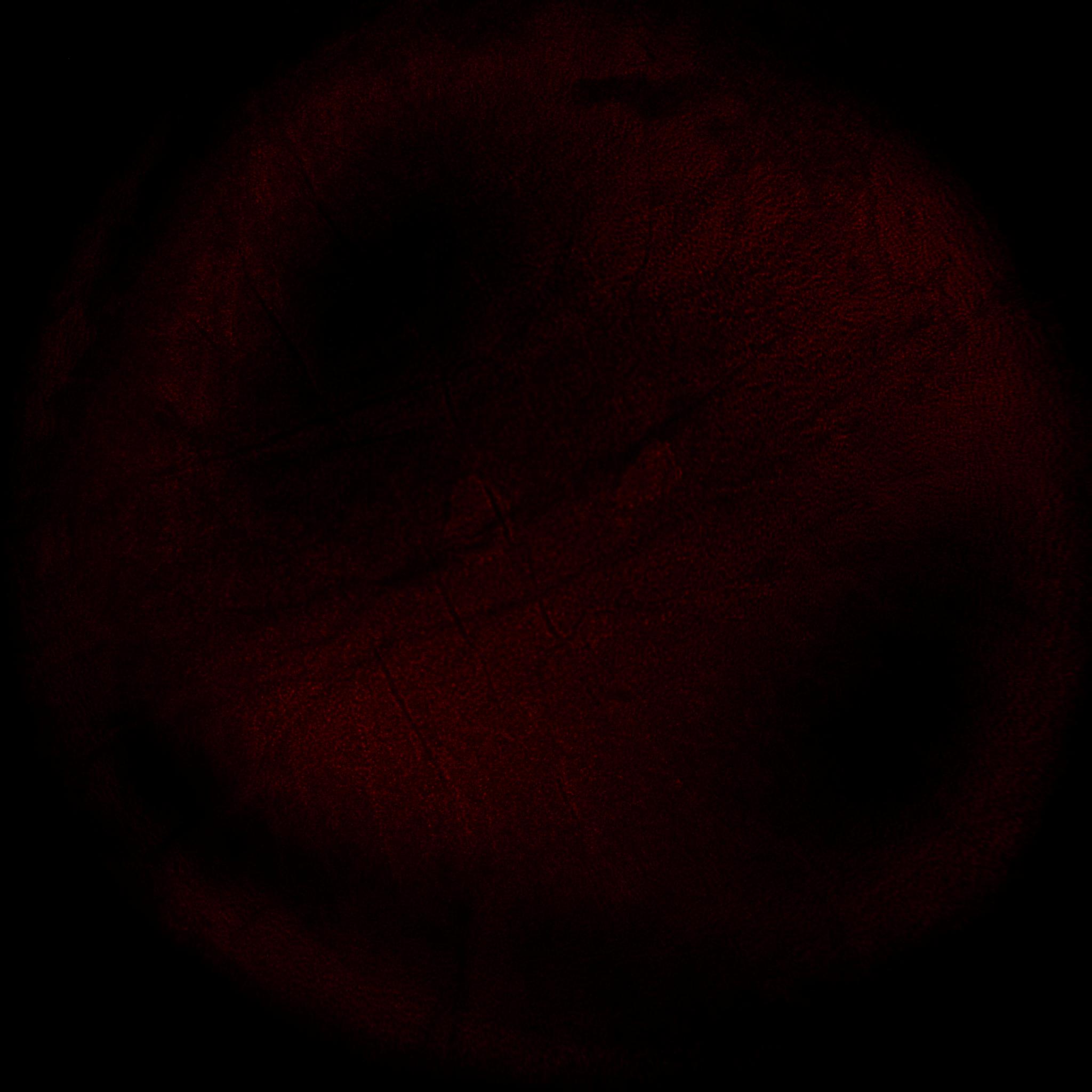

Supplement: S1 File — (ZIP) [file pone.0308204.s001.zip › S1 file. Birefringence Images/B-PK/45 degee/2751OD/IW7.jpg]

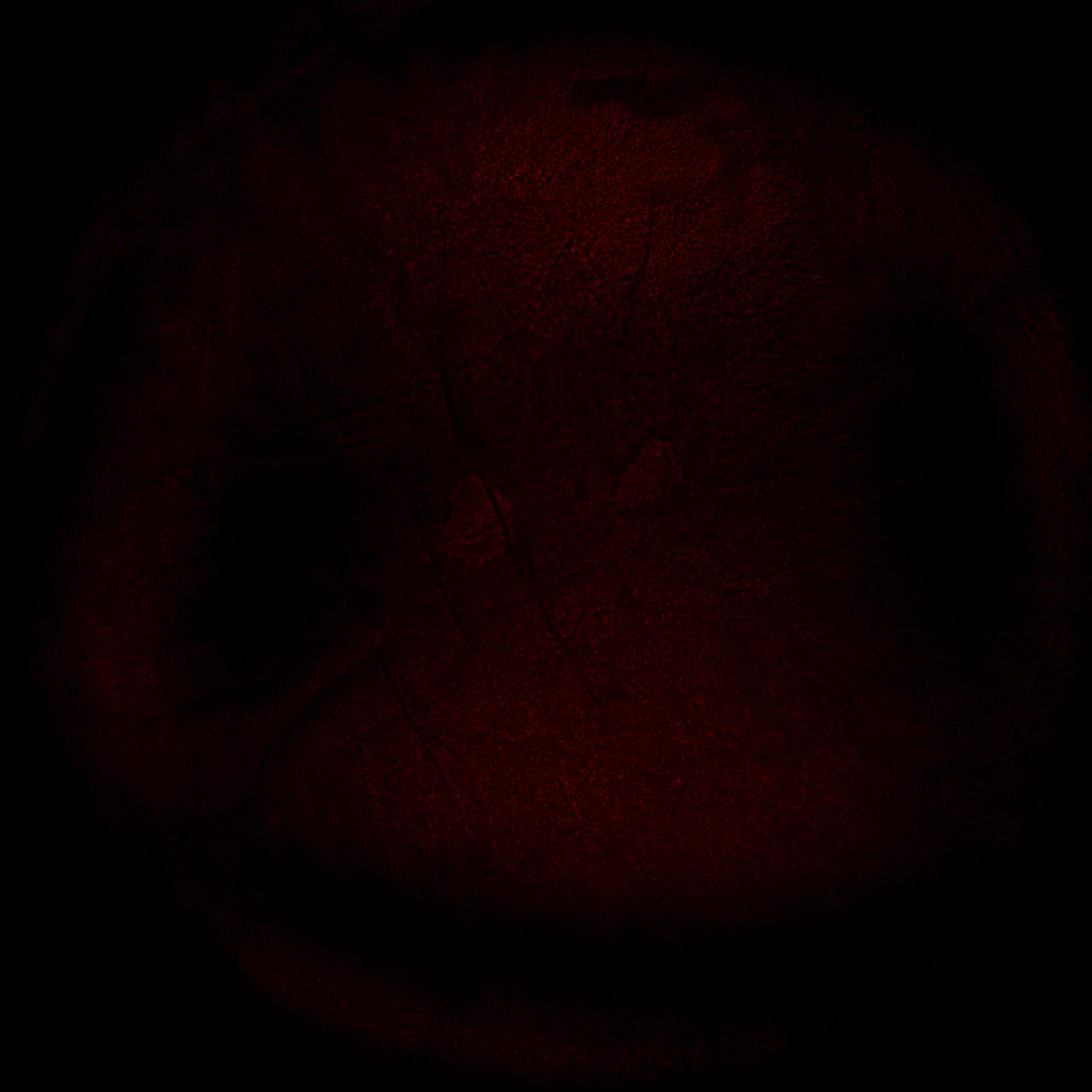

Supplement: S1 File — (ZIP) [file pone.0308204.s001.zip › S1 file. Birefringence Images/B-PK/45 degee/2751OD/IW8.jpg]

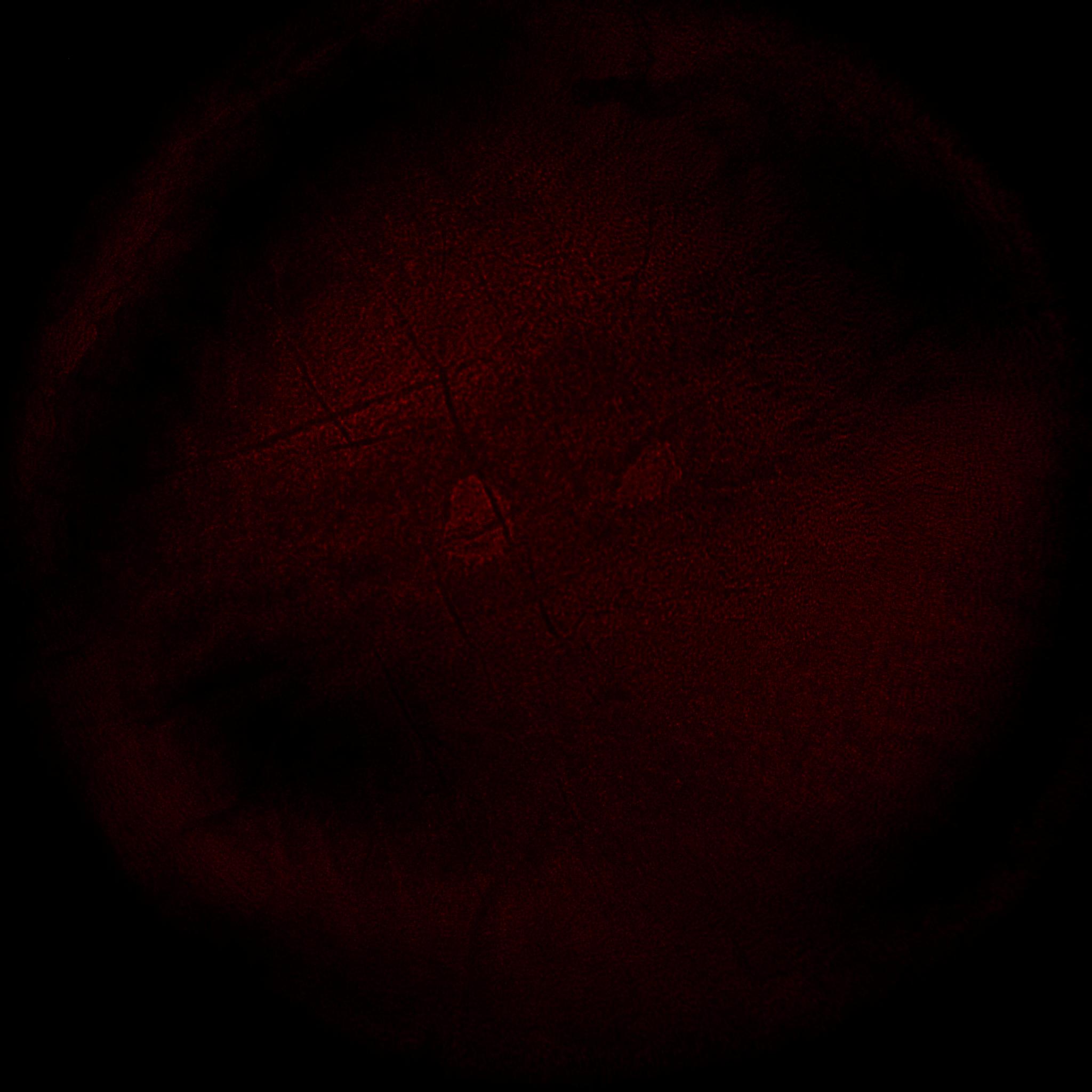

Supplement: S1 File — (ZIP) [file pone.0308204.s001.zip › S1 file. Birefringence Images/B-PK/45 degee/2751OD/IW9.jpg]

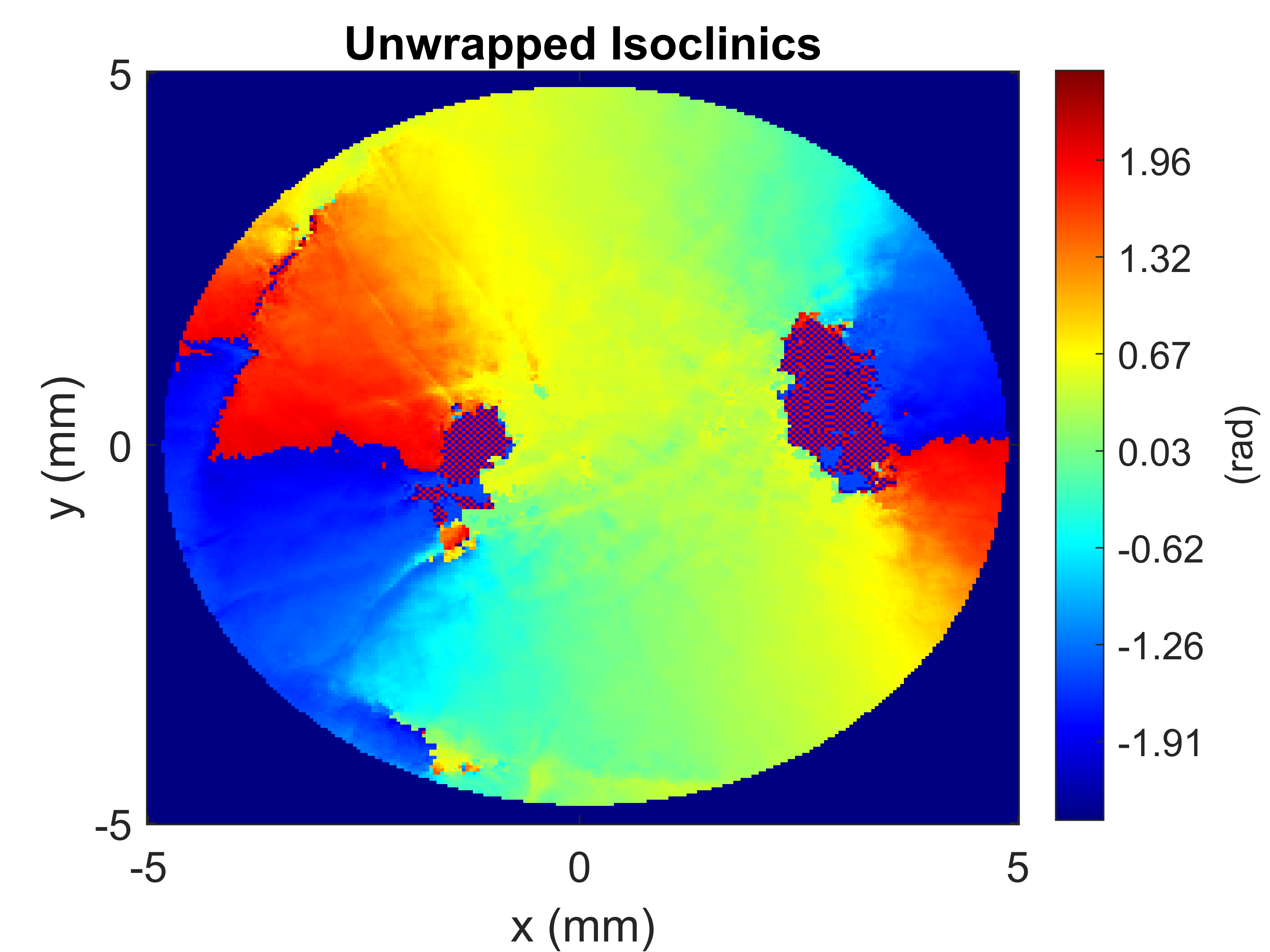

Supplement: S1 File — (ZIP) [file pone.0308204.s001.zip › S1 file. Birefringence Images/B-PK/45 degee/2751OD/unwppedISOCH.tif]

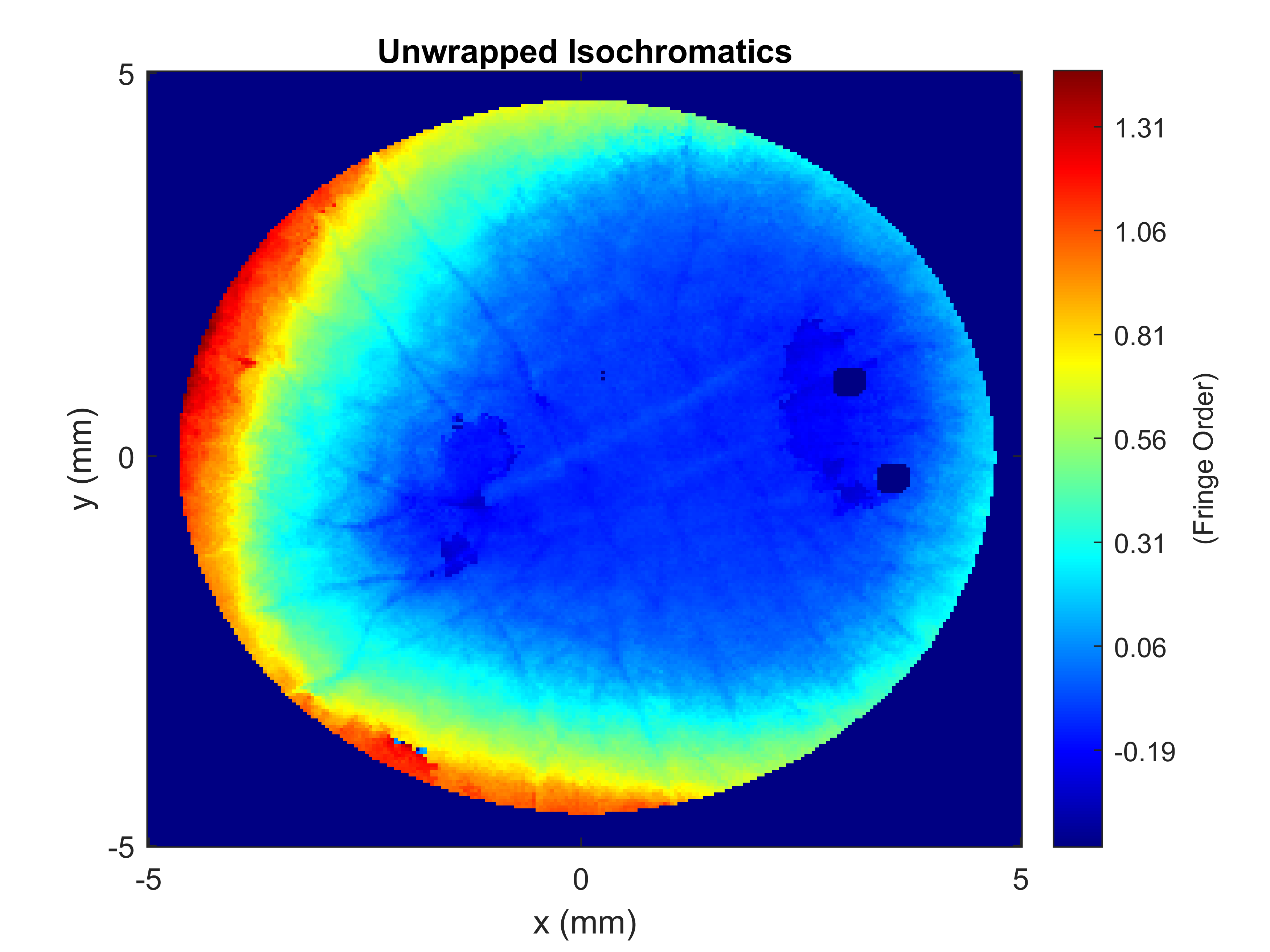

Supplement: S1 File — (ZIP) [file pone.0308204.s001.zip › S1 file. Birefringence Images/B-PK/45 degee/2751OD/unwppedISOCHcolo.tif]

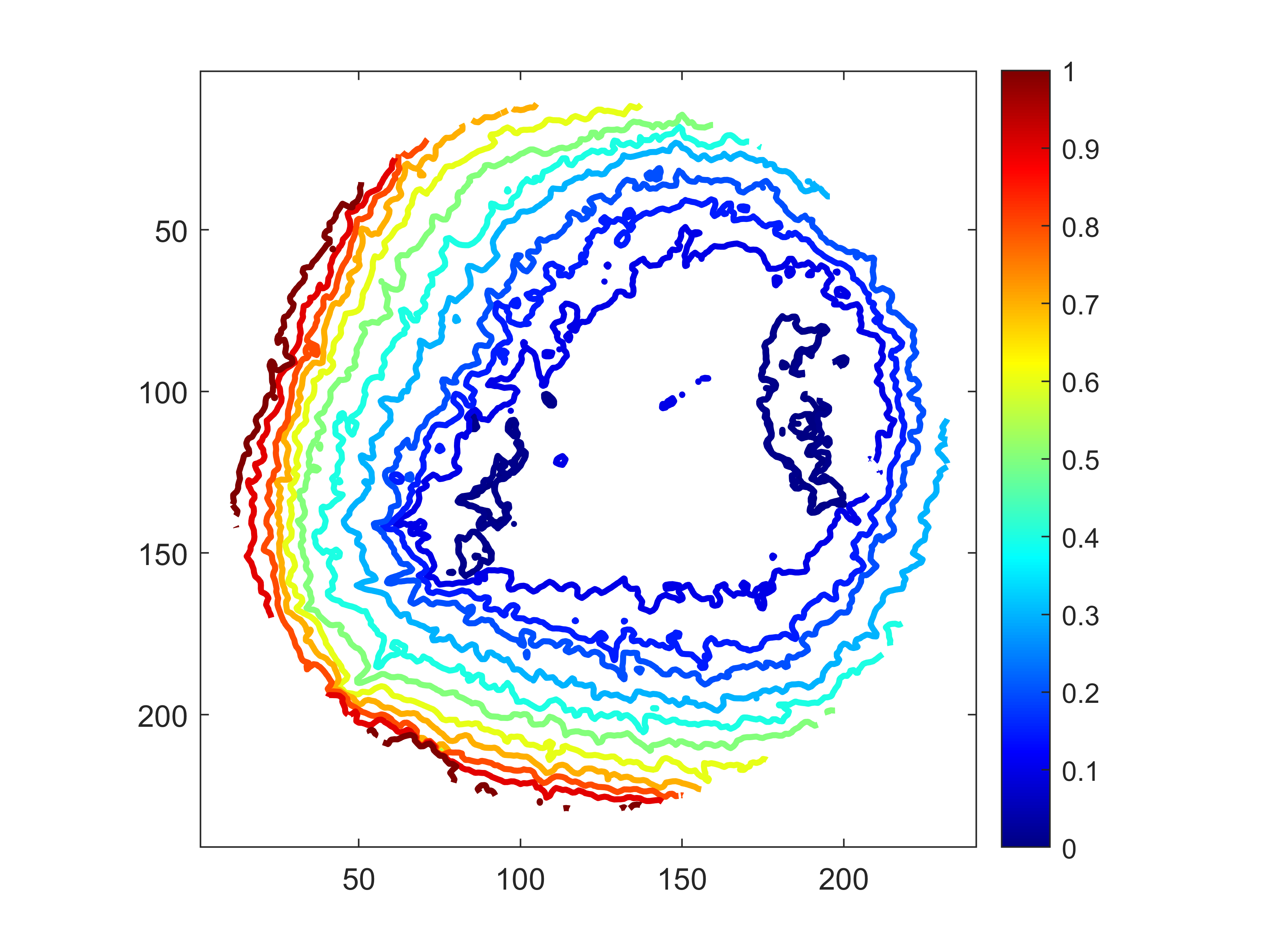

Supplement: S1 File — (ZIP) [file pone.0308204.s001.zip › S1 file. Birefringence Images/B-PK/45 degee/2751OD/unwppedISOCHcoloconou.tif]

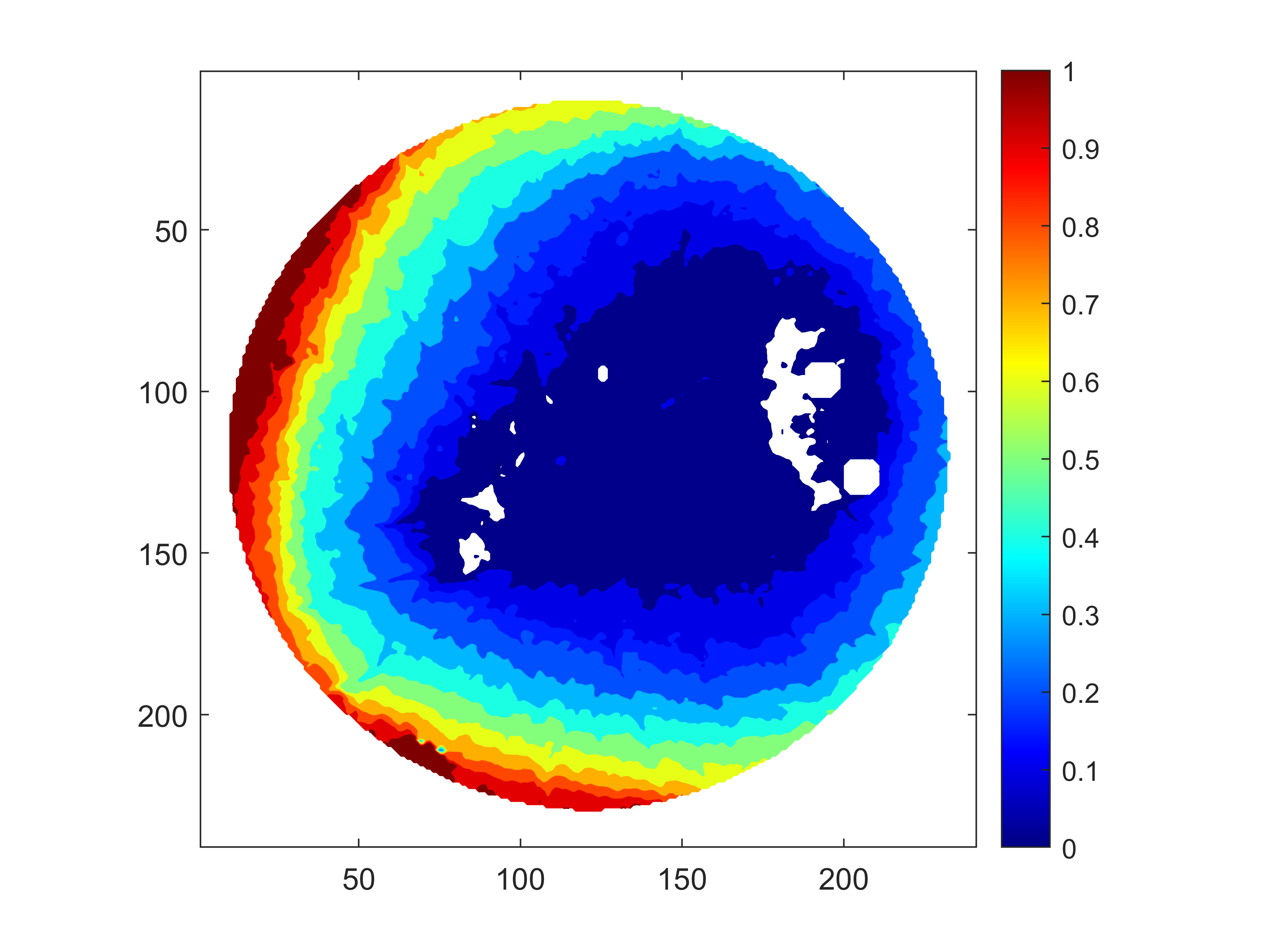

Supplement: S1 File — (ZIP) [file pone.0308204.s001.zip › S1 file. Birefringence Images/B-PK/45 degee/2751OD/unwppedISOCHcolofilled.tif]

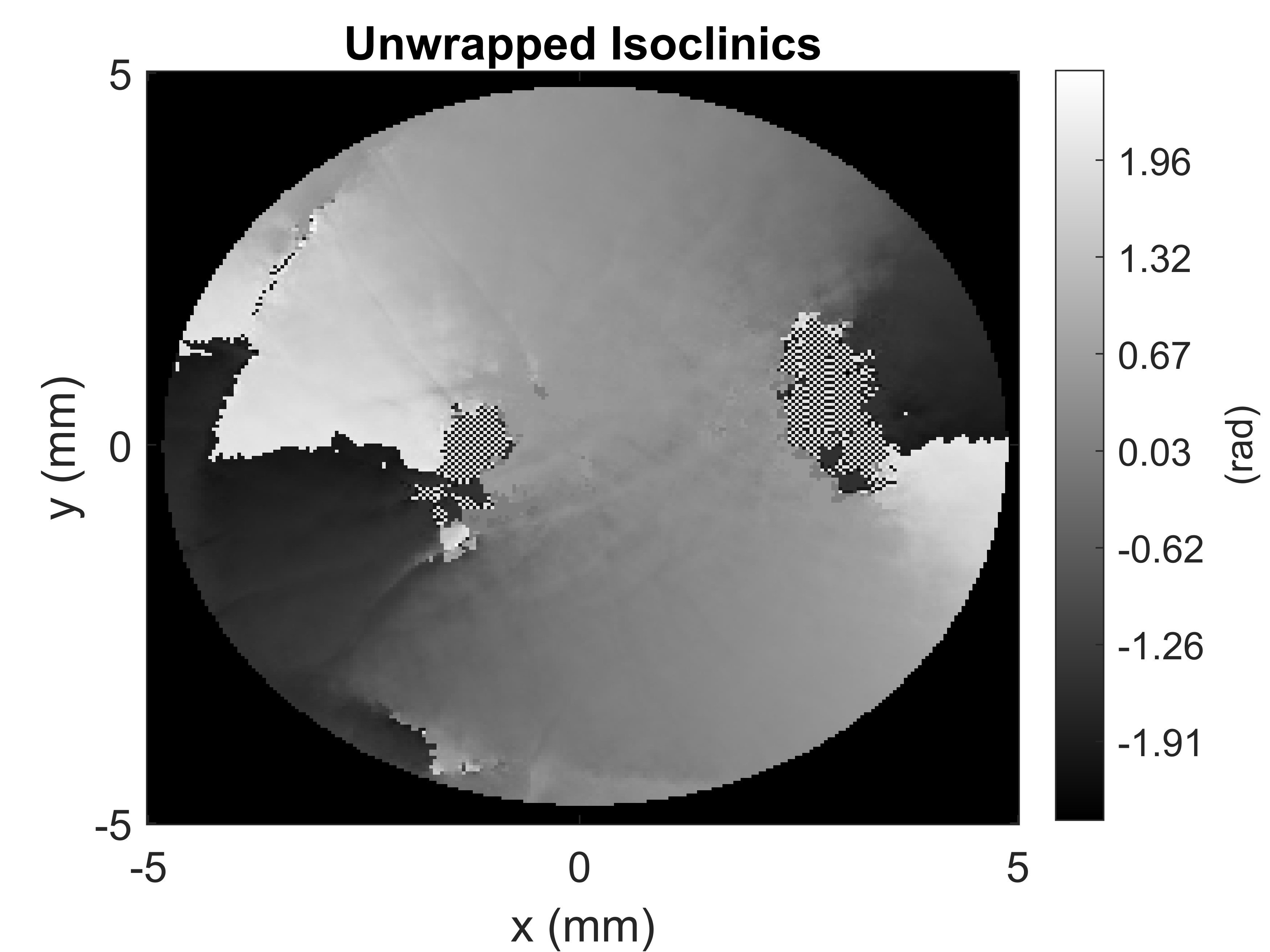

Supplement: S1 File — (ZIP) [file pone.0308204.s001.zip › S1 file. Birefringence Images/B-PK/45 degee/2751OD/unwppedISOCHgay.tif]

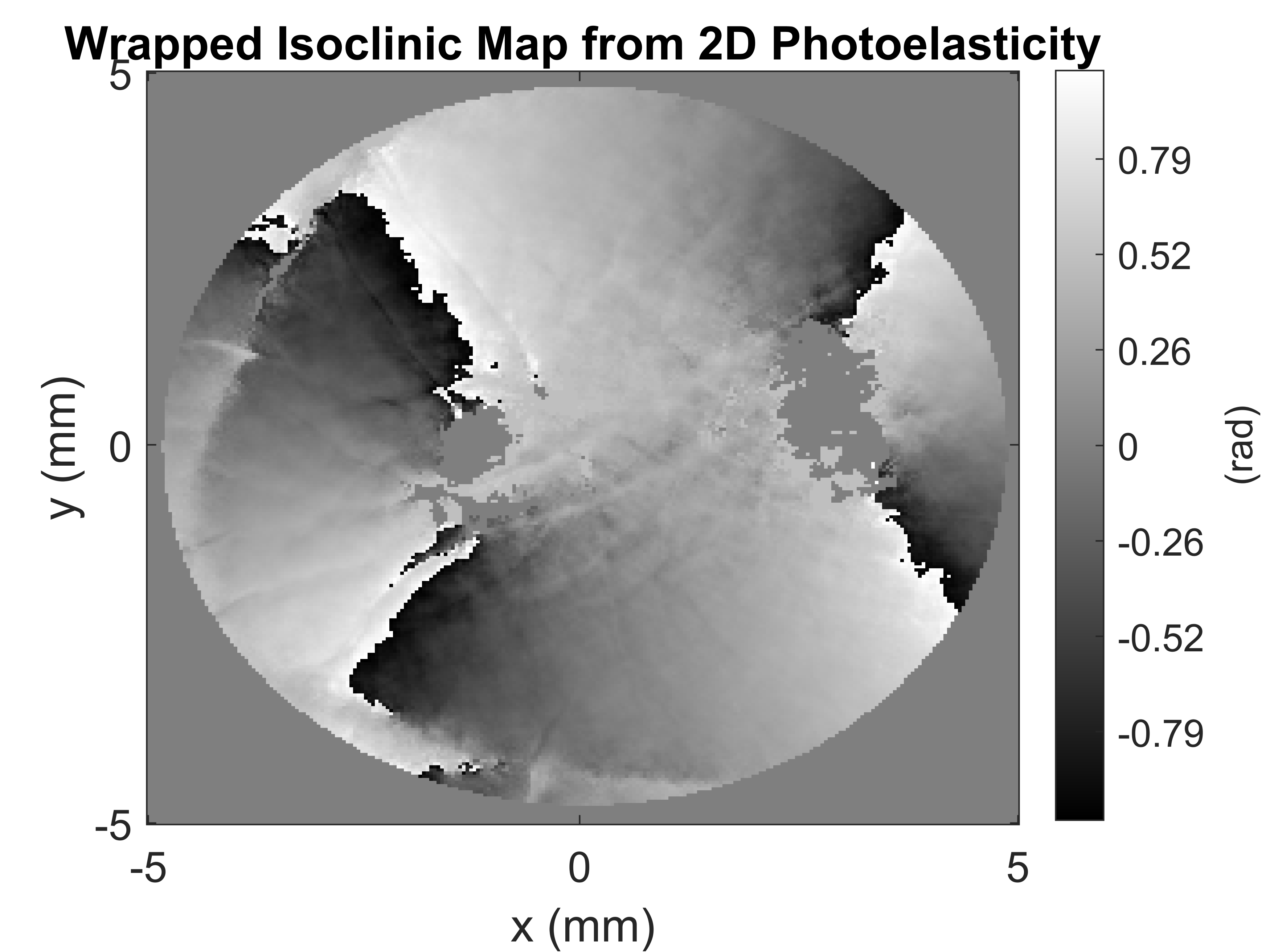

Supplement: S1 File — (ZIP) [file pone.0308204.s001.zip › S1 file. Birefringence Images/B-PK/45 degee/2751OD/wappedISOCH.tif]

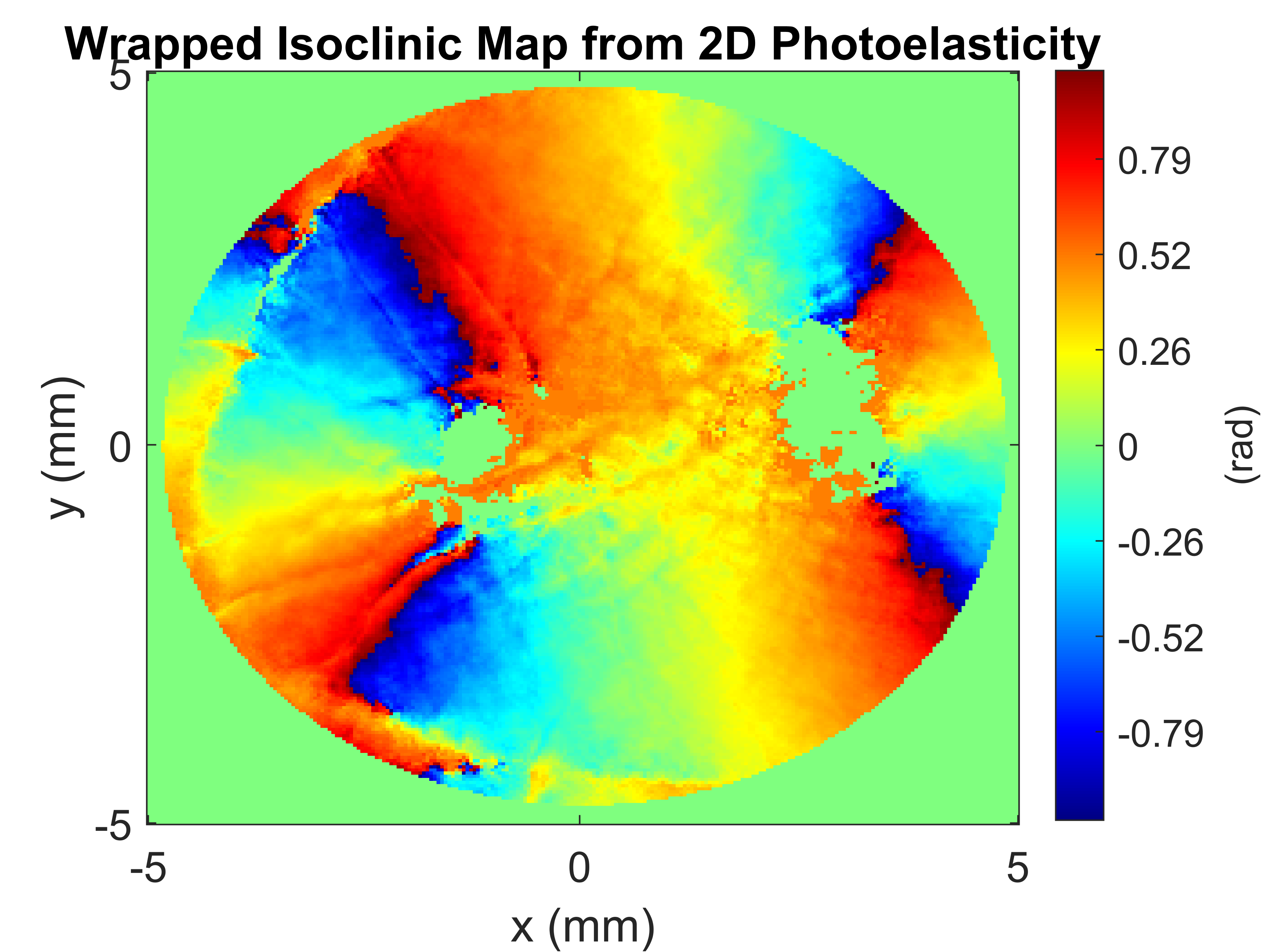

Supplement: S1 File — (ZIP) [file pone.0308204.s001.zip › S1 file. Birefringence Images/B-PK/45 degee/2751OD/wappedISOCHcolo.tif]

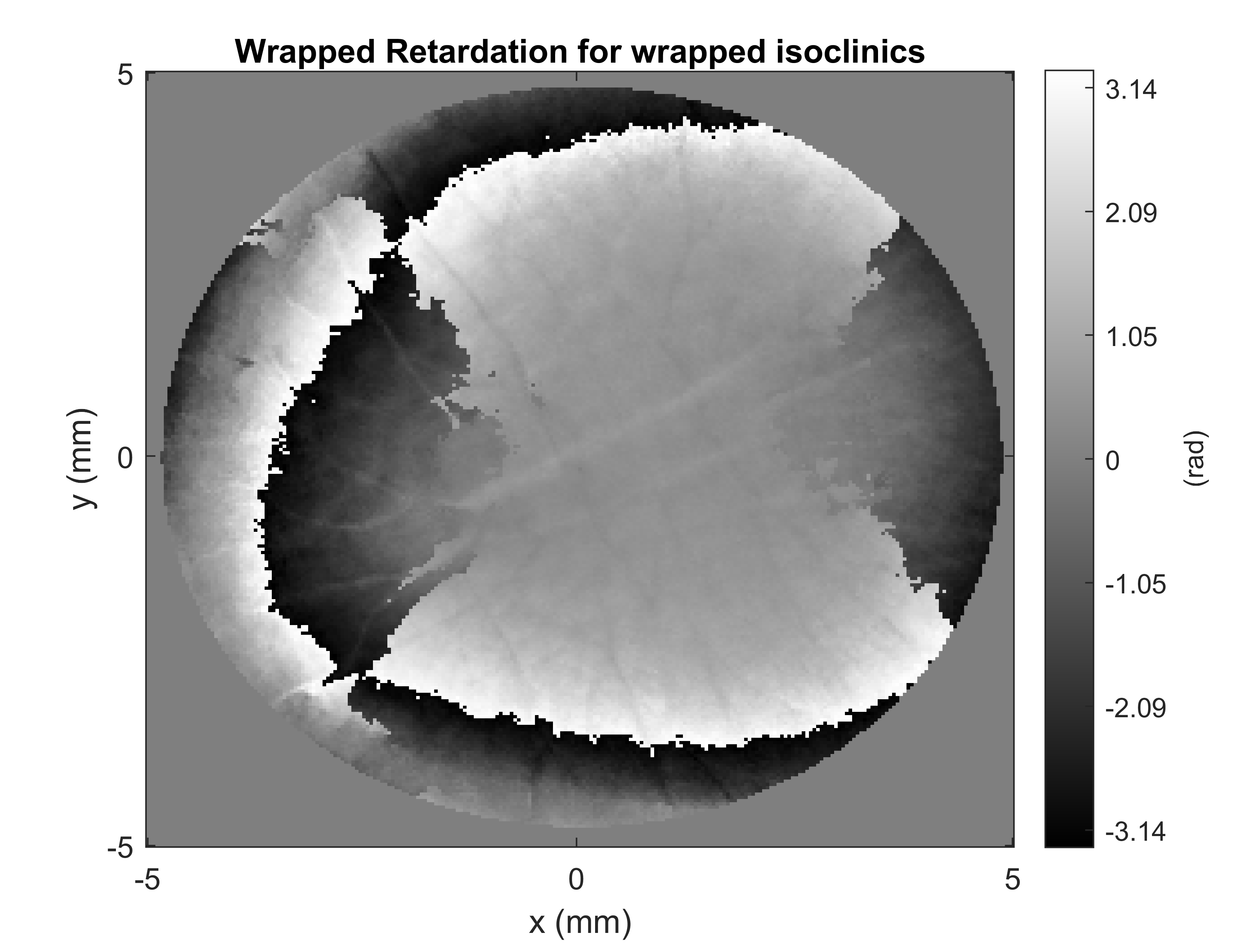

Supplement: S1 File — (ZIP) [file pone.0308204.s001.zip › S1 file. Birefringence Images/B-PK/45 degee/2751OD/wppedISOCHwppedISO.tif]

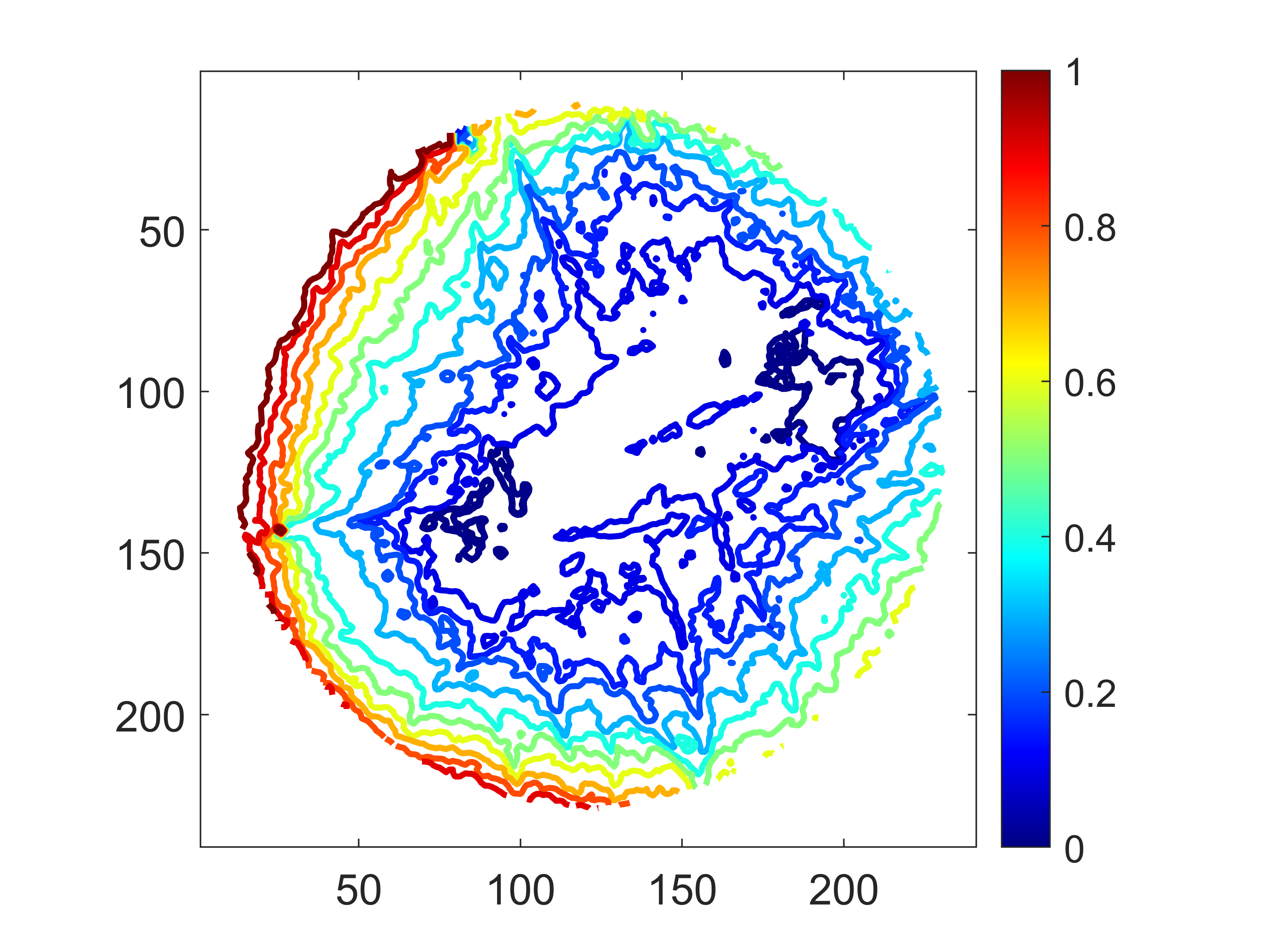

Supplement: S1 File — (ZIP) [file pone.0308204.s001.zip › S1 file. Birefringence Images/B-PK/60 degree/2693OS/contous.tif]

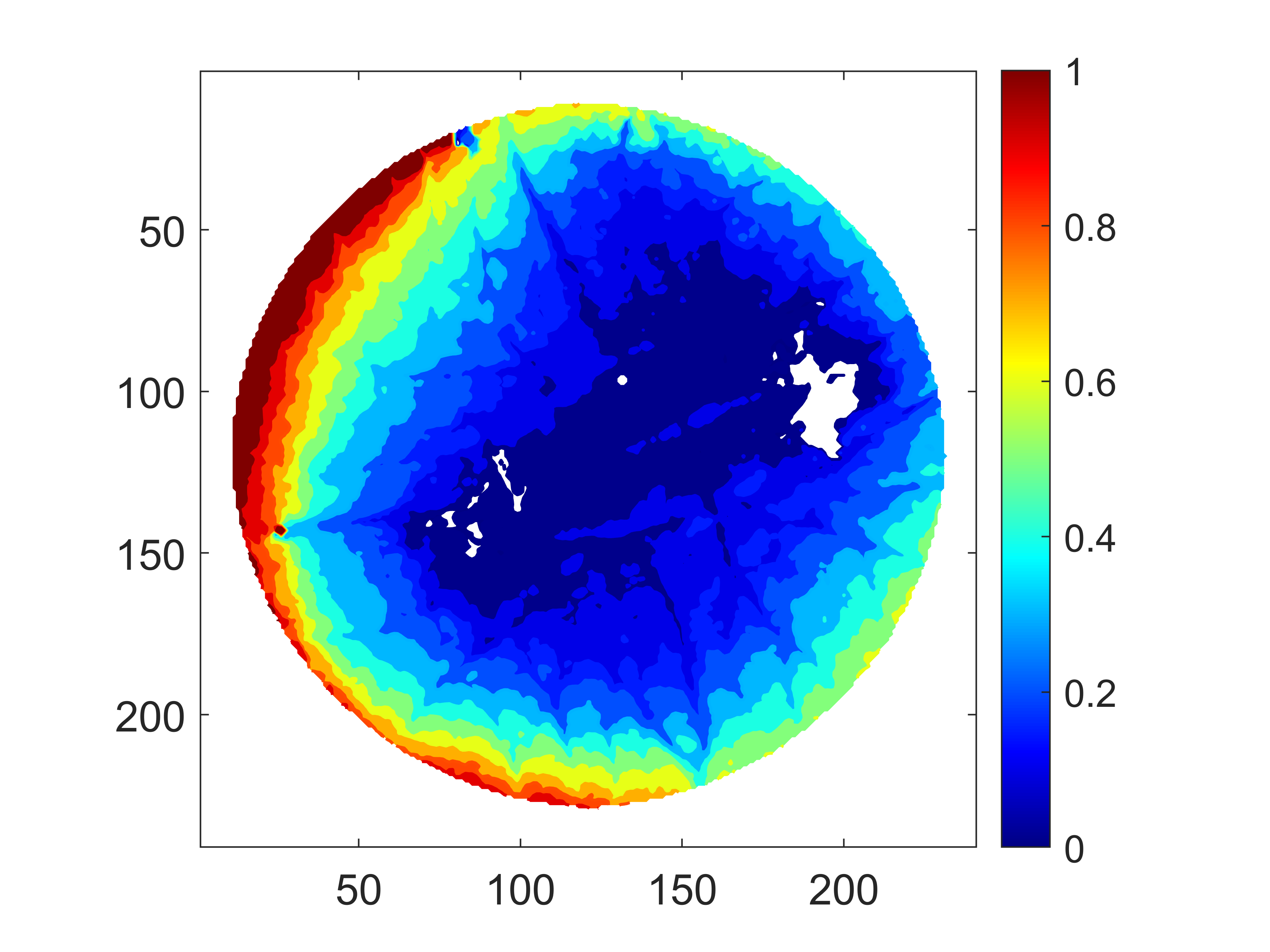

Supplement: S1 File — (ZIP) [file pone.0308204.s001.zip › S1 file. Birefringence Images/B-PK/60 degree/2693OS/contousfilled.tif]

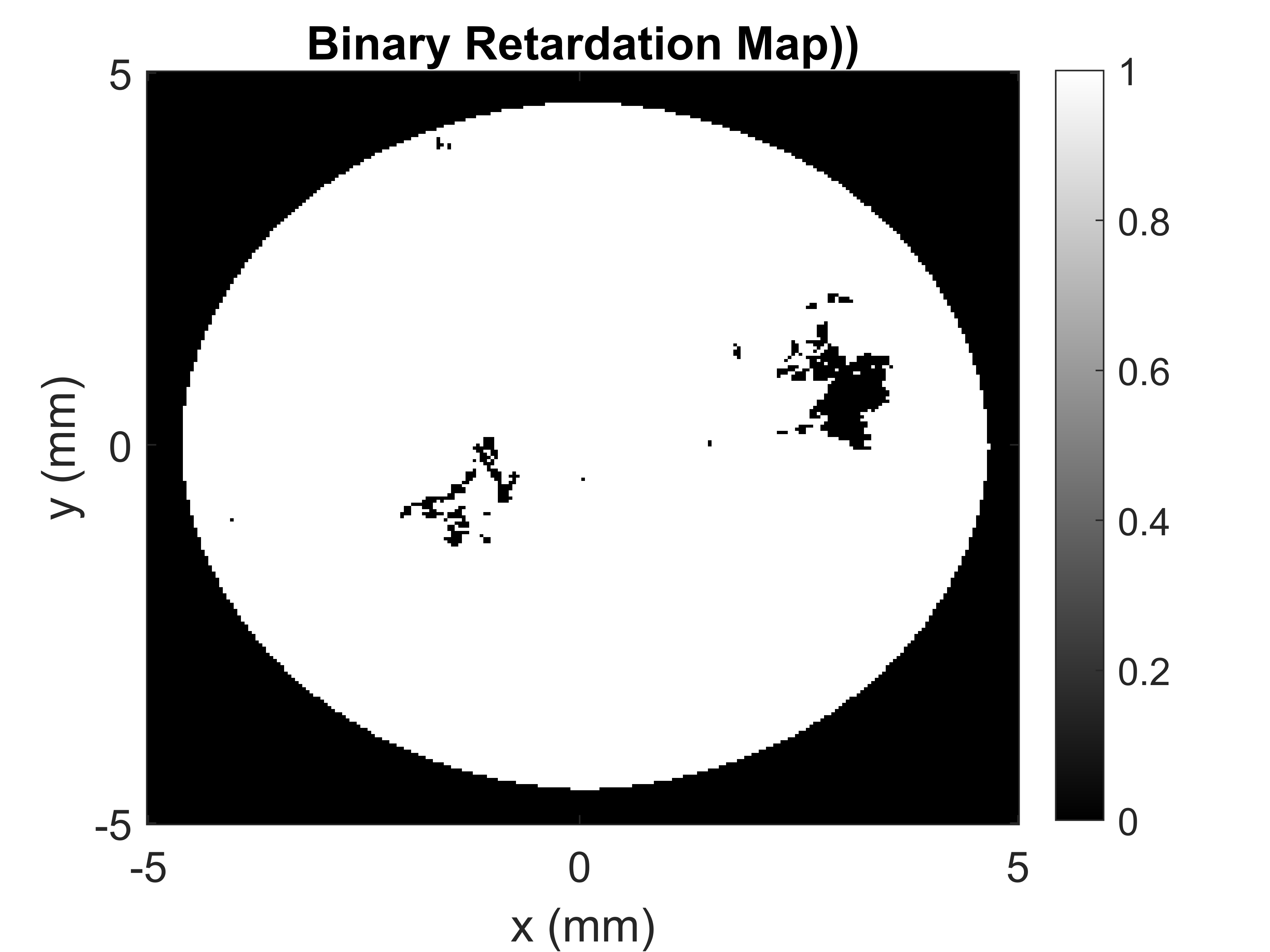

Supplement: S1 File — (ZIP) [file pone.0308204.s001.zip › S1 file. Birefringence Images/B-PK/60 degree/2693OS/isoropic.tif]

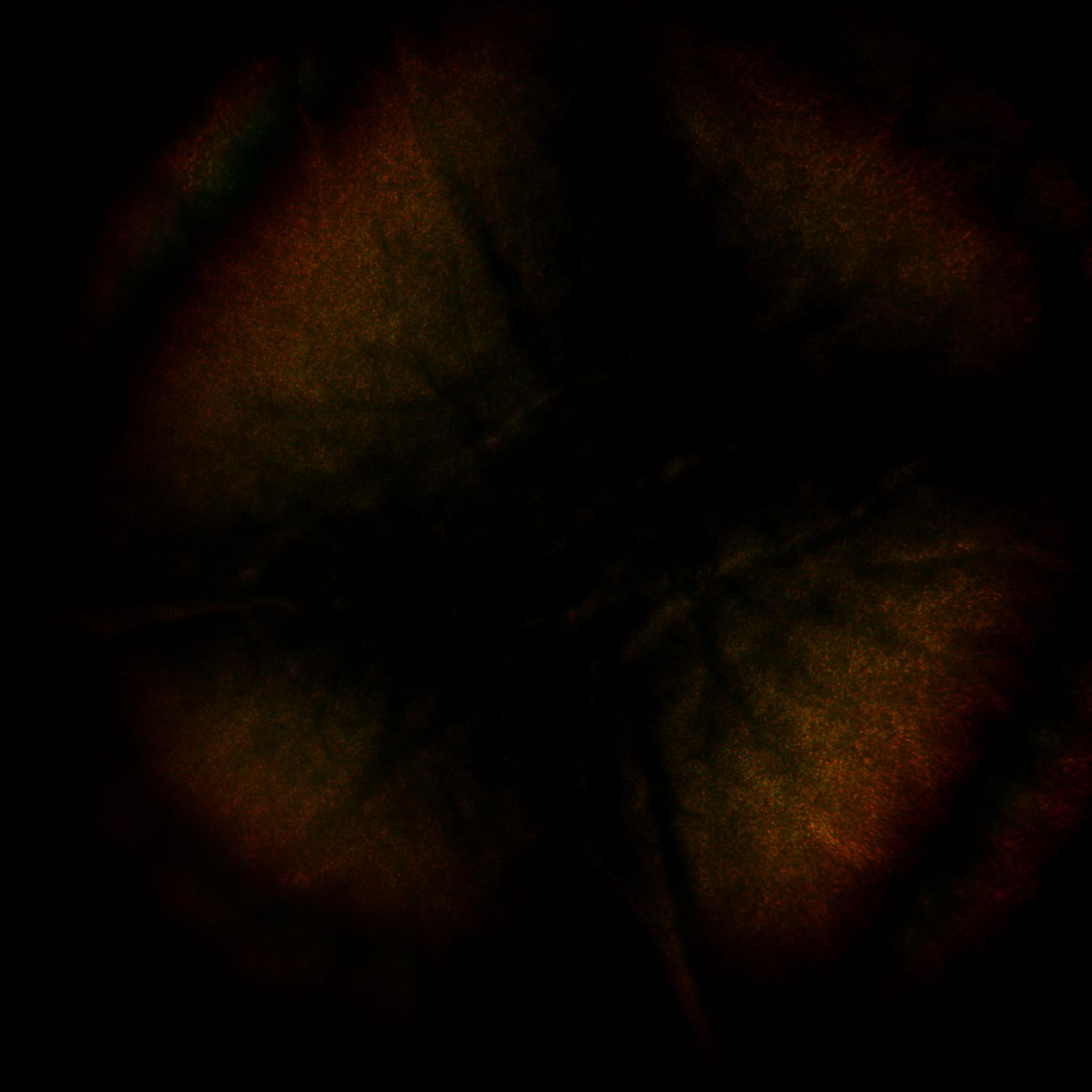

Supplement: S1 File — (ZIP) [file pone.0308204.s001.zip › S1 file. Birefringence Images/B-PK/60 degree/2693OS/IW1.jpg]

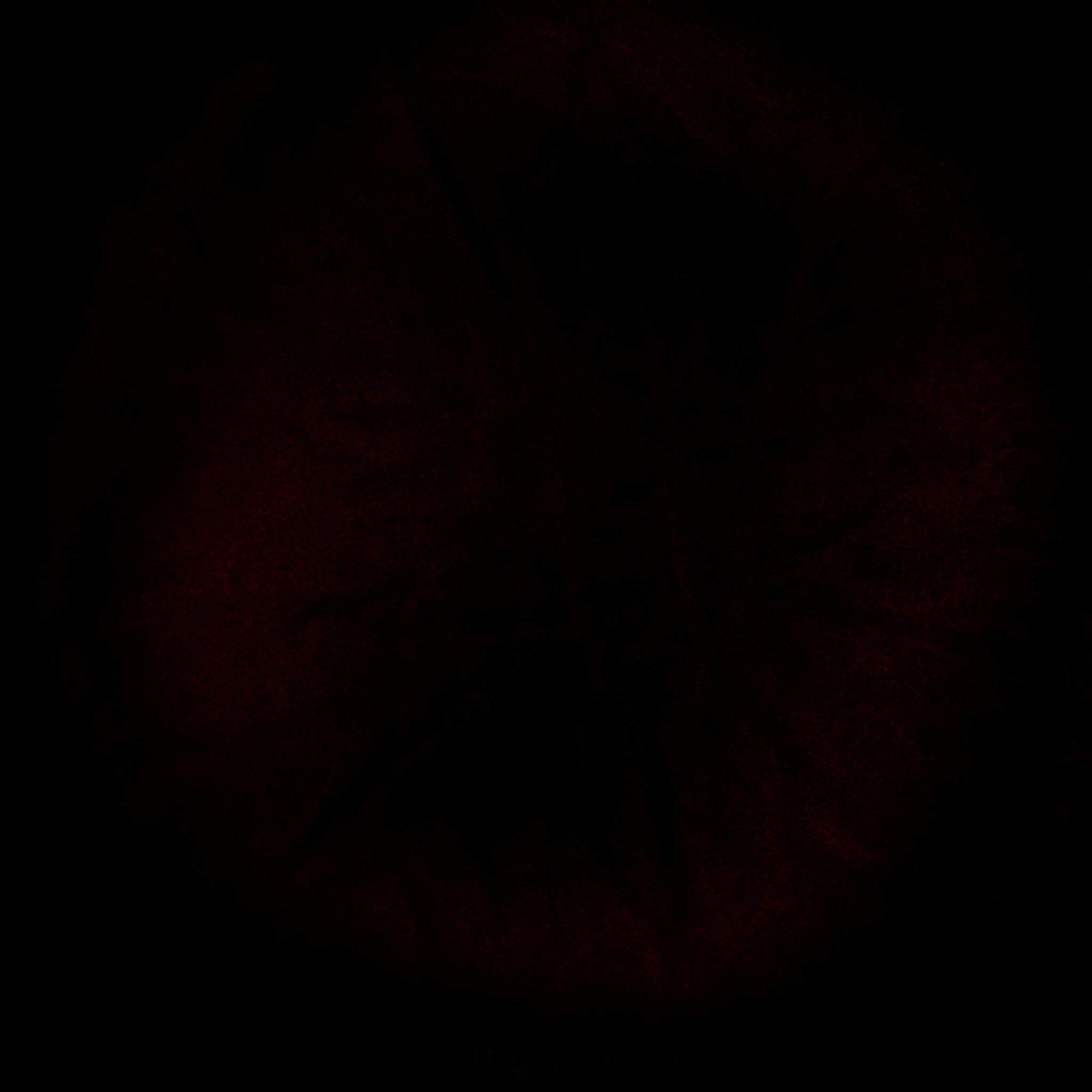

Supplement: S1 File — (ZIP) [file pone.0308204.s001.zip › S1 file. Birefringence Images/B-PK/60 degree/2693OS/IW10.jpg]

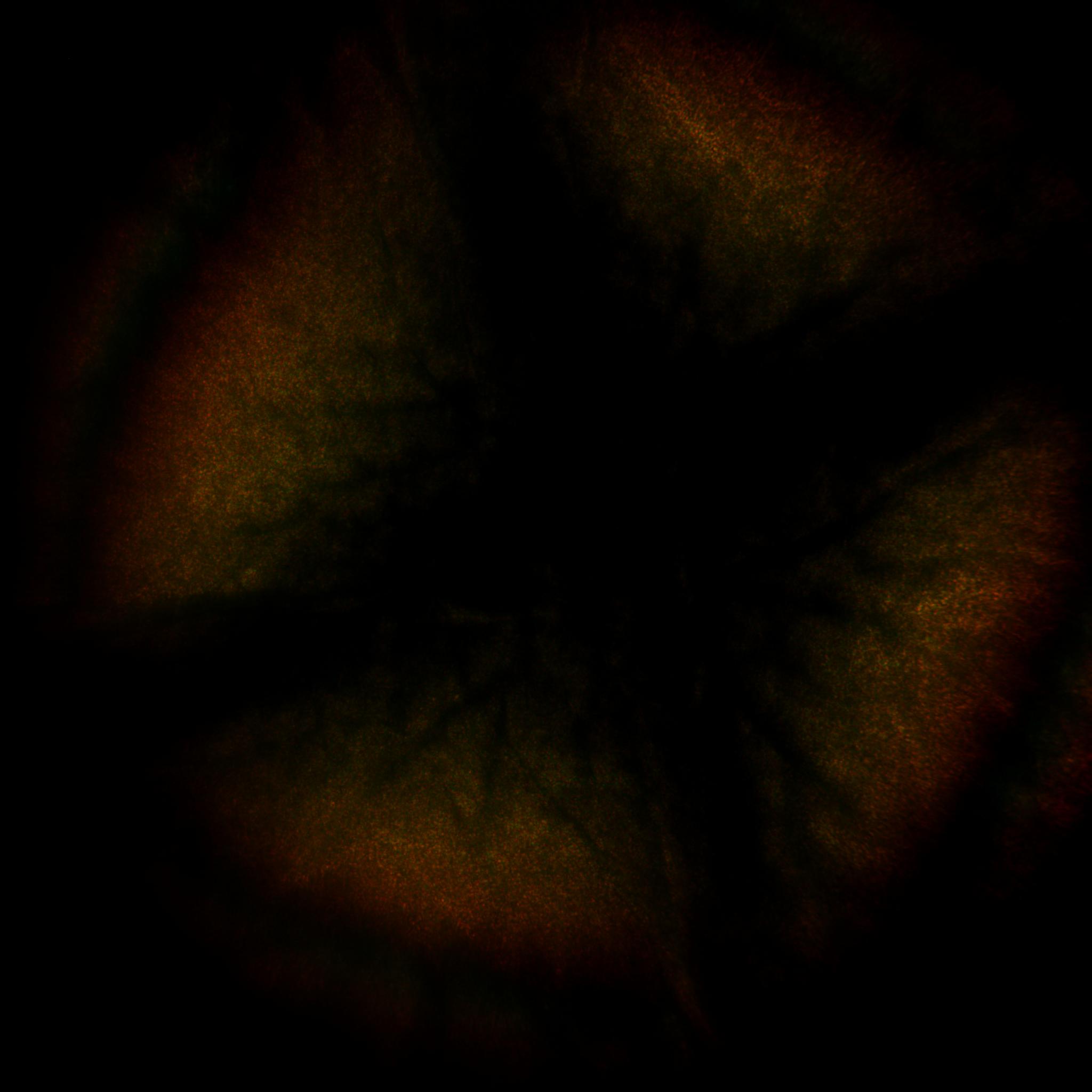

Supplement: S1 File — (ZIP) [file pone.0308204.s001.zip › S1 file. Birefringence Images/B-PK/60 degree/2693OS/IW2.jpg]

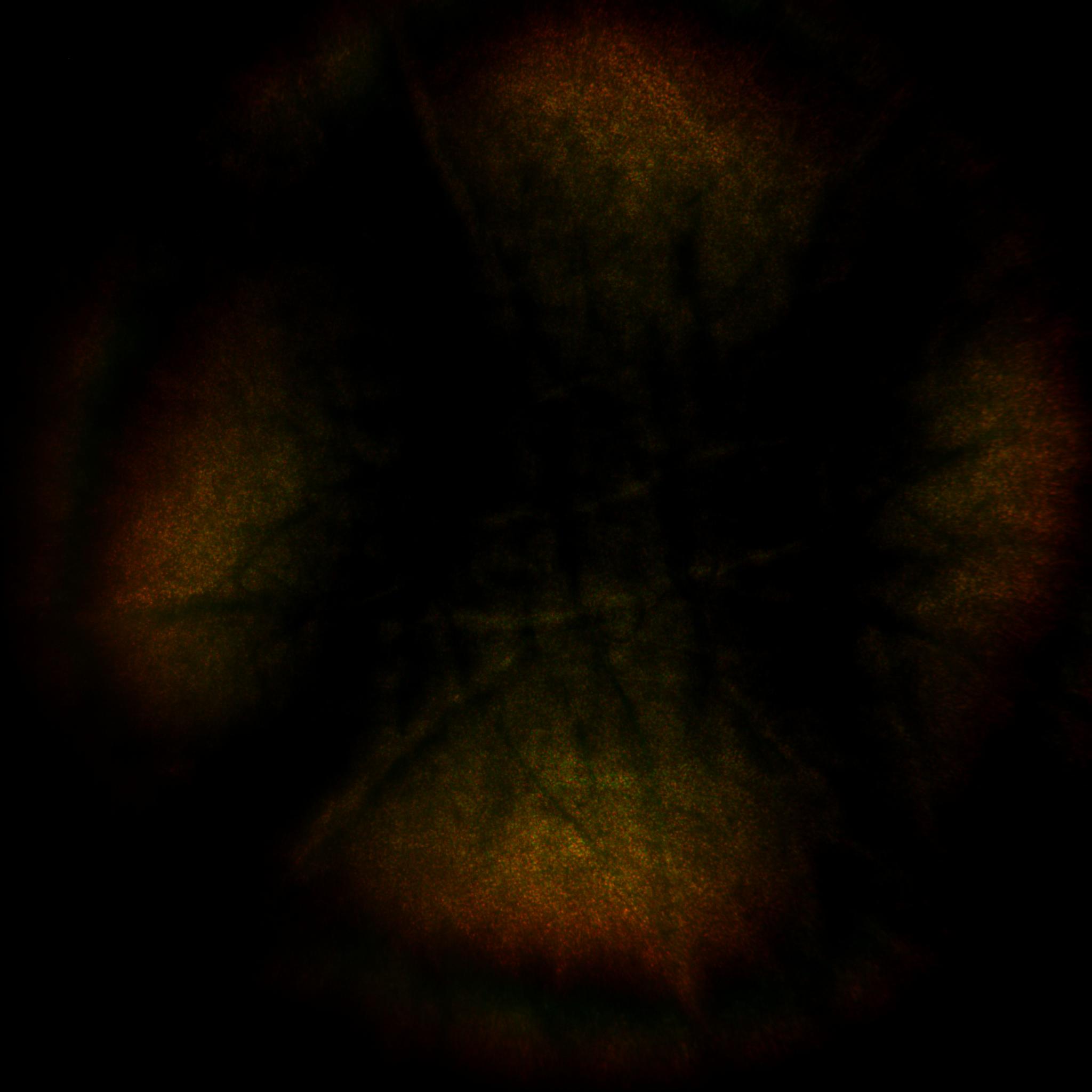

Supplement: S1 File — (ZIP) [file pone.0308204.s001.zip › S1 file. Birefringence Images/B-PK/60 degree/2693OS/IW3.jpg]

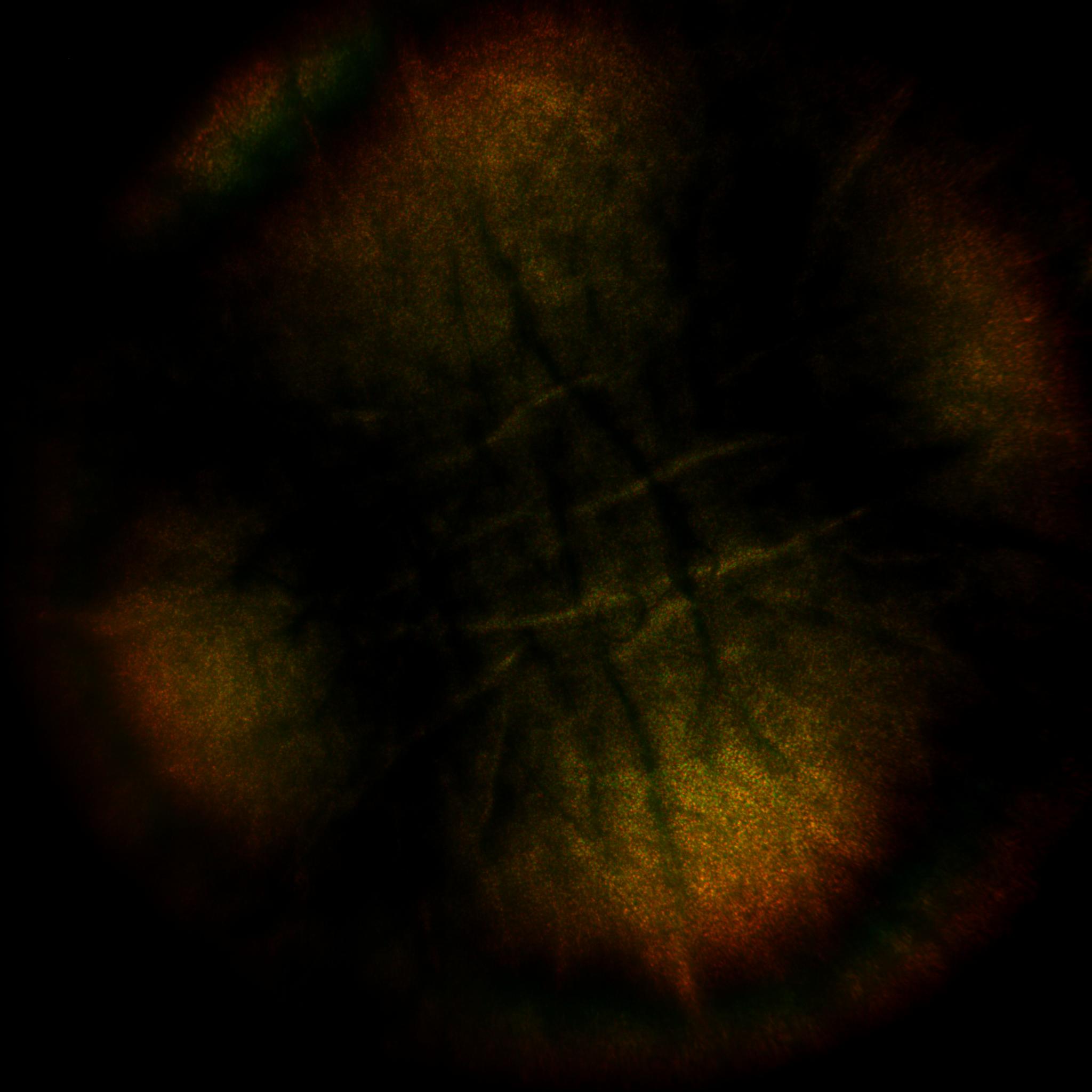

Supplement: S1 File — (ZIP) [file pone.0308204.s001.zip › S1 file. Birefringence Images/B-PK/60 degree/2693OS/IW4.jpg]

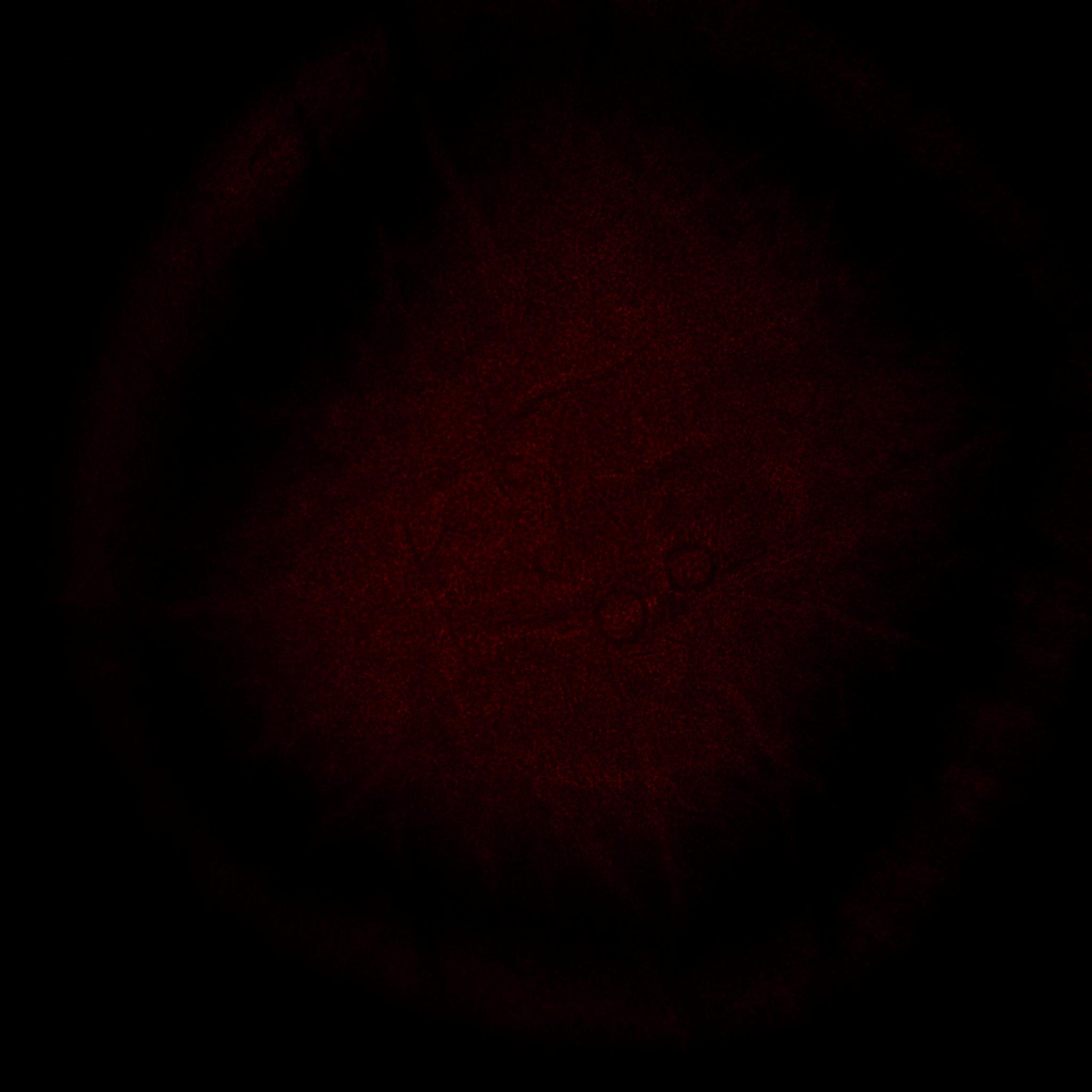

Supplement: S1 File — (ZIP) [file pone.0308204.s001.zip › S1 file. Birefringence Images/B-PK/60 degree/2693OS/IW5.jpg]

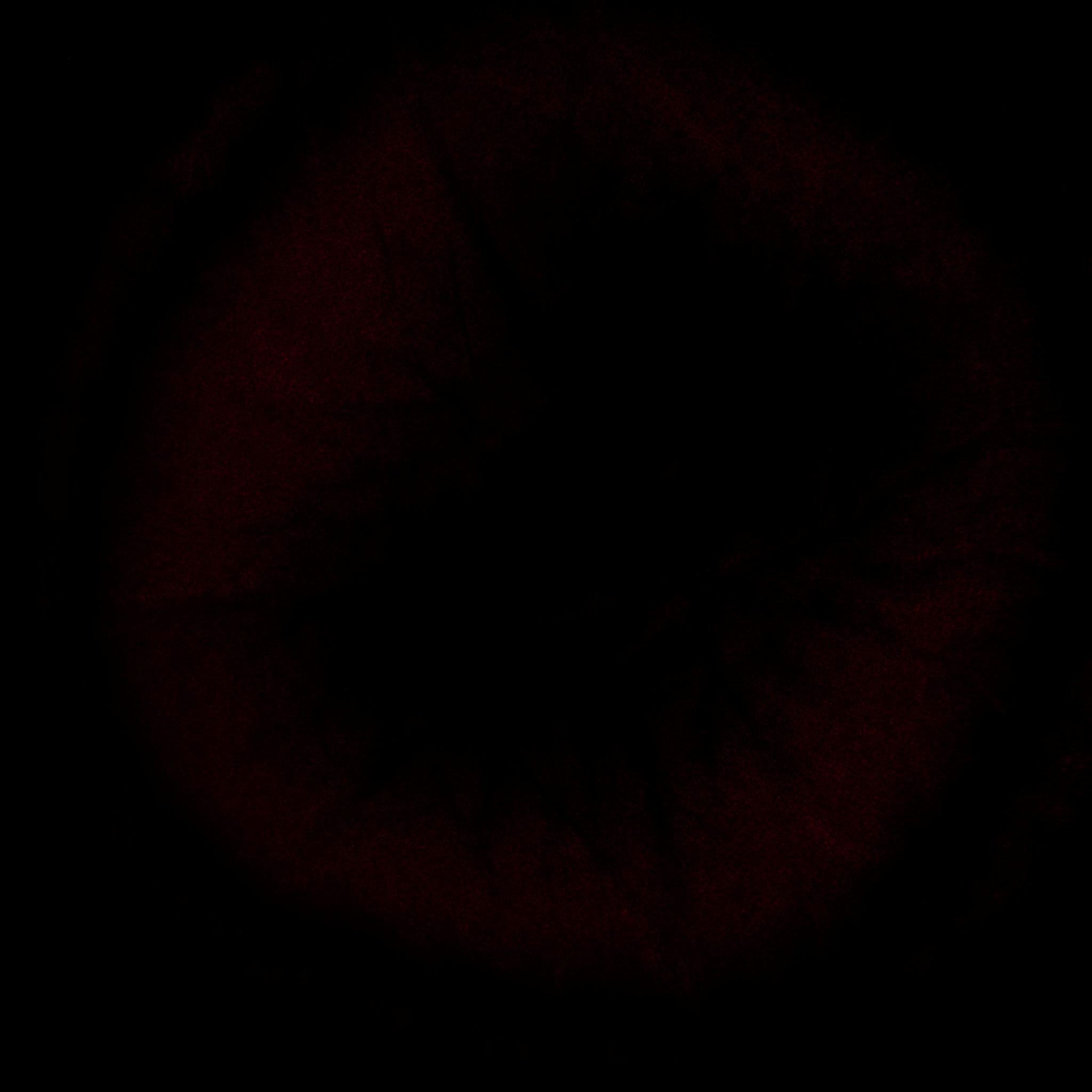

Supplement: S1 File — (ZIP) [file pone.0308204.s001.zip › S1 file. Birefringence Images/B-PK/60 degree/2693OS/IW6.jpg]

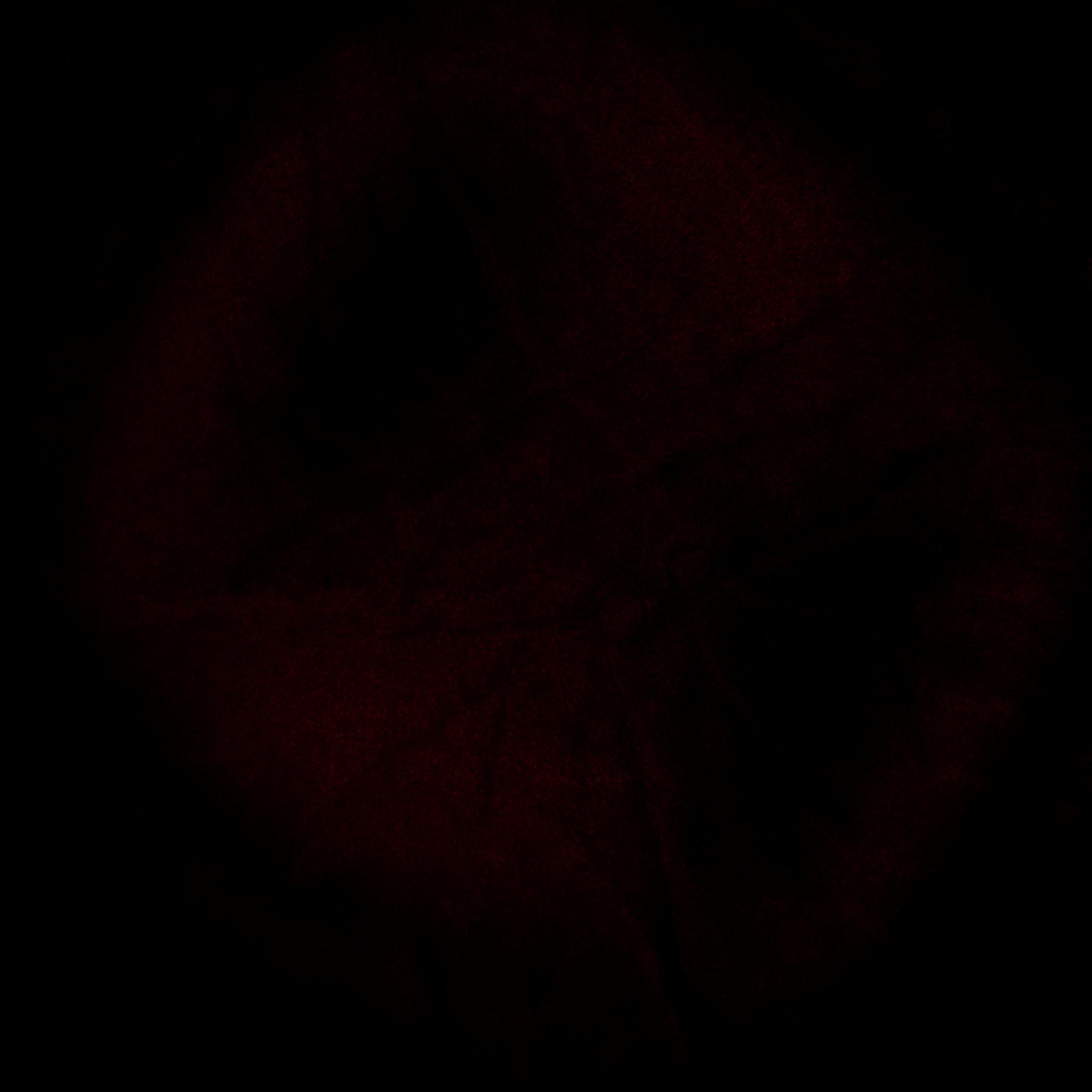

Supplement: S1 File — (ZIP) [file pone.0308204.s001.zip › S1 file. Birefringence Images/B-PK/60 degree/2693OS/IW7.jpg]

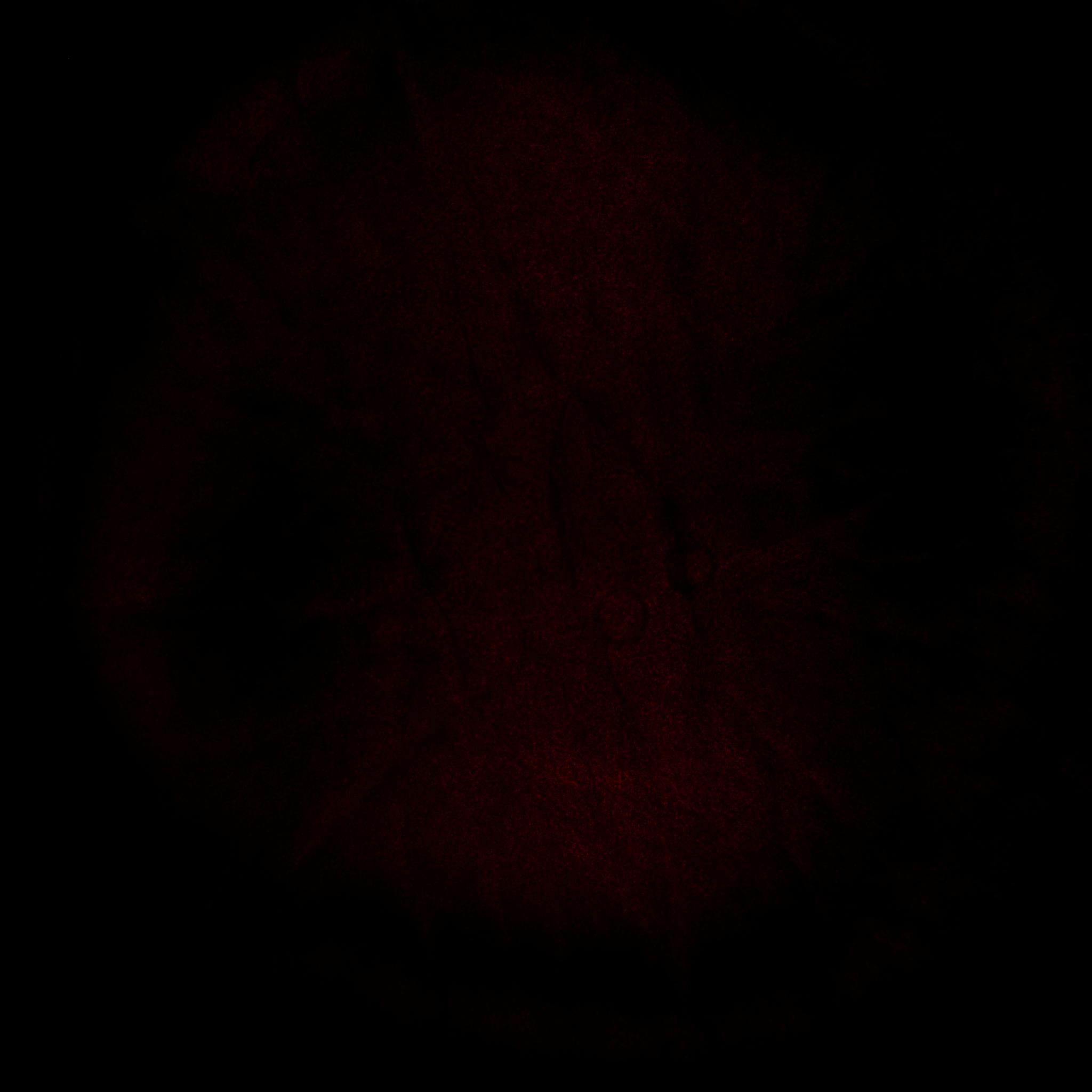

Supplement: S1 File — (ZIP) [file pone.0308204.s001.zip › S1 file. Birefringence Images/B-PK/60 degree/2693OS/IW8.jpg]

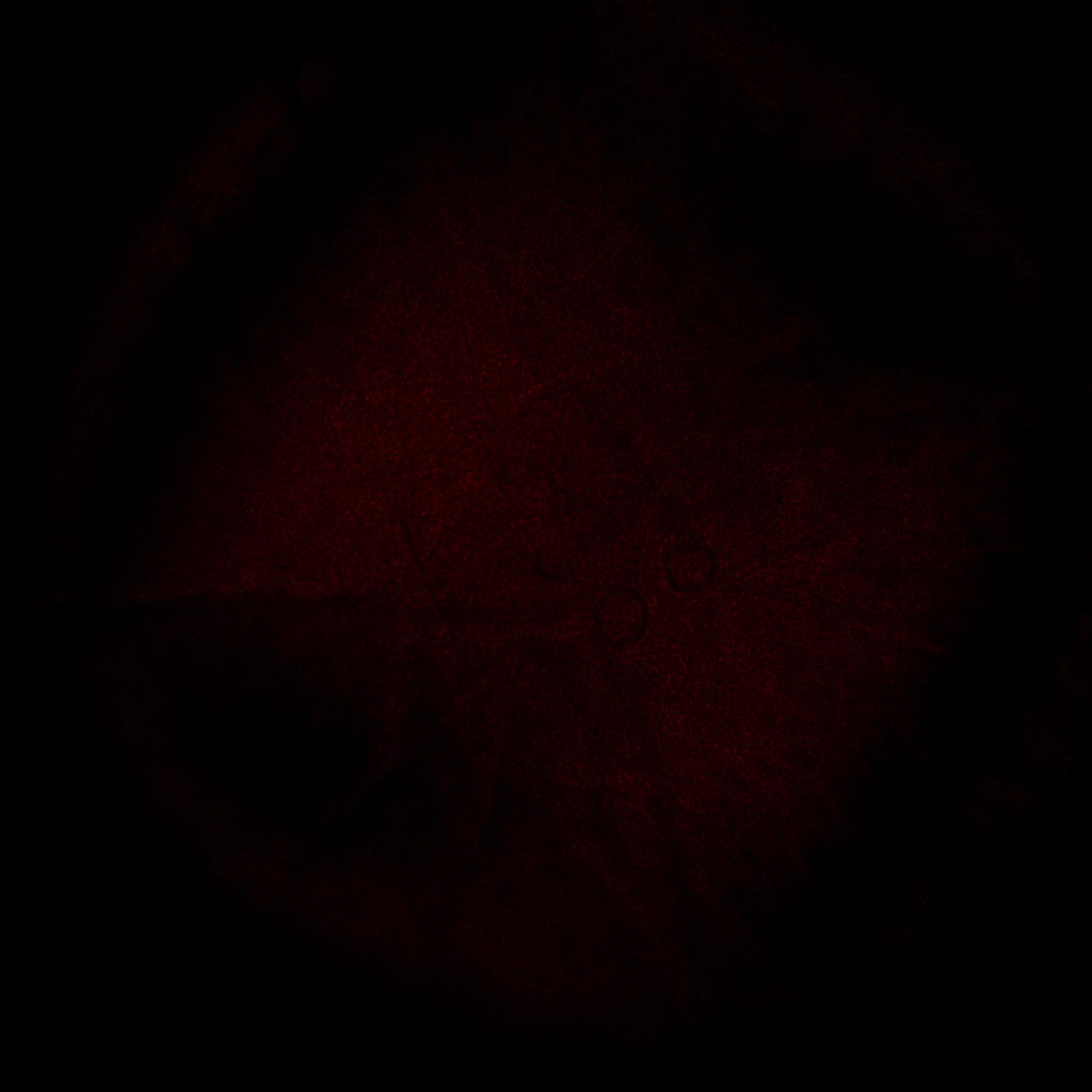

Supplement: S1 File — (ZIP) [file pone.0308204.s001.zip › S1 file. Birefringence Images/B-PK/60 degree/2693OS/IW9.jpg]

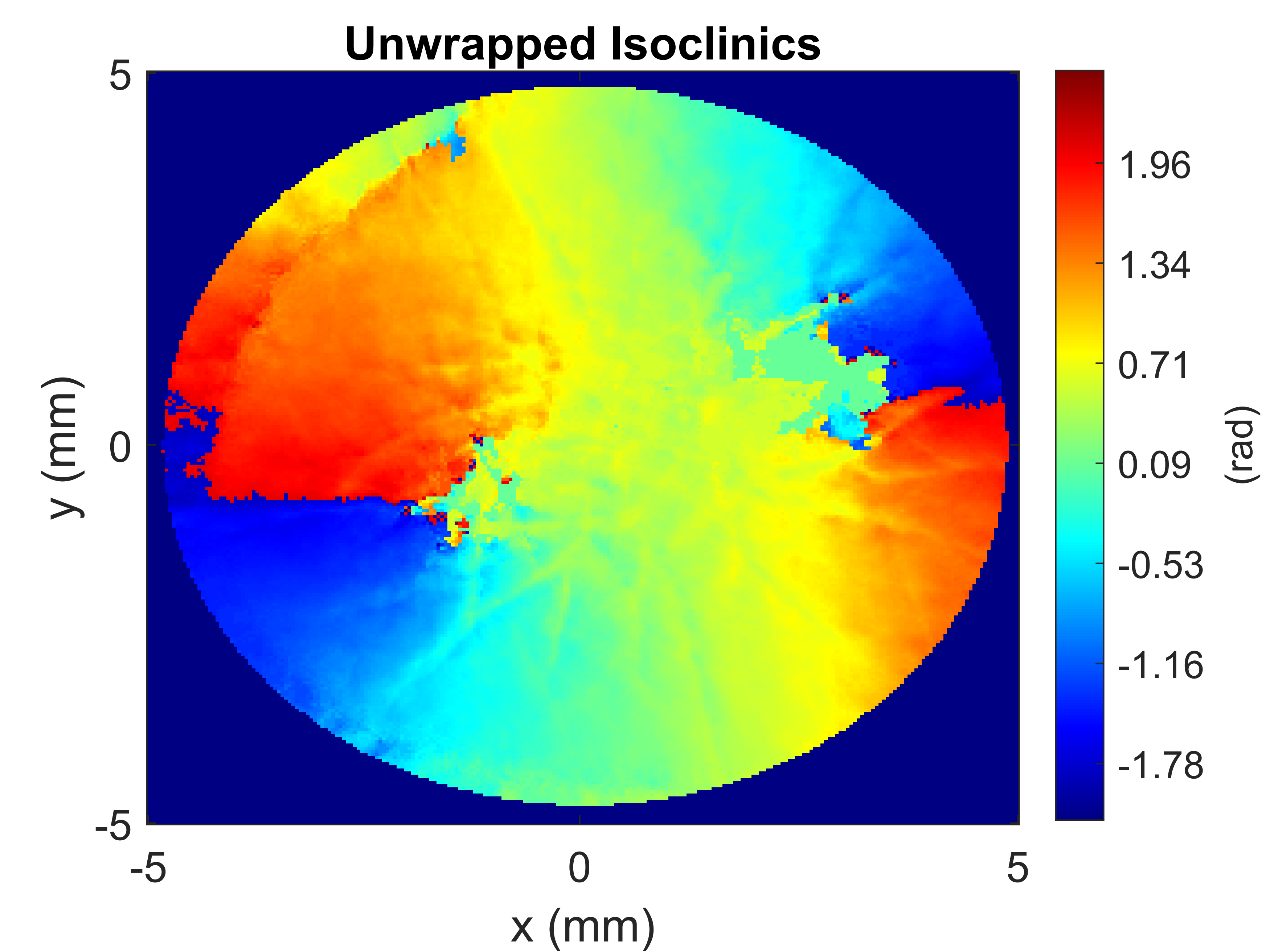

Supplement: S1 File — (ZIP) [file pone.0308204.s001.zip › S1 file. Birefringence Images/B-PK/60 degree/2693OS/unwappedISO.tif]

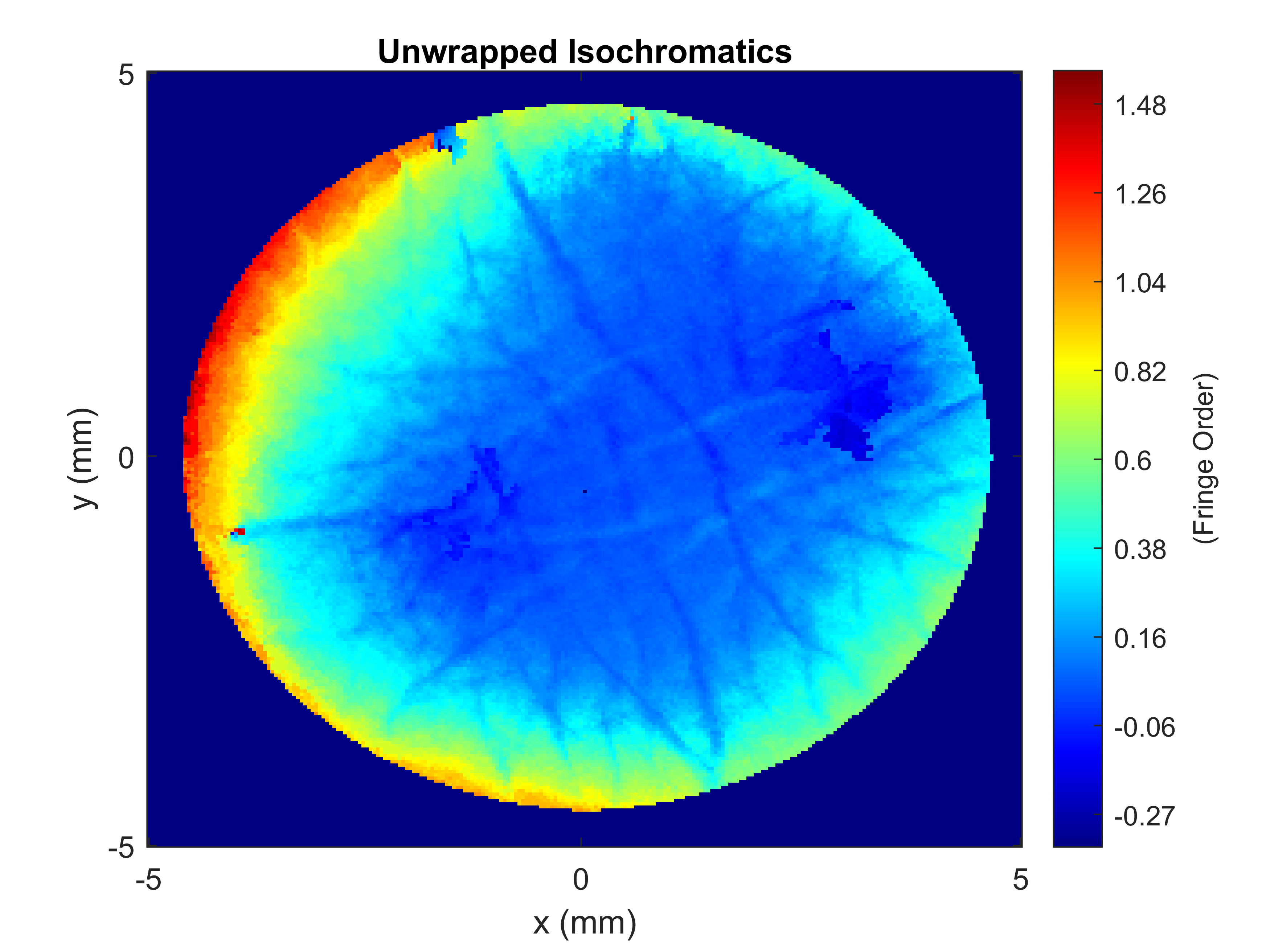

Supplement: S1 File — (ZIP) [file pone.0308204.s001.zip › S1 file. Birefringence Images/B-PK/60 degree/2693OS/unwappedISOCHcolo.tif]

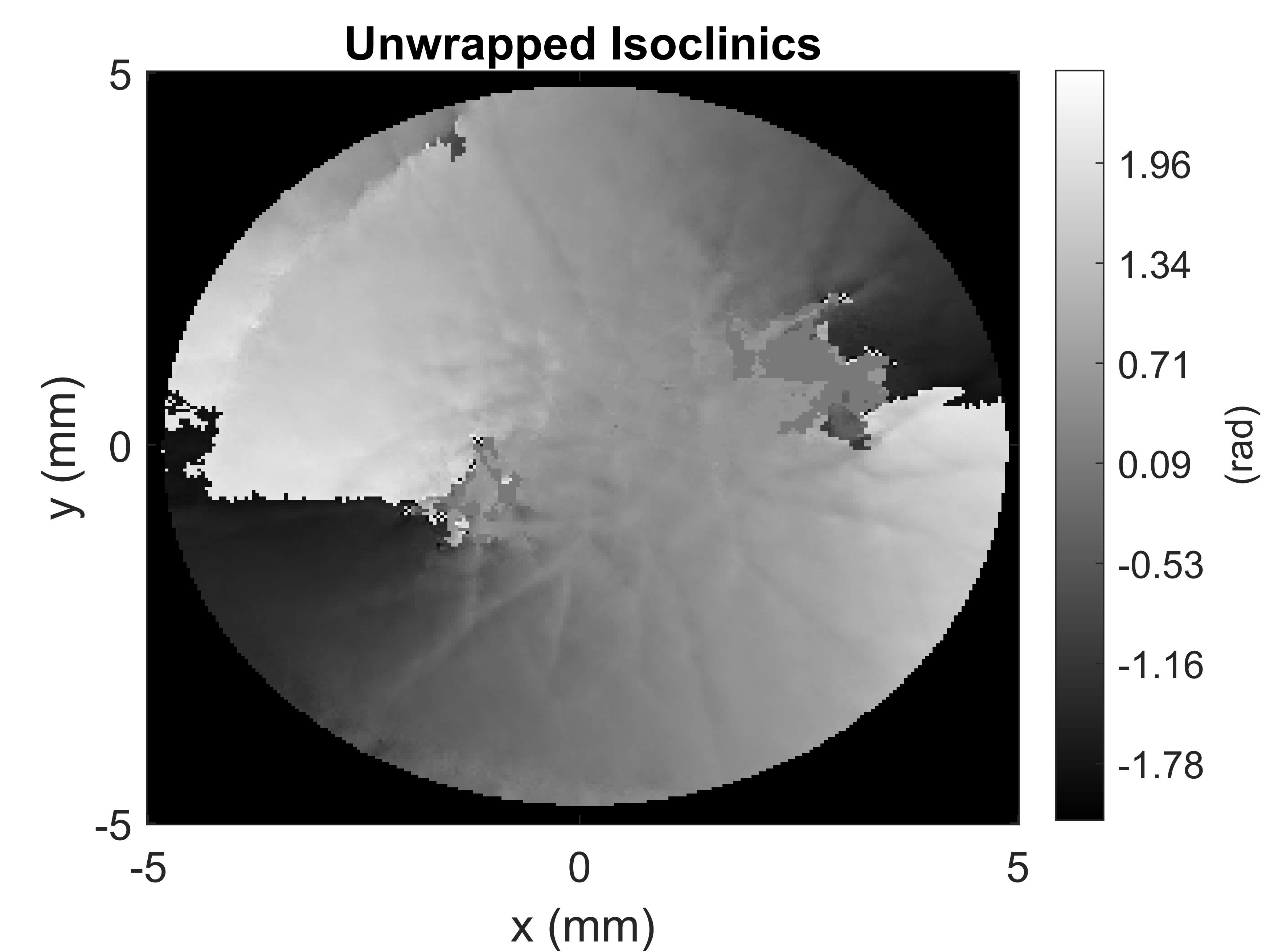

Supplement: S1 File — (ZIP) [file pone.0308204.s001.zip › S1 file. Birefringence Images/B-PK/60 degree/2693OS/wrappedISOcolo.tif]

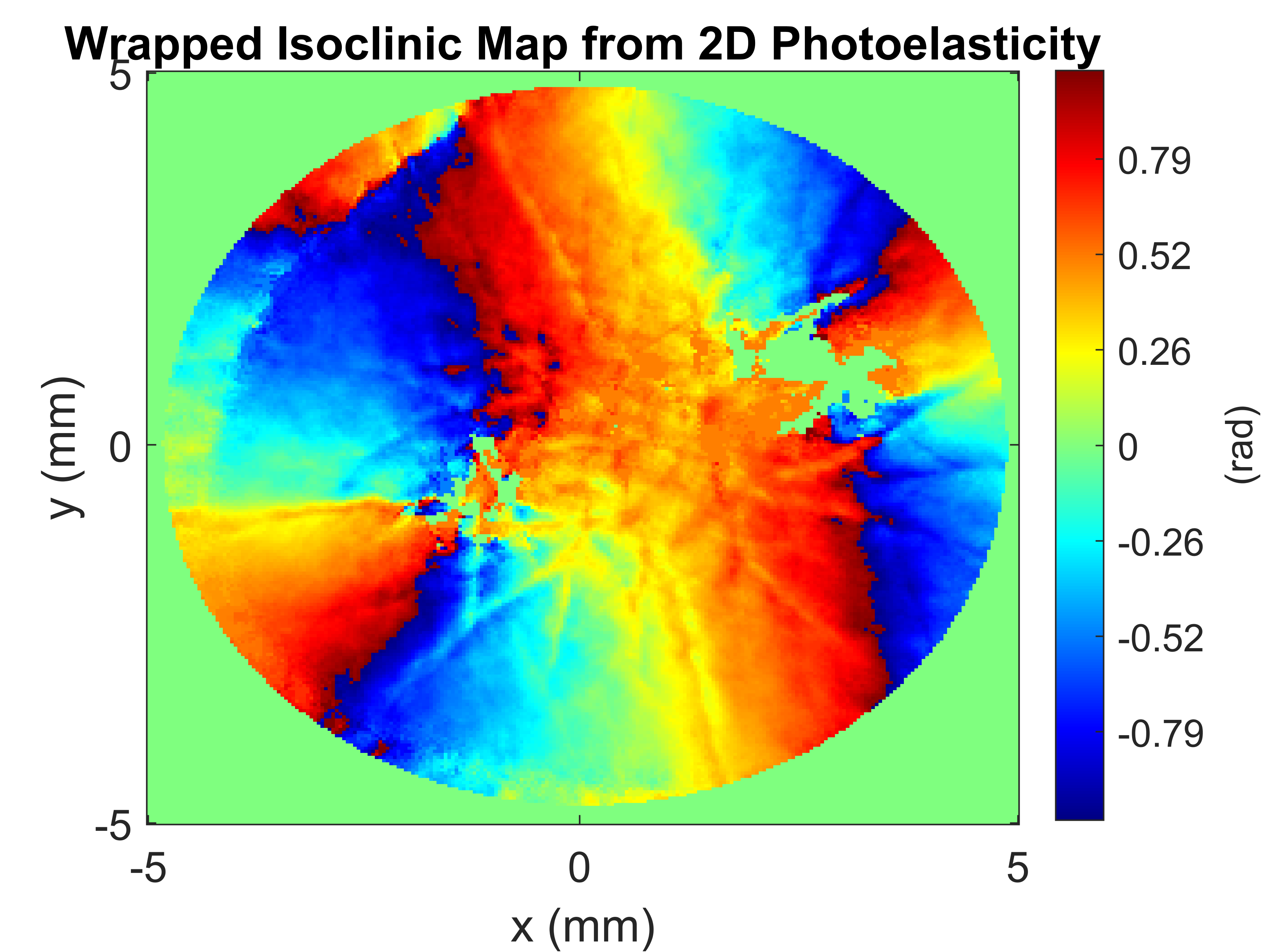

Supplement: S1 File — (ZIP) [file pone.0308204.s001.zip › S1 file. Birefringence Images/B-PK/60 degree/2693OS/wrappedISOcolor.tif]

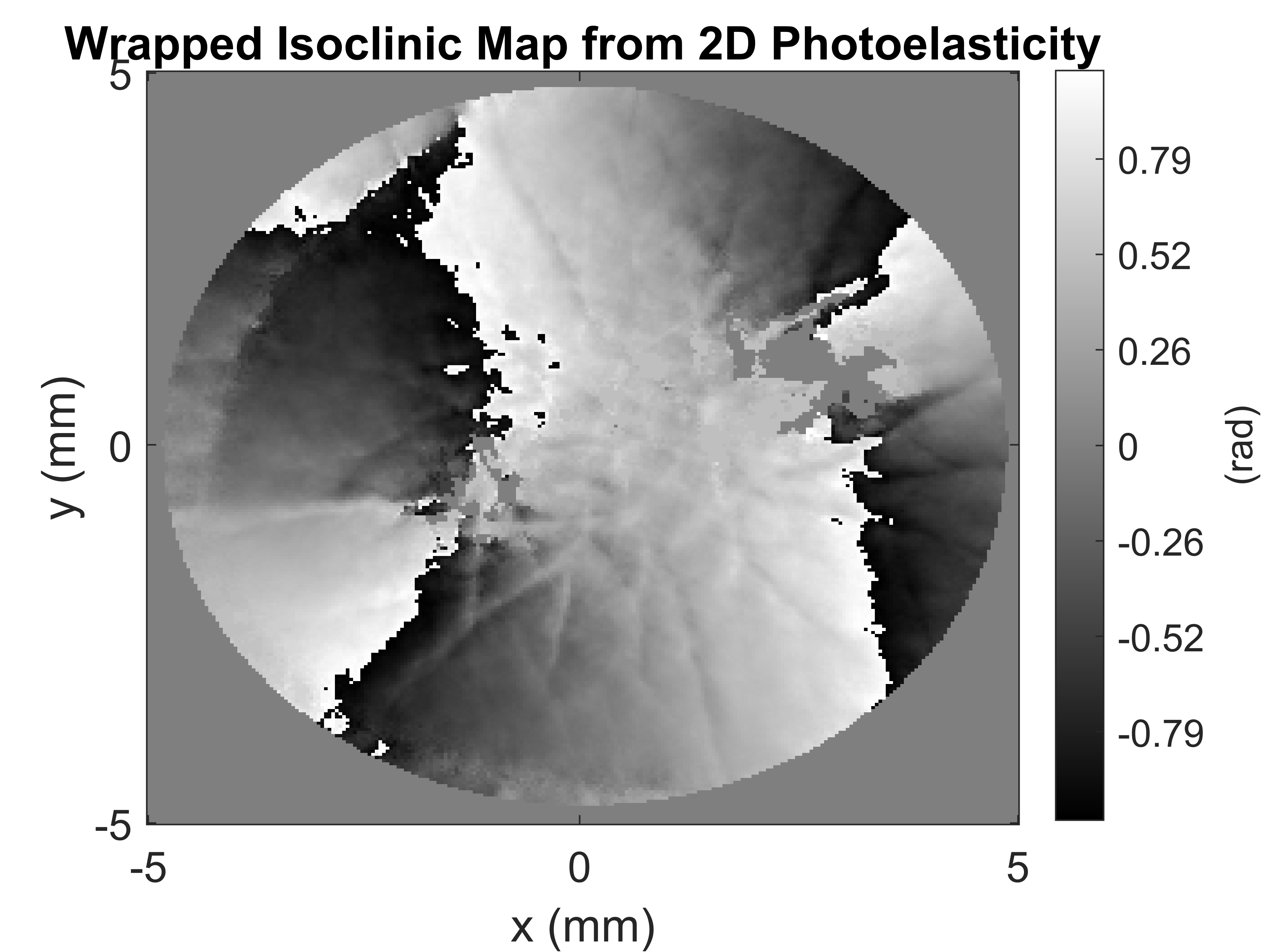

Supplement: S1 File — (ZIP) [file pone.0308204.s001.zip › S1 file. Birefringence Images/B-PK/60 degree/2693OS/wrappedISOgray.tif]

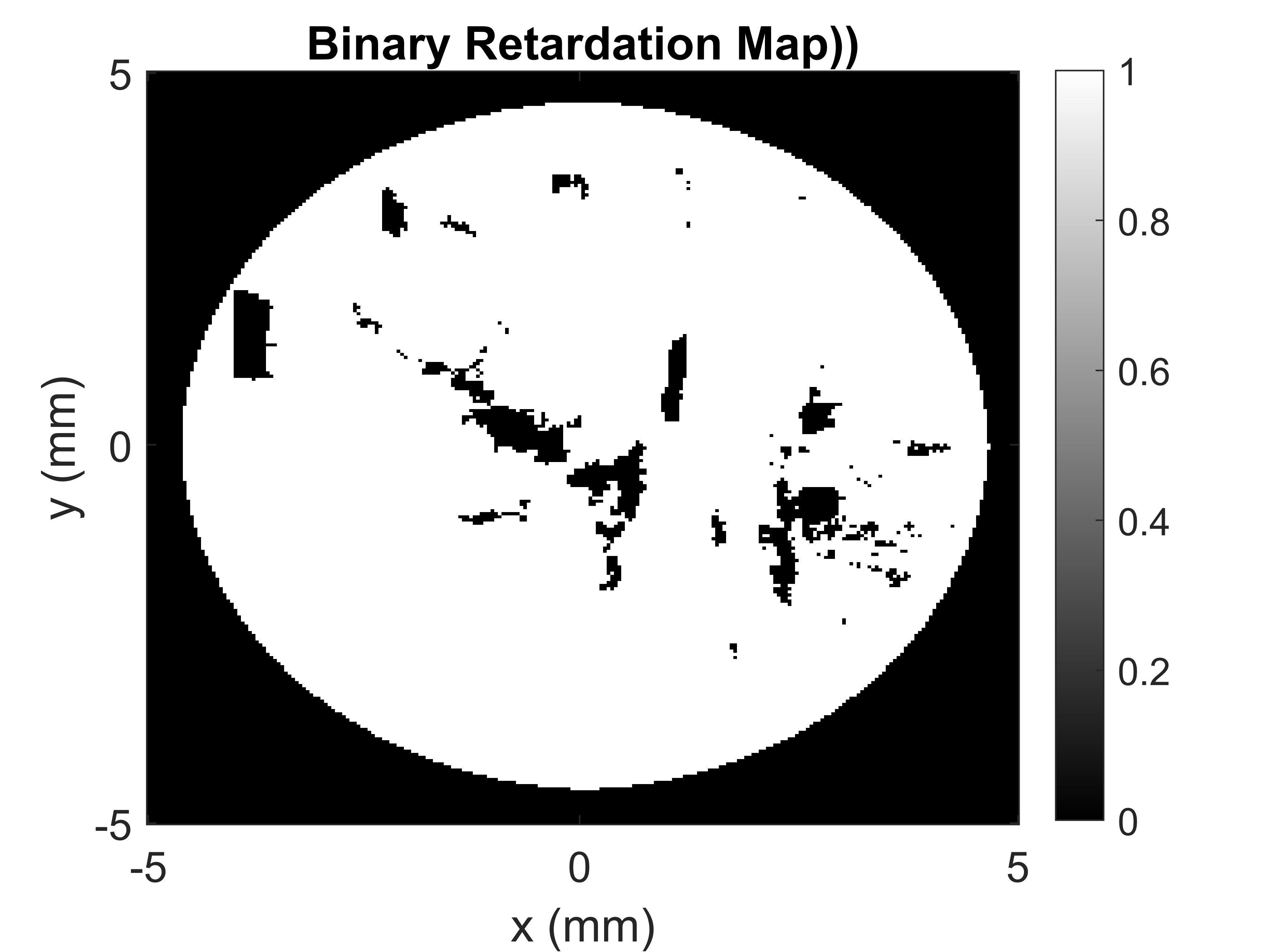

Supplement: S1 File — (ZIP) [file pone.0308204.s001.zip › S1 file. Birefringence Images/B-PK/90 degee/2845OD/isoriopic.tif]

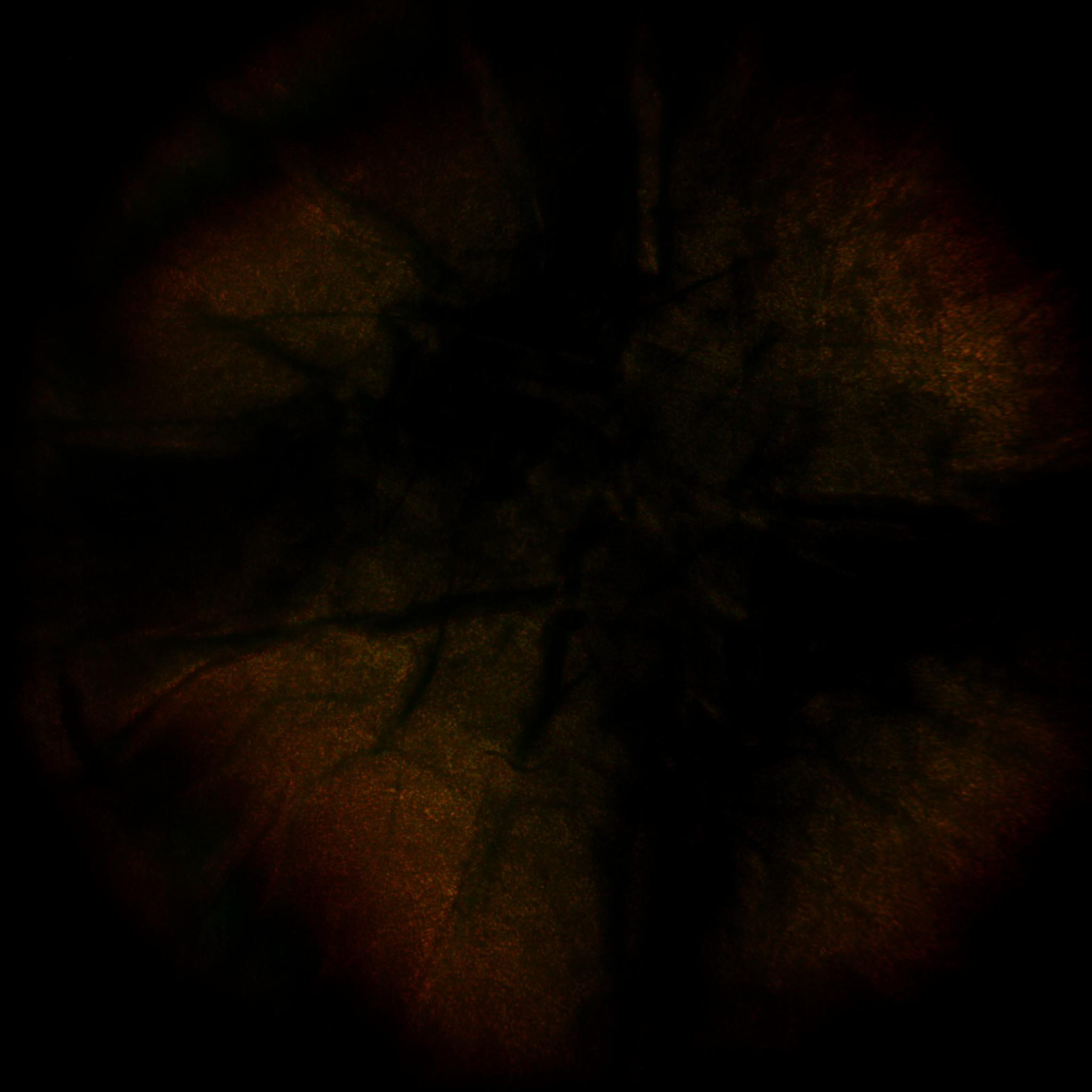

Supplement: S1 File — (ZIP) [file pone.0308204.s001.zip › S1 file. Birefringence Images/B-PK/90 degee/2845OD/IW1.jpg]

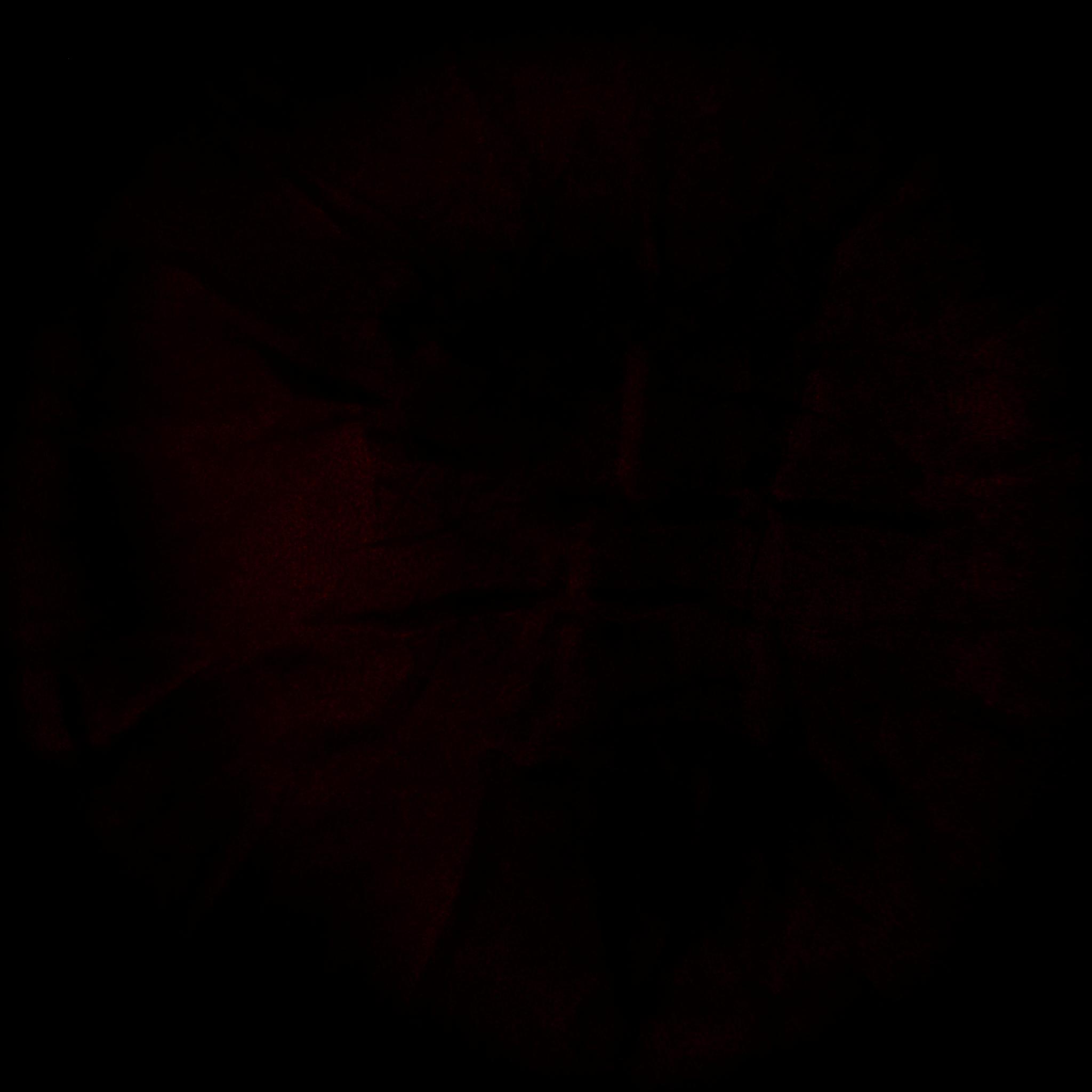

Supplement: S1 File — (ZIP) [file pone.0308204.s001.zip › S1 file. Birefringence Images/B-PK/90 degee/2845OD/IW10.jpg]

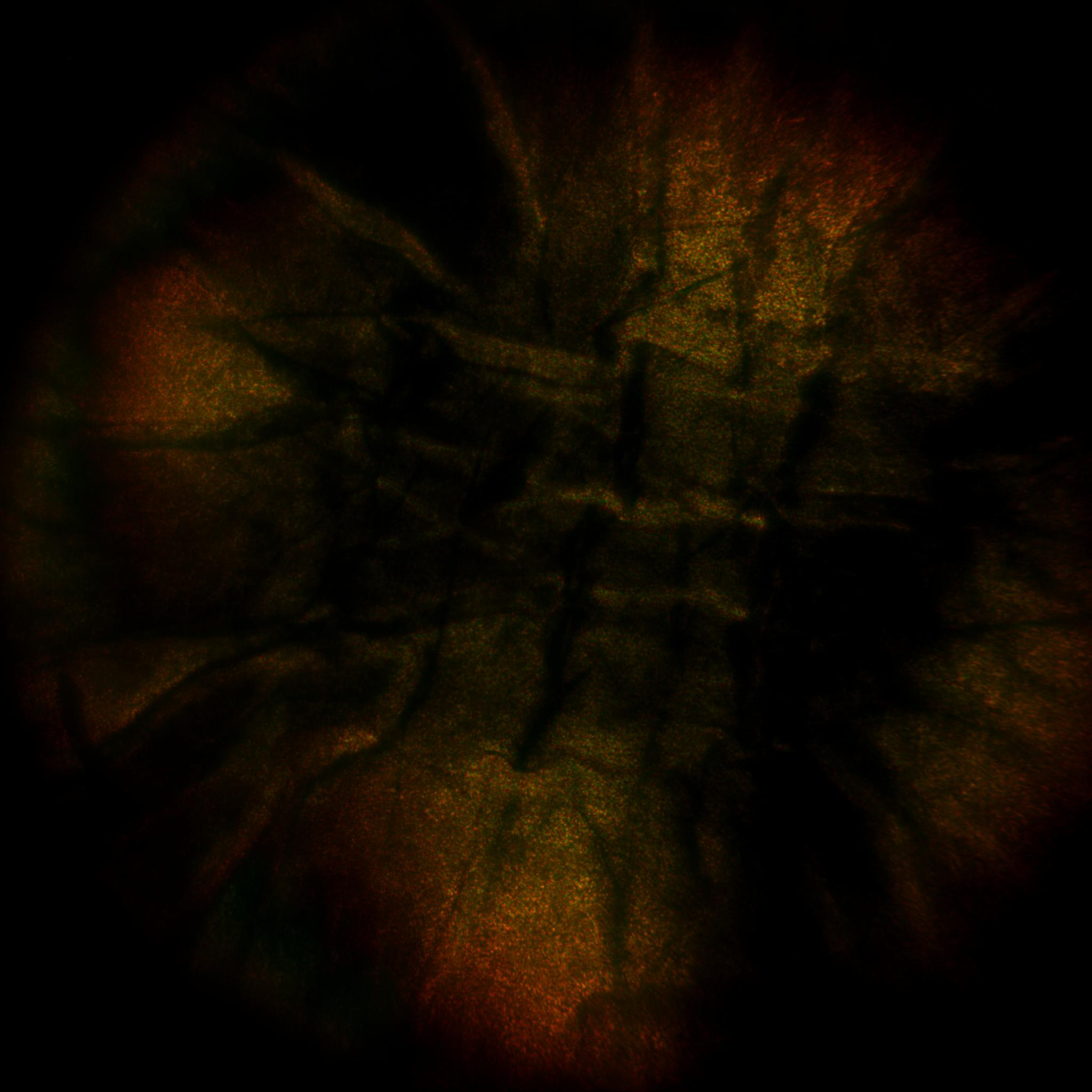

Supplement: S1 File — (ZIP) [file pone.0308204.s001.zip › S1 file. Birefringence Images/B-PK/90 degee/2845OD/IW2.jpg]

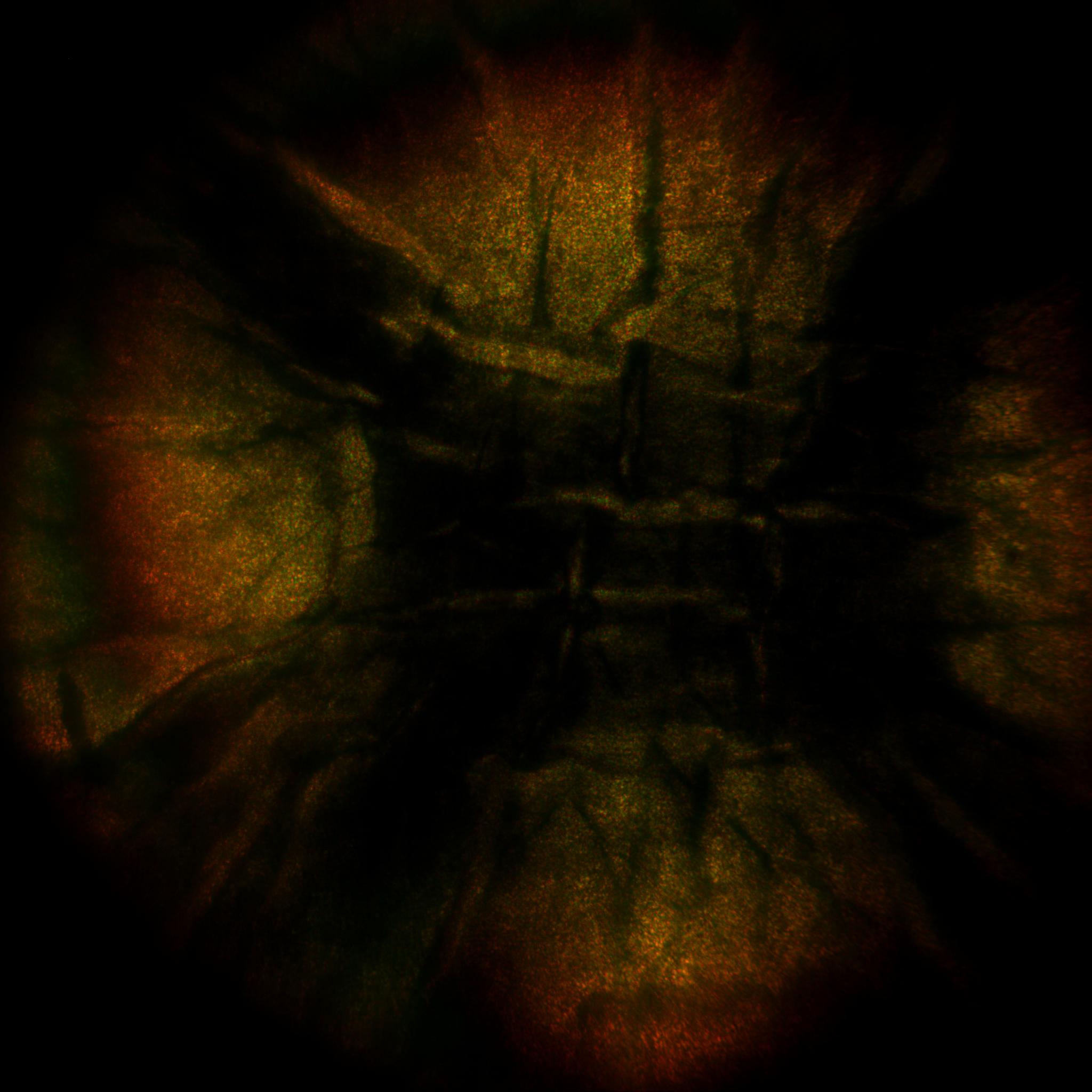

Supplement: S1 File — (ZIP) [file pone.0308204.s001.zip › S1 file. Birefringence Images/B-PK/90 degee/2845OD/IW3.jpg]

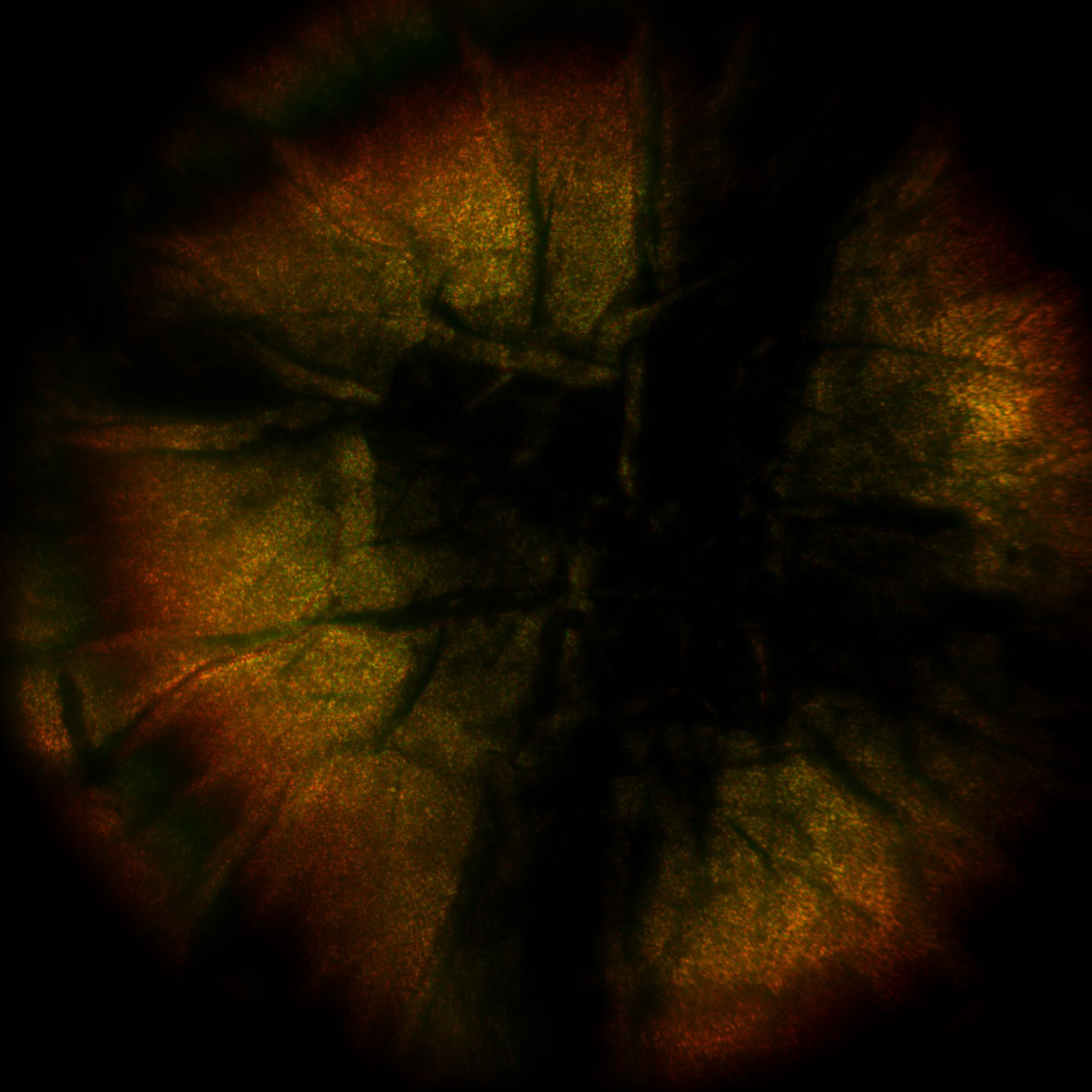

Supplement: S1 File — (ZIP) [file pone.0308204.s001.zip › S1 file. Birefringence Images/B-PK/90 degee/2845OD/IW4.jpg]

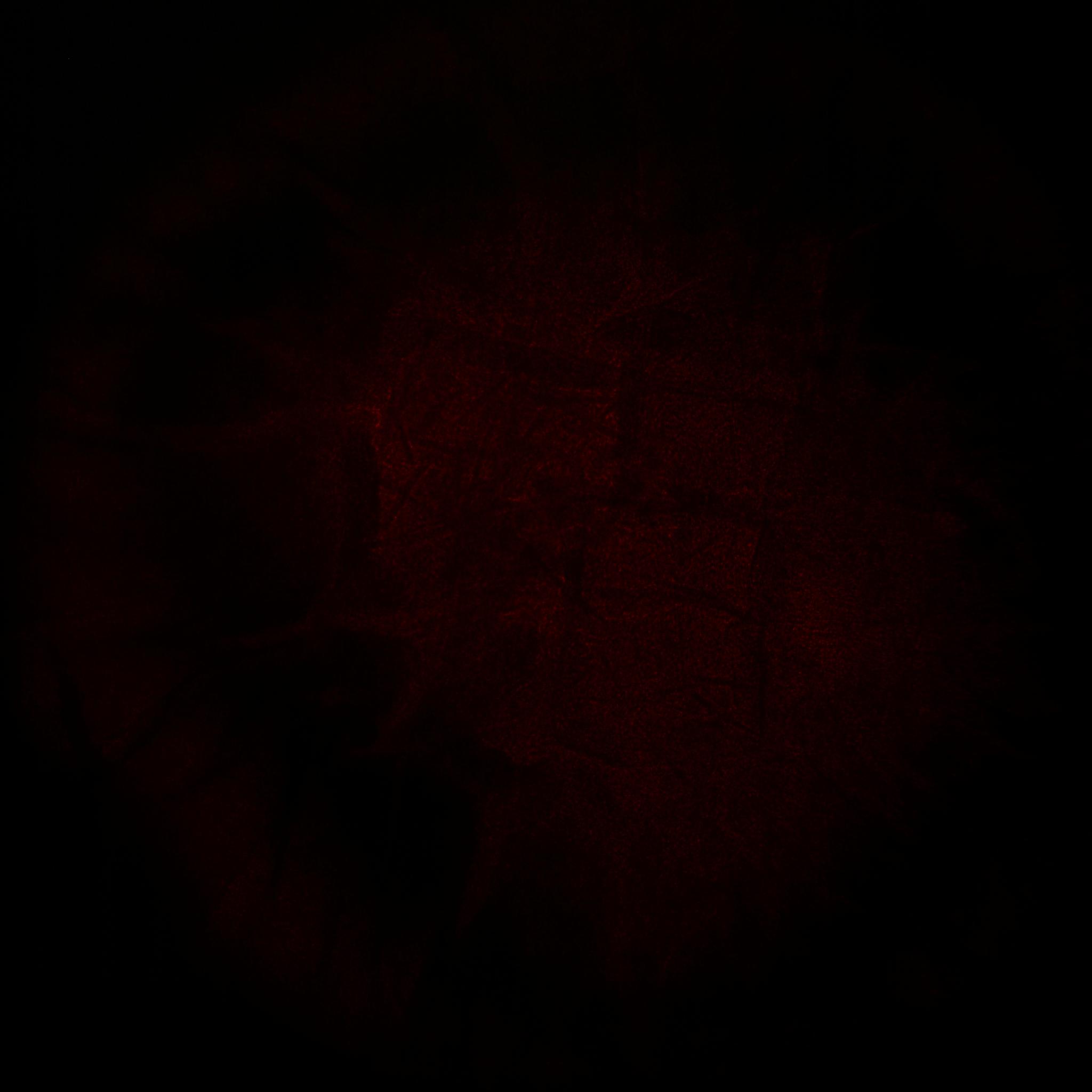

Supplement: S1 File — (ZIP) [file pone.0308204.s001.zip › S1 file. Birefringence Images/B-PK/90 degee/2845OD/IW5.jpg]

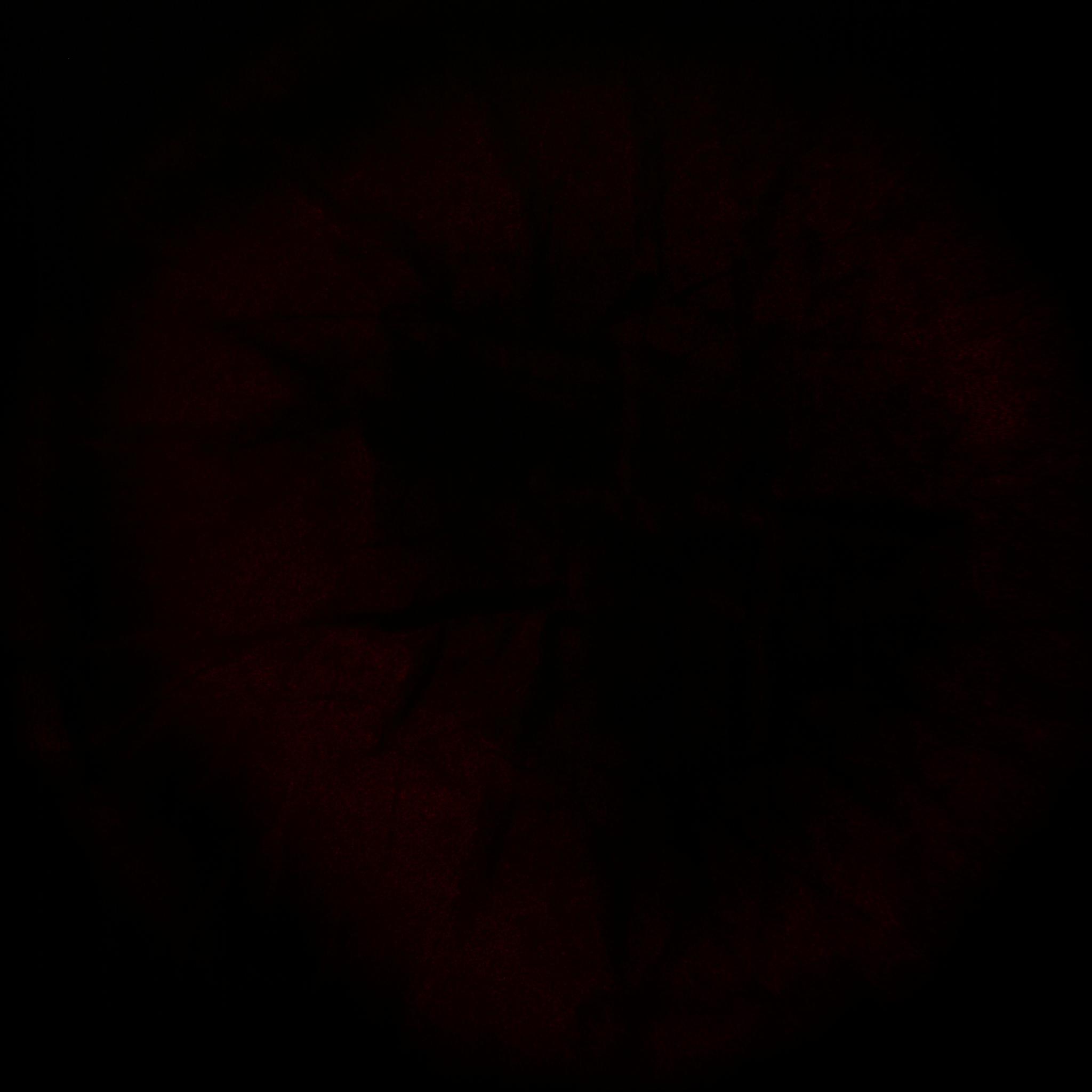

Supplement: S1 File — (ZIP) [file pone.0308204.s001.zip › S1 file. Birefringence Images/B-PK/90 degee/2845OD/IW6.jpg]

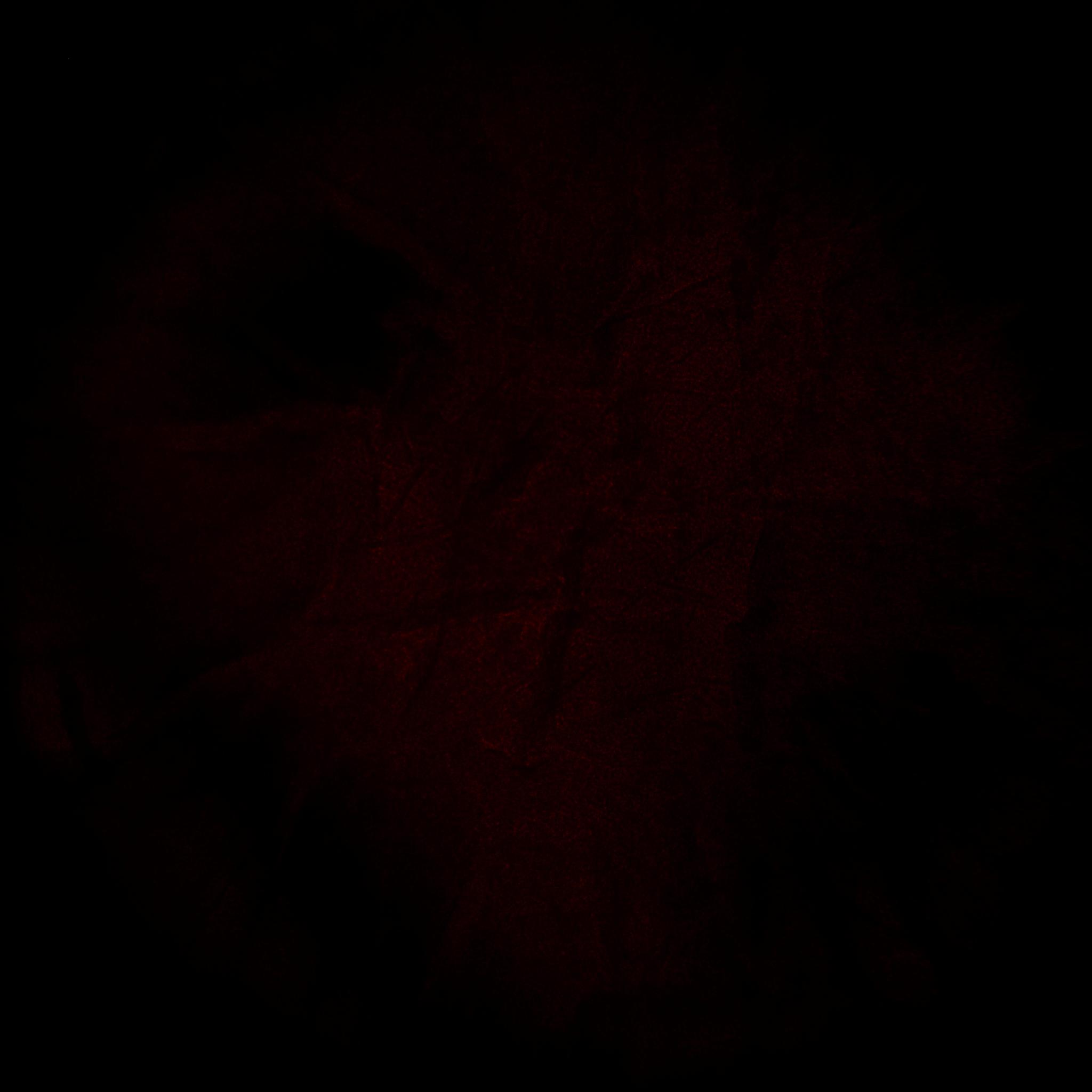

Supplement: S1 File — (ZIP) [file pone.0308204.s001.zip › S1 file. Birefringence Images/B-PK/90 degee/2845OD/IW7.jpg]

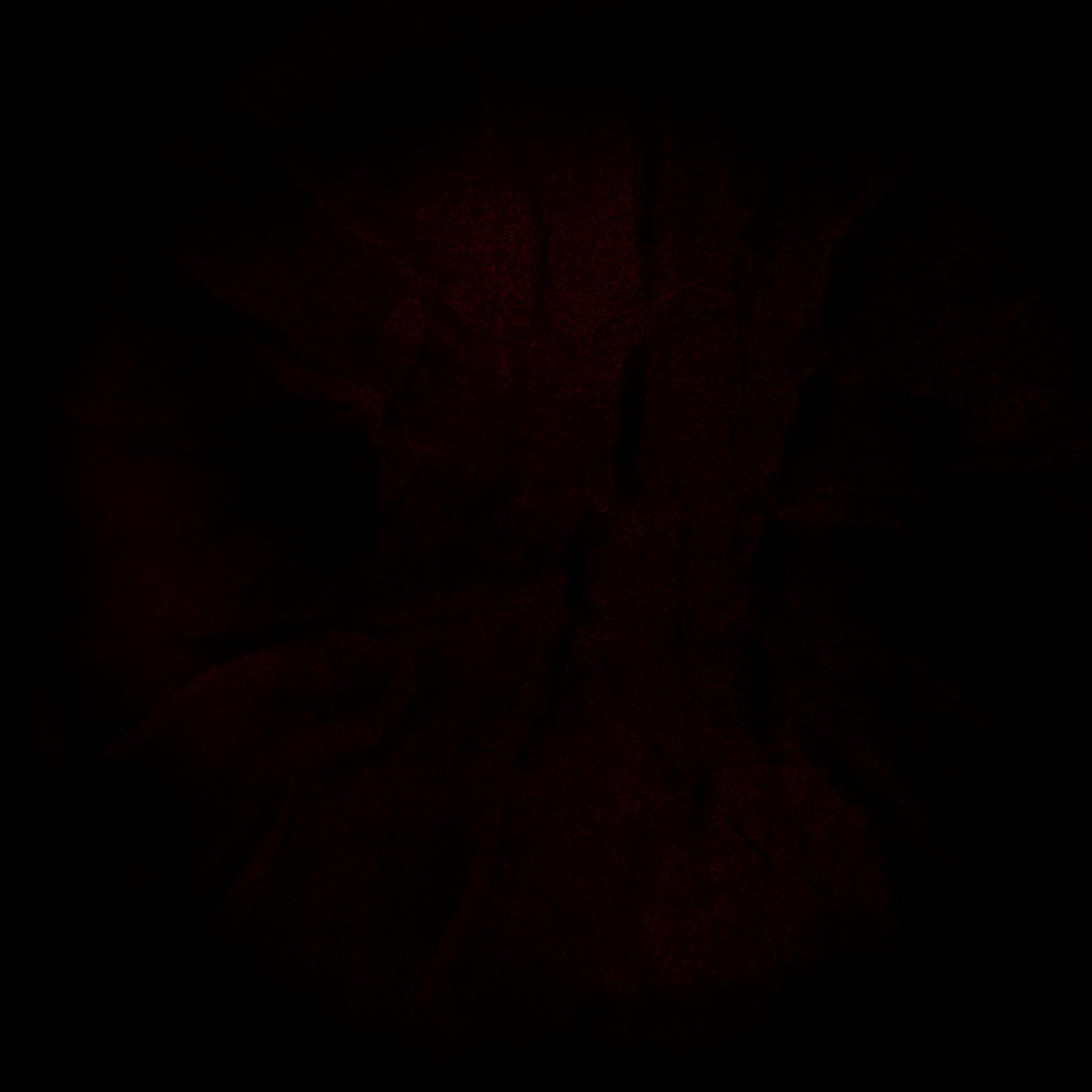

Supplement: S1 File — (ZIP) [file pone.0308204.s001.zip › S1 file. Birefringence Images/B-PK/90 degee/2845OD/IW8.jpg]

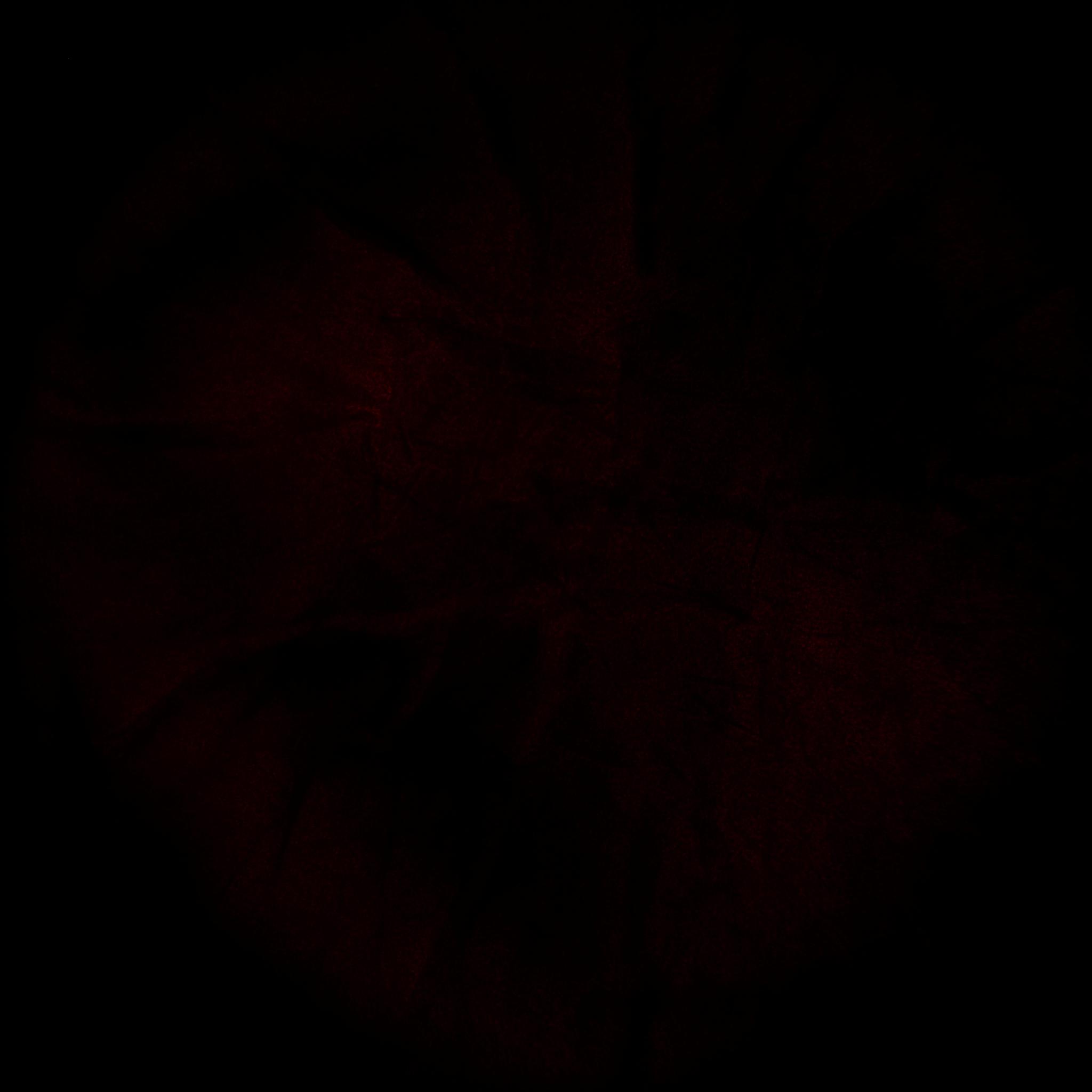

Supplement: S1 File — (ZIP) [file pone.0308204.s001.zip › S1 file. Birefringence Images/B-PK/90 degee/2845OD/IW9.jpg]

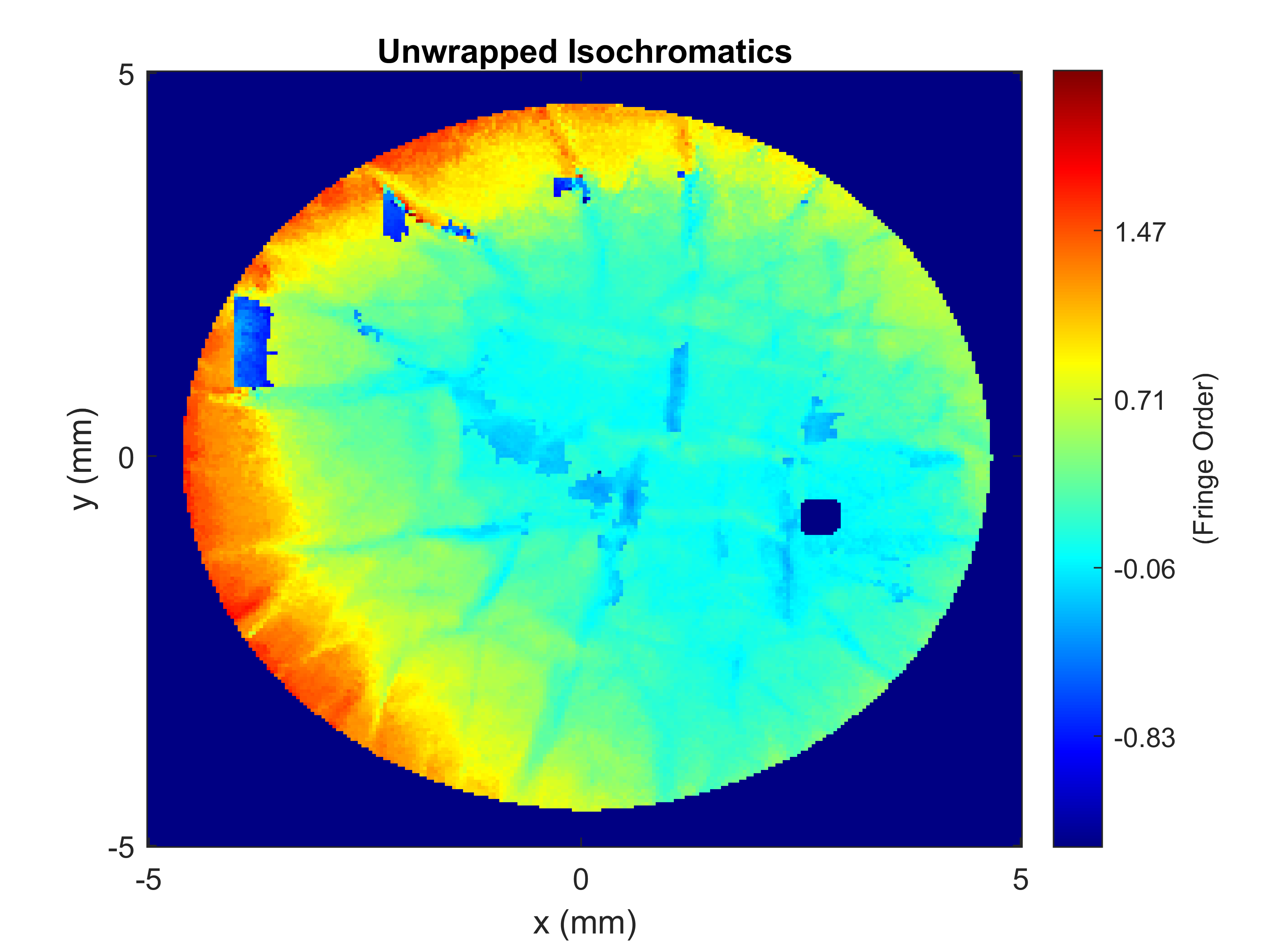

Supplement: S1 File — (ZIP) [file pone.0308204.s001.zip › S1 file. Birefringence Images/B-PK/90 degee/2845OD/unwrappedISOCHcolor.tif]

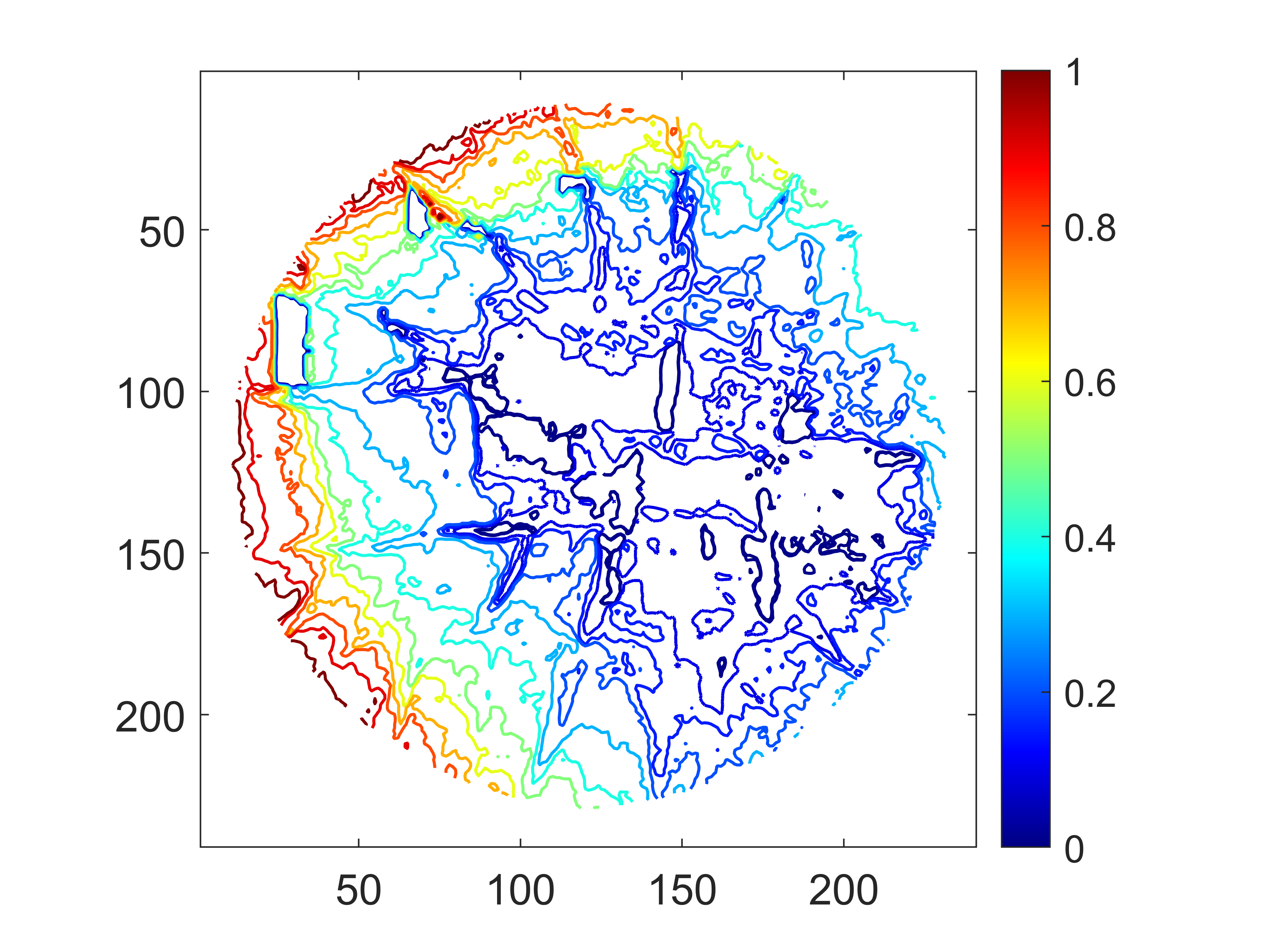

Supplement: S1 File — (ZIP) [file pone.0308204.s001.zip › S1 file. Birefringence Images/B-PK/90 degee/2845OD/unwrappedISOCHcolorconour.tif]

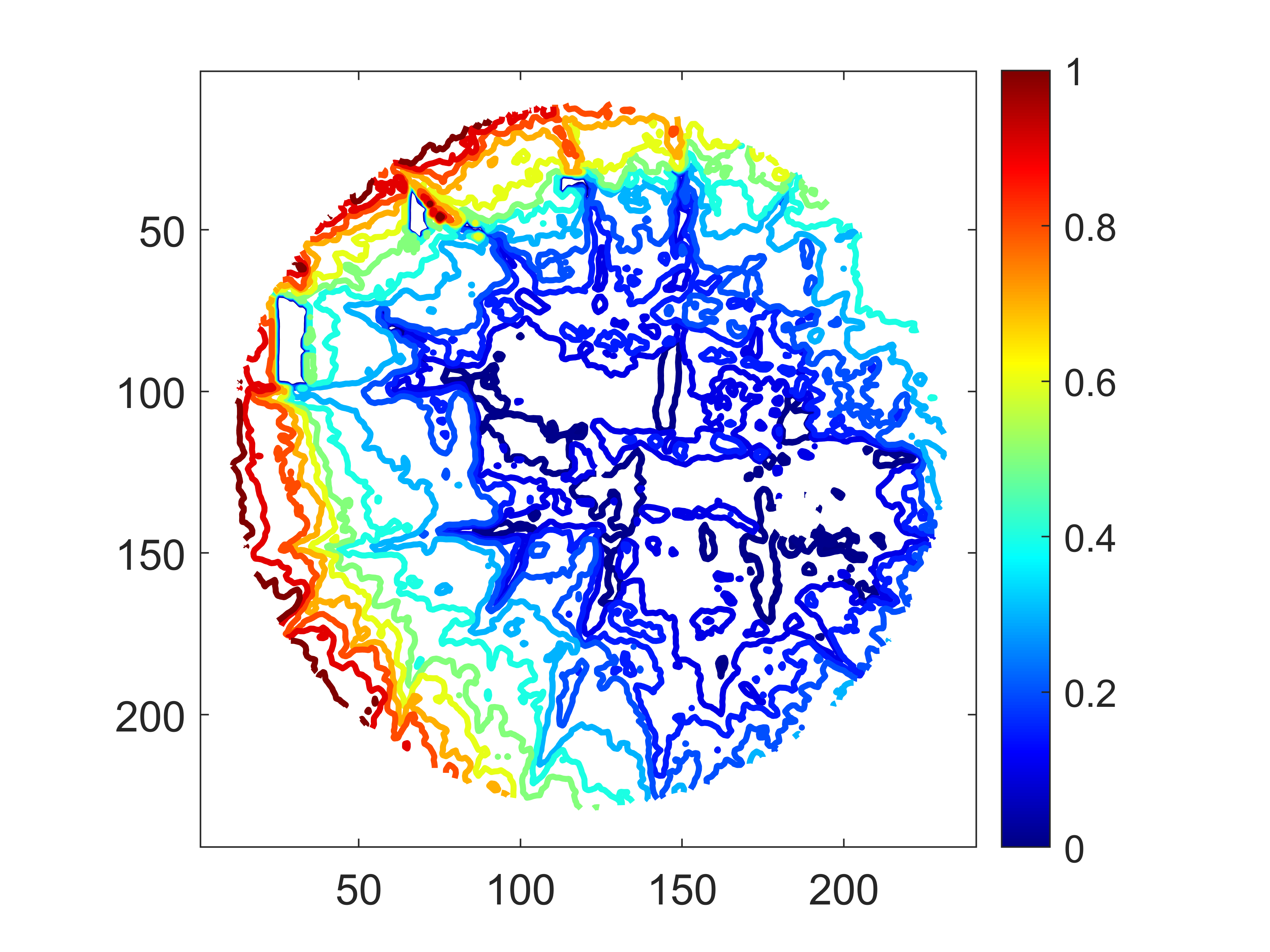

Supplement: S1 File — (ZIP) [file pone.0308204.s001.zip › S1 file. Birefringence Images/B-PK/90 degee/2845OD/unwrappedISOCHcolorconour2.tif]

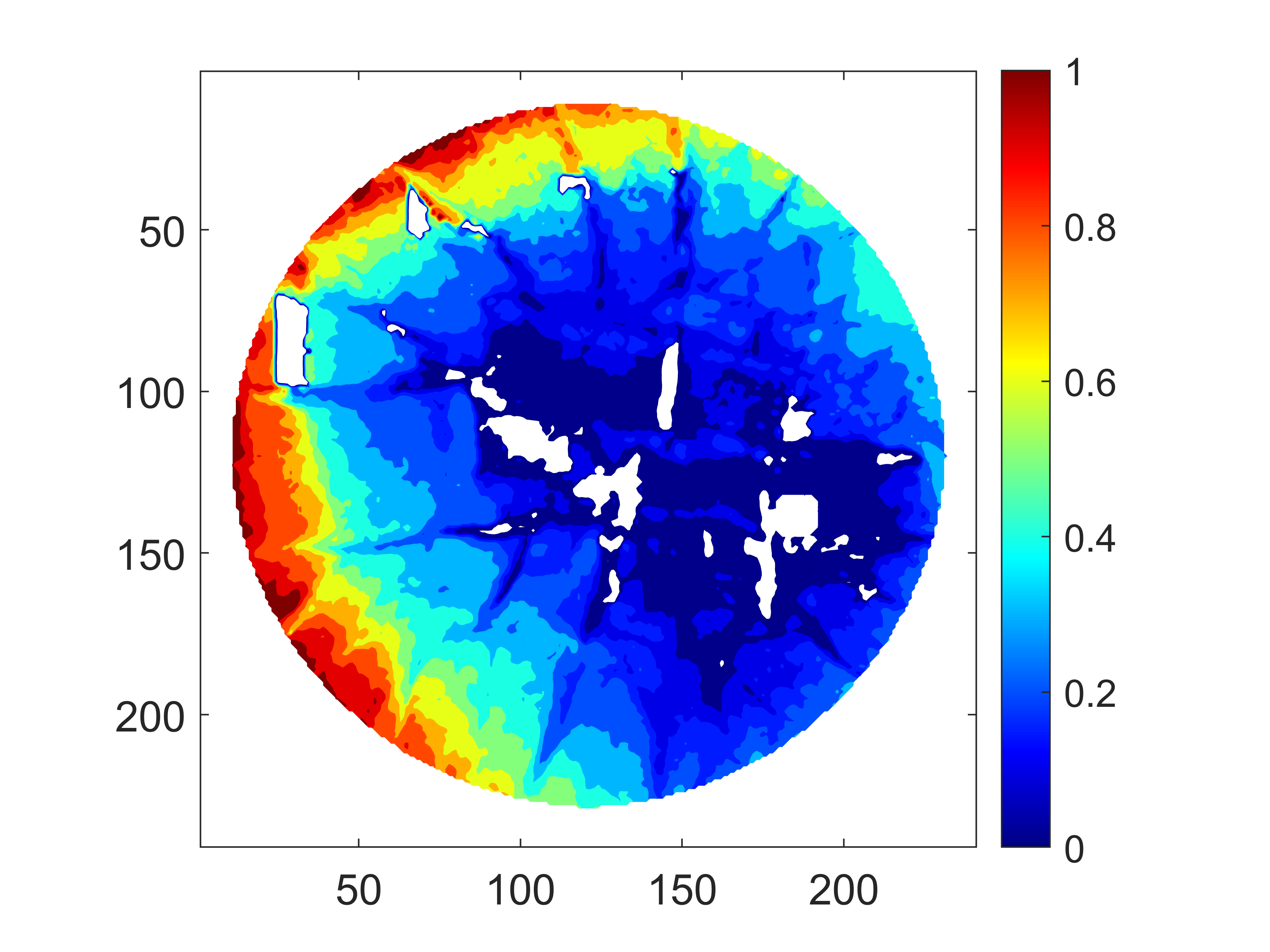

Supplement: S1 File — (ZIP) [file pone.0308204.s001.zip › S1 file. Birefringence Images/B-PK/90 degee/2845OD/unwrappedISOCHcolorfilled.tif]

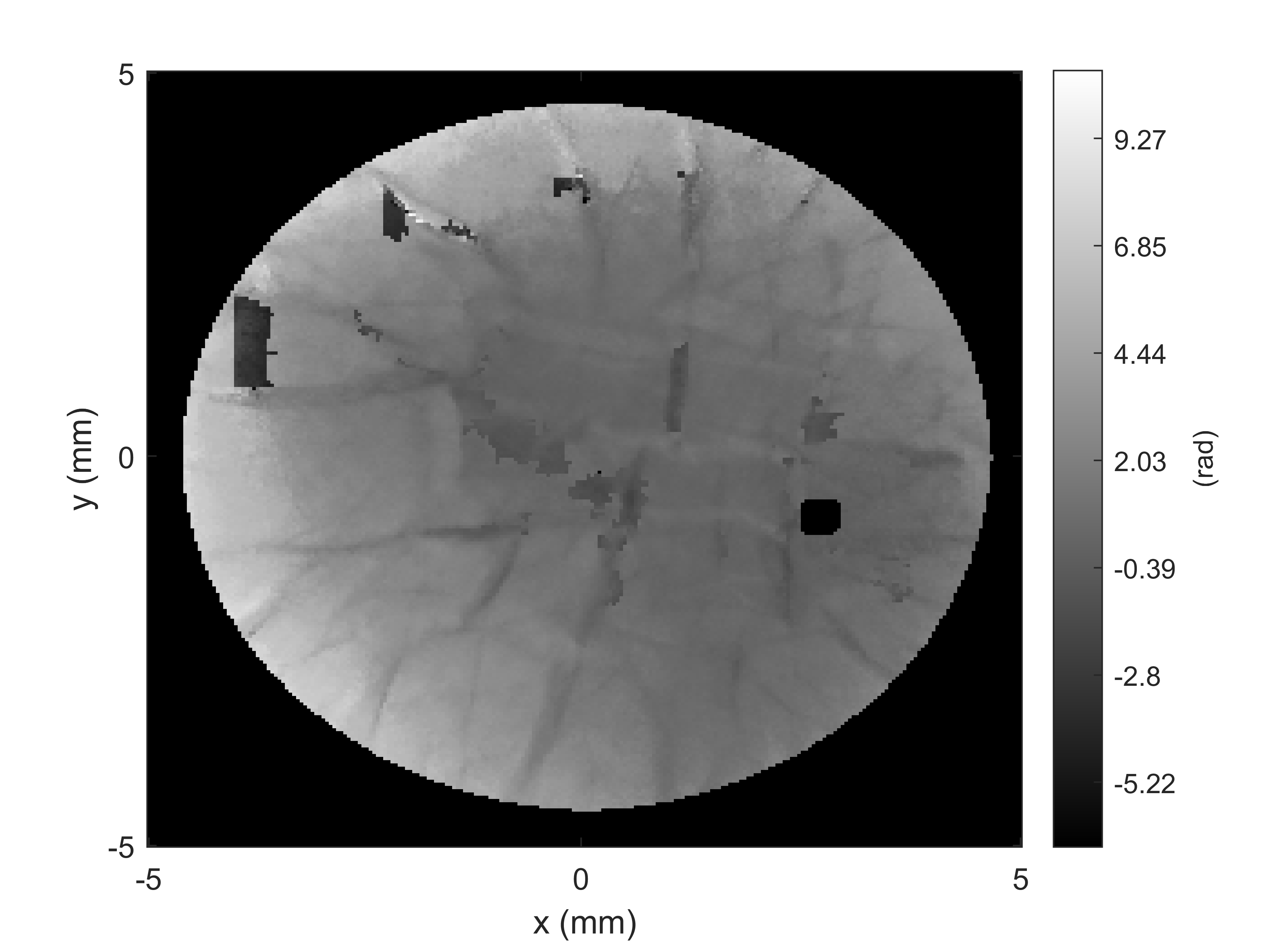

Supplement: S1 File — (ZIP) [file pone.0308204.s001.zip › S1 file. Birefringence Images/B-PK/90 degee/2845OD/unwrappedISOCHgay.tif]

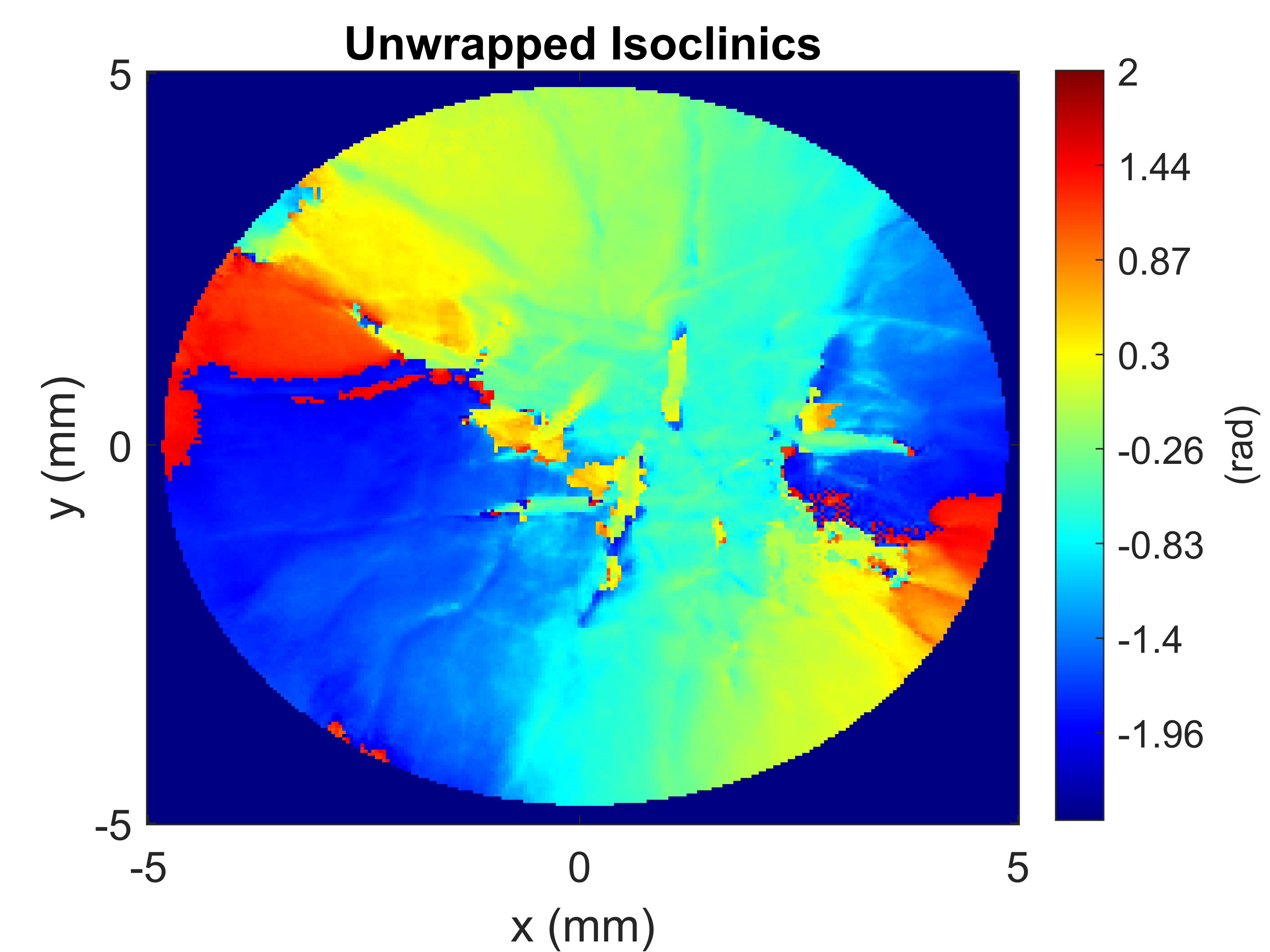

Supplement: S1 File — (ZIP) [file pone.0308204.s001.zip › S1 file. Birefringence Images/B-PK/90 degee/2845OD/unwrappedISOcolor.tif]

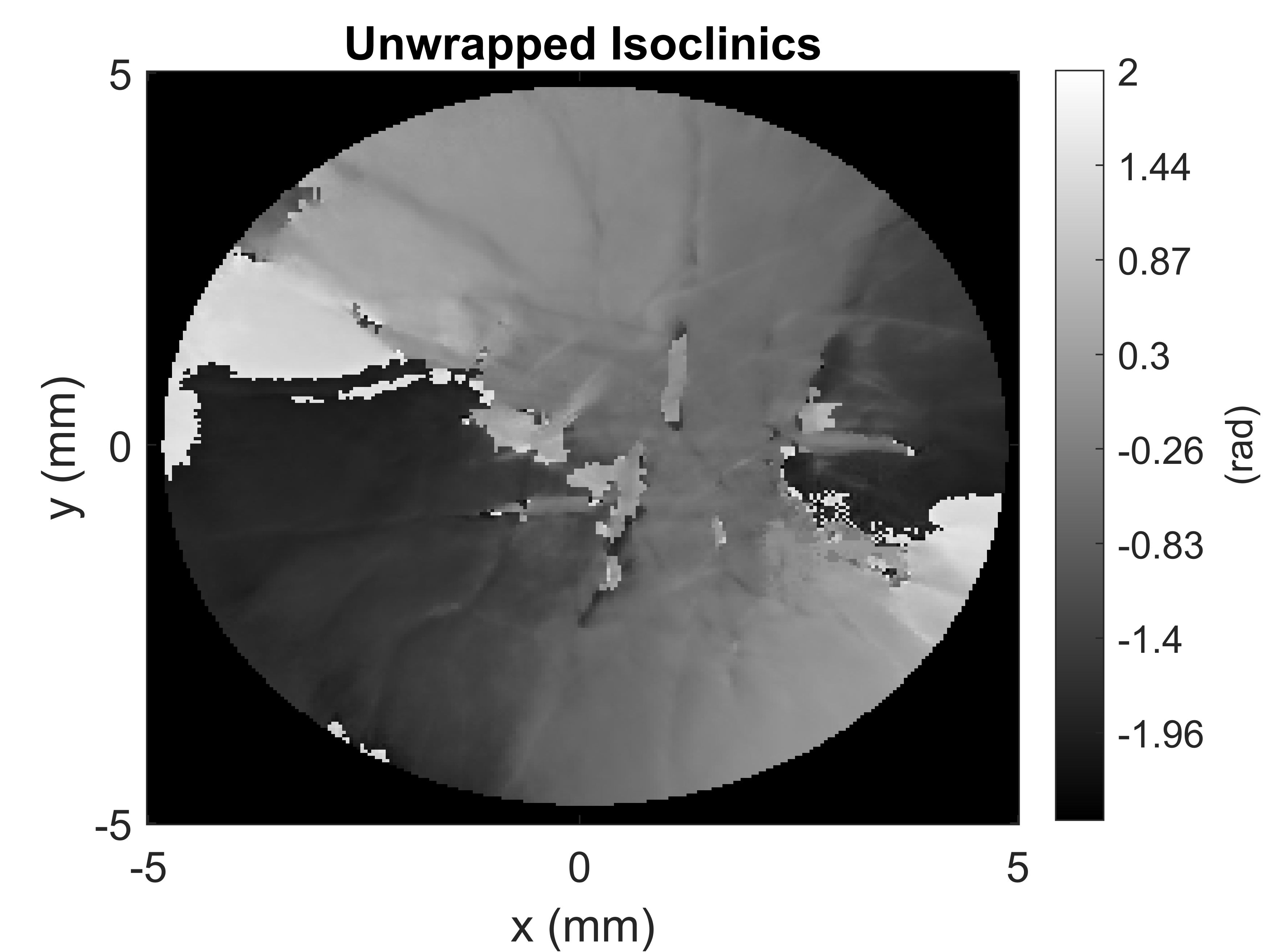

Supplement: S1 File — (ZIP) [file pone.0308204.s001.zip › S1 file. Birefringence Images/B-PK/90 degee/2845OD/unwrappedISOgray.tif]

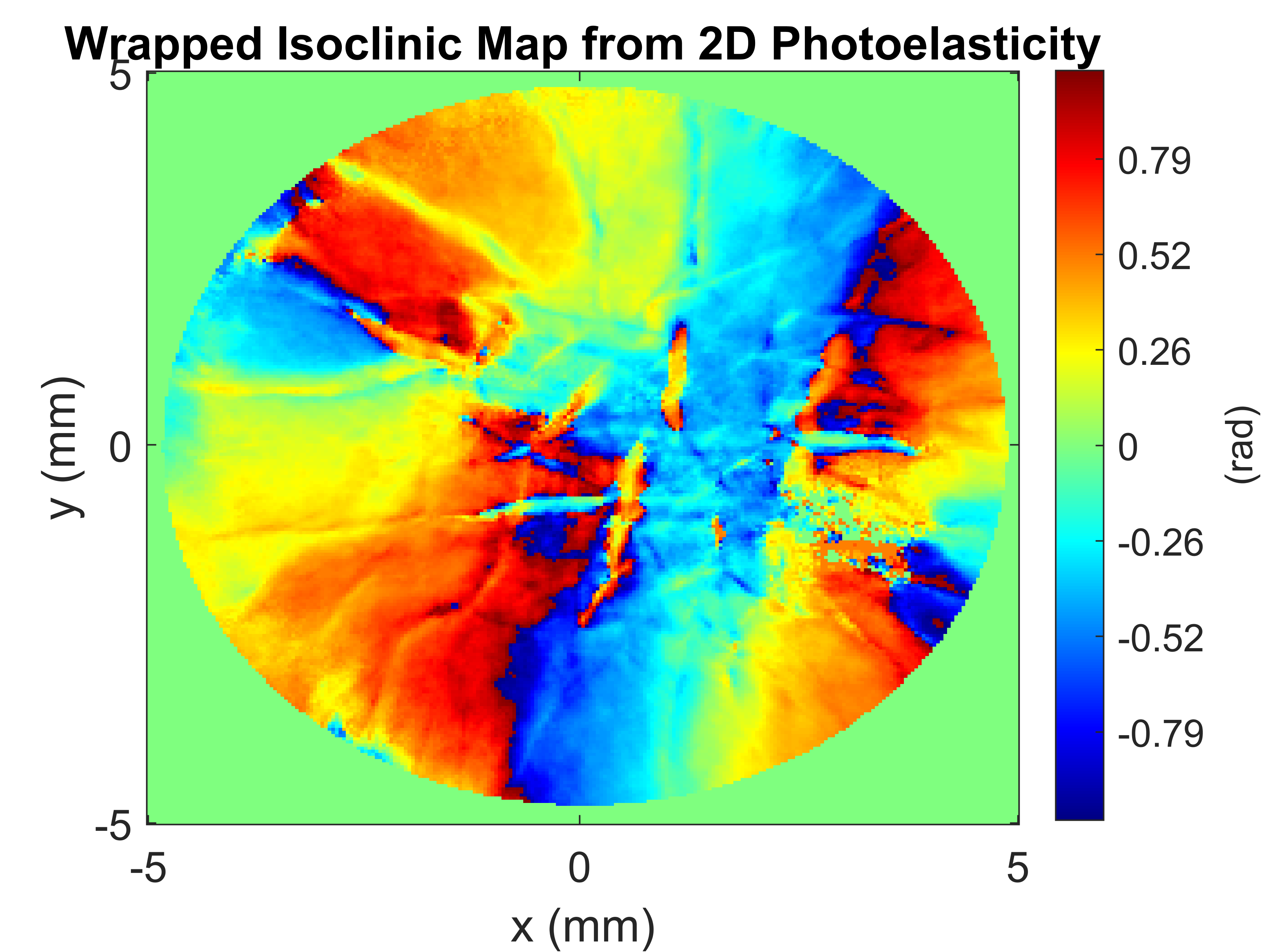

Supplement: S1 File — (ZIP) [file pone.0308204.s001.zip › S1 file. Birefringence Images/B-PK/90 degee/2845OD/wappedISOcolor.tif]

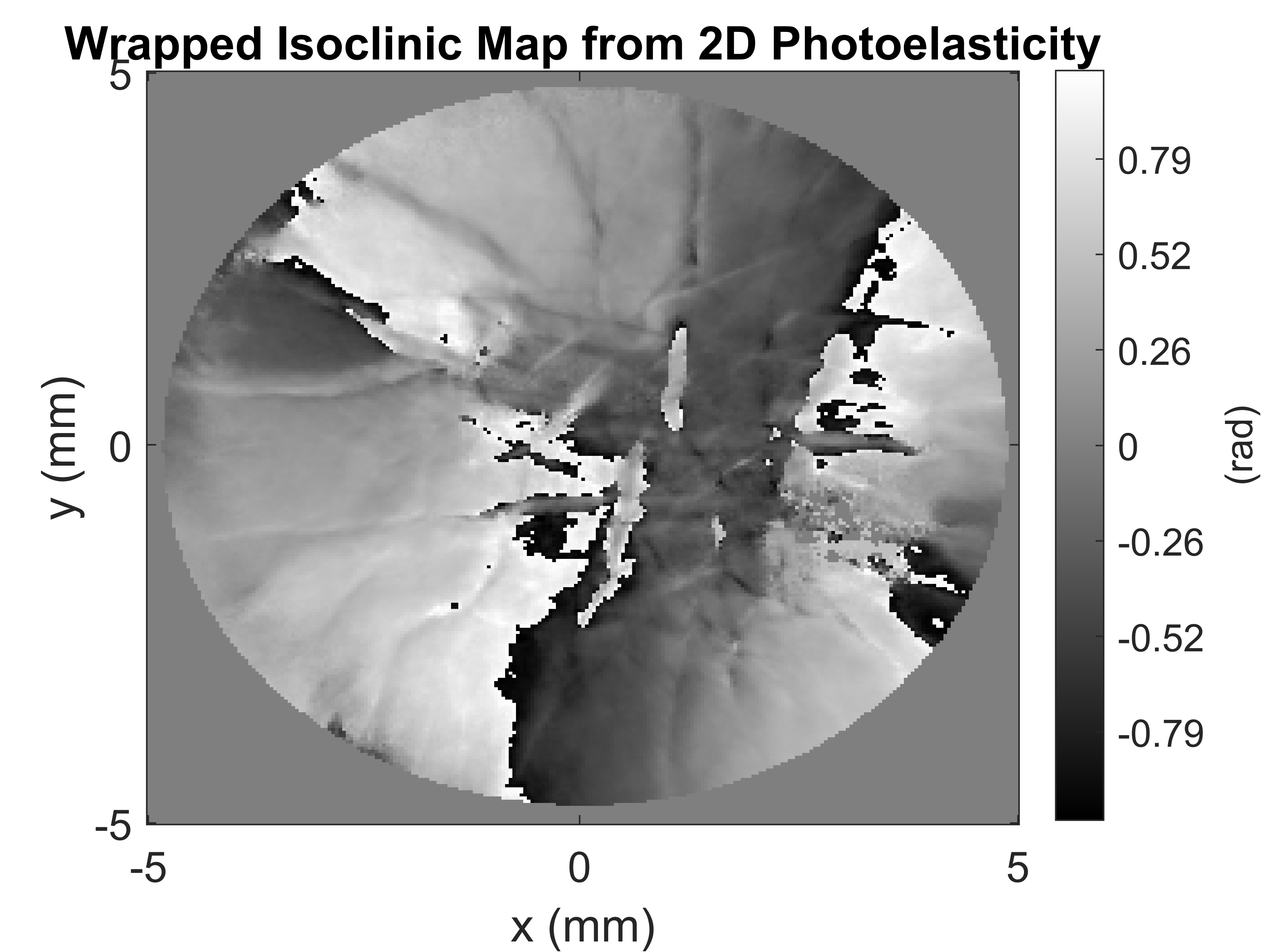

Supplement: S1 File — (ZIP) [file pone.0308204.s001.zip › S1 file. Birefringence Images/B-PK/90 degee/2845OD/wappedISOgray.tif]
