# Supplementary figures and images for: Collagen imaging reveals synergistic effects of sutures and host-donor misalignment on topographical irregularities in penetrating keratoplasty (part 1 of 2)
Source: PLoS One. 2024 Aug 8;19(8):e0308204. doi: 10.1371/journal.pone.0308204 (PMC11309498; doi:10.1371/journal.pone.0308204)

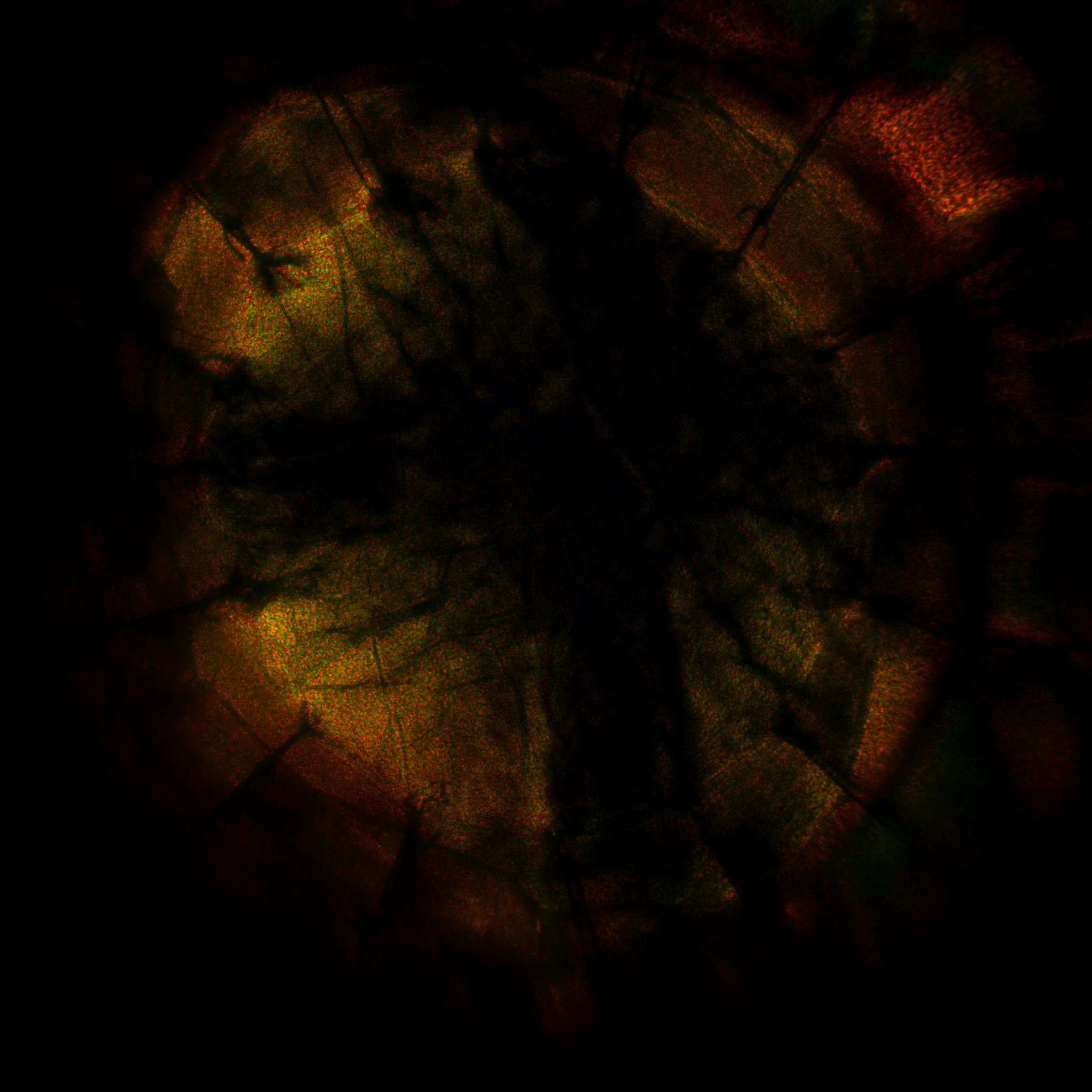

Supplement: S1 File — (ZIP) [file pone.0308204.s001.zip › S1 file. Birefringence Images/A-PK/0 degee/2349OD/IW1.jpg]

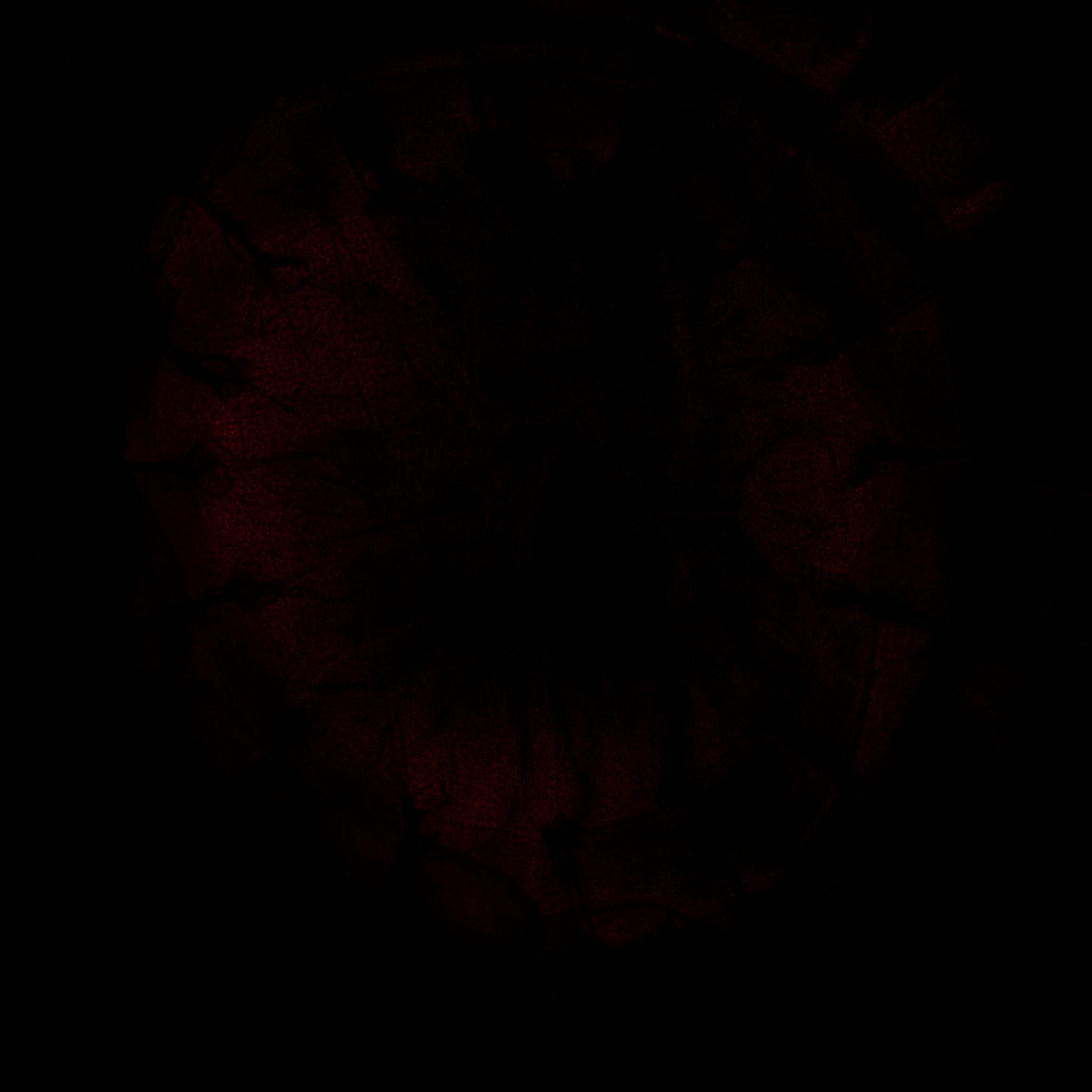

Supplement: S1 File — (ZIP) [file pone.0308204.s001.zip › S1 file. Birefringence Images/A-PK/0 degee/2349OD/IW10.jpg]

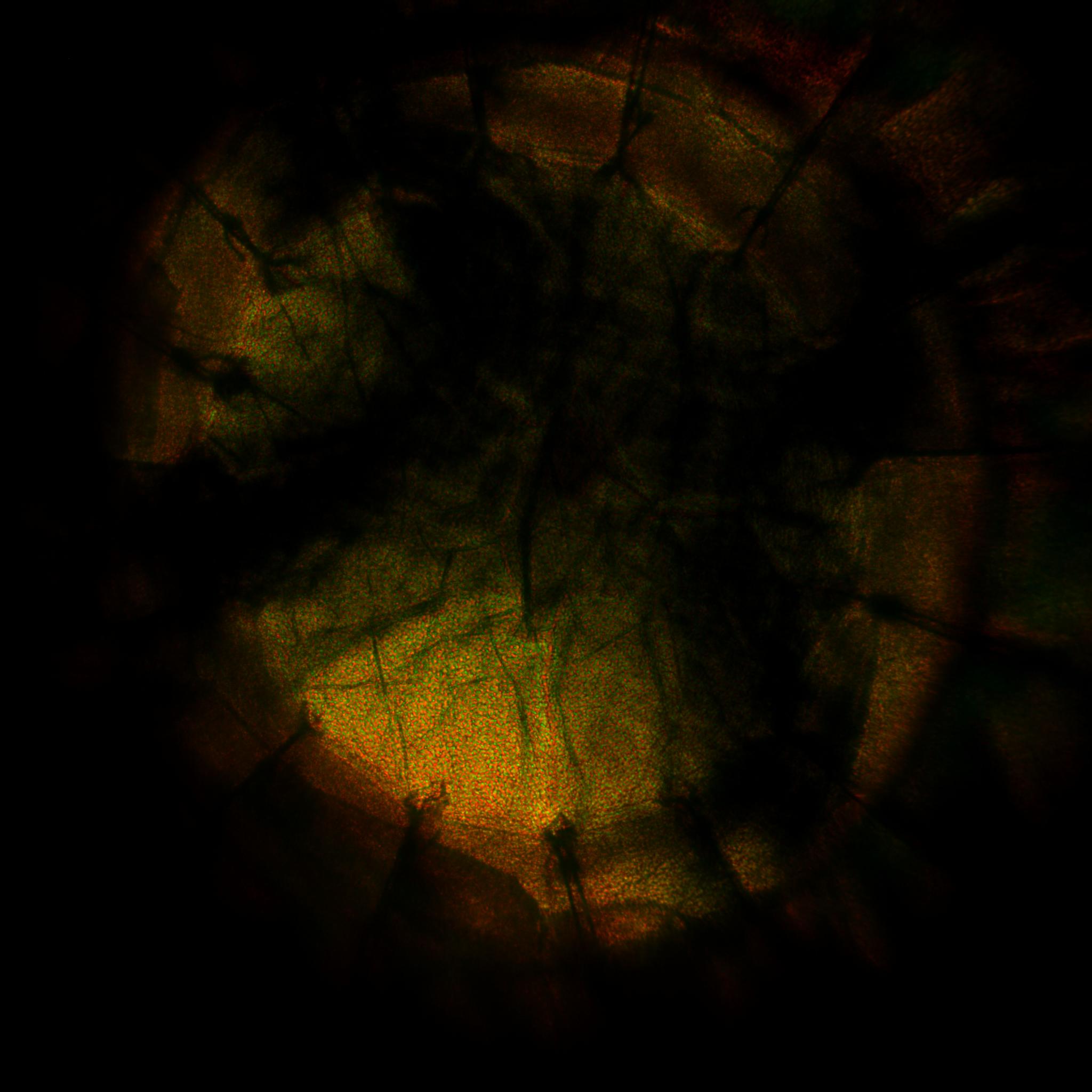

Supplement: S1 File — (ZIP) [file pone.0308204.s001.zip › S1 file. Birefringence Images/A-PK/0 degee/2349OD/IW2.jpg]

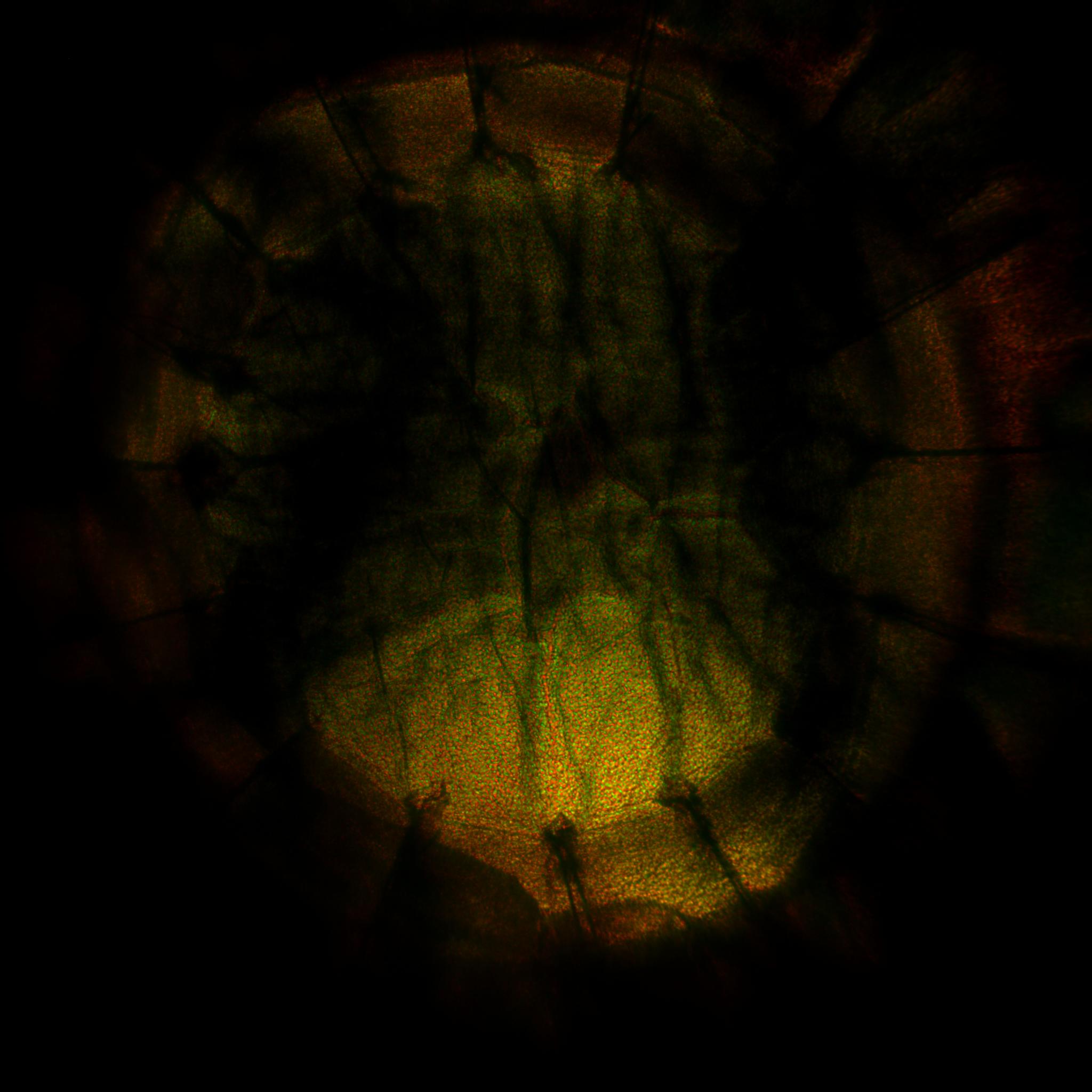

Supplement: S1 File — (ZIP) [file pone.0308204.s001.zip › S1 file. Birefringence Images/A-PK/0 degee/2349OD/IW3.jpg]

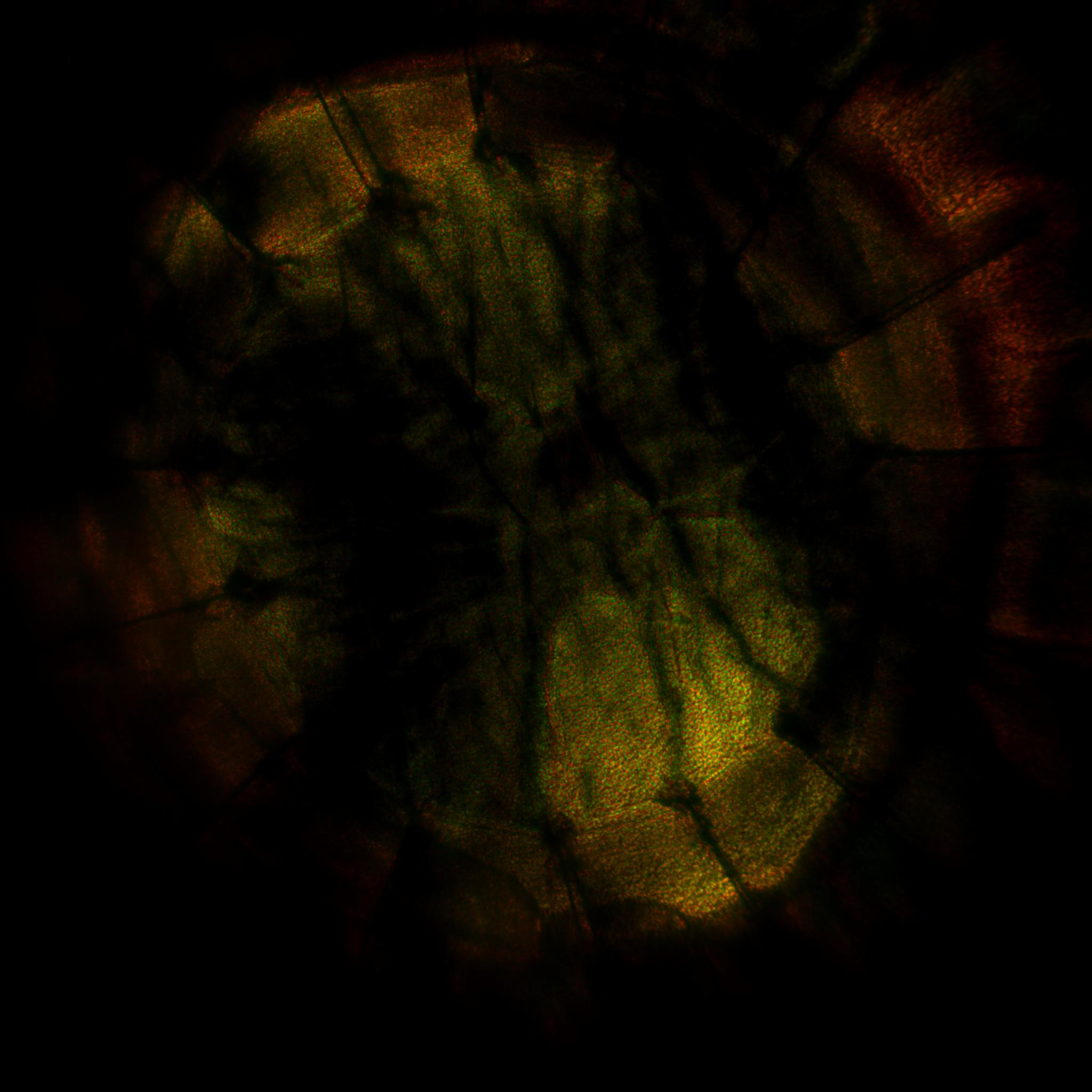

Supplement: S1 File — (ZIP) [file pone.0308204.s001.zip › S1 file. Birefringence Images/A-PK/0 degee/2349OD/IW4.jpg]

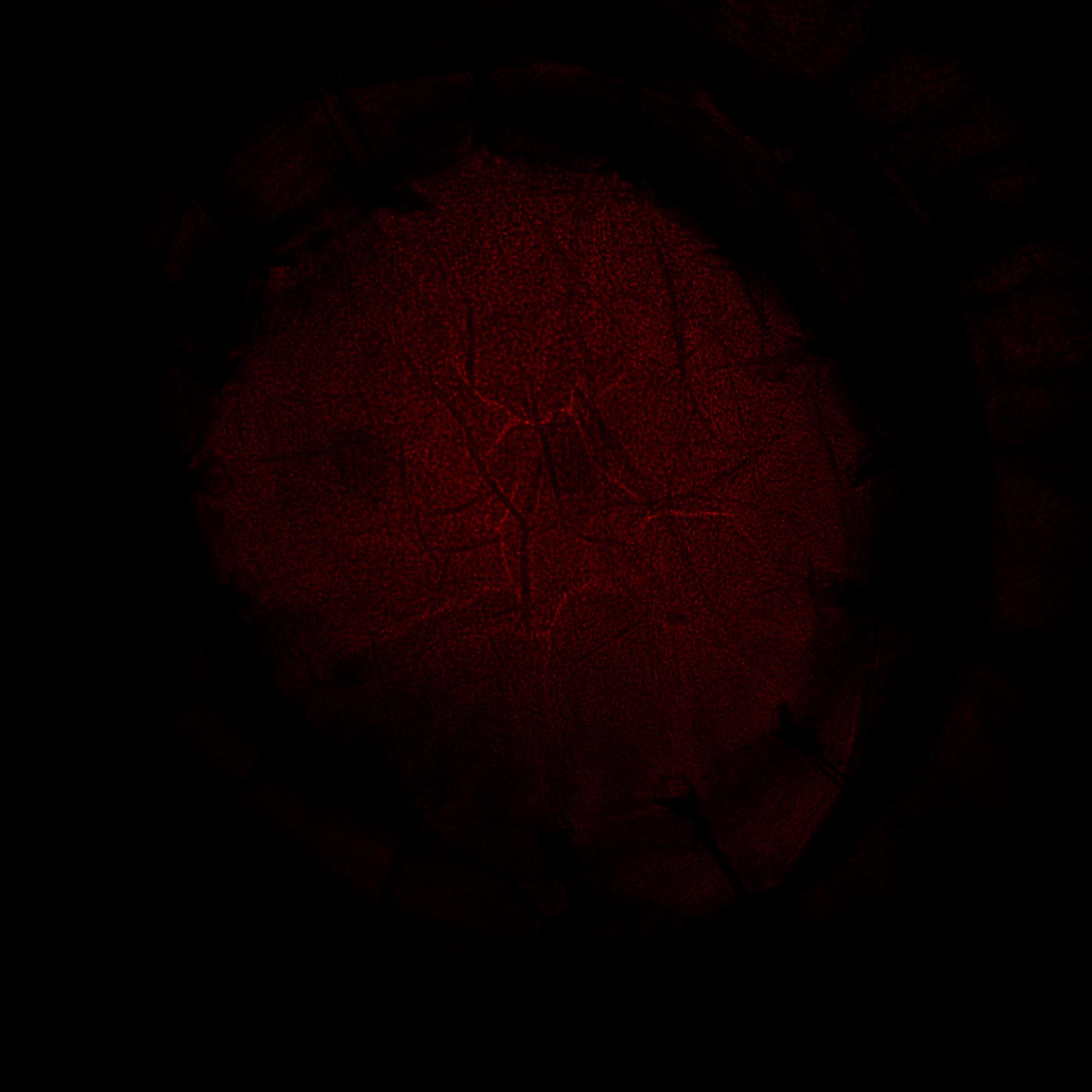

Supplement: S1 File — (ZIP) [file pone.0308204.s001.zip › S1 file. Birefringence Images/A-PK/0 degee/2349OD/IW5.jpg]

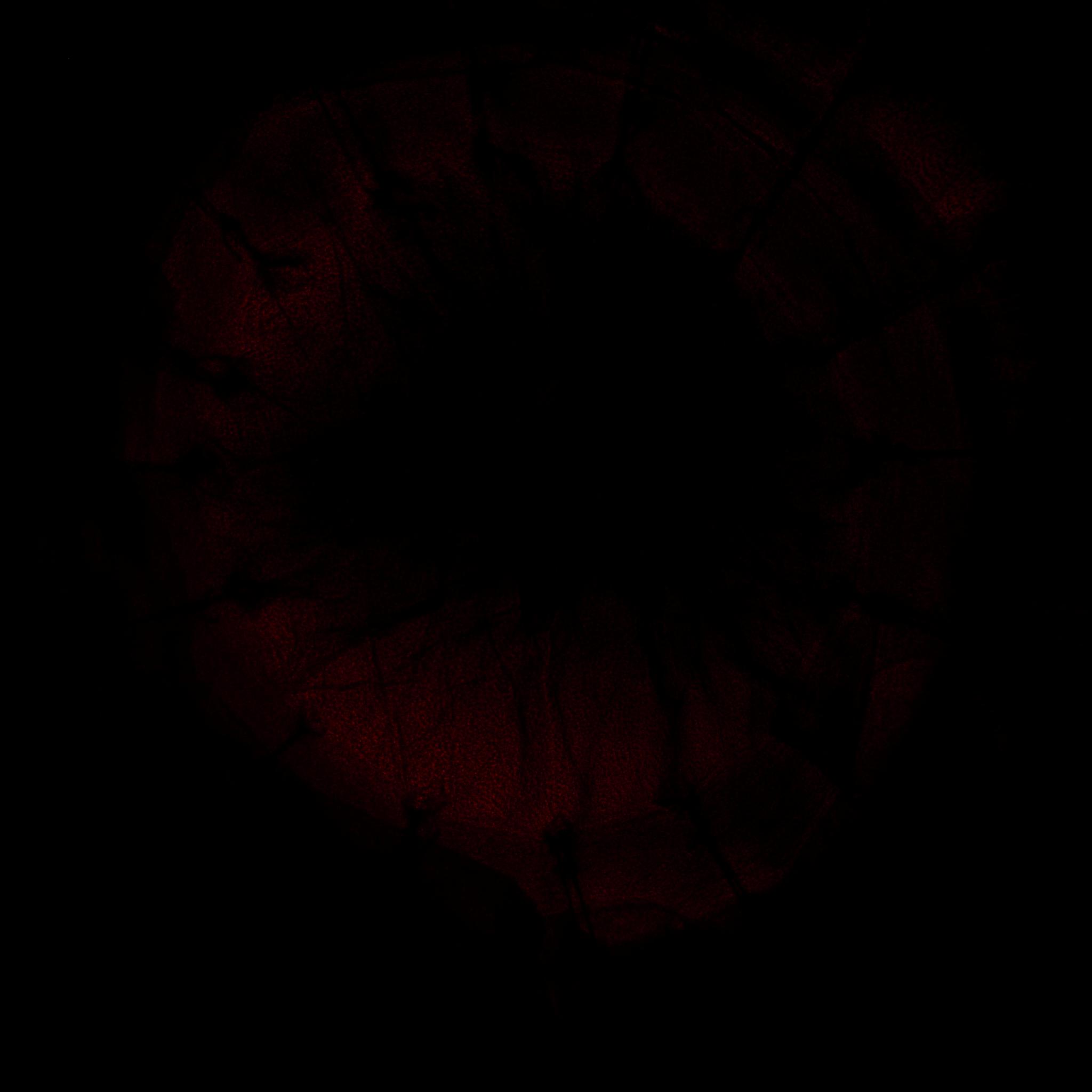

Supplement: S1 File — (ZIP) [file pone.0308204.s001.zip › S1 file. Birefringence Images/A-PK/0 degee/2349OD/IW6.jpg]

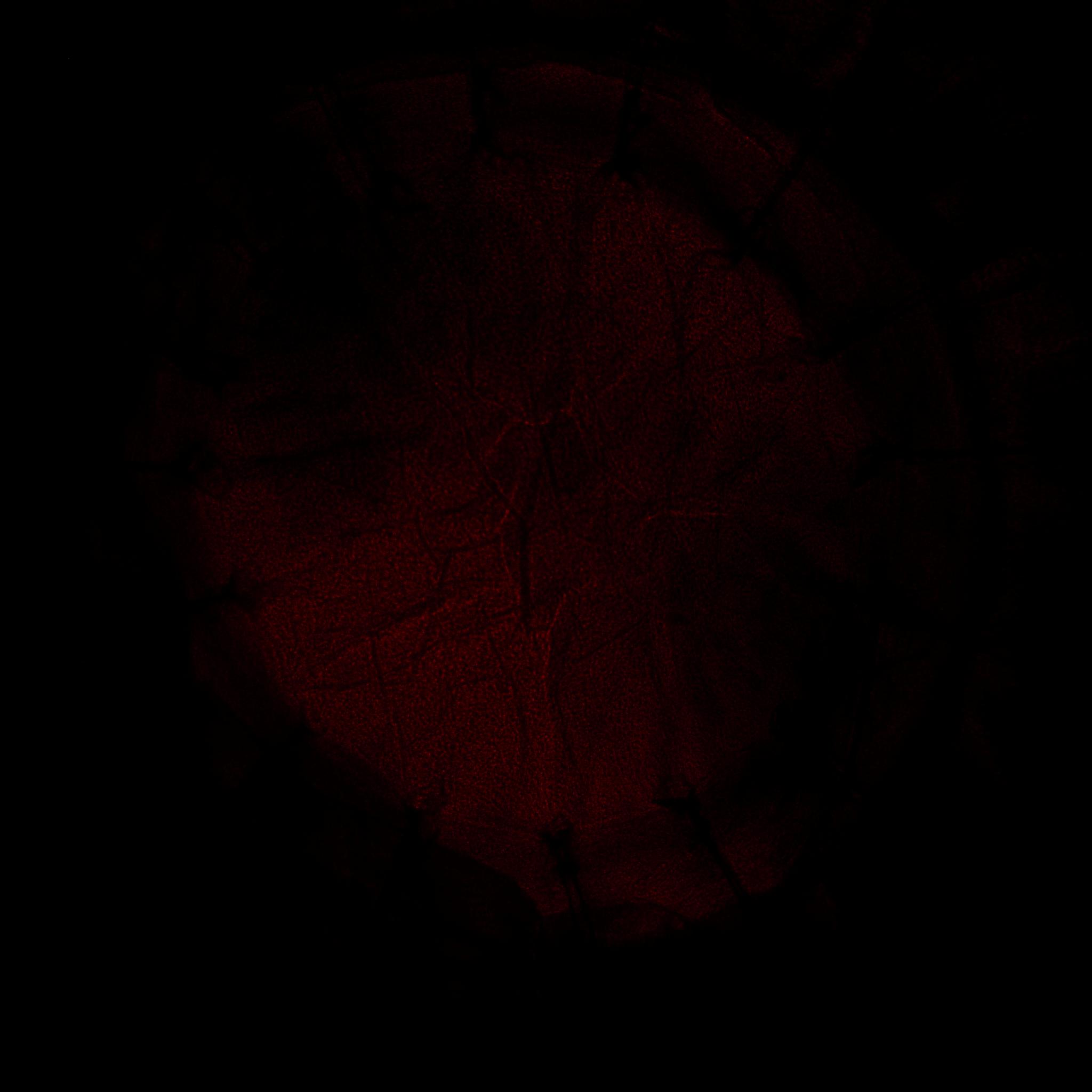

Supplement: S1 File — (ZIP) [file pone.0308204.s001.zip › S1 file. Birefringence Images/A-PK/0 degee/2349OD/IW7.jpg]

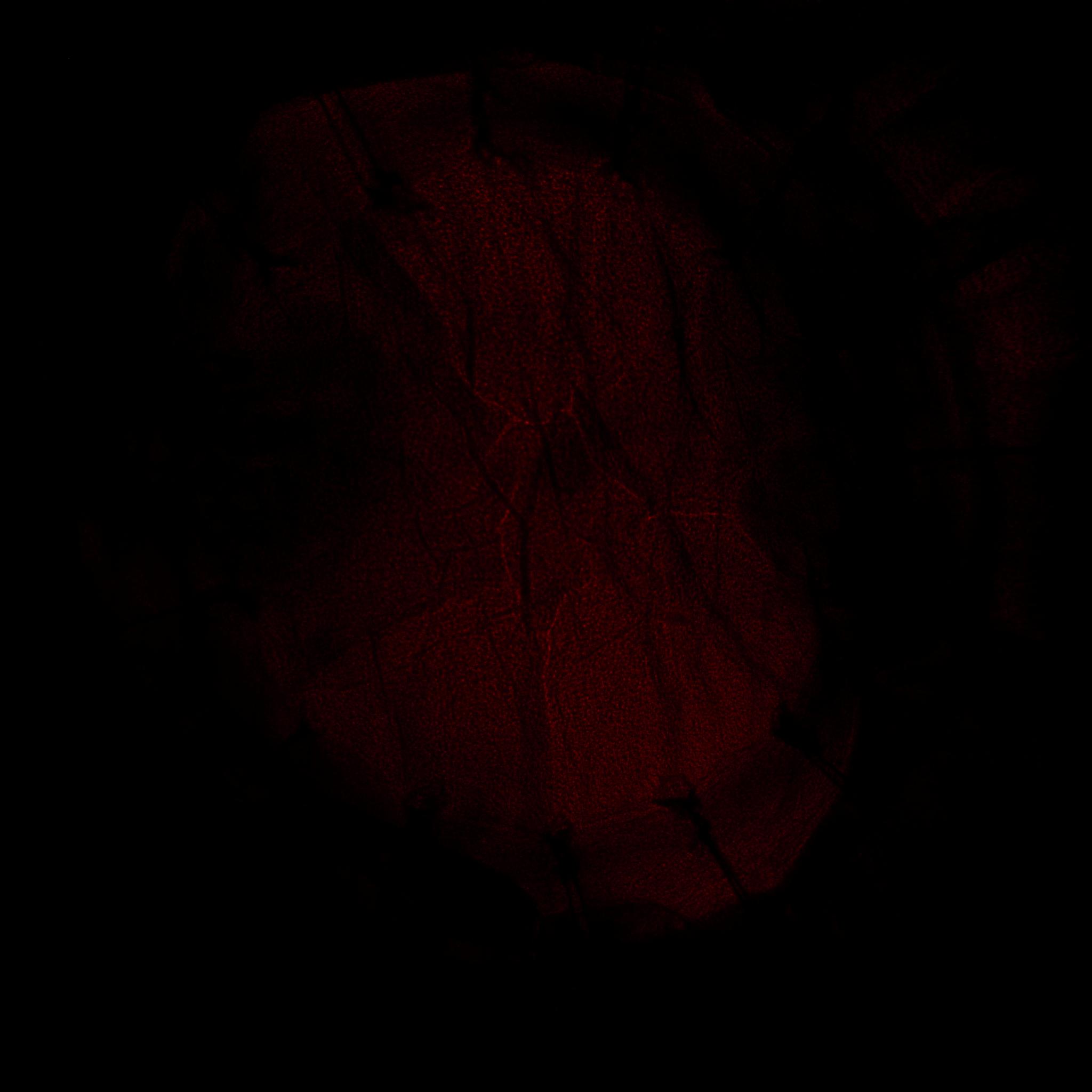

Supplement: S1 File — (ZIP) [file pone.0308204.s001.zip › S1 file. Birefringence Images/A-PK/0 degee/2349OD/IW8.jpg]

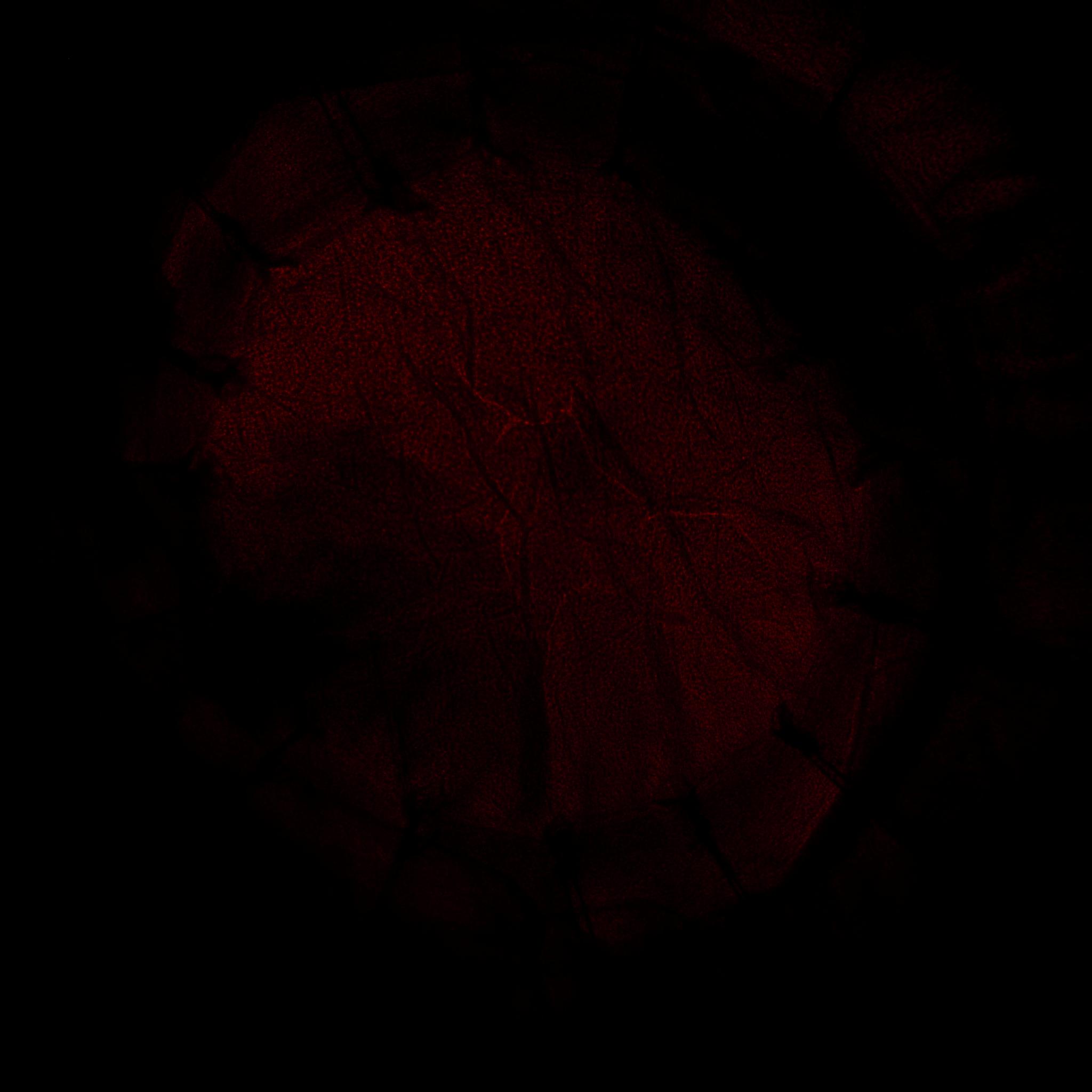

Supplement: S1 File — (ZIP) [file pone.0308204.s001.zip › S1 file. Birefringence Images/A-PK/0 degee/2349OD/IW9.jpg]

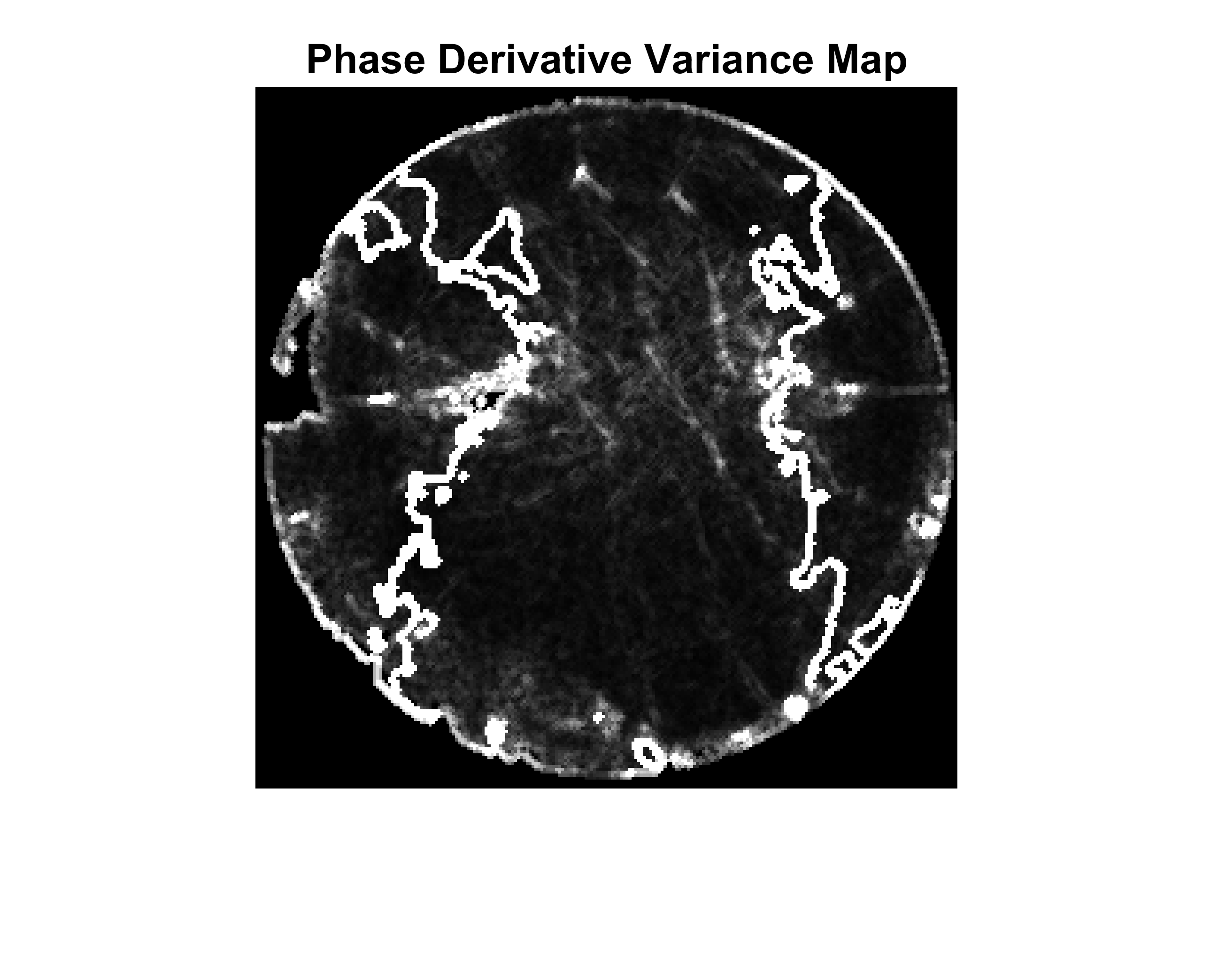

Supplement: S1 File — (ZIP) [file pone.0308204.s001.zip › S1 file. Birefringence Images/A-PK/0 degee/2349OD/PDV.tif]

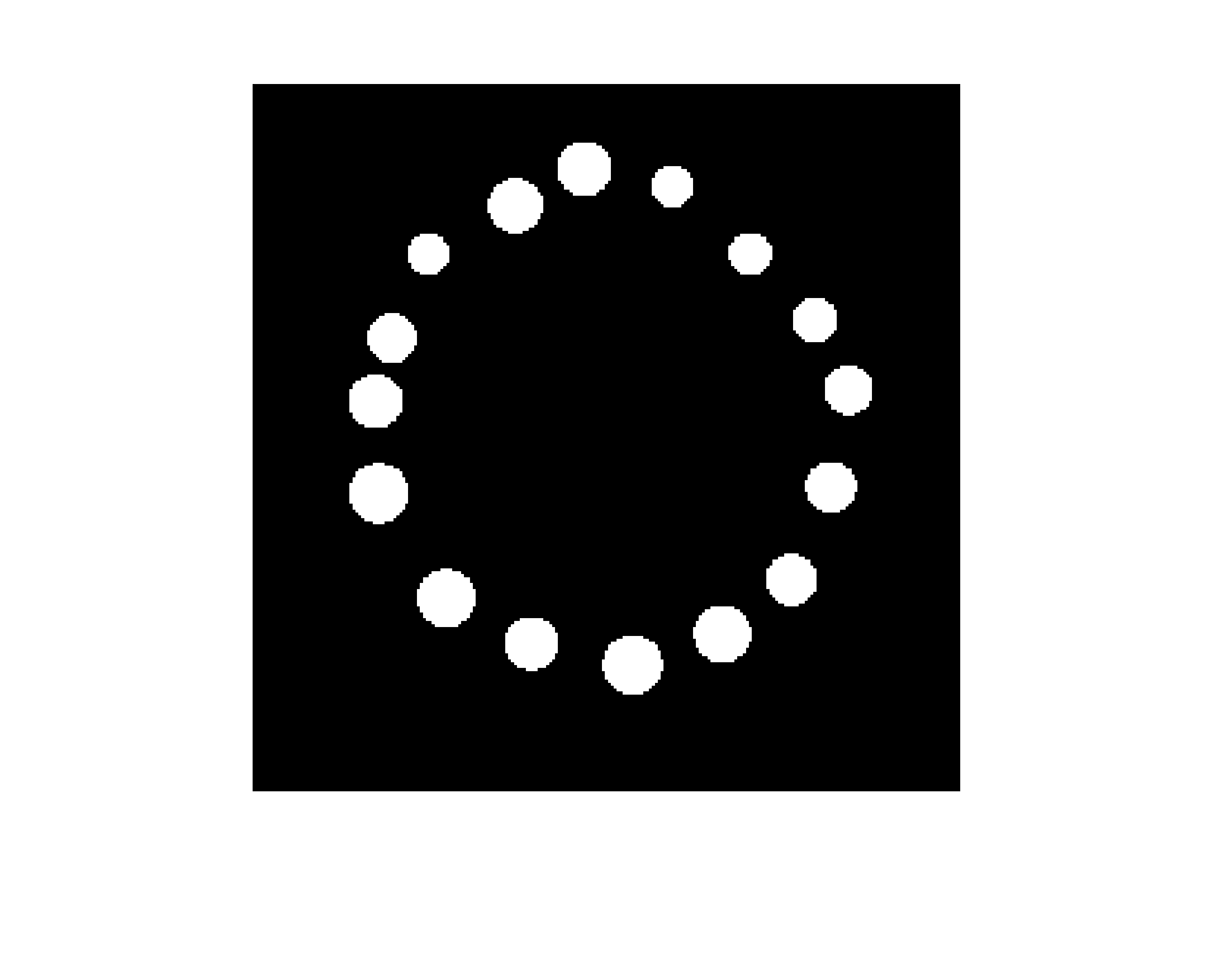

Supplement: S1 File — (ZIP) [file pone.0308204.s001.zip › S1 file. Birefringence Images/A-PK/0 degee/2349OD/suuemask.tif]

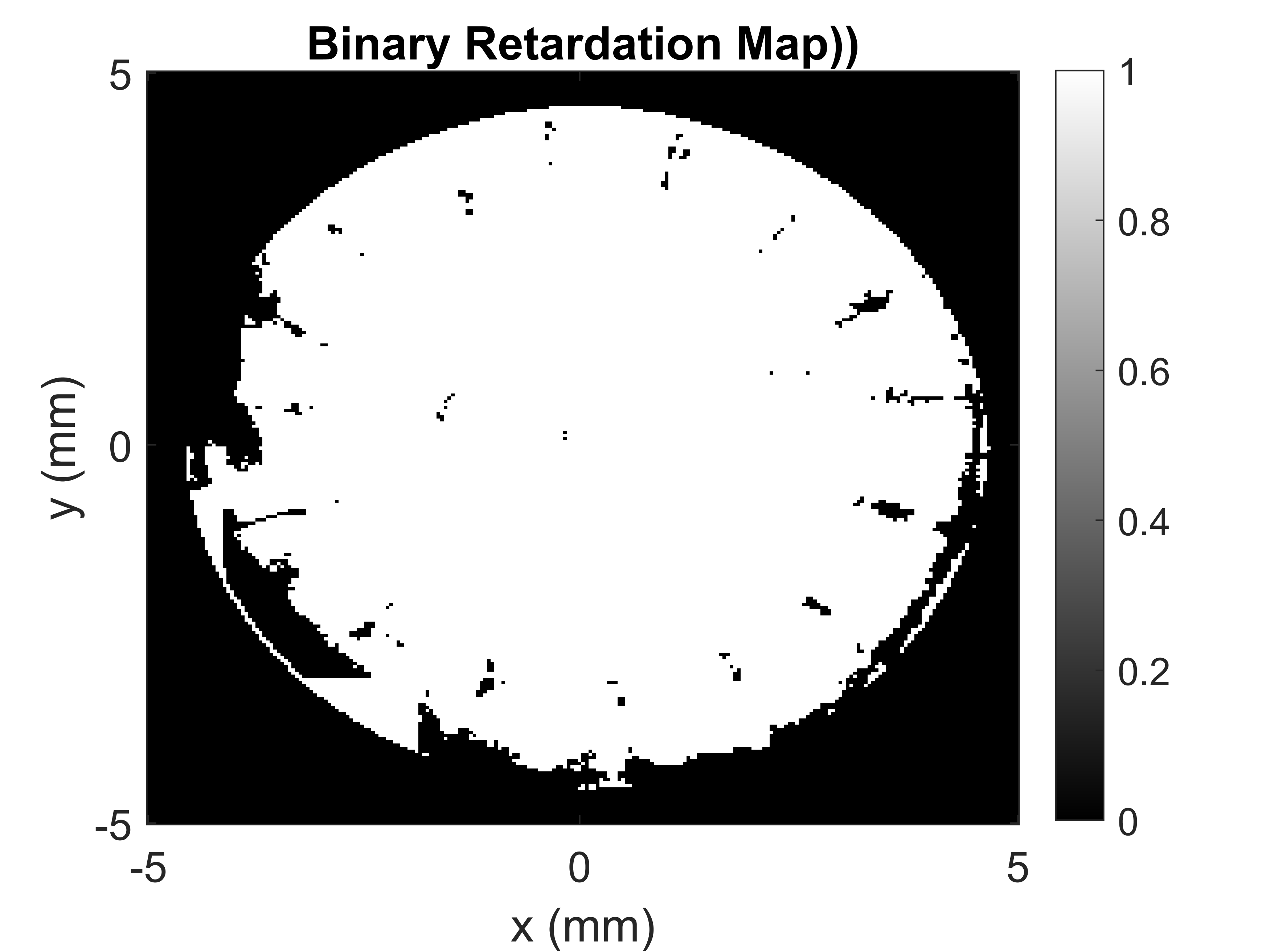

Supplement: S1 File — (ZIP) [file pone.0308204.s001.zip › S1 file. Birefringence Images/A-PK/0 degee/2349OD/suuepoins.tif]

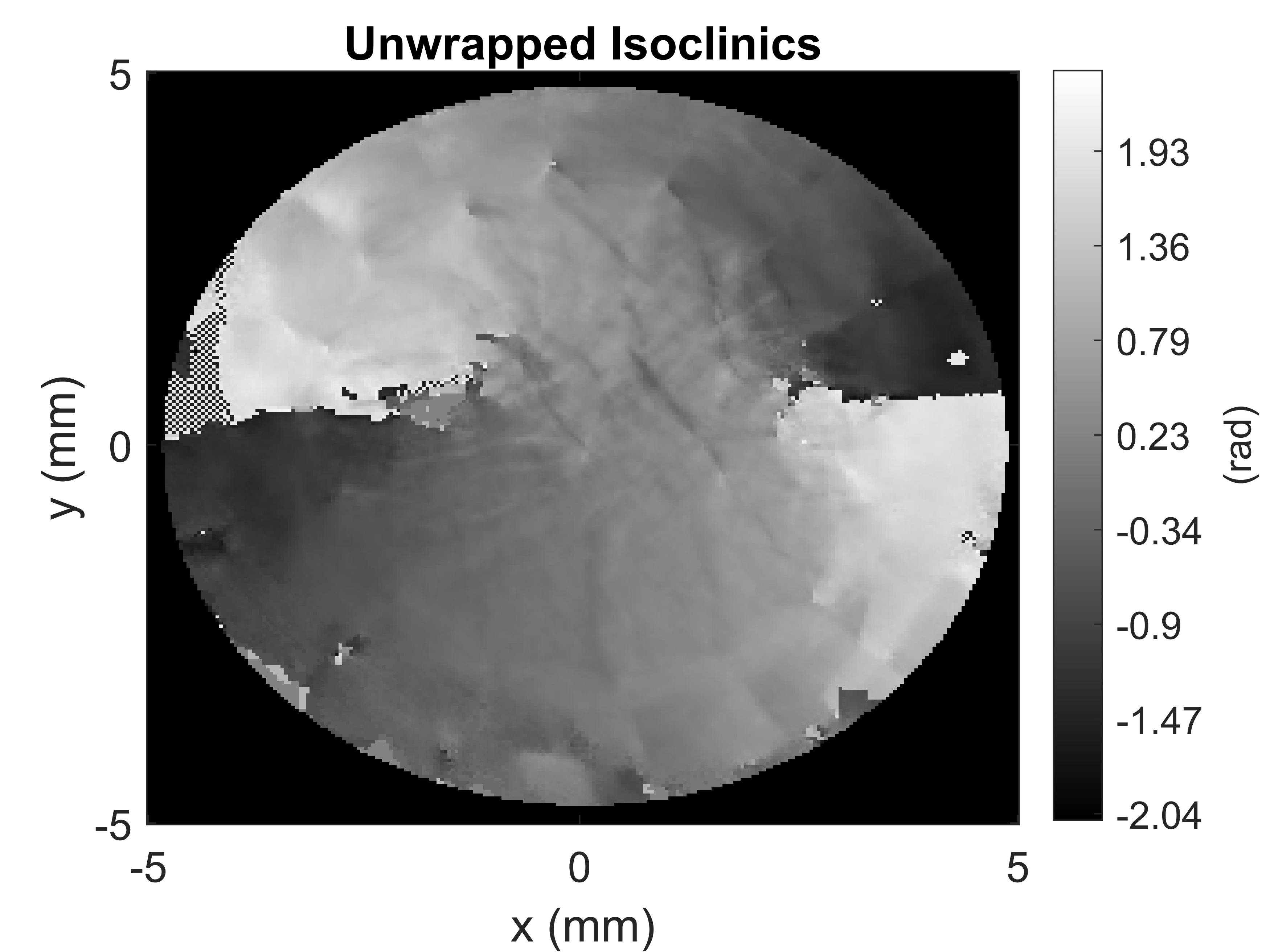

Supplement: S1 File — (ZIP) [file pone.0308204.s001.zip › S1 file. Birefringence Images/A-PK/0 degee/2349OD/unwappedISO.tif]

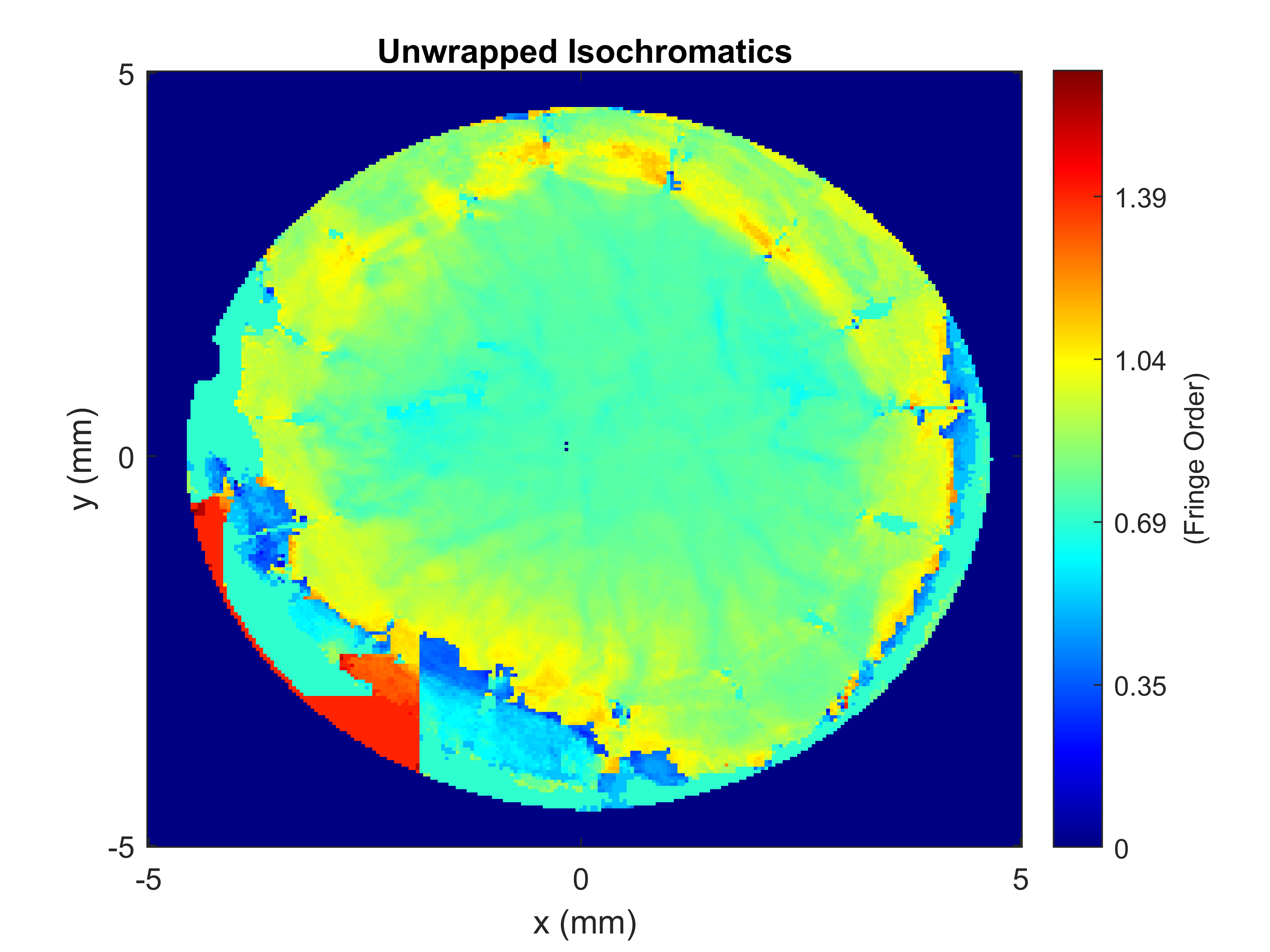

Supplement: S1 File — (ZIP) [file pone.0308204.s001.zip › S1 file. Birefringence Images/A-PK/0 degee/2349OD/unwappedISOCH.tif]

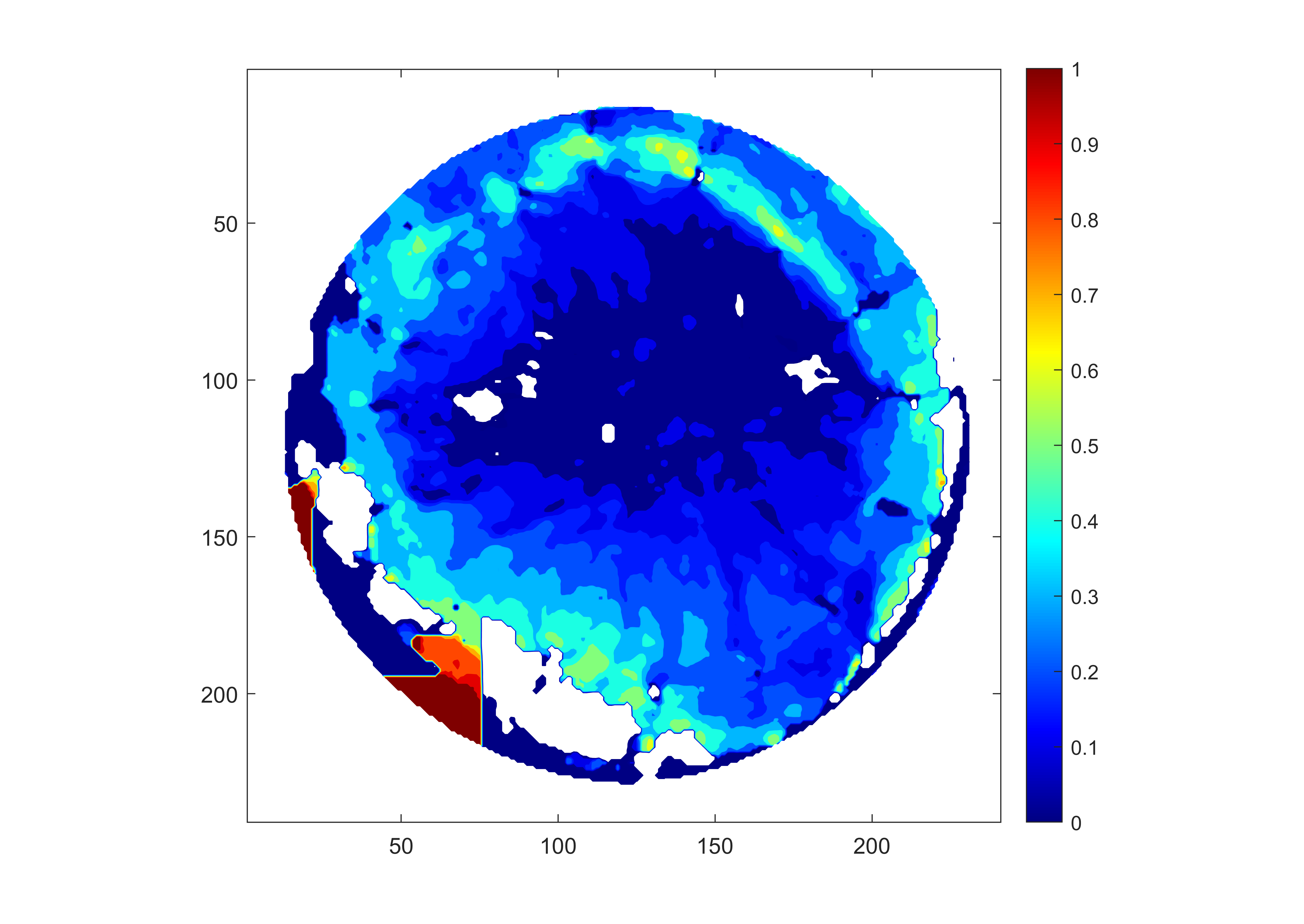

Supplement: S1 File — (ZIP) [file pone.0308204.s001.zip › S1 file. Birefringence Images/A-PK/0 degee/2349OD/unwappedISOCHcolofilled.tif]

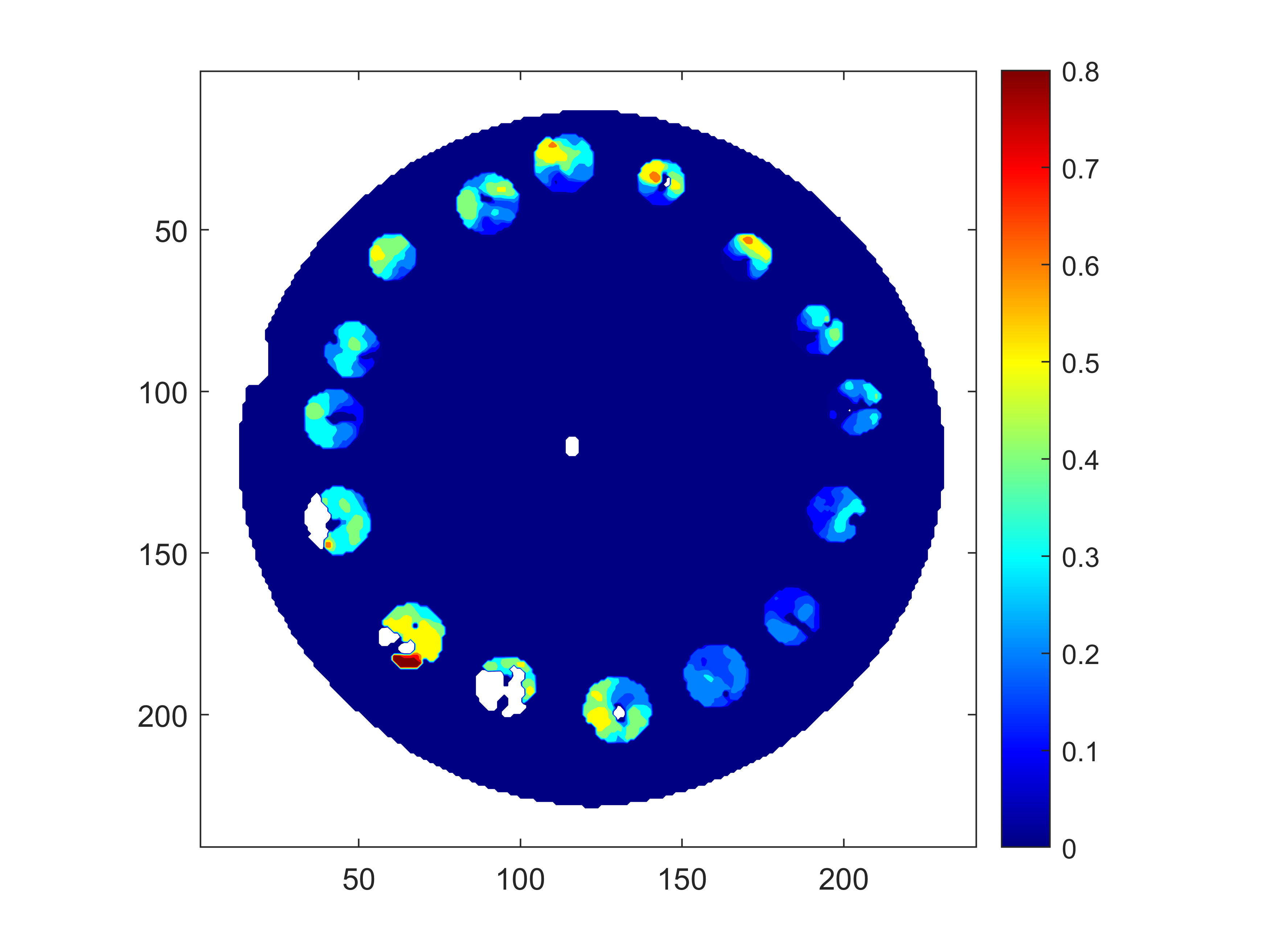

Supplement: S1 File — (ZIP) [file pone.0308204.s001.zip › S1 file. Birefringence Images/A-PK/0 degee/2349OD/unwappedISOCHcolofilledmasked.tif]

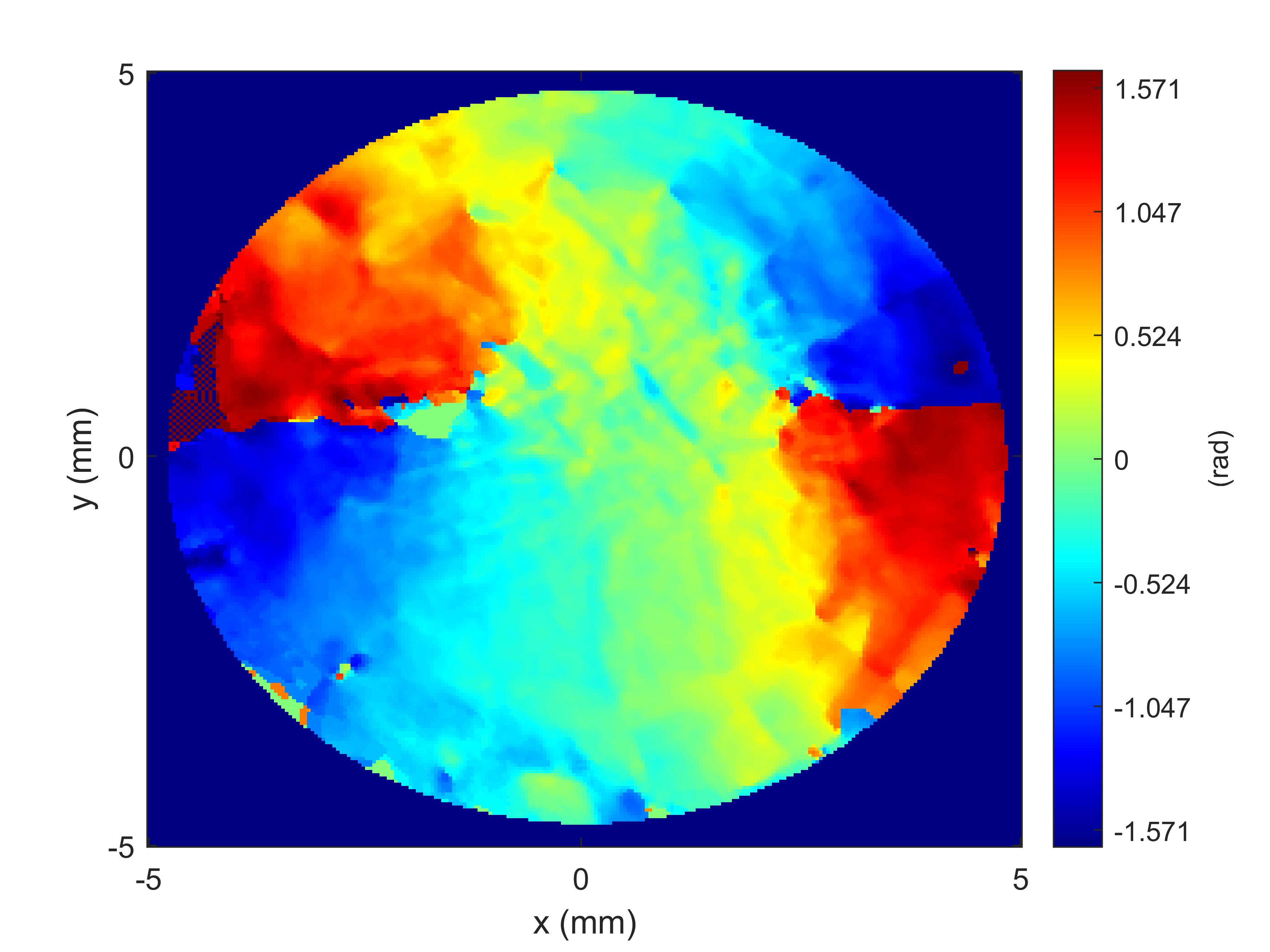

Supplement: S1 File — (ZIP) [file pone.0308204.s001.zip › S1 file. Birefringence Images/A-PK/0 degee/2349OD/unwappedISOcolo.tif]

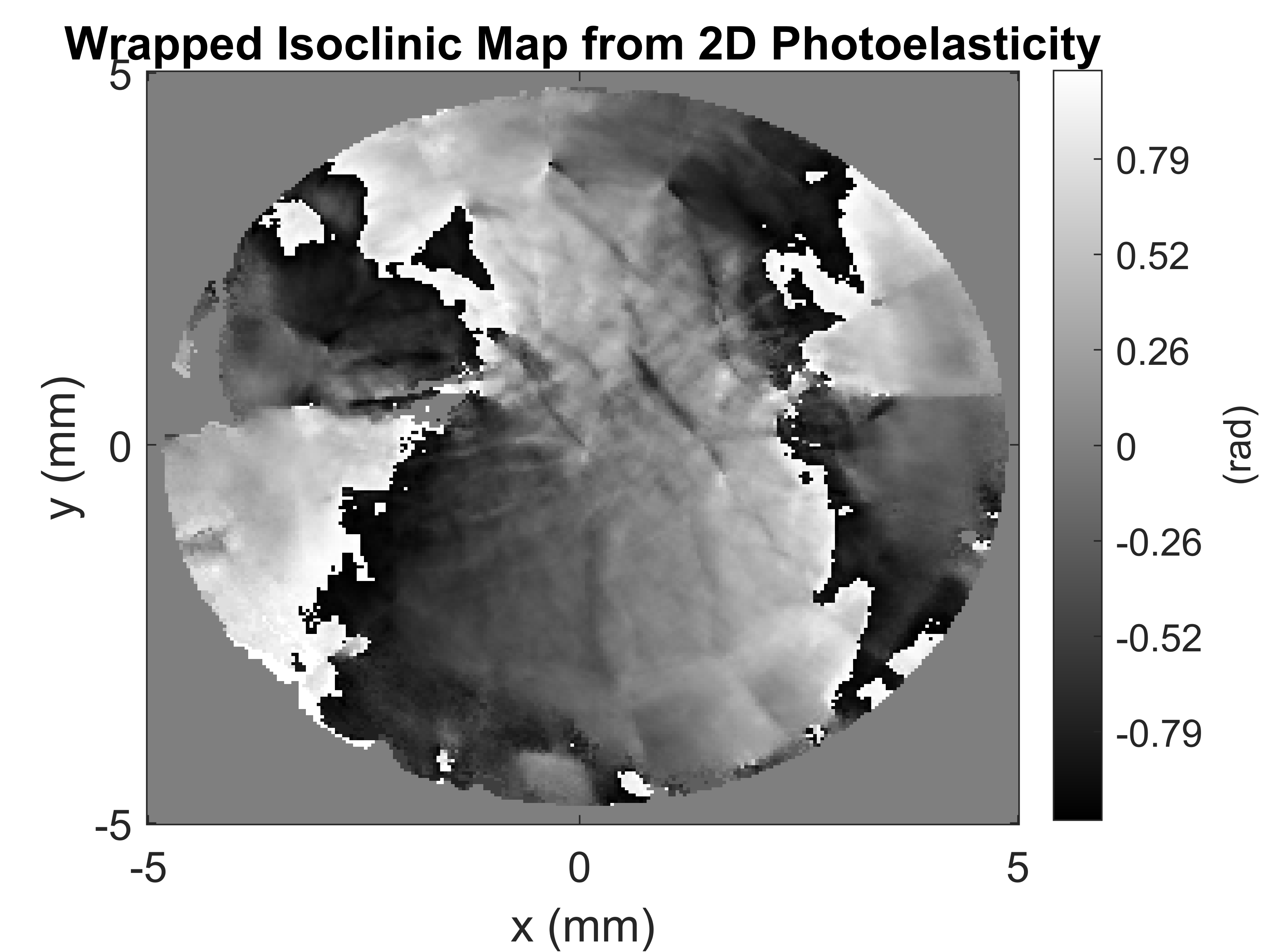

Supplement: S1 File — (ZIP) [file pone.0308204.s001.zip › S1 file. Birefringence Images/A-PK/0 degee/2349OD/wappedISOCH.tif]

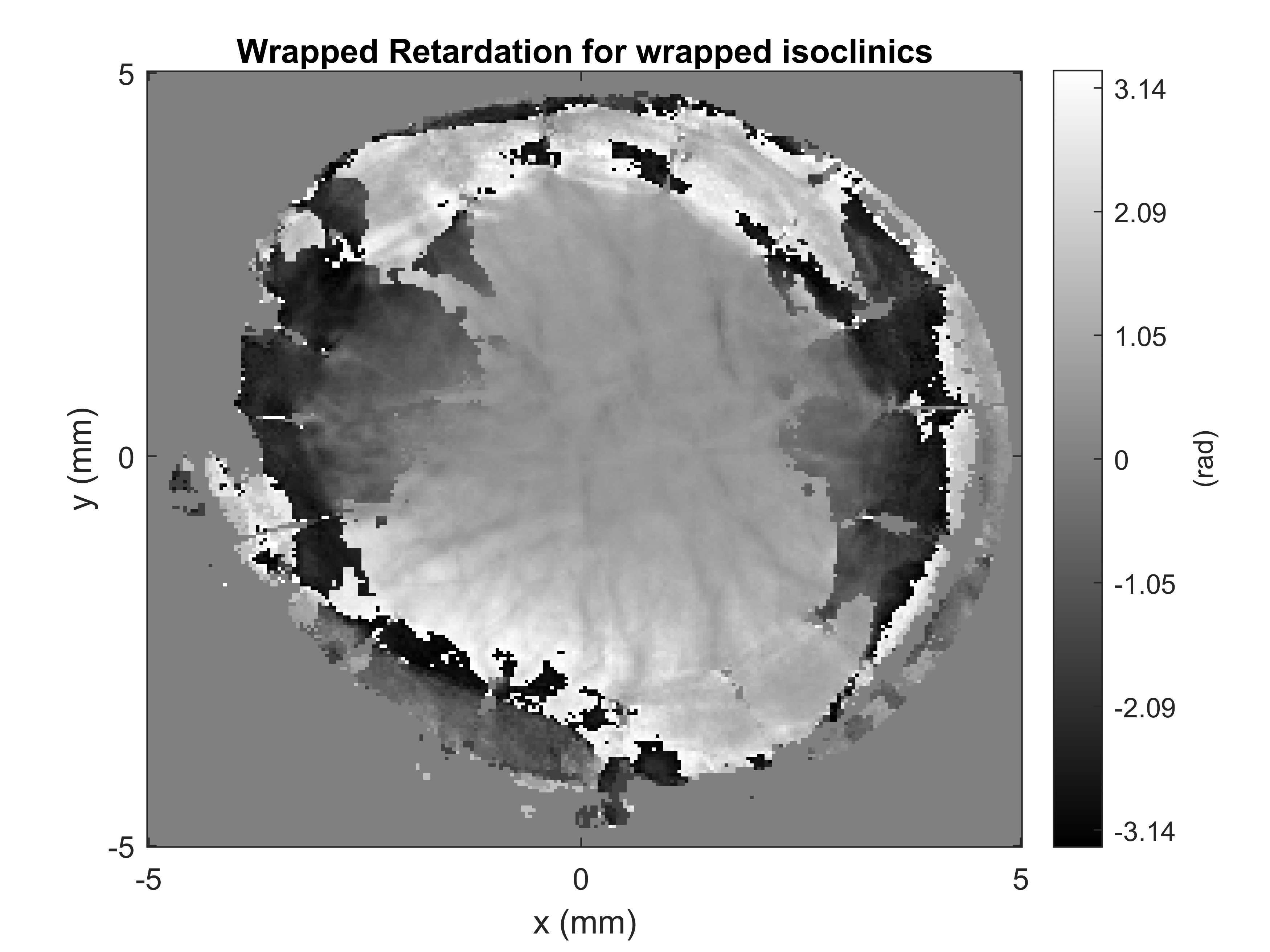

Supplement: S1 File — (ZIP) [file pone.0308204.s001.zip › S1 file. Birefringence Images/A-PK/0 degee/2349OD/wappedISOCHwappedISO.tif]

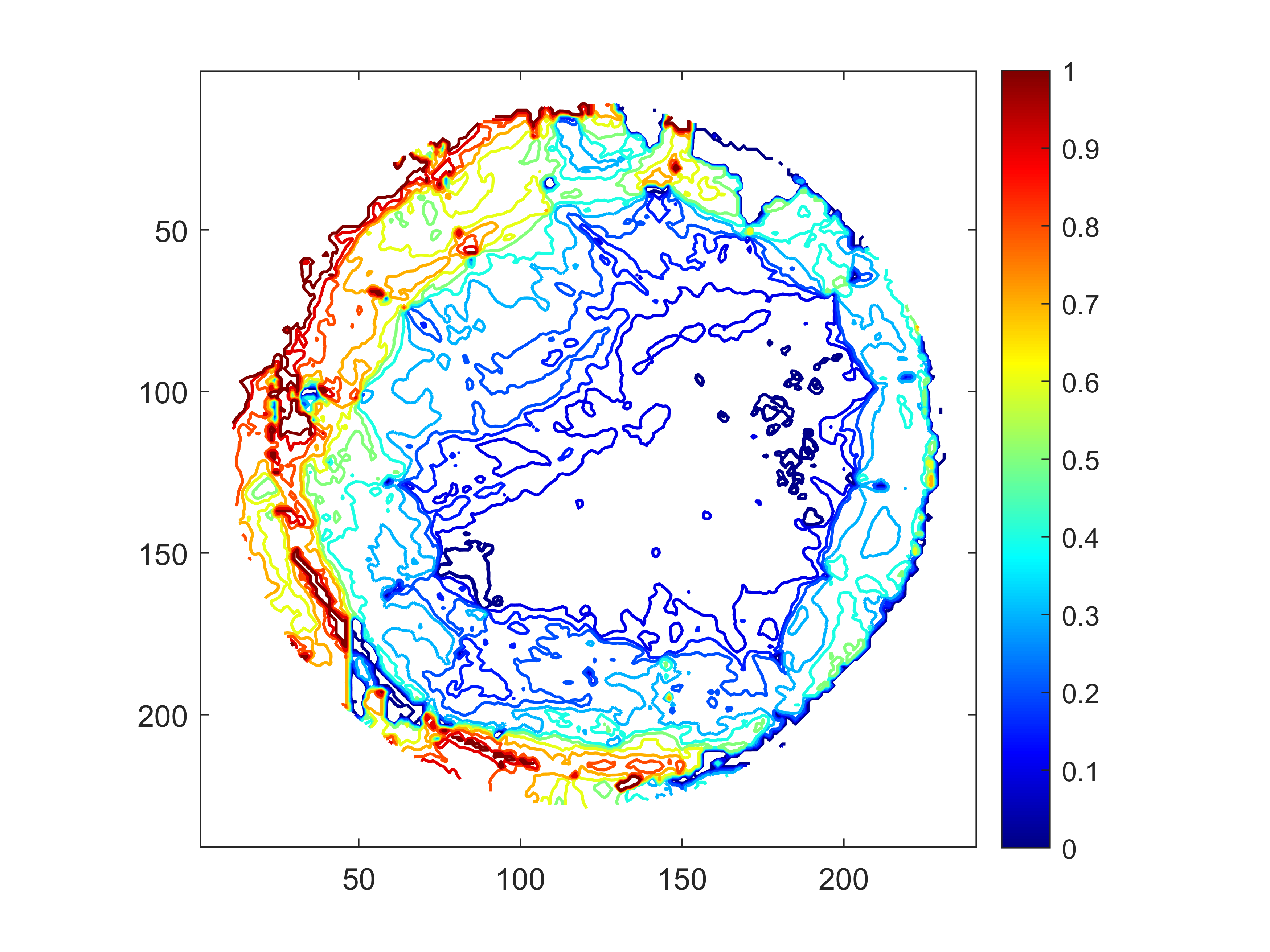

Supplement: S1 File — (ZIP) [file pone.0308204.s001.zip › S1 file. Birefringence Images/A-PK/30 degee/ISOCHconours.tif]

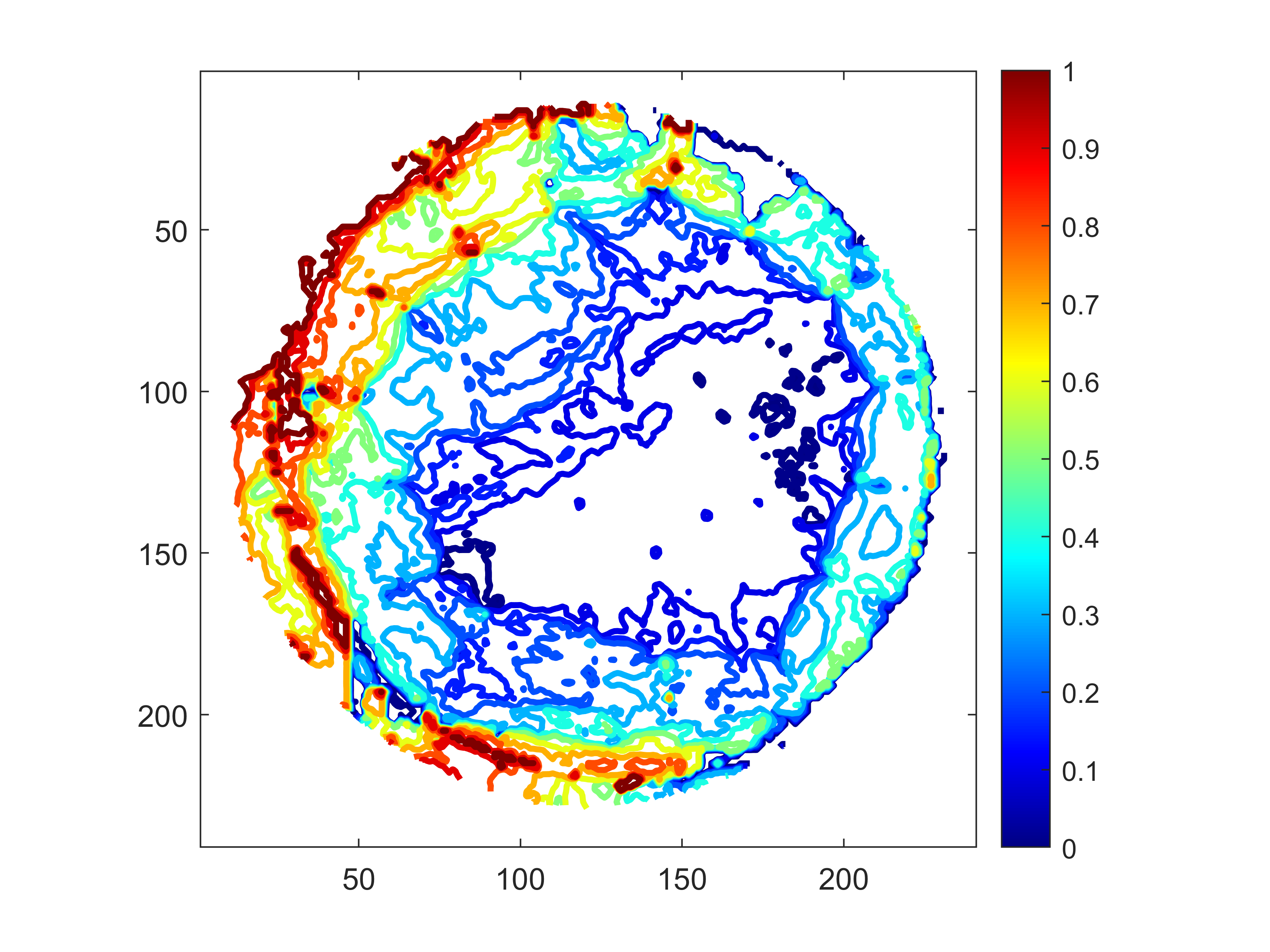

Supplement: S1 File — (ZIP) [file pone.0308204.s001.zip › S1 file. Birefringence Images/A-PK/30 degee/ISOCHconours2.tif]

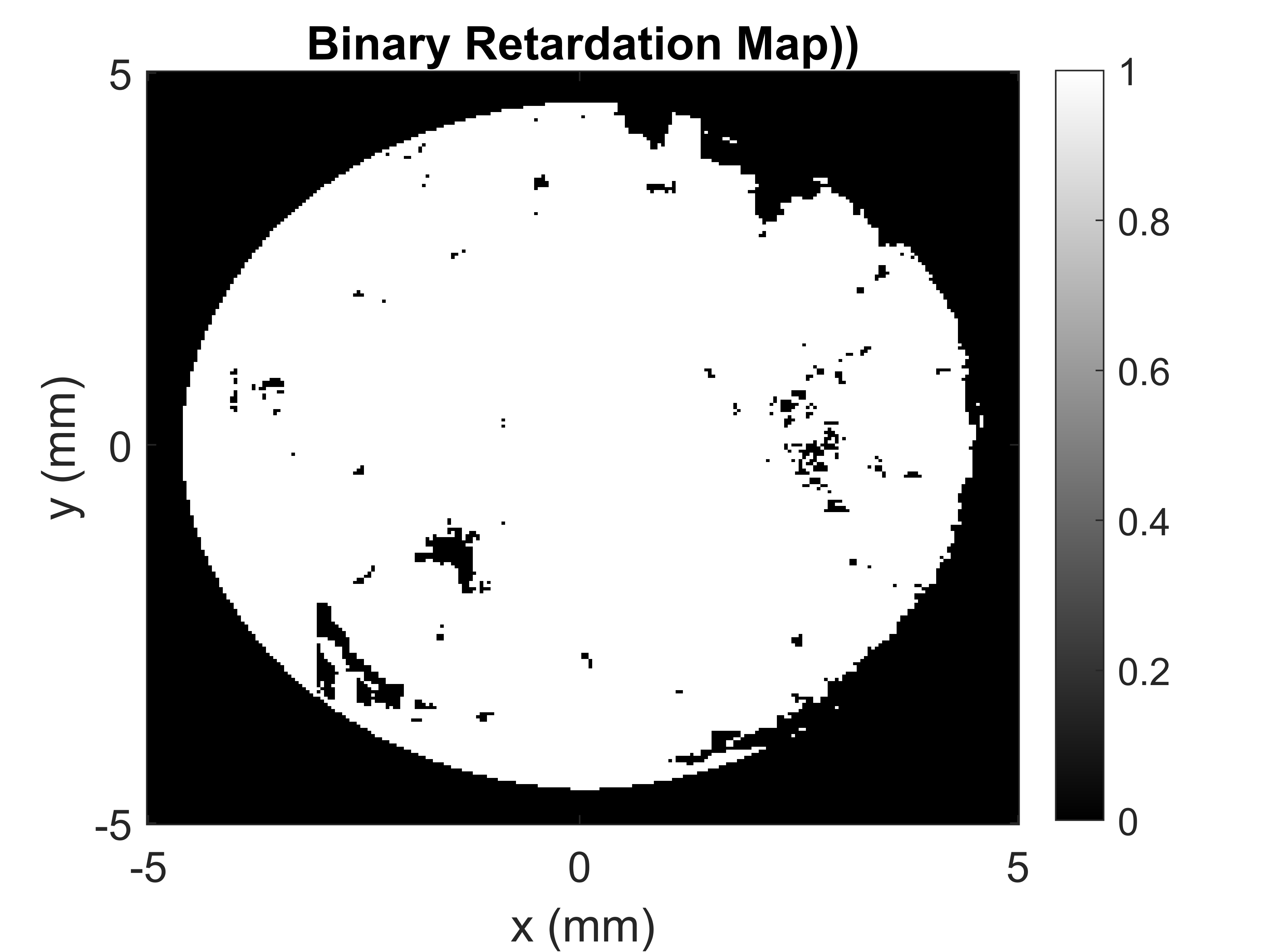

Supplement: S1 File — (ZIP) [file pone.0308204.s001.zip › S1 file. Birefringence Images/A-PK/30 degee/isoopic poins.tif]

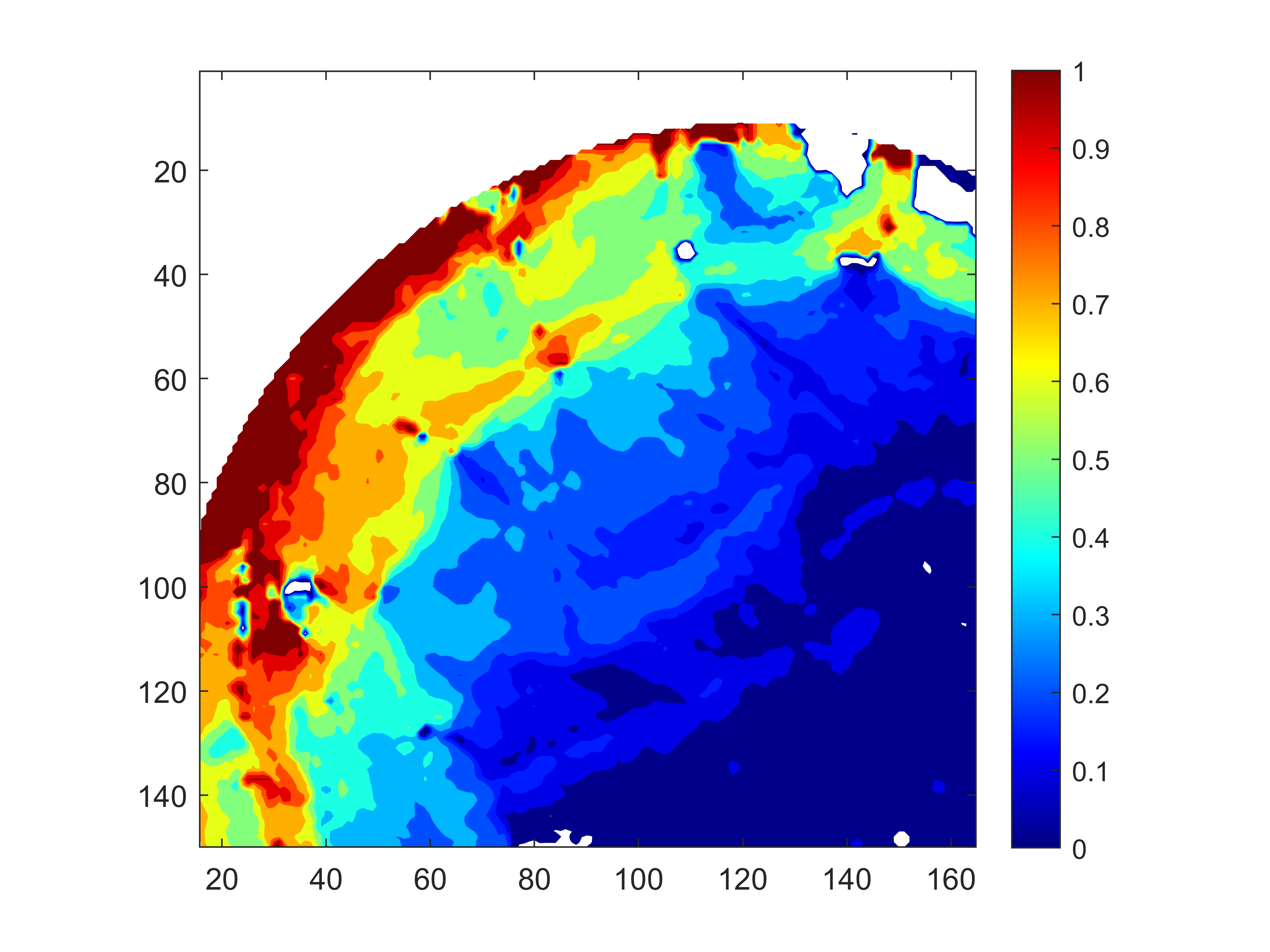

Supplement: S1 File — (ZIP) [file pone.0308204.s001.zip › S1 file. Birefringence Images/A-PK/30 degee/isozoomed.tif]

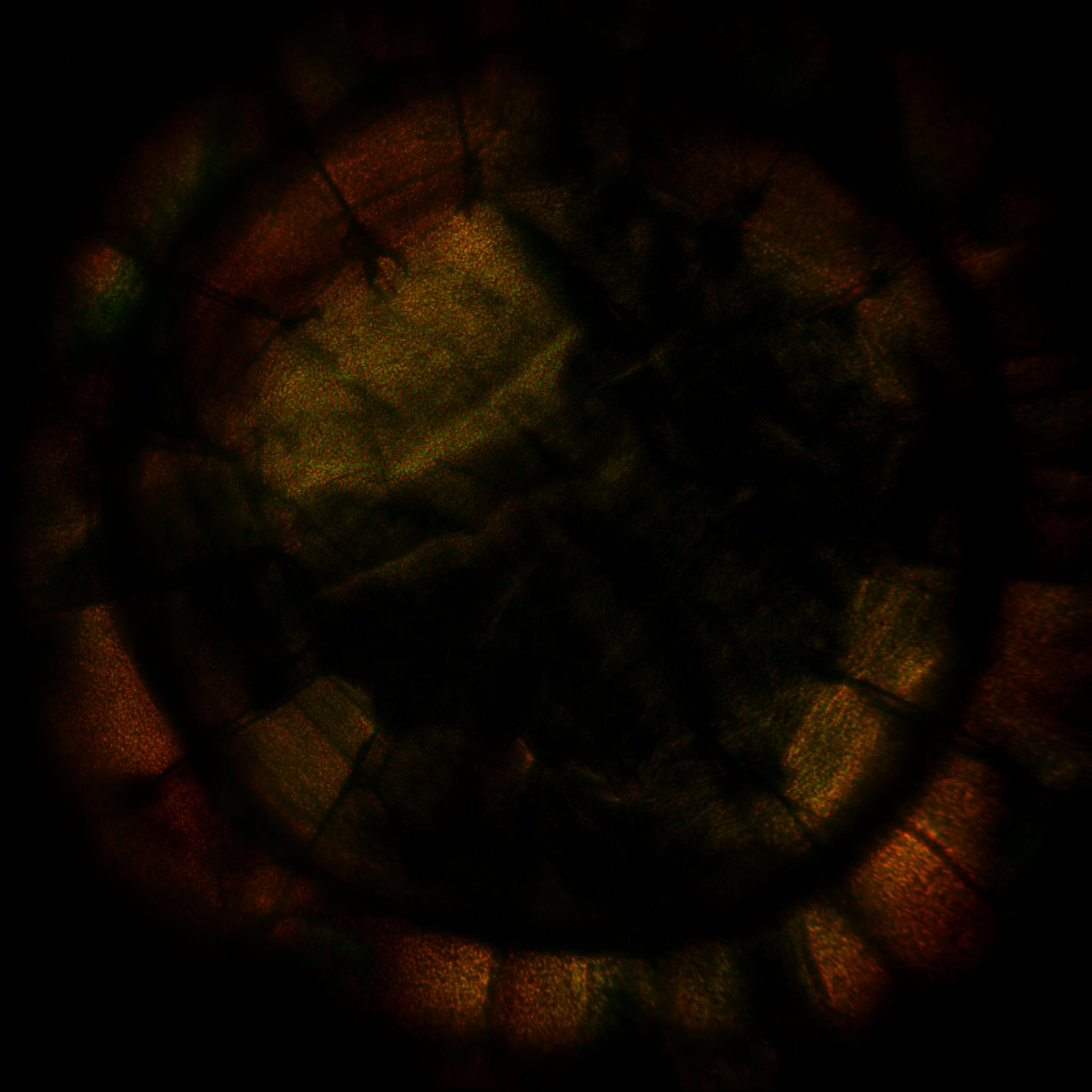

Supplement: S1 File — (ZIP) [file pone.0308204.s001.zip › S1 file. Birefringence Images/A-PK/30 degee/IW1.jpg]

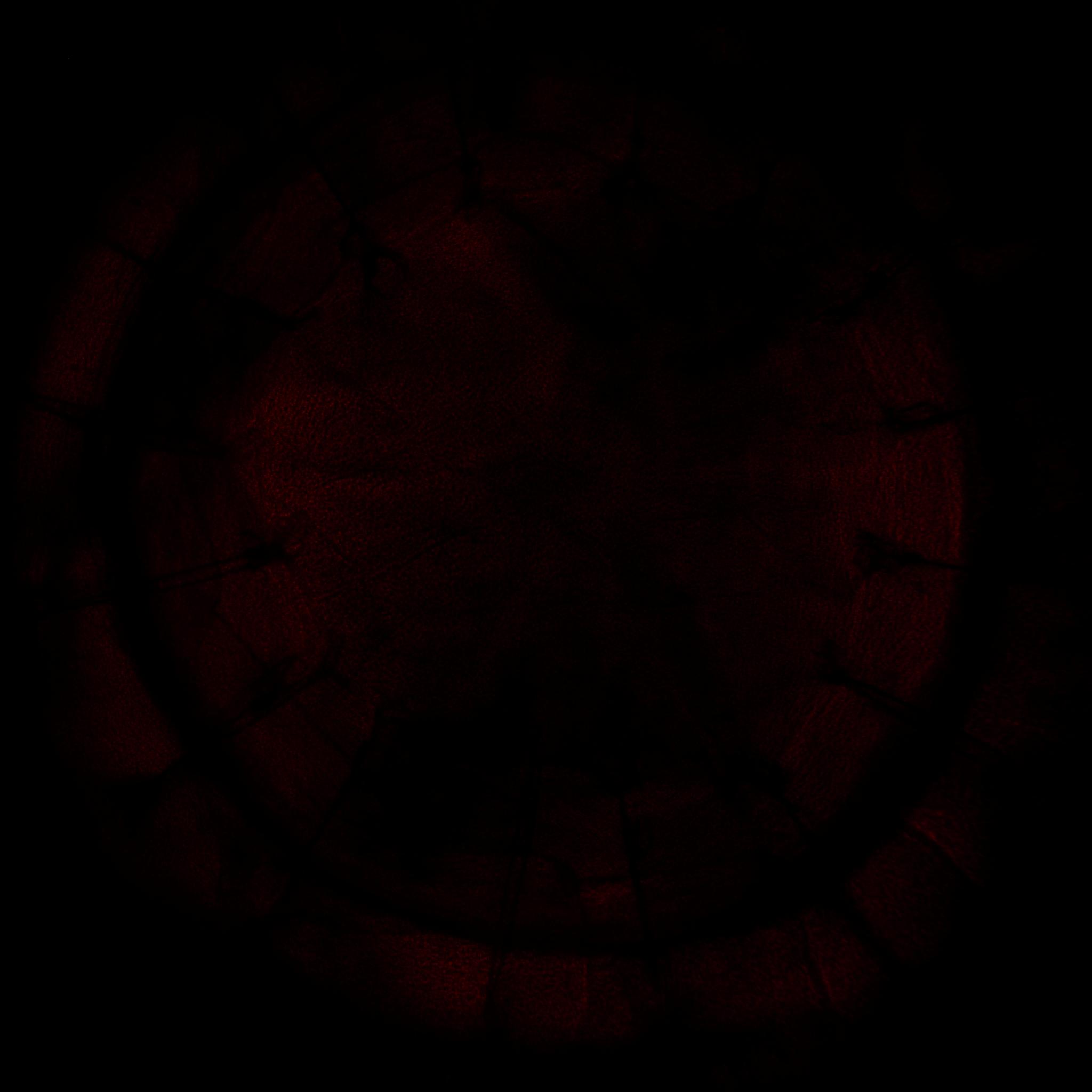

Supplement: S1 File — (ZIP) [file pone.0308204.s001.zip › S1 file. Birefringence Images/A-PK/30 degee/IW10.jpg]

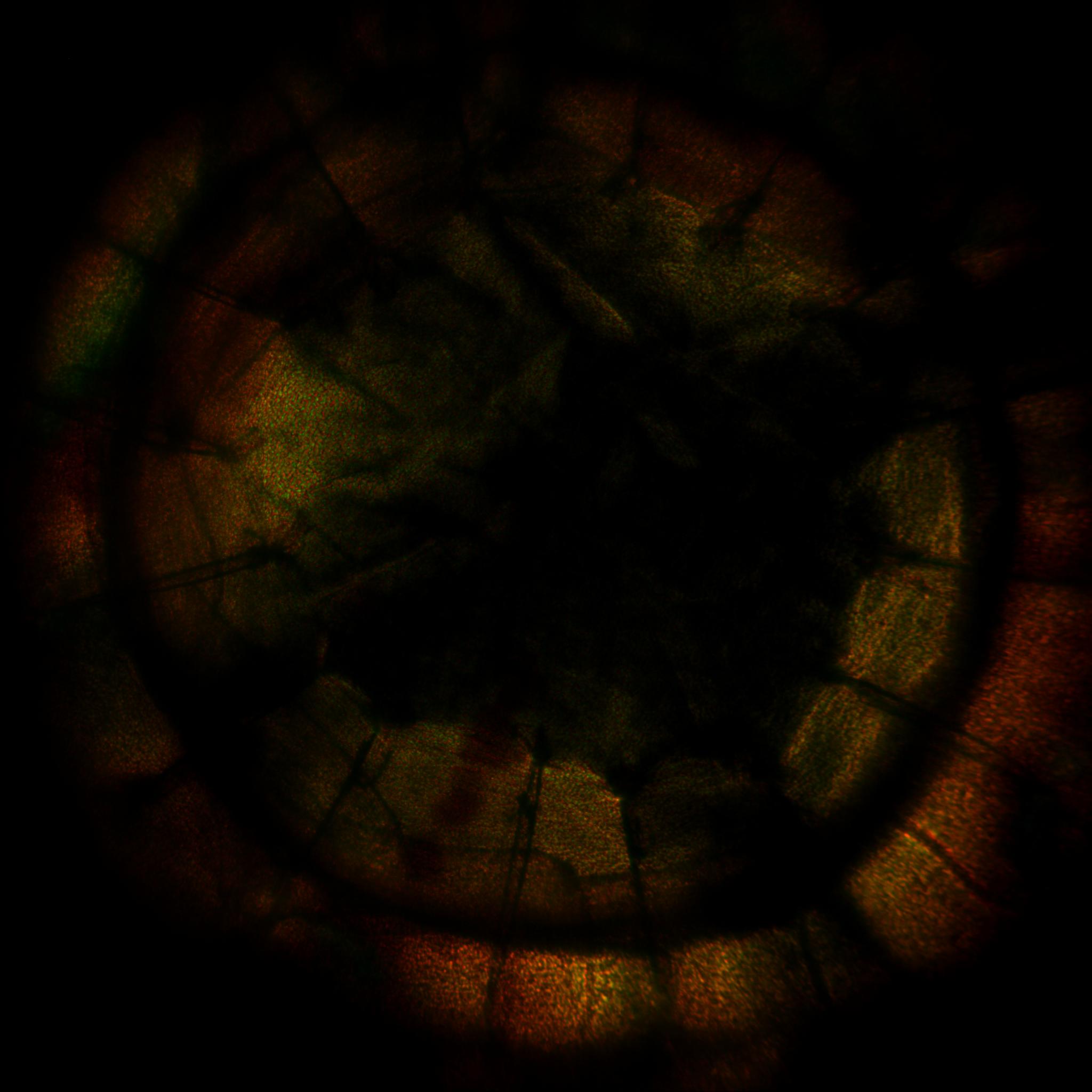

Supplement: S1 File — (ZIP) [file pone.0308204.s001.zip › S1 file. Birefringence Images/A-PK/30 degee/IW2.jpg]

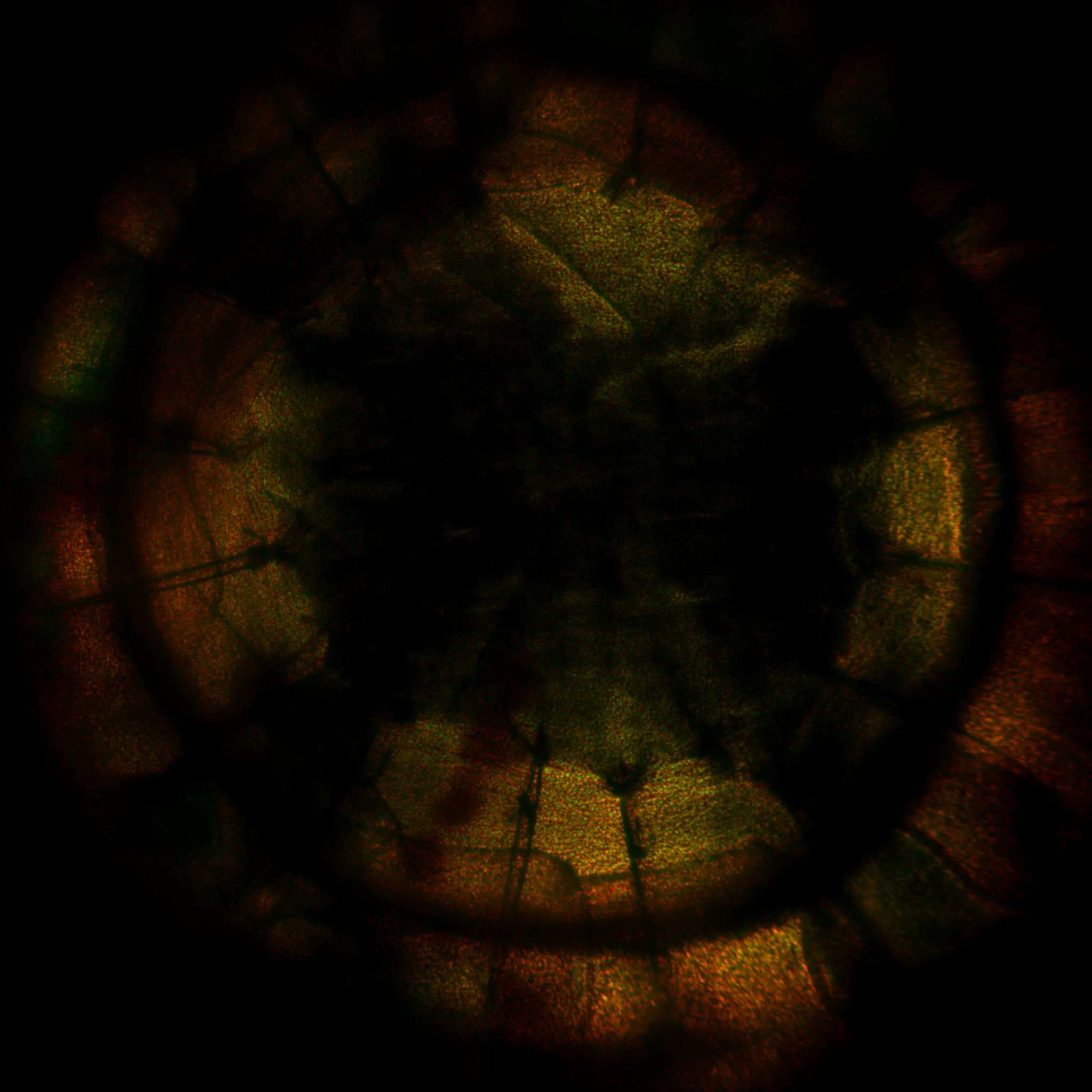

Supplement: S1 File — (ZIP) [file pone.0308204.s001.zip › S1 file. Birefringence Images/A-PK/30 degee/IW3.jpg]

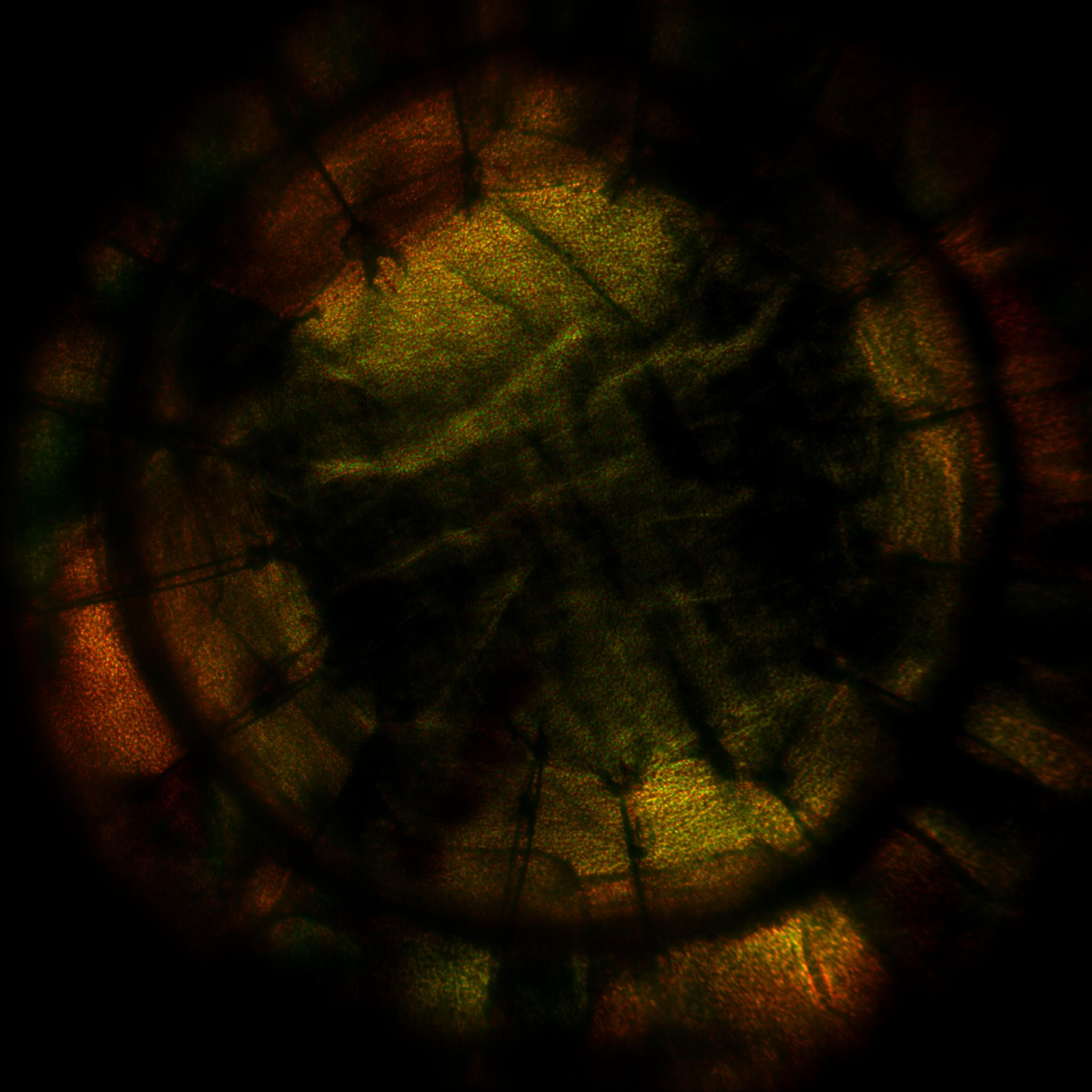

Supplement: S1 File — (ZIP) [file pone.0308204.s001.zip › S1 file. Birefringence Images/A-PK/30 degee/IW4.jpg]

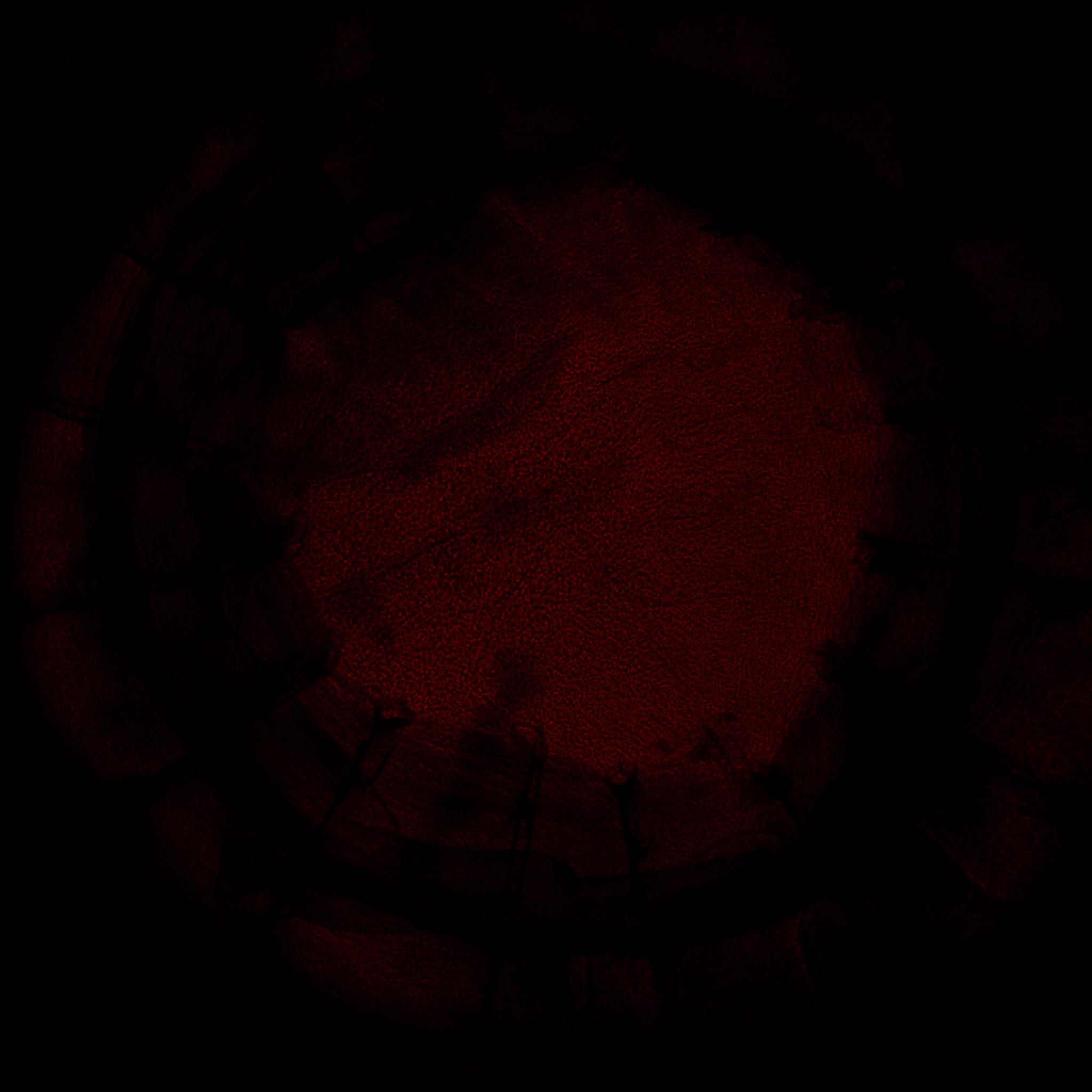

Supplement: S1 File — (ZIP) [file pone.0308204.s001.zip › S1 file. Birefringence Images/A-PK/30 degee/IW5.jpg]

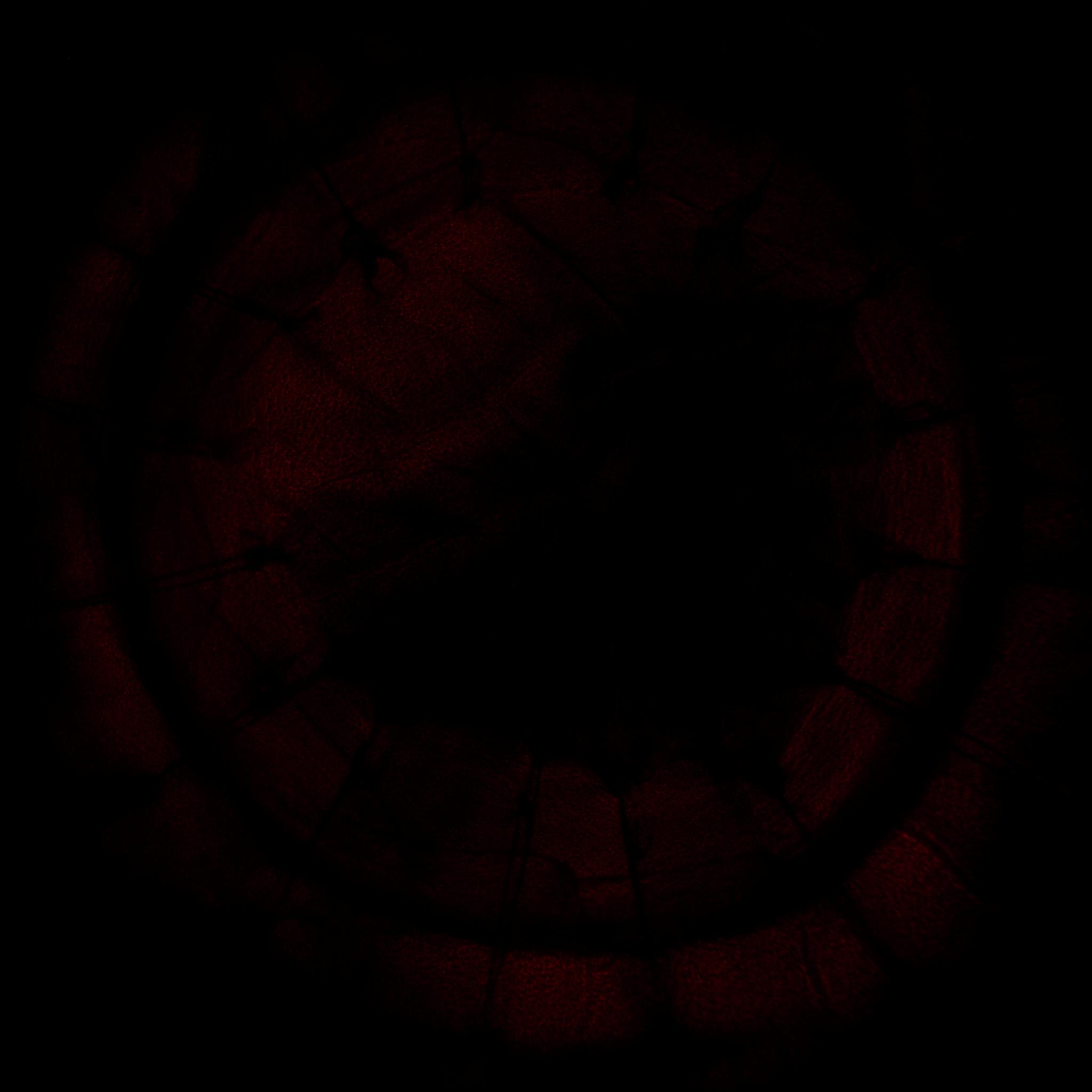

Supplement: S1 File — (ZIP) [file pone.0308204.s001.zip › S1 file. Birefringence Images/A-PK/30 degee/IW6.jpg]

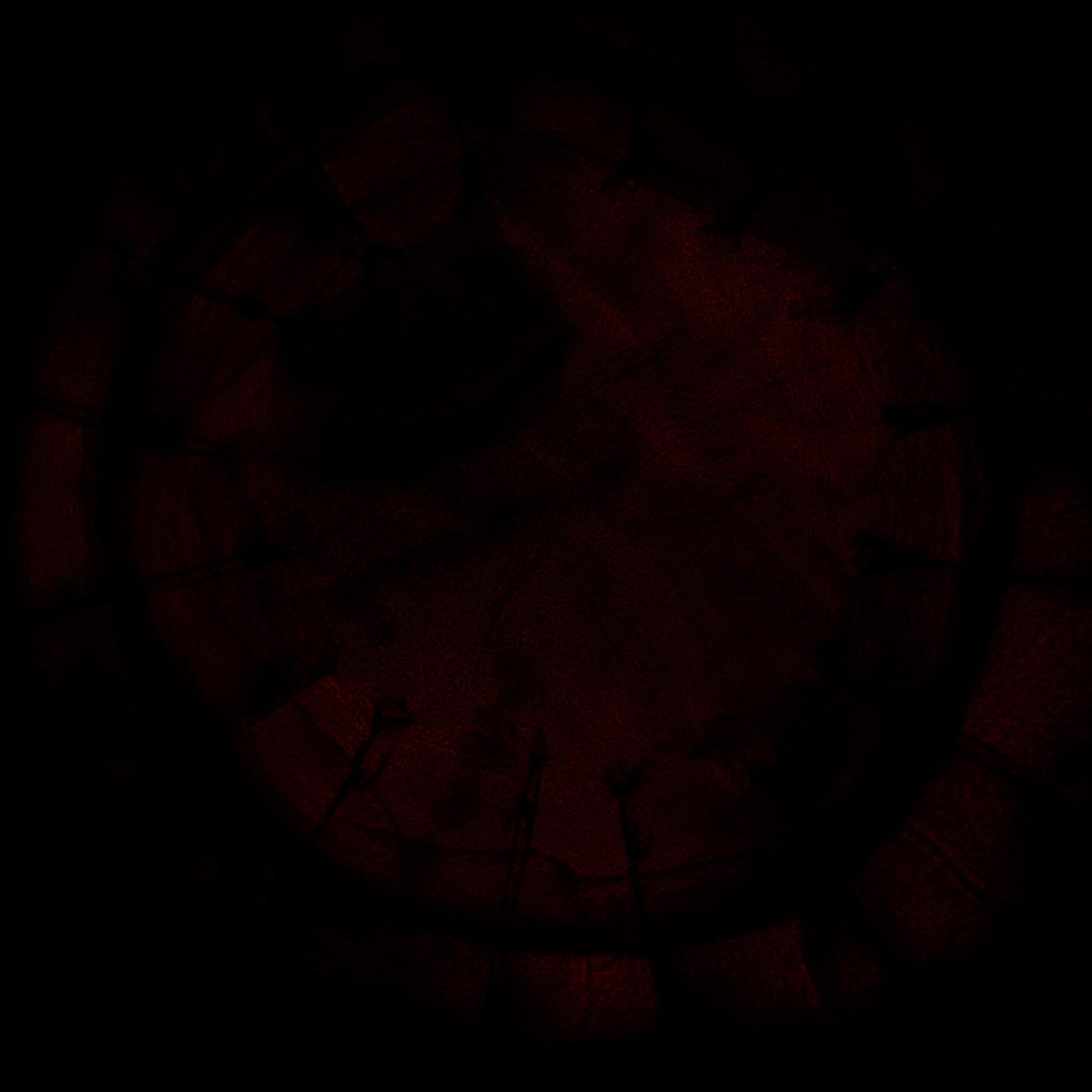

Supplement: S1 File — (ZIP) [file pone.0308204.s001.zip › S1 file. Birefringence Images/A-PK/30 degee/IW7.jpg]

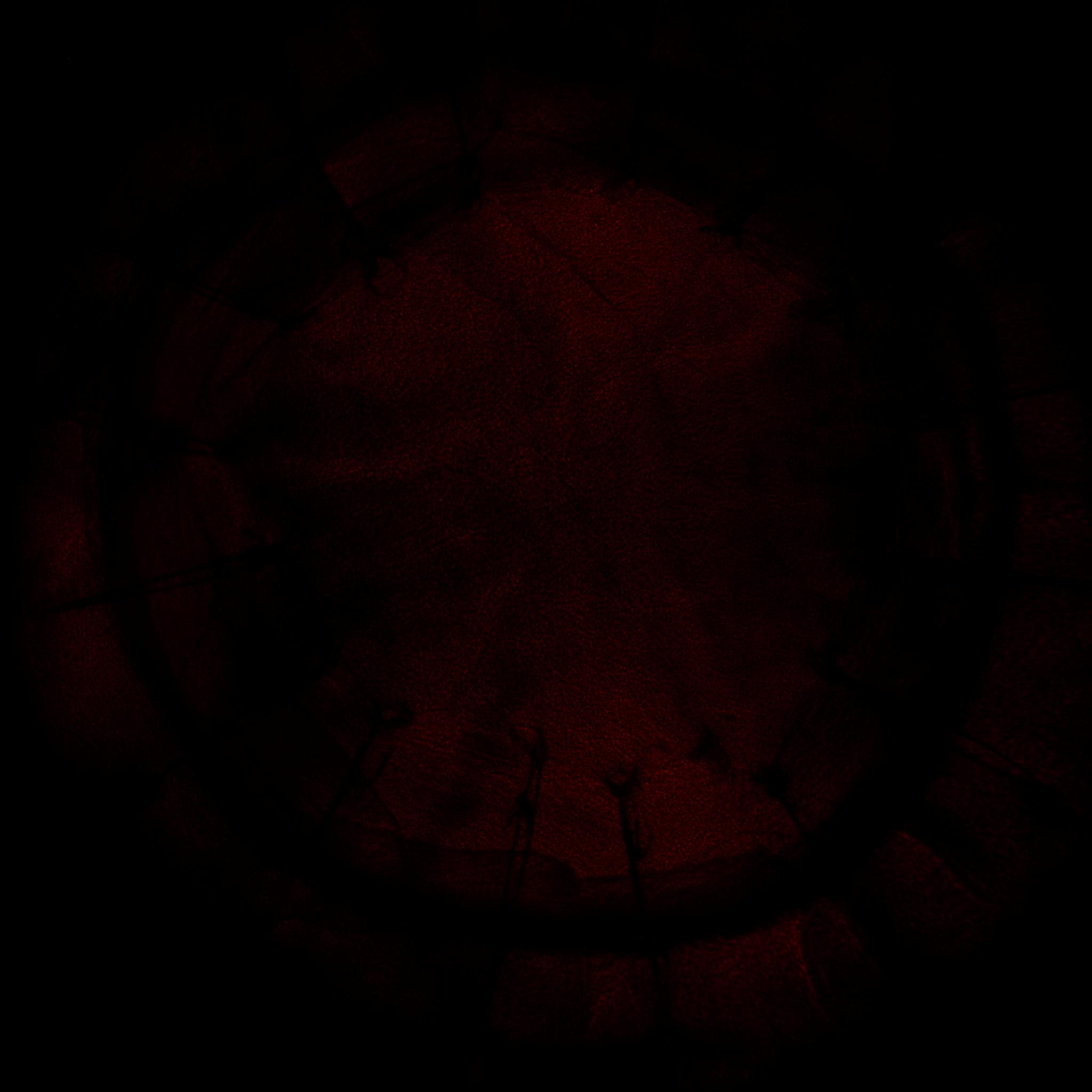

Supplement: S1 File — (ZIP) [file pone.0308204.s001.zip › S1 file. Birefringence Images/A-PK/30 degee/IW8.jpg]

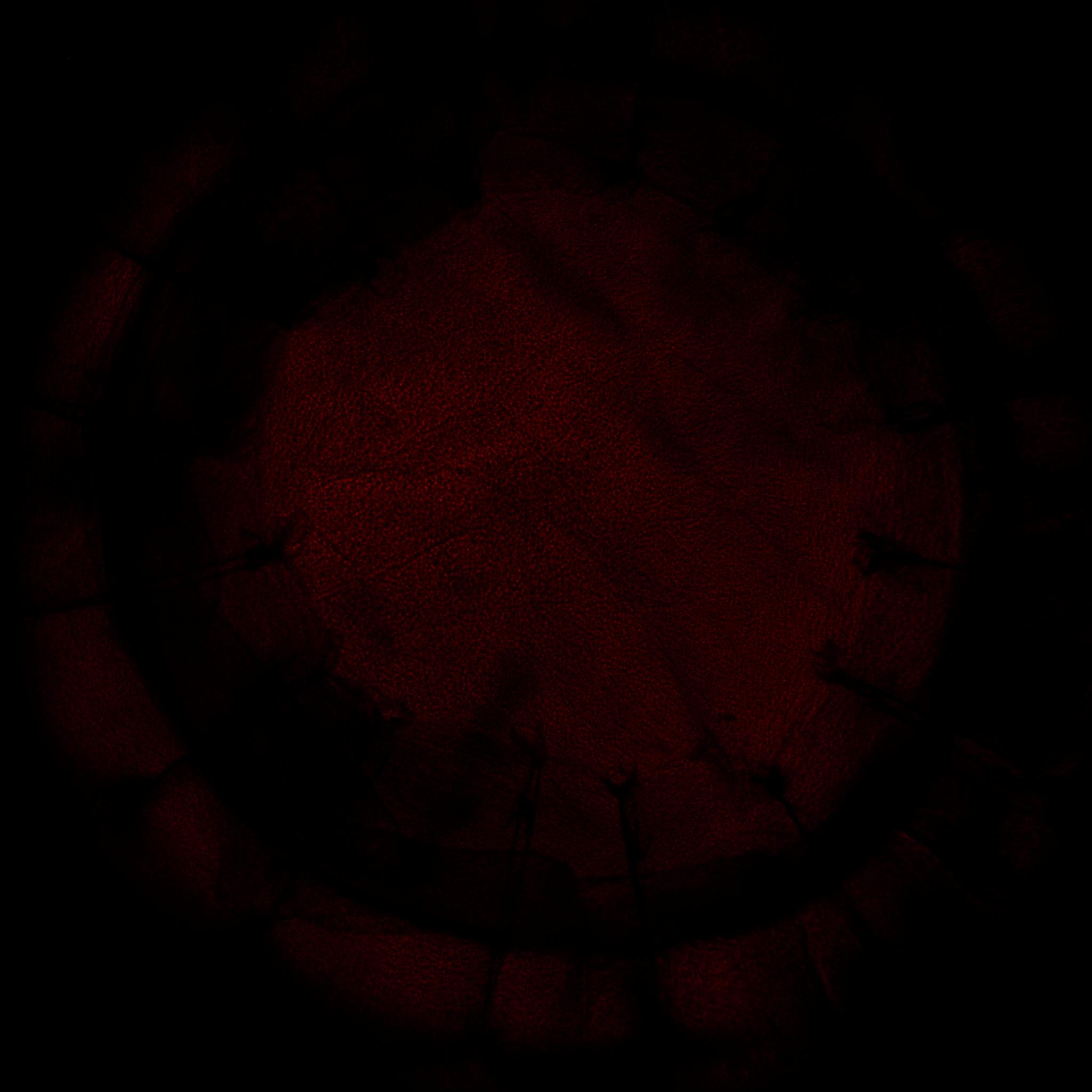

Supplement: S1 File — (ZIP) [file pone.0308204.s001.zip › S1 file. Birefringence Images/A-PK/30 degee/IW9.jpg]

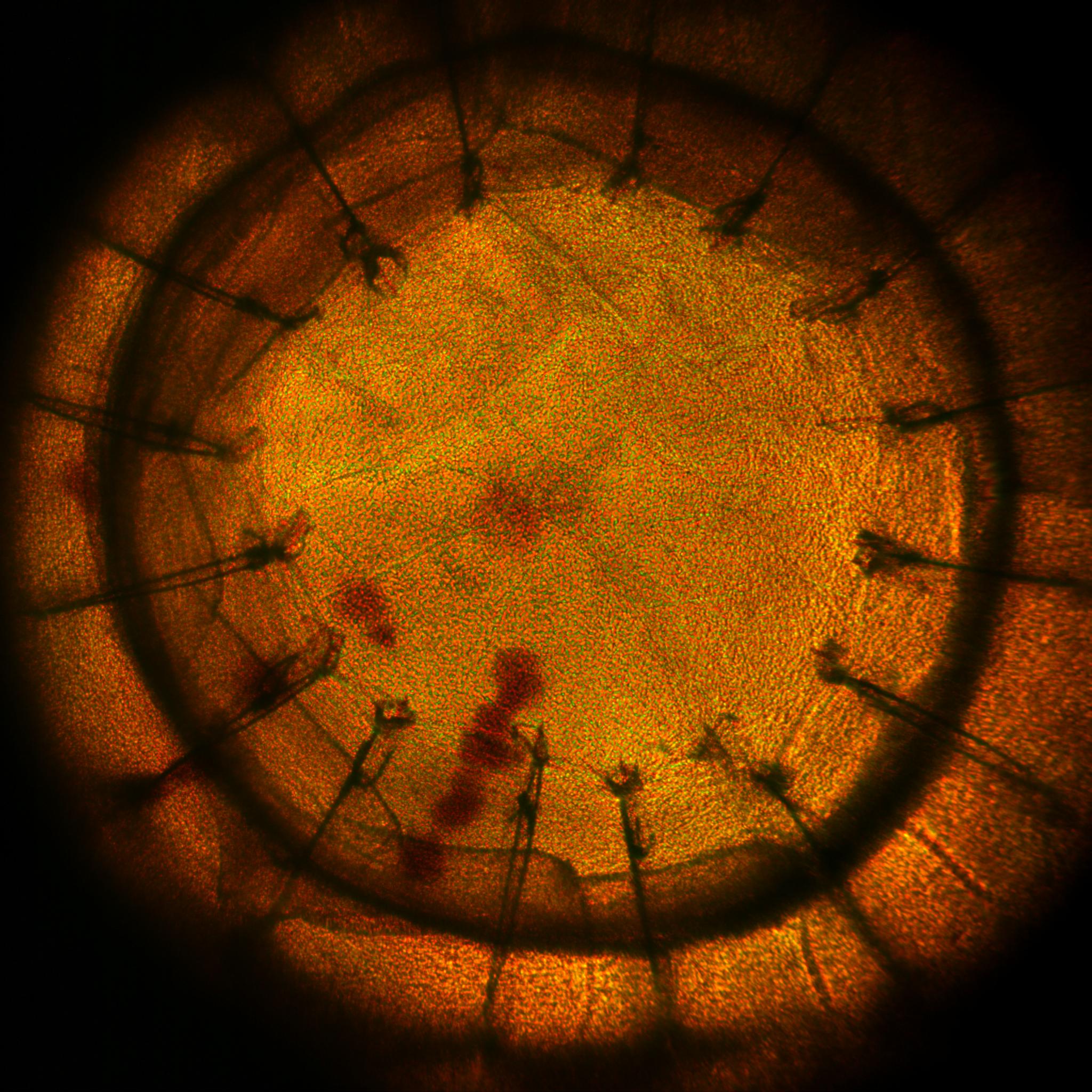

Supplement: S1 File — (ZIP) [file pone.0308204.s001.zip › S1 file. Birefringence Images/A-PK/30 degee/Suures.jpg]

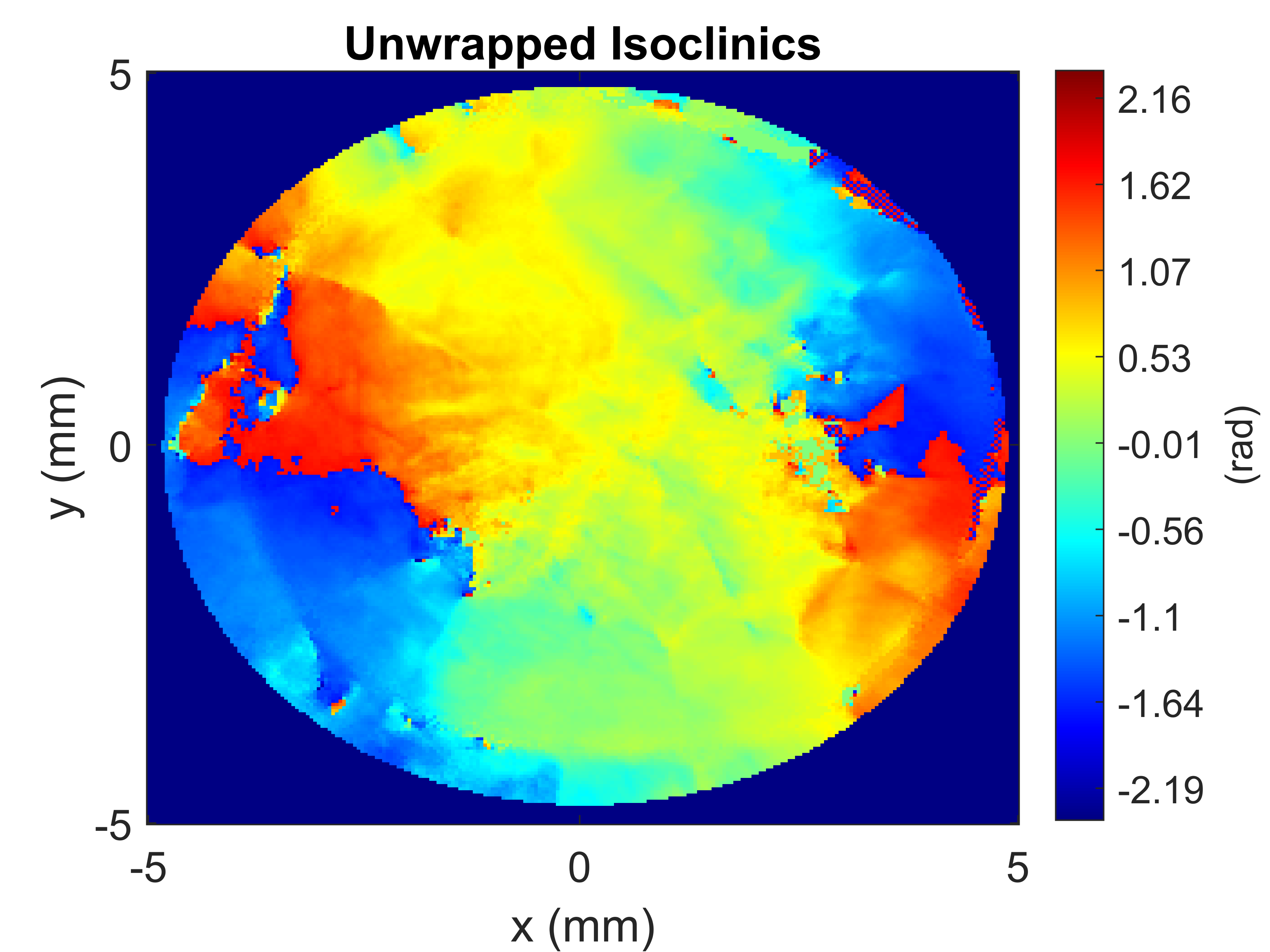

Supplement: S1 File — (ZIP) [file pone.0308204.s001.zip › S1 file. Birefringence Images/A-PK/30 degee/unwppedISOcolor.tif]

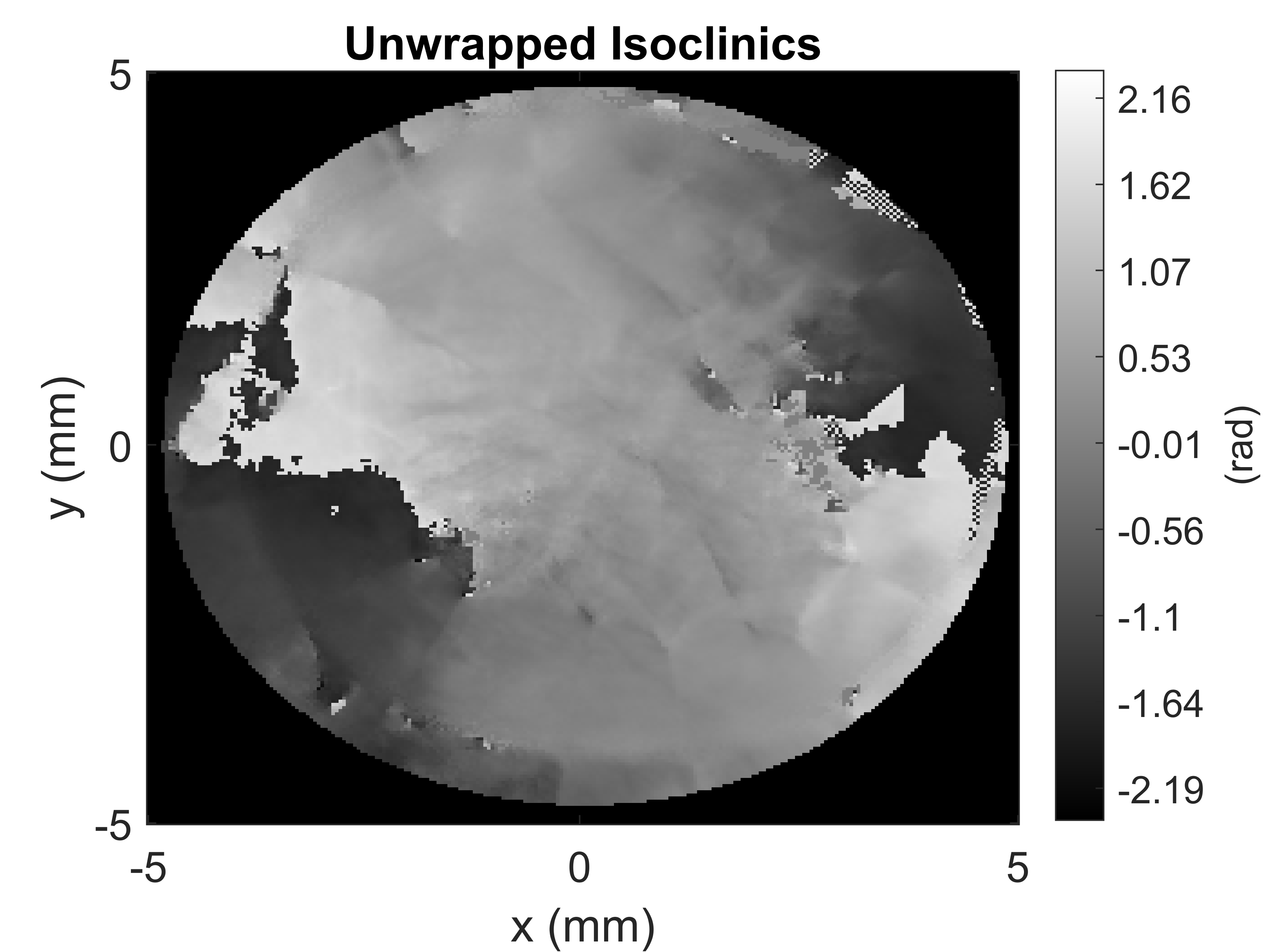

Supplement: S1 File — (ZIP) [file pone.0308204.s001.zip › S1 file. Birefringence Images/A-PK/30 degee/unwppedISOgay.tif]

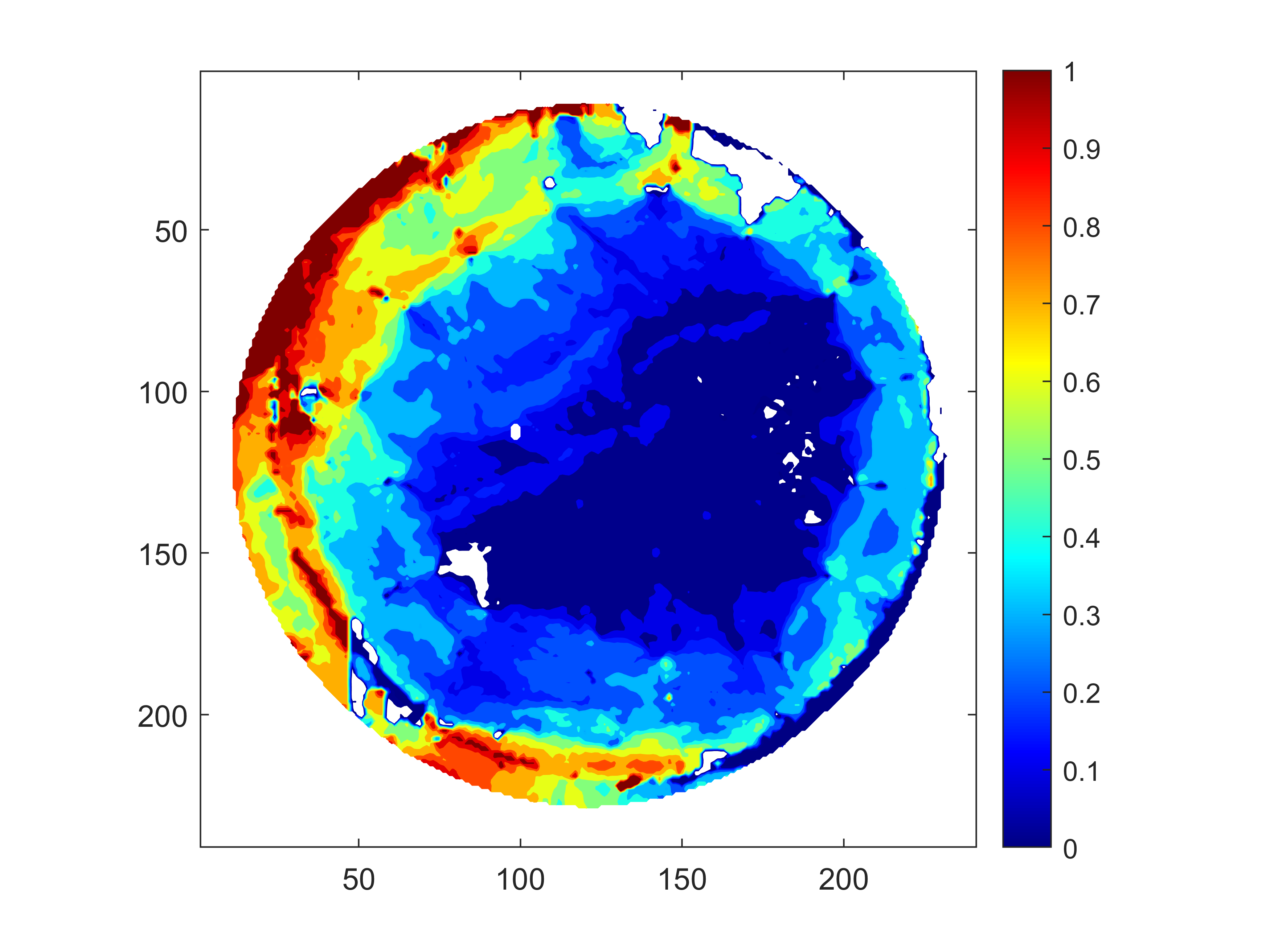

Supplement: S1 File — (ZIP) [file pone.0308204.s001.zip › S1 file. Birefringence Images/A-PK/30 degee/unwrappedISOCHcolor.tif]

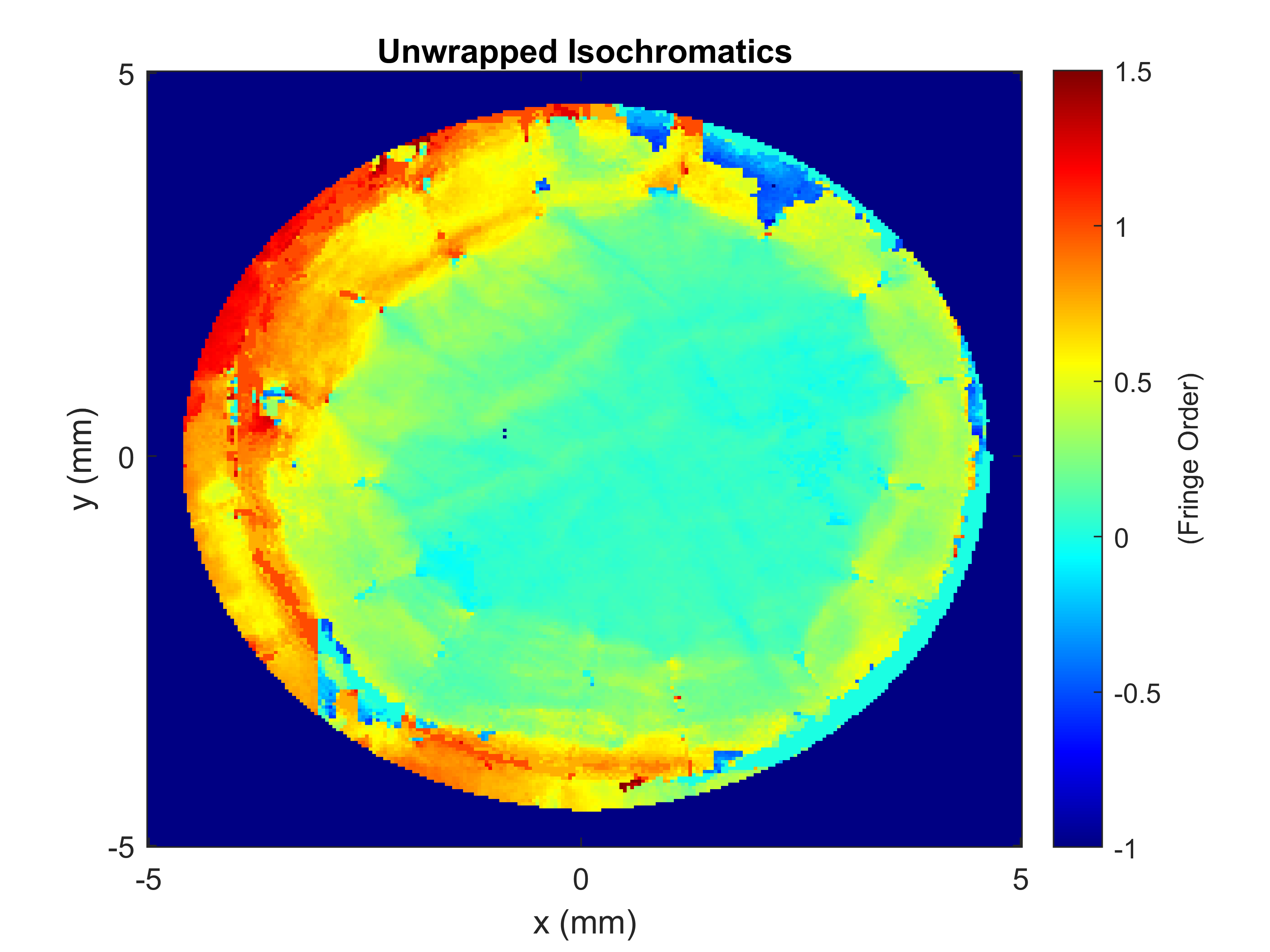

Supplement: S1 File — (ZIP) [file pone.0308204.s001.zip › S1 file. Birefringence Images/A-PK/30 degee/unwrappedISOCHcolorunfilered.tif]

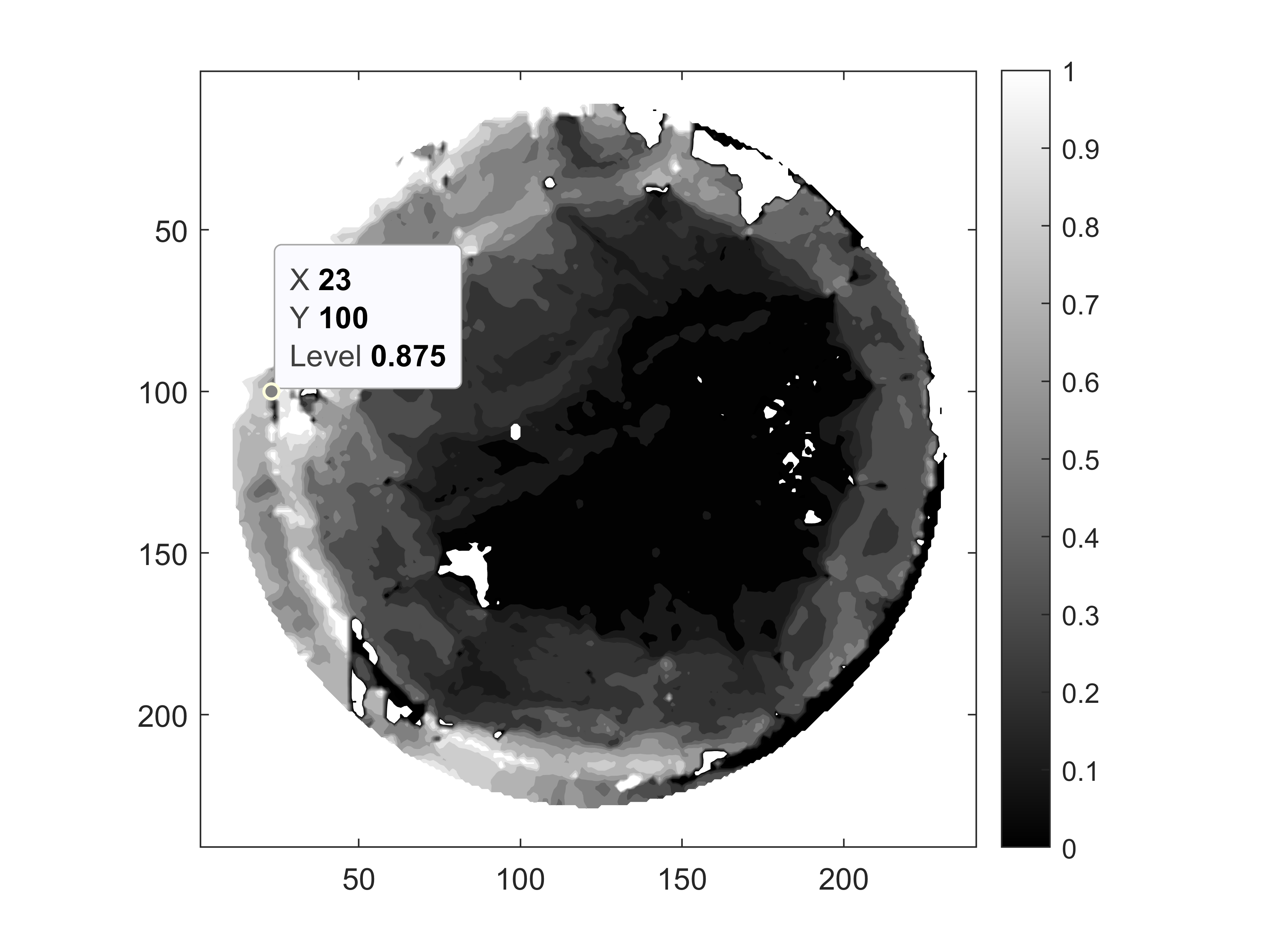

Supplement: S1 File — (ZIP) [file pone.0308204.s001.zip › S1 file. Birefringence Images/A-PK/30 degee/unwrappedISOCHgray.tif]

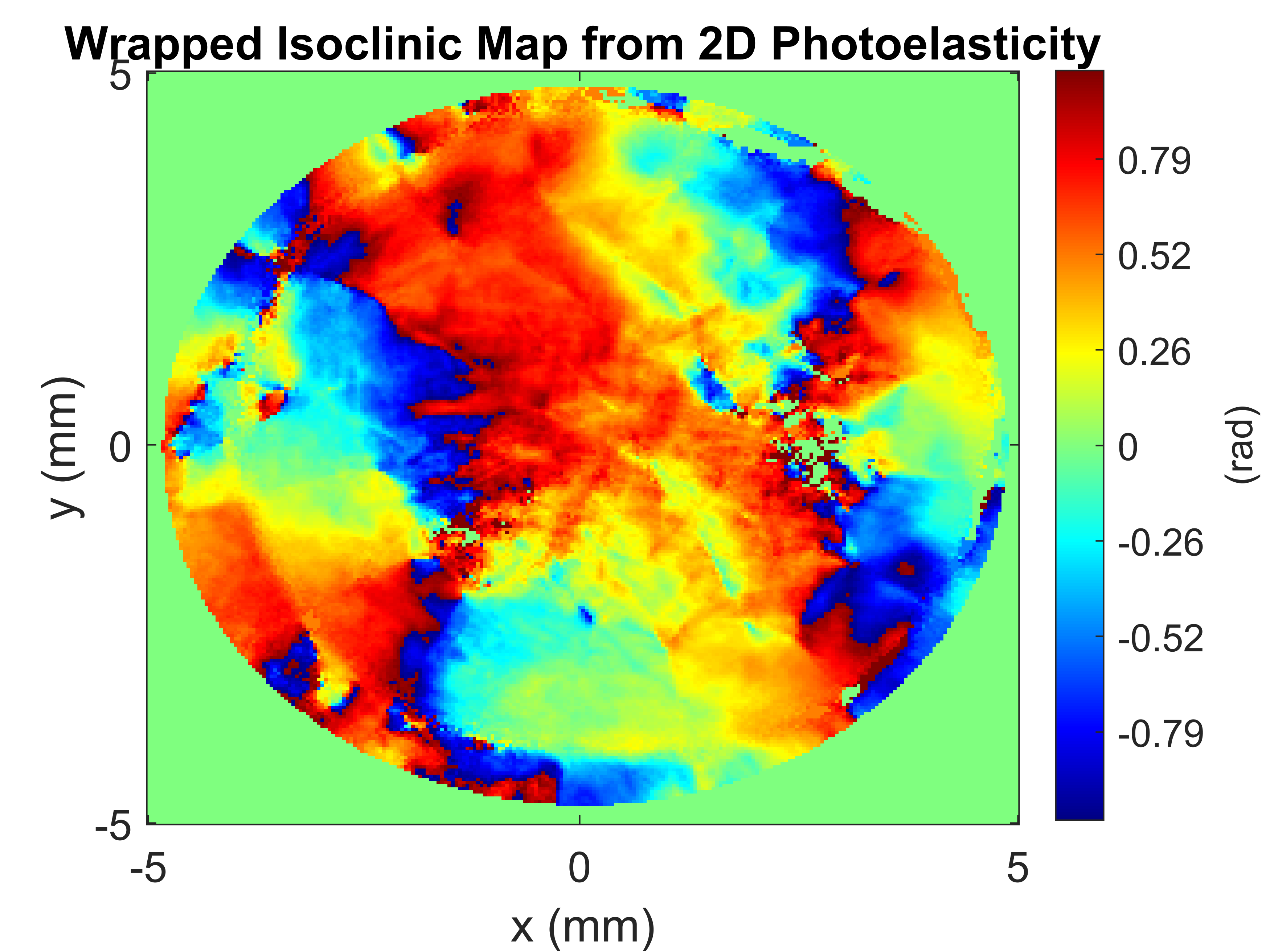

Supplement: S1 File — (ZIP) [file pone.0308204.s001.zip › S1 file. Birefringence Images/A-PK/30 degee/wppedISO.tif]

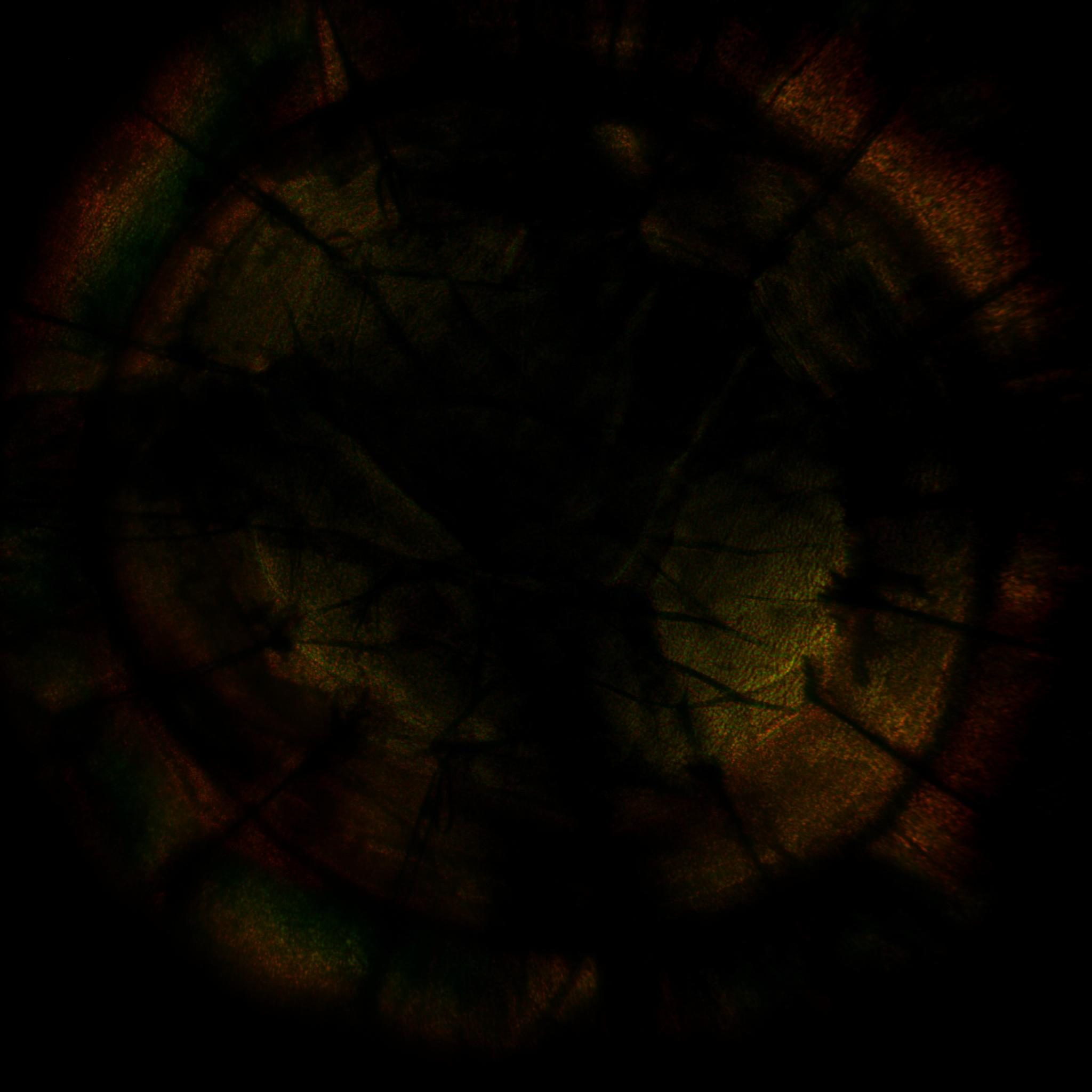

Supplement: S1 File — (ZIP) [file pone.0308204.s001.zip › S1 file. Birefringence Images/A-PK/45 degee/2751OD/IW1.jpg]

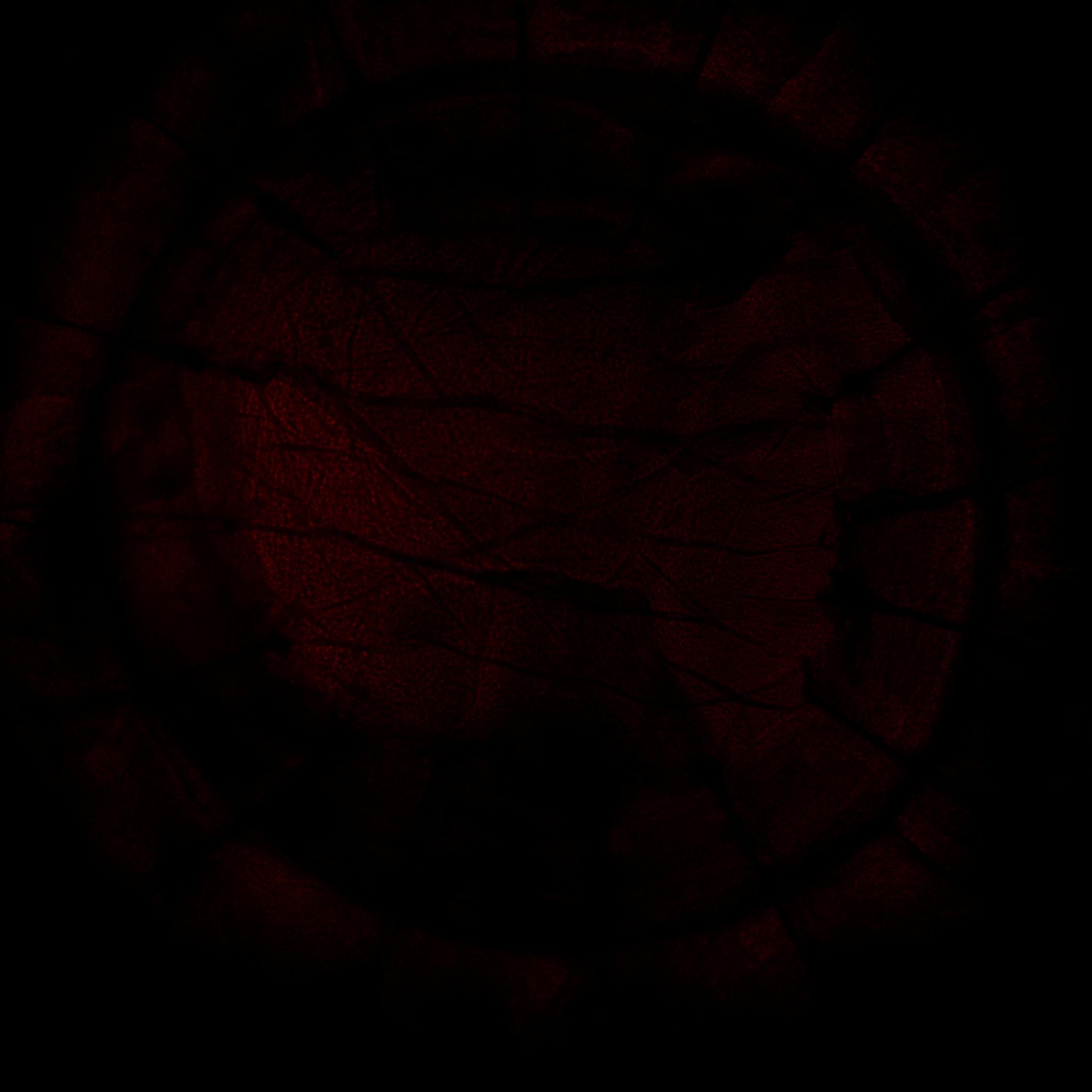

Supplement: S1 File — (ZIP) [file pone.0308204.s001.zip › S1 file. Birefringence Images/A-PK/45 degee/2751OD/IW10.jpg]

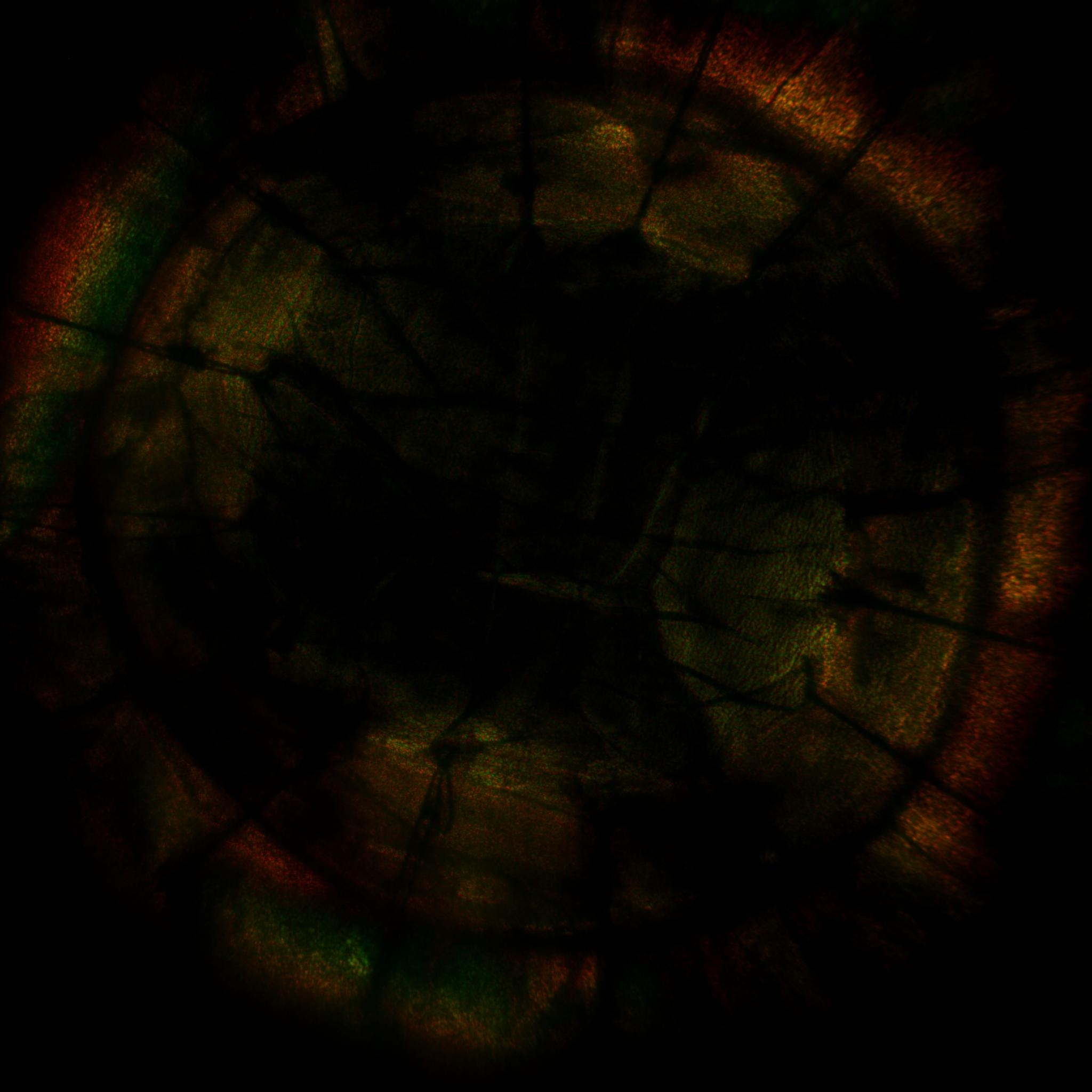

Supplement: S1 File — (ZIP) [file pone.0308204.s001.zip › S1 file. Birefringence Images/A-PK/45 degee/2751OD/IW2.jpg]

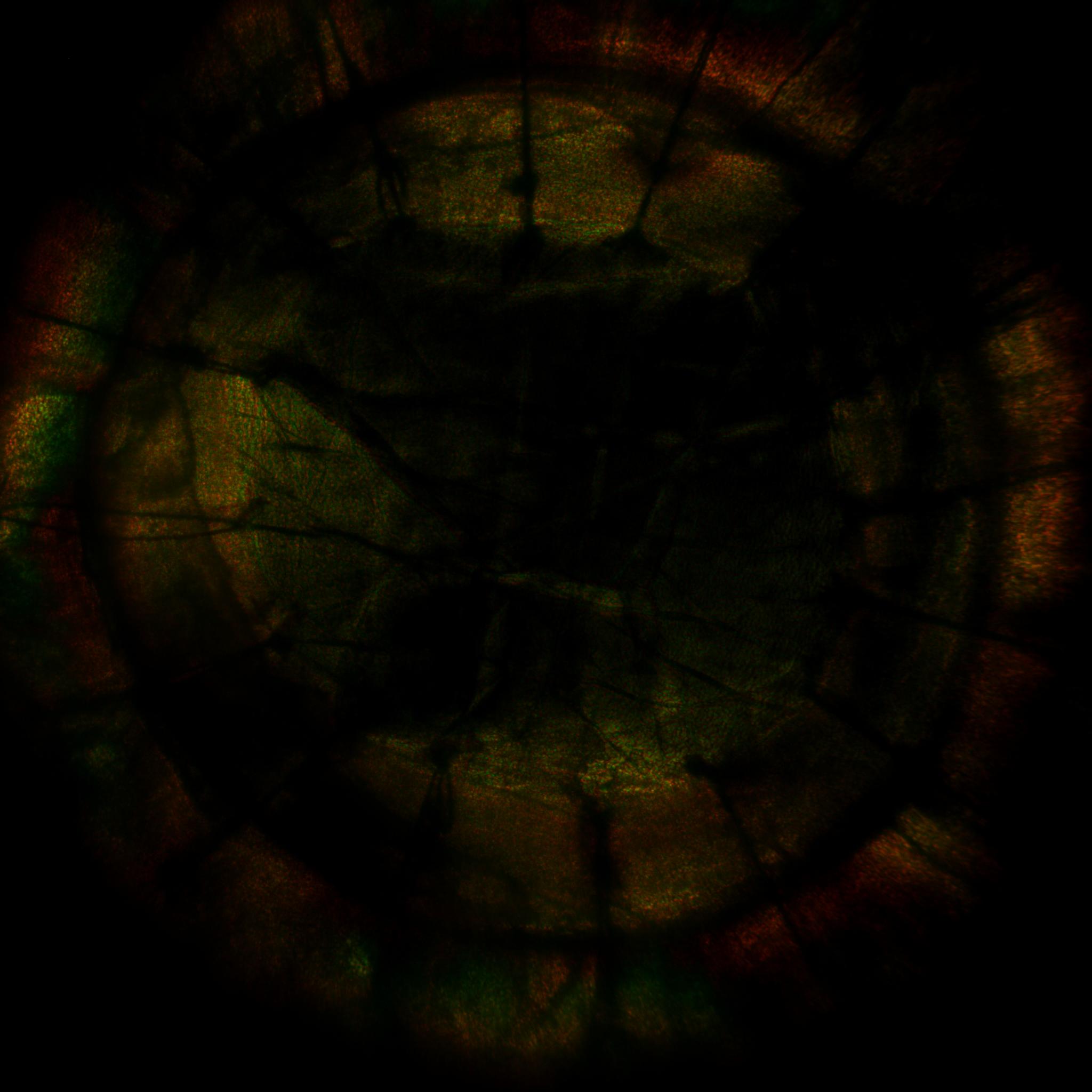

Supplement: S1 File — (ZIP) [file pone.0308204.s001.zip › S1 file. Birefringence Images/A-PK/45 degee/2751OD/IW3.jpg]

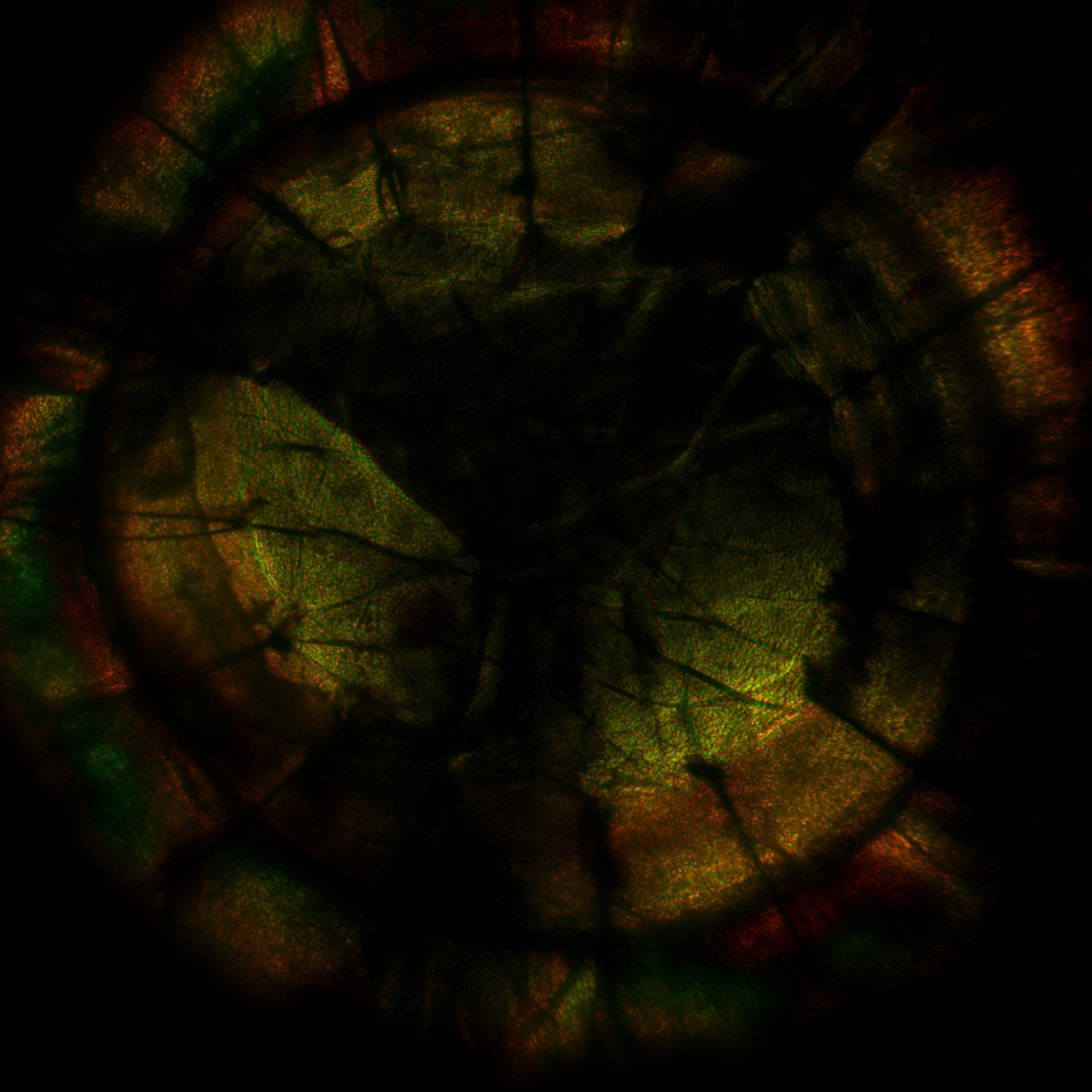

Supplement: S1 File — (ZIP) [file pone.0308204.s001.zip › S1 file. Birefringence Images/A-PK/45 degee/2751OD/IW4.jpg]

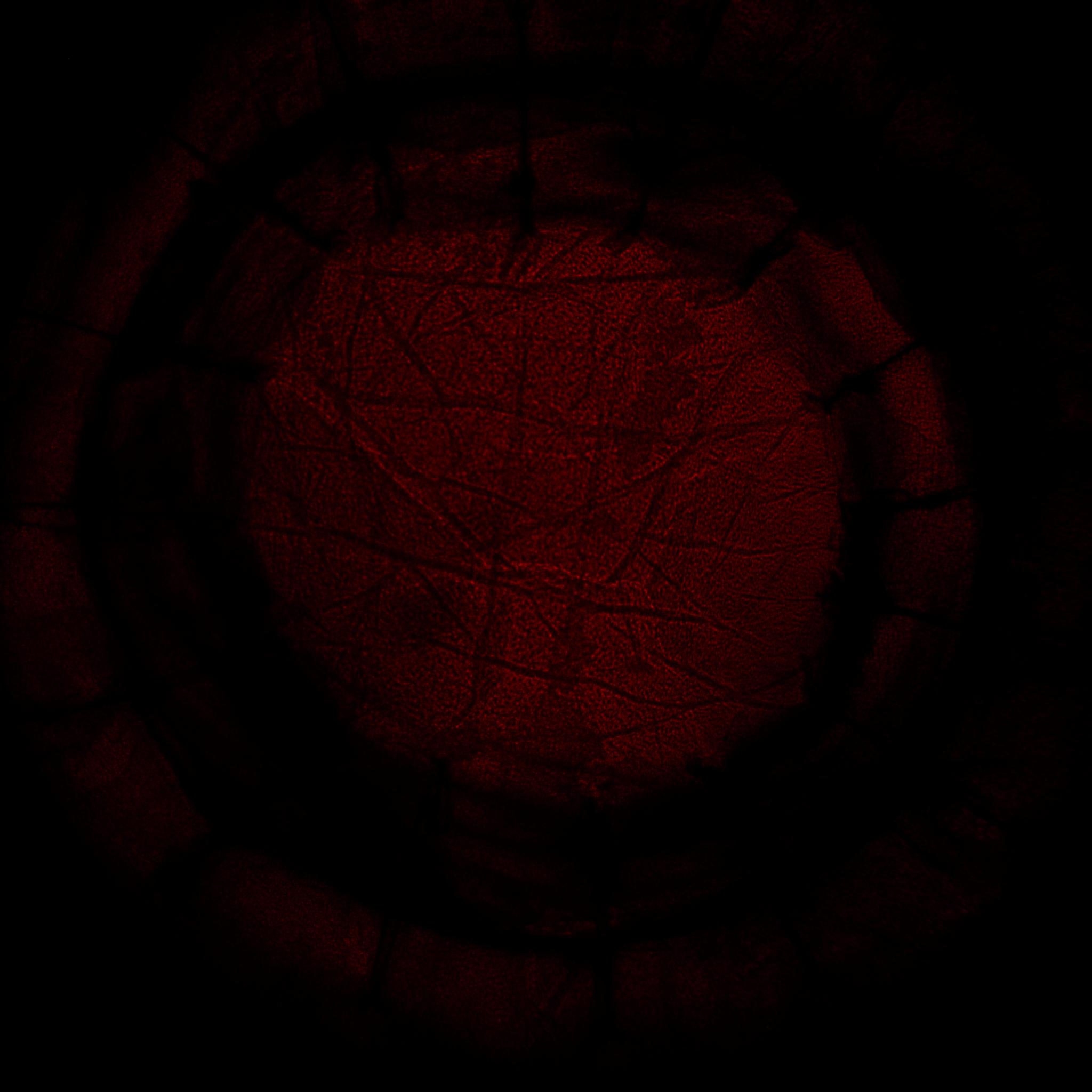

Supplement: S1 File — (ZIP) [file pone.0308204.s001.zip › S1 file. Birefringence Images/A-PK/45 degee/2751OD/IW5.jpg]

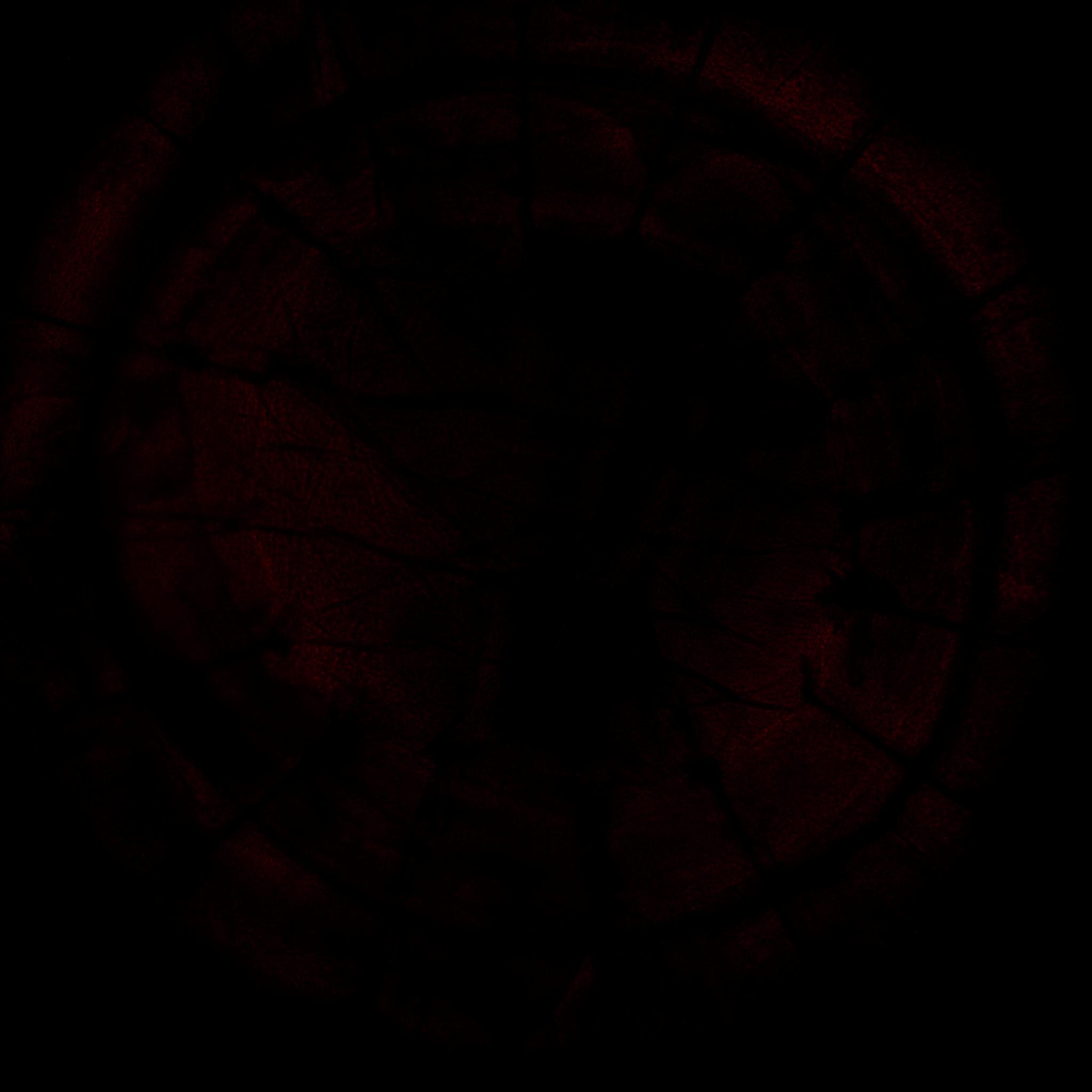

Supplement: S1 File — (ZIP) [file pone.0308204.s001.zip › S1 file. Birefringence Images/A-PK/45 degee/2751OD/IW6.jpg]

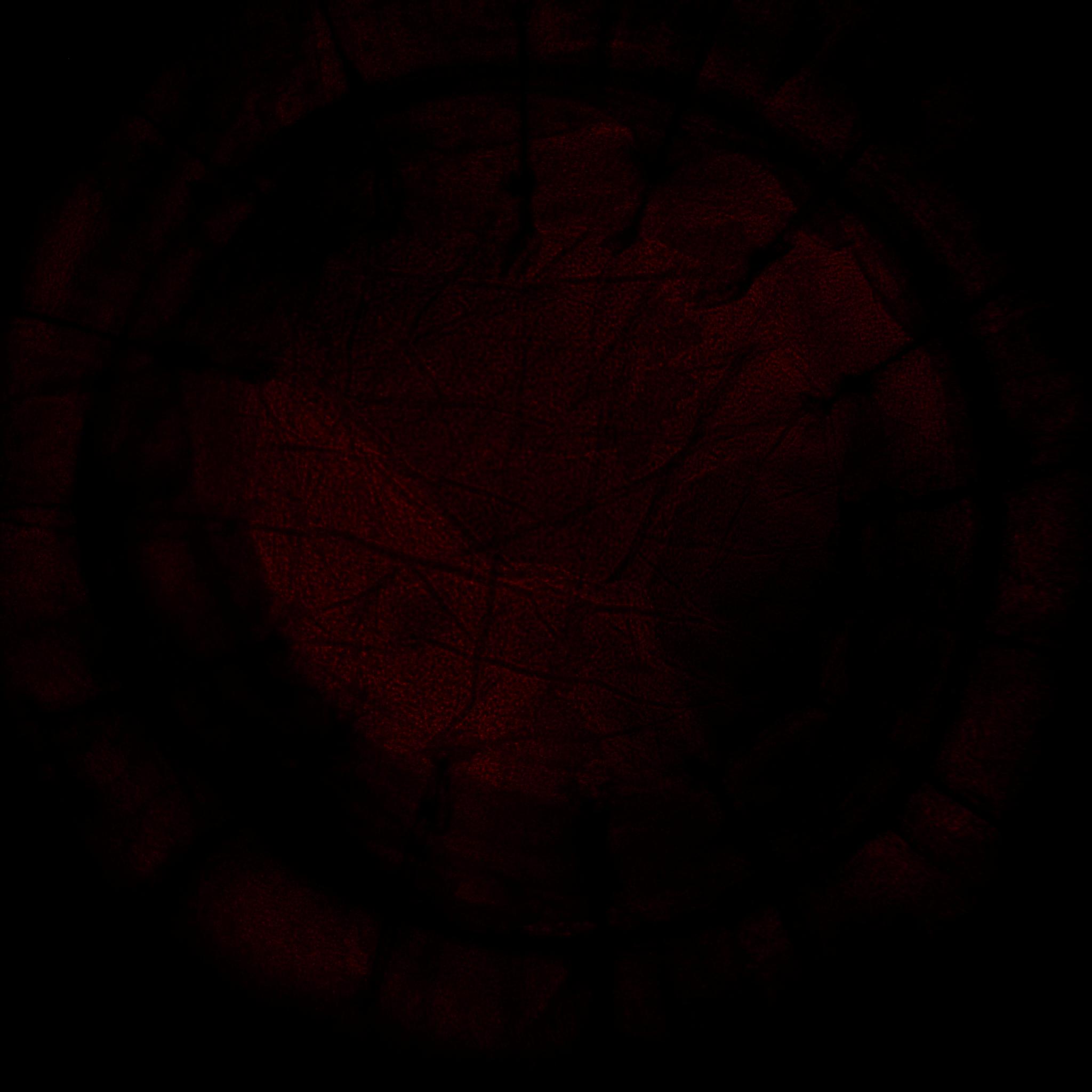

Supplement: S1 File — (ZIP) [file pone.0308204.s001.zip › S1 file. Birefringence Images/A-PK/45 degee/2751OD/IW7.jpg]

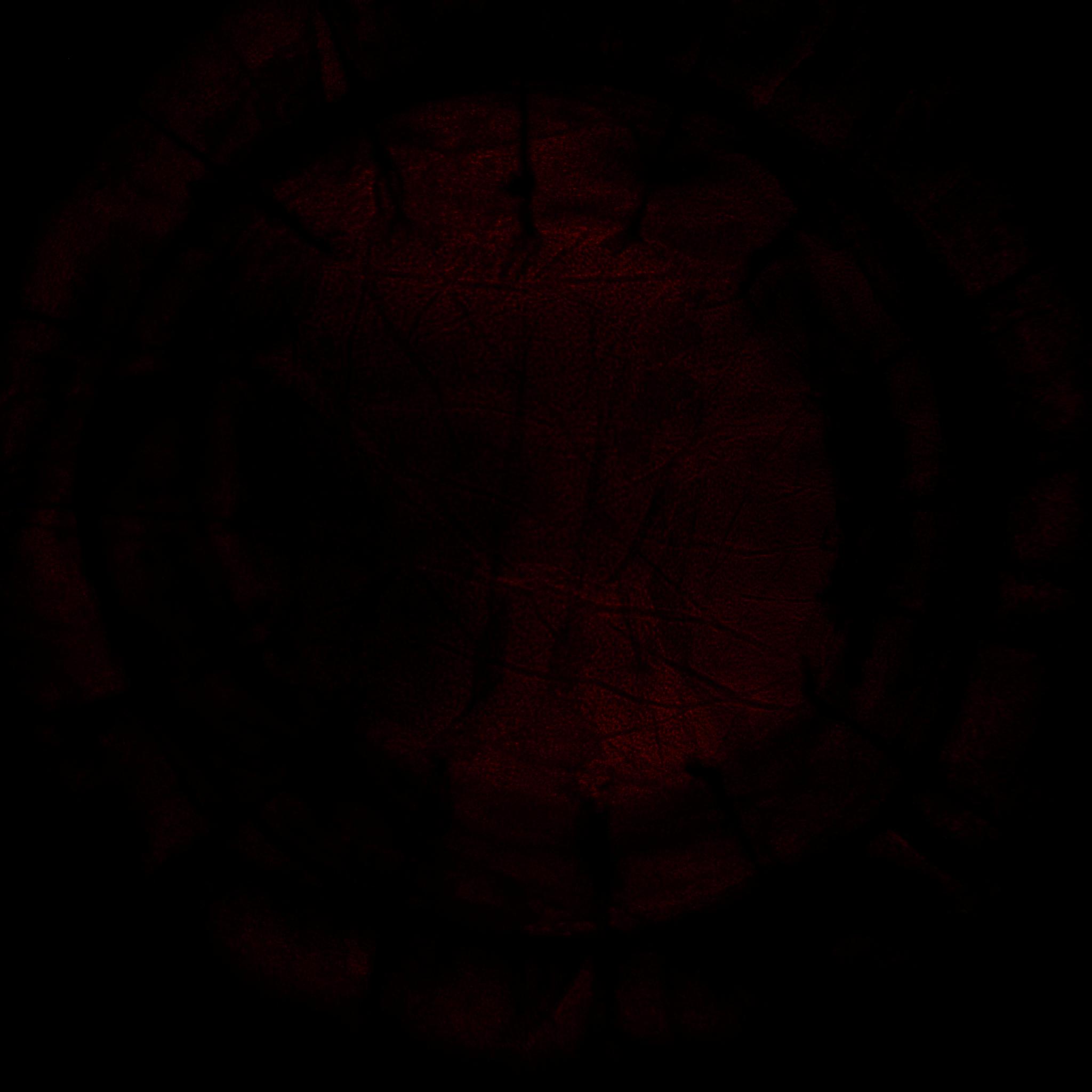

Supplement: S1 File — (ZIP) [file pone.0308204.s001.zip › S1 file. Birefringence Images/A-PK/45 degee/2751OD/IW8.jpg]

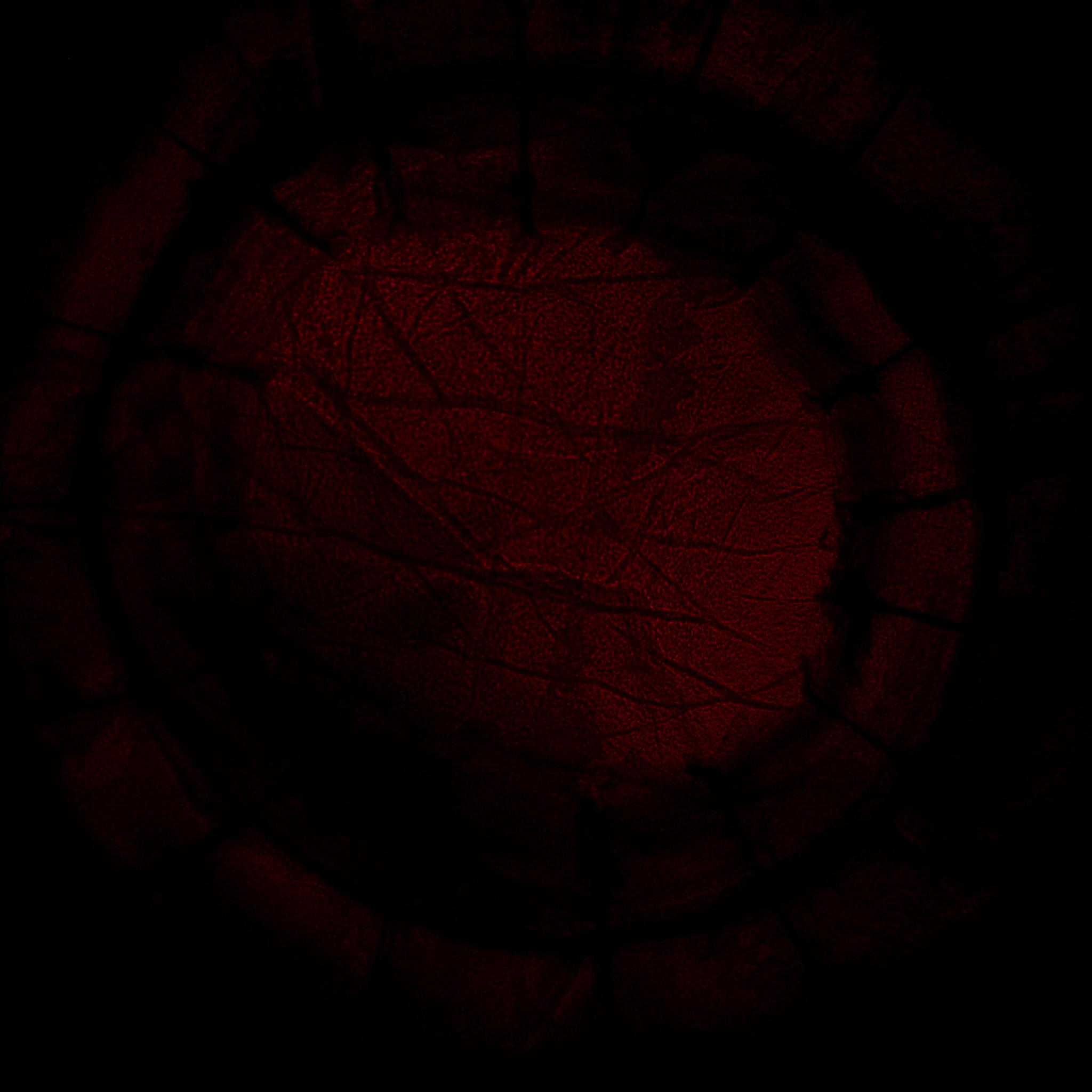

Supplement: S1 File — (ZIP) [file pone.0308204.s001.zip › S1 file. Birefringence Images/A-PK/45 degee/2751OD/IW9.jpg]

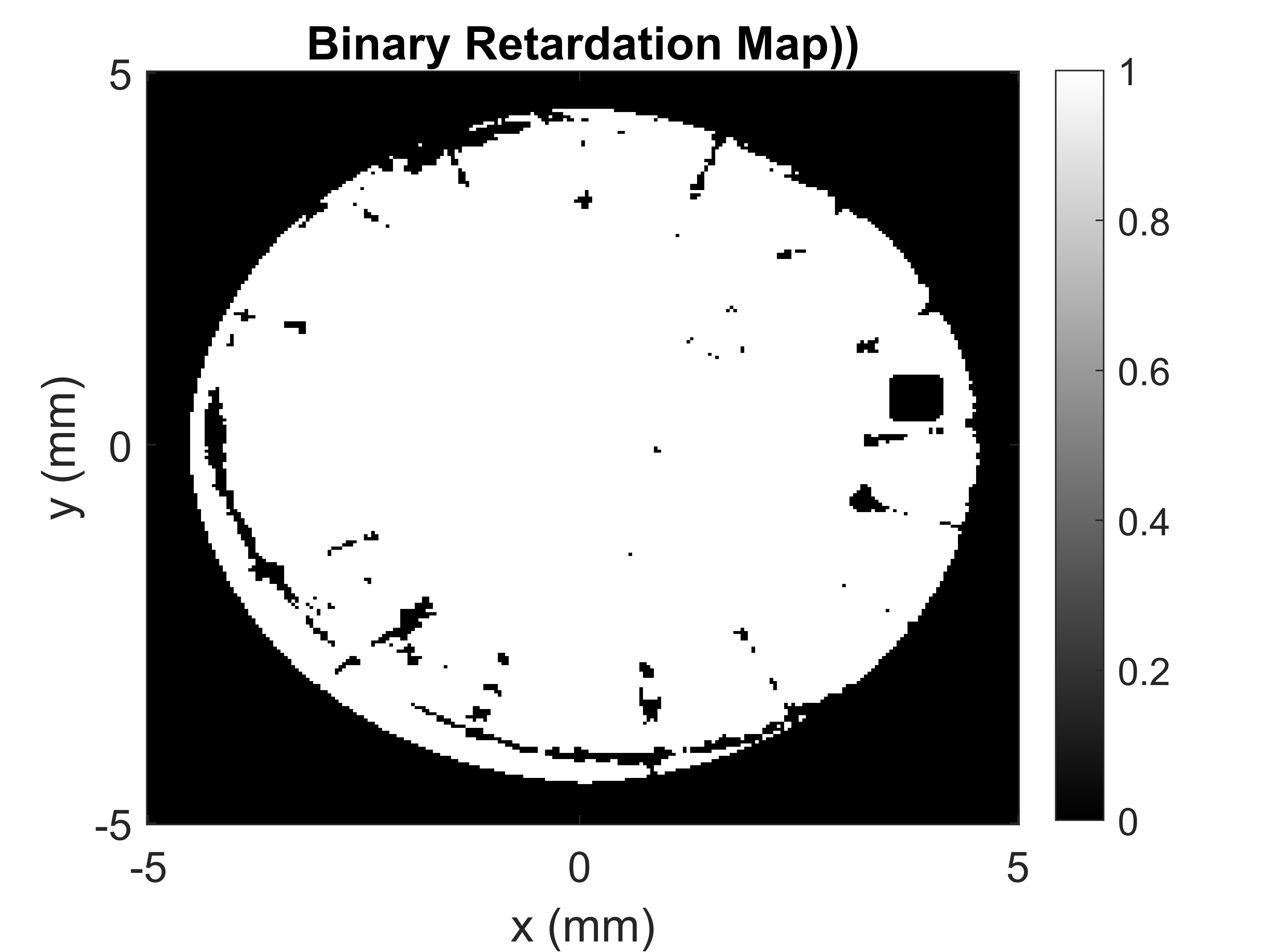

Supplement: S1 File — (ZIP) [file pone.0308204.s001.zip › S1 file. Birefringence Images/A-PK/45 degee/2751OD/suuepoins.tif]

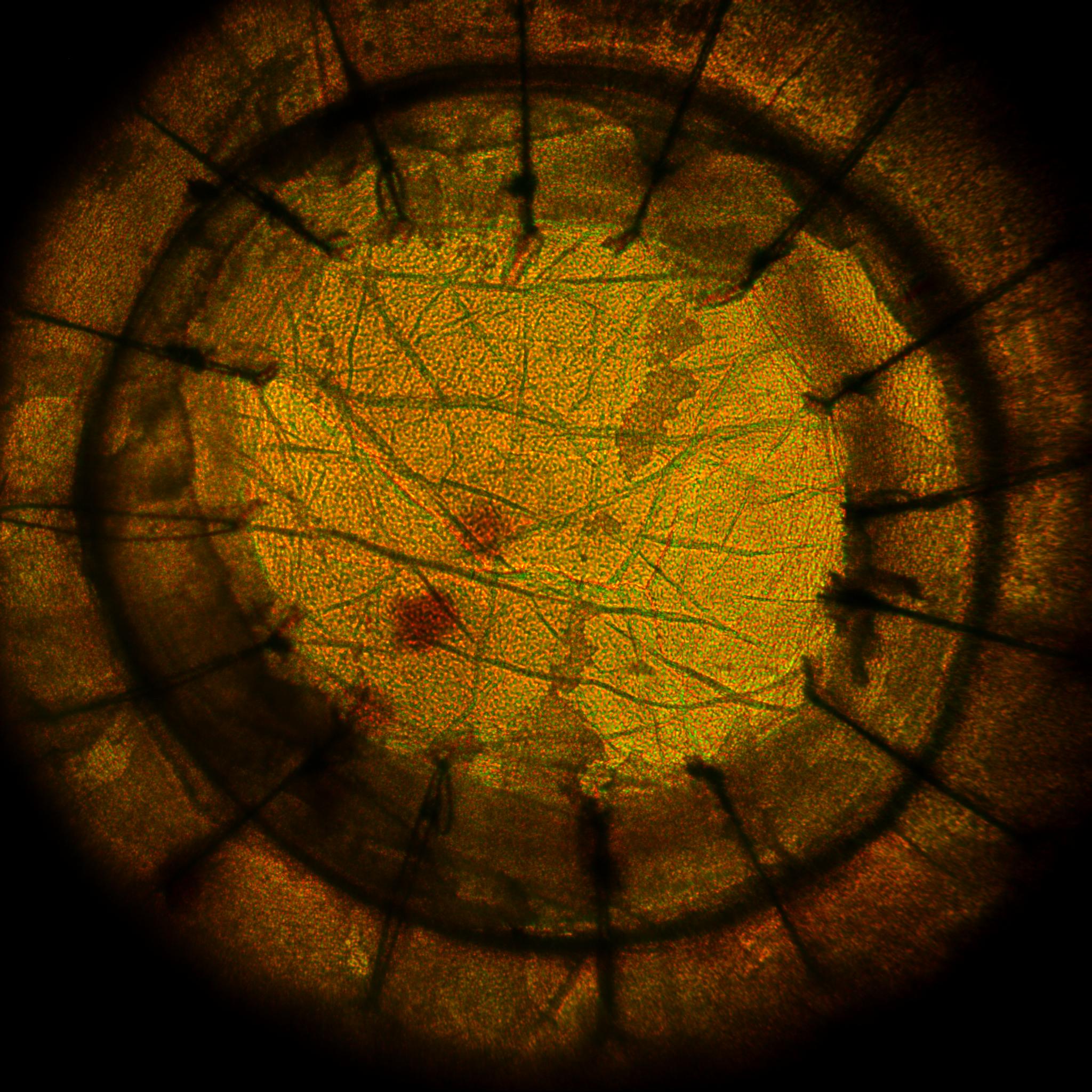

Supplement: S1 File — (ZIP) [file pone.0308204.s001.zip › S1 file. Birefringence Images/A-PK/45 degee/2751OD/suures.jpg]

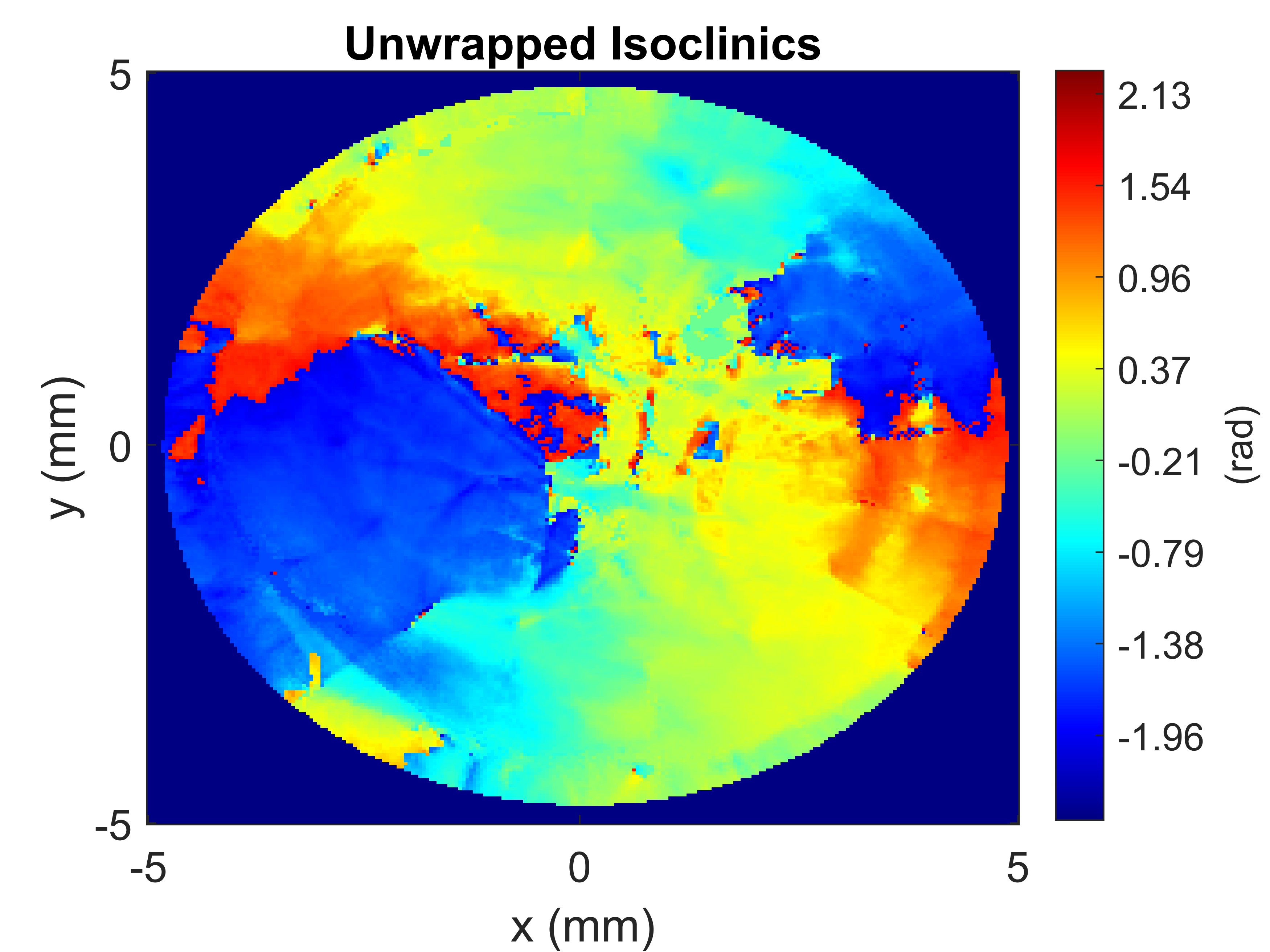

Supplement: S1 File — (ZIP) [file pone.0308204.s001.zip › S1 file. Birefringence Images/A-PK/45 degee/2751OD/unwappedISO.tif]

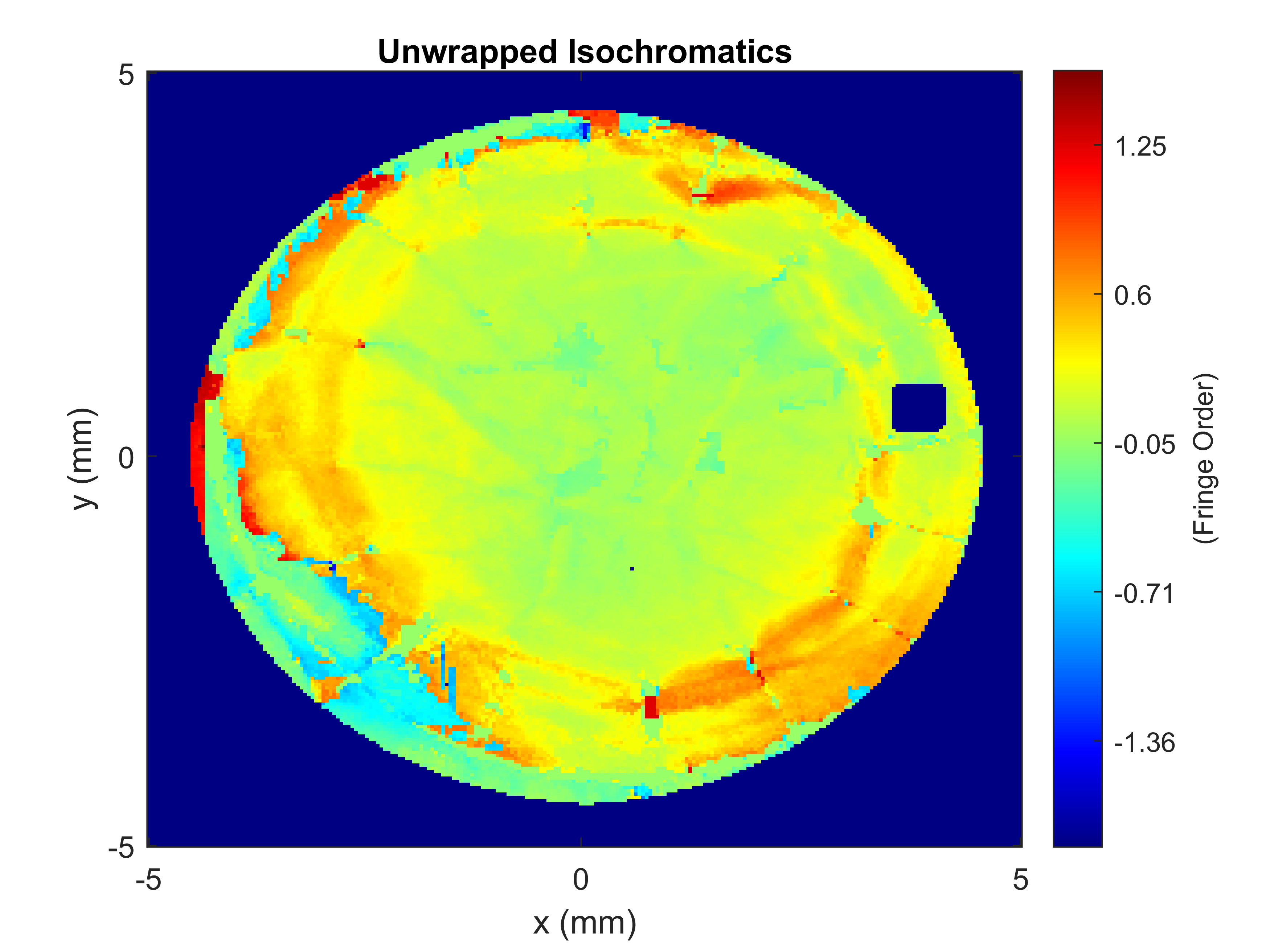

Supplement: S1 File — (ZIP) [file pone.0308204.s001.zip › S1 file. Birefringence Images/A-PK/45 degee/2751OD/unwappedISOCH.tif]

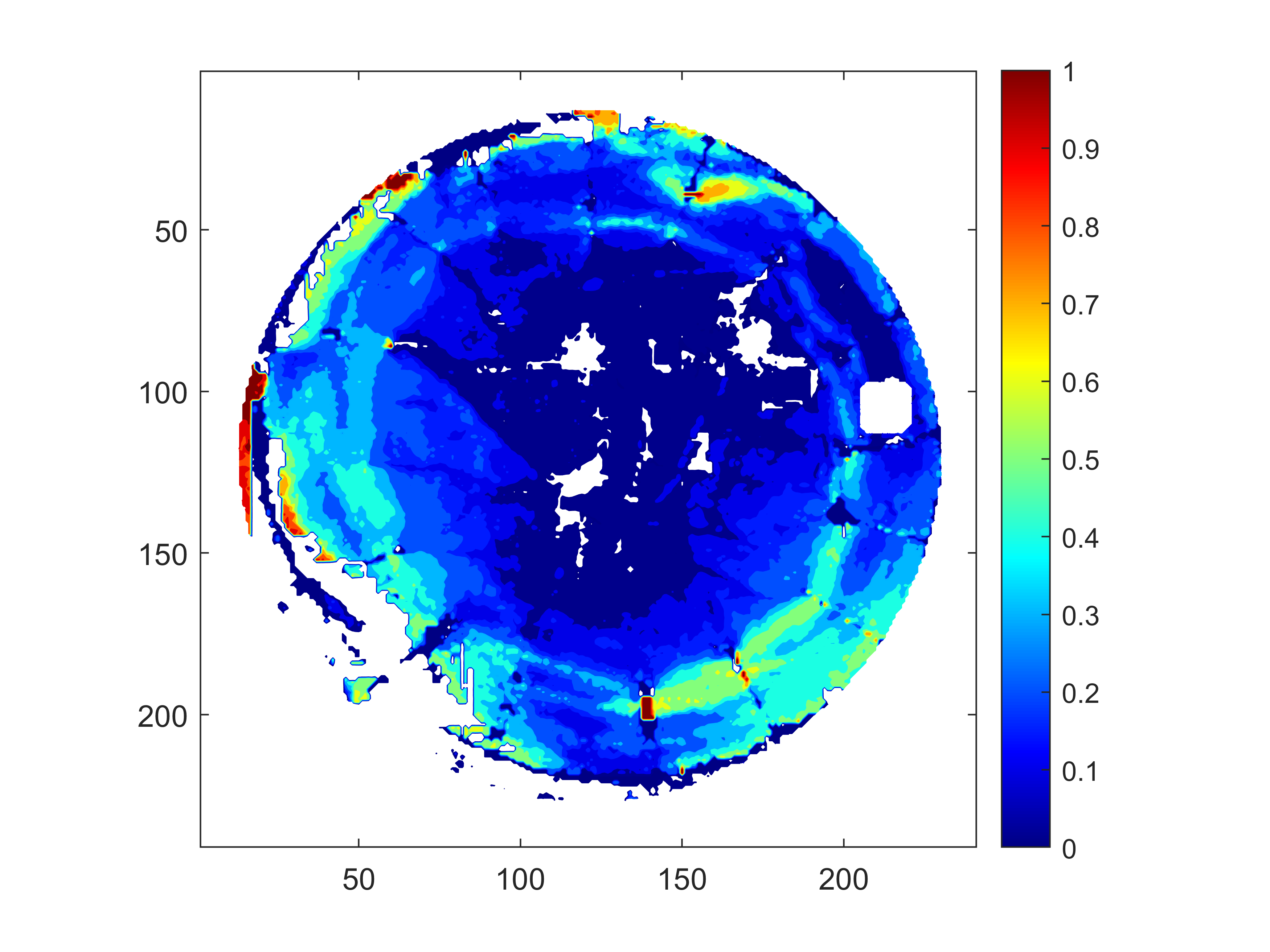

Supplement: S1 File — (ZIP) [file pone.0308204.s001.zip › S1 file. Birefringence Images/A-PK/45 degee/2751OD/unwappedISOCHfilled.tif]

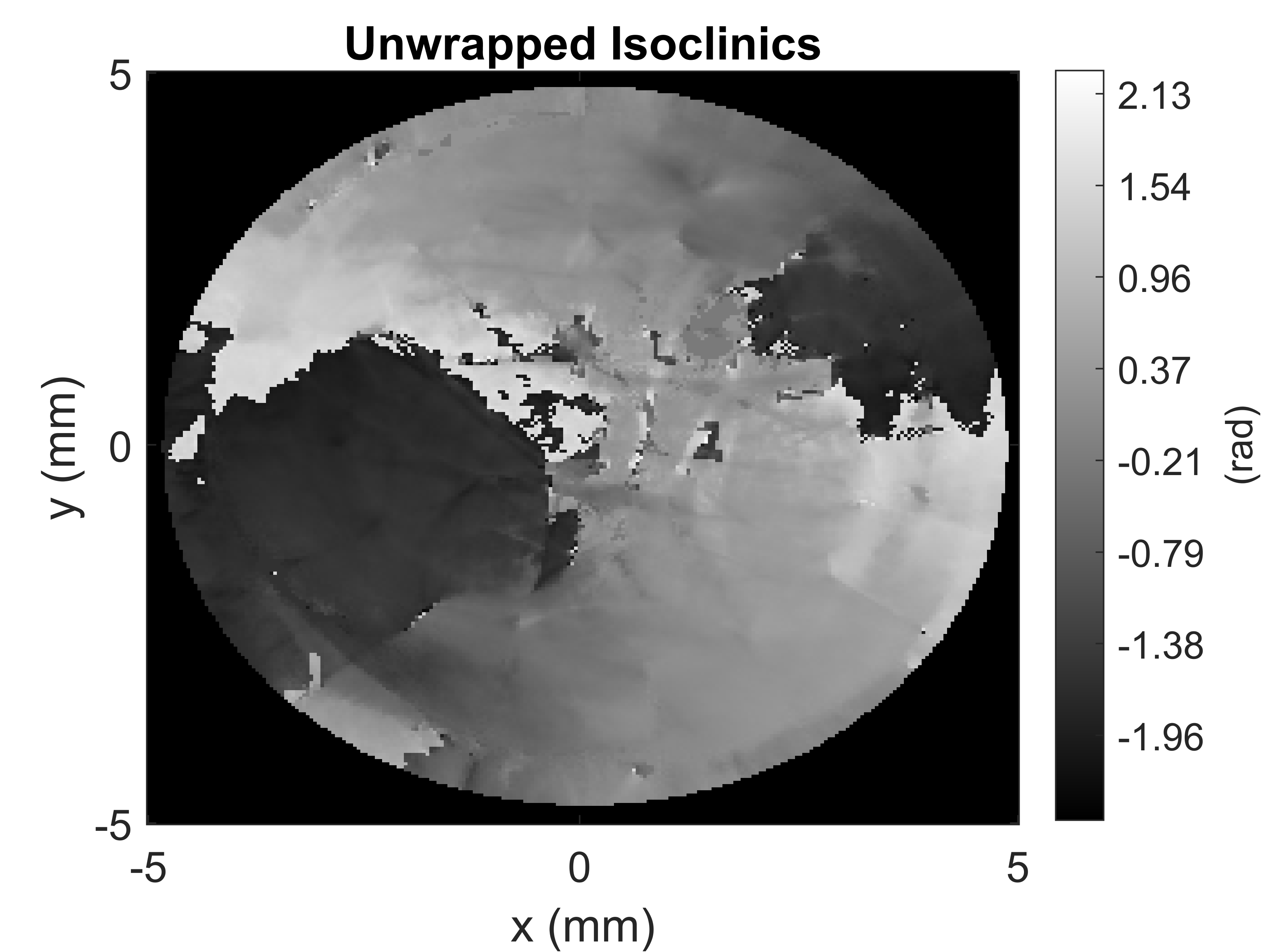

Supplement: S1 File — (ZIP) [file pone.0308204.s001.zip › S1 file. Birefringence Images/A-PK/45 degee/2751OD/unwappedISOgy.tif]

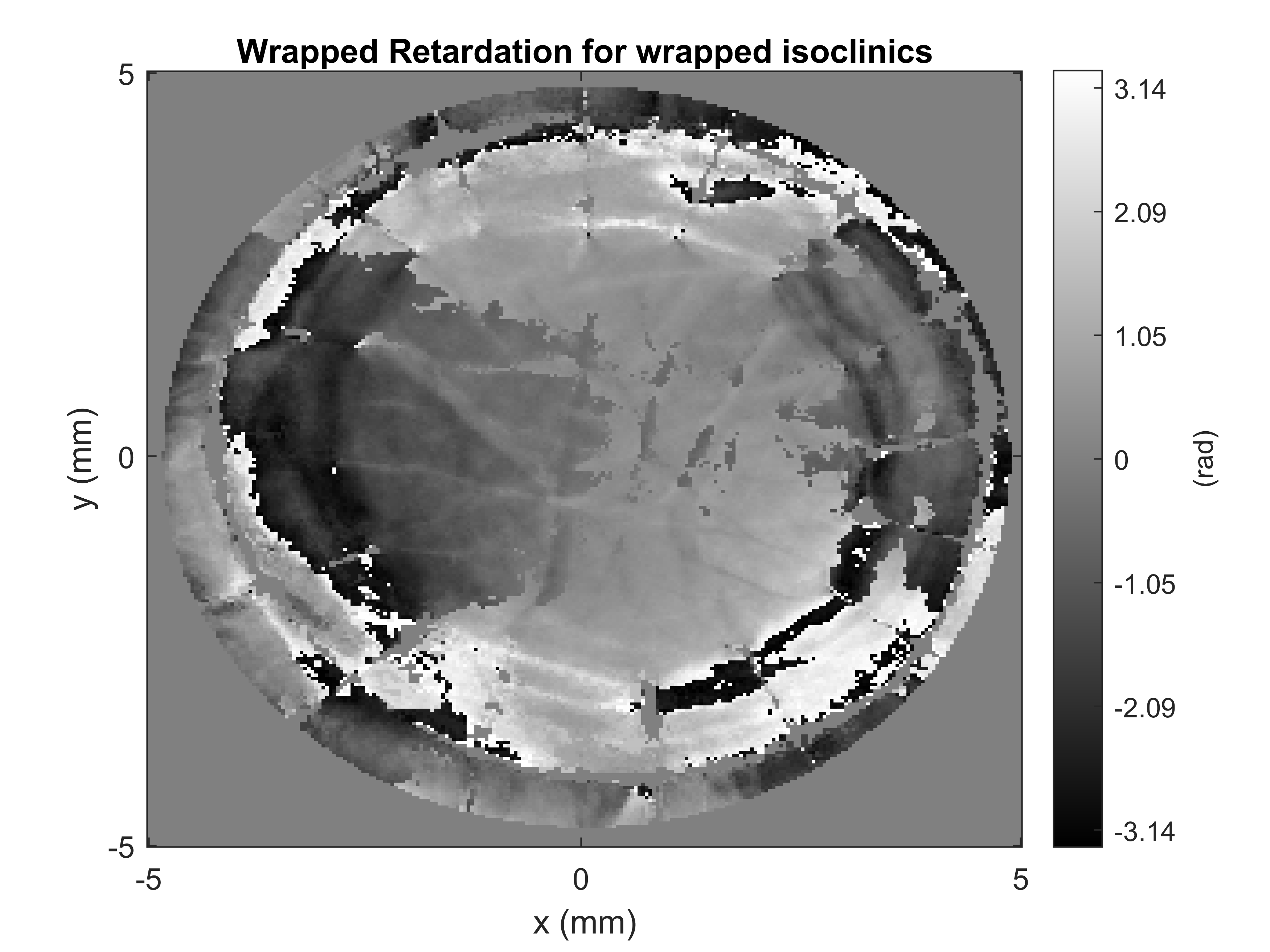

Supplement: S1 File — (ZIP) [file pone.0308204.s001.zip › S1 file. Birefringence Images/A-PK/45 degee/2751OD/wappedISOCHwappedISO.tif]

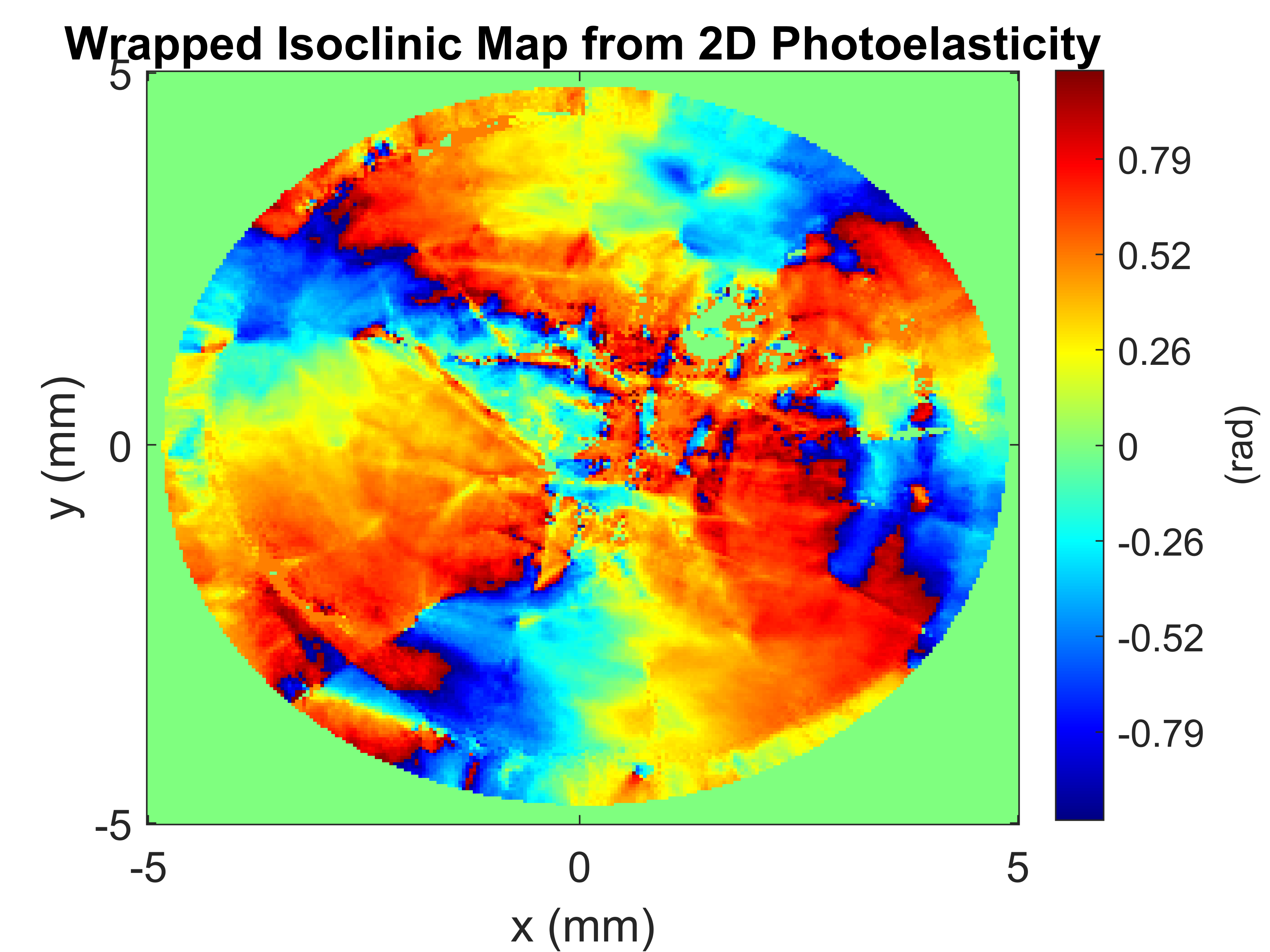

Supplement: S1 File — (ZIP) [file pone.0308204.s001.zip › S1 file. Birefringence Images/A-PK/45 degee/2751OD/wppedISO.tif]

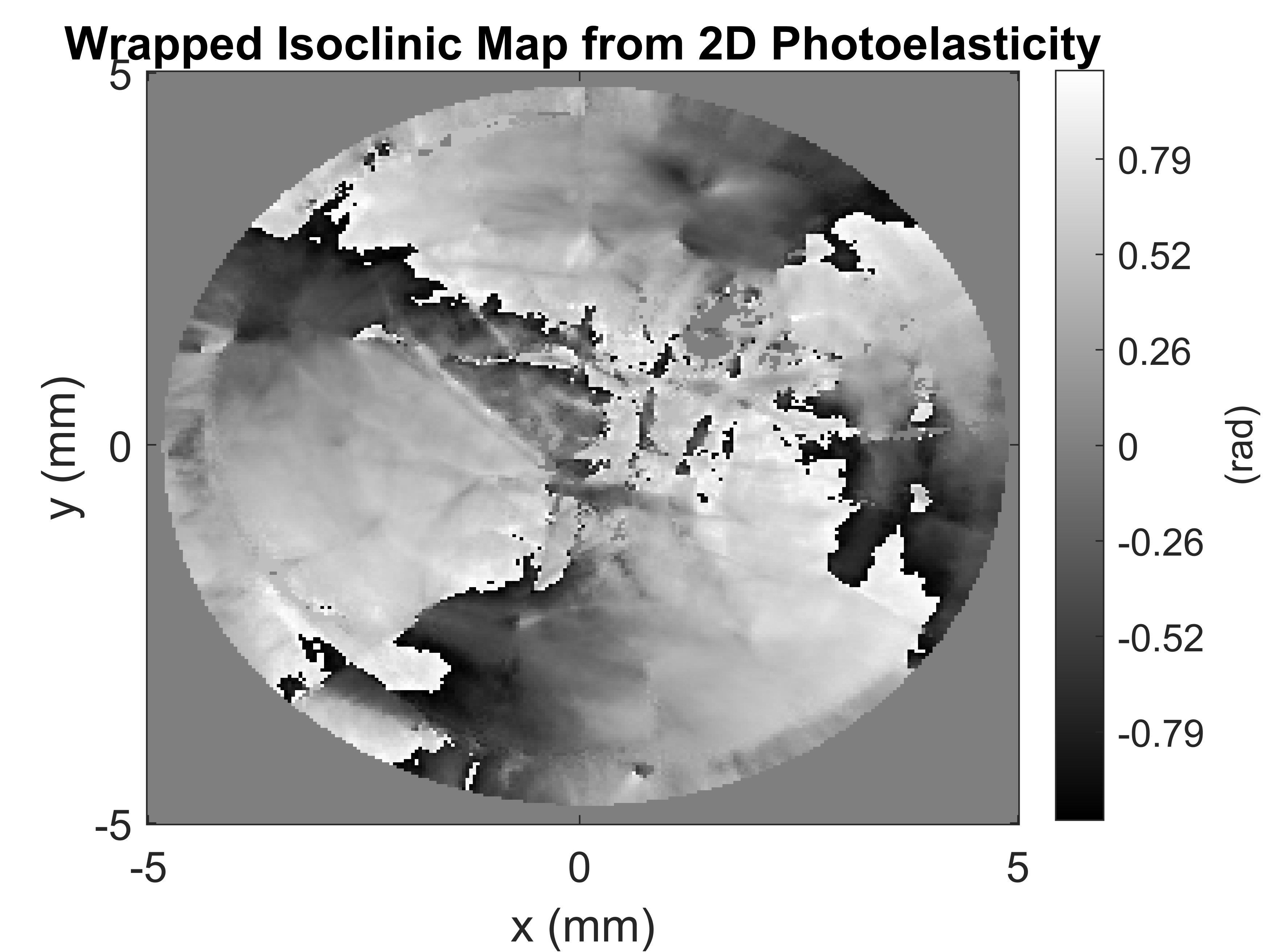

Supplement: S1 File — (ZIP) [file pone.0308204.s001.zip › S1 file. Birefringence Images/A-PK/45 degee/2751OD/wppedISOgay.tif]

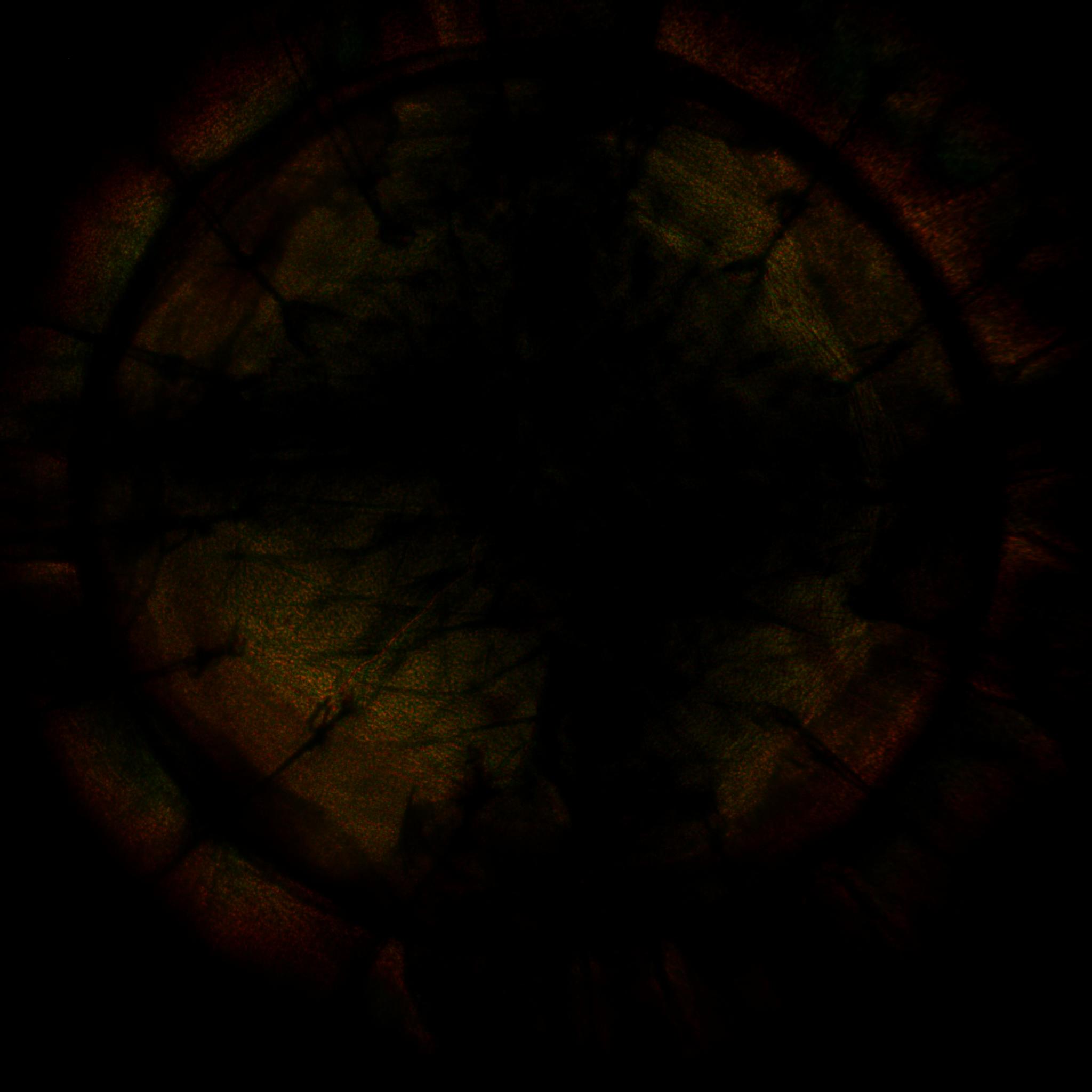

Supplement: S1 File — (ZIP) [file pone.0308204.s001.zip › S1 file. Birefringence Images/A-PK/60 degee/2693OS/IW1.jpg]

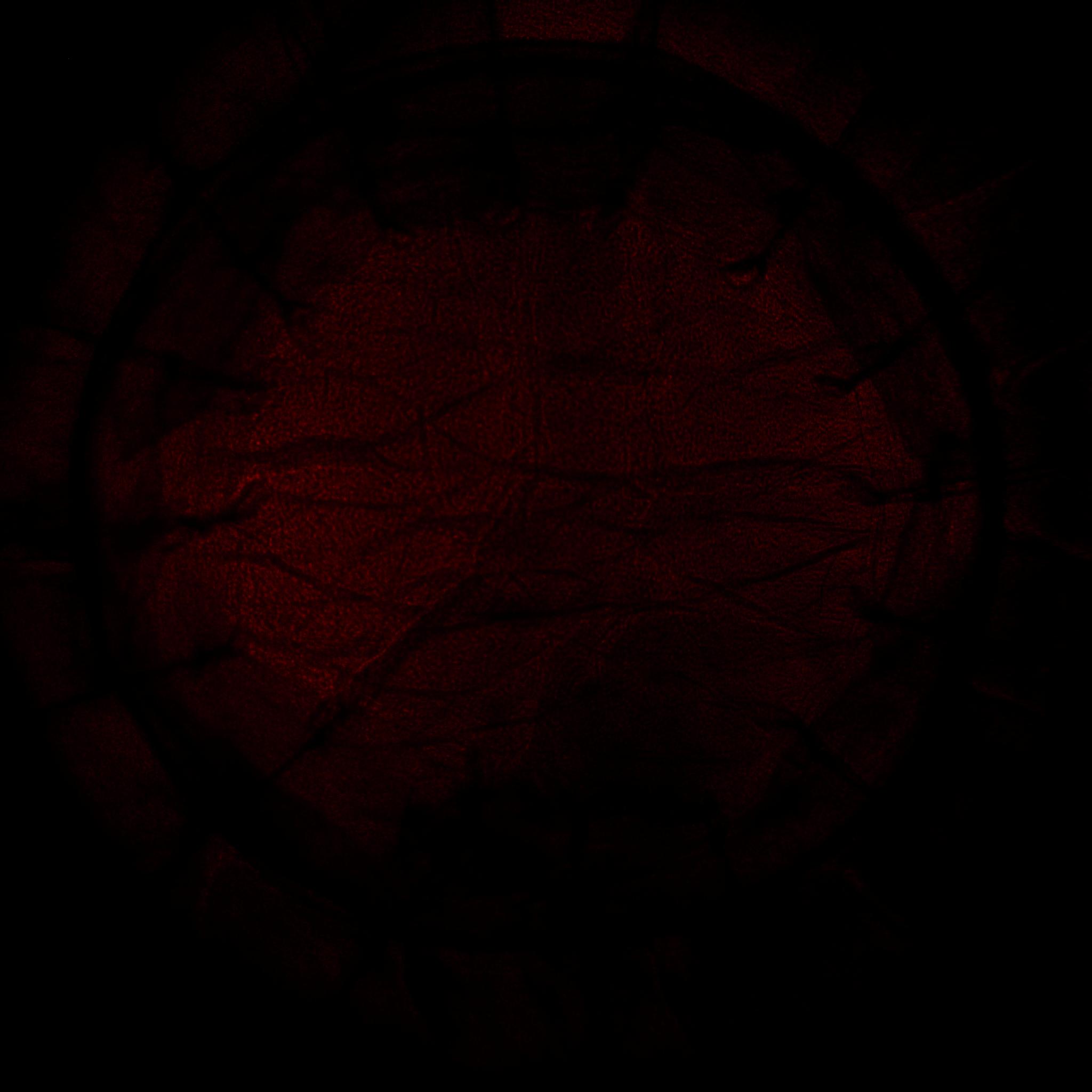

Supplement: S1 File — (ZIP) [file pone.0308204.s001.zip › S1 file. Birefringence Images/A-PK/60 degee/2693OS/IW10.jpg]

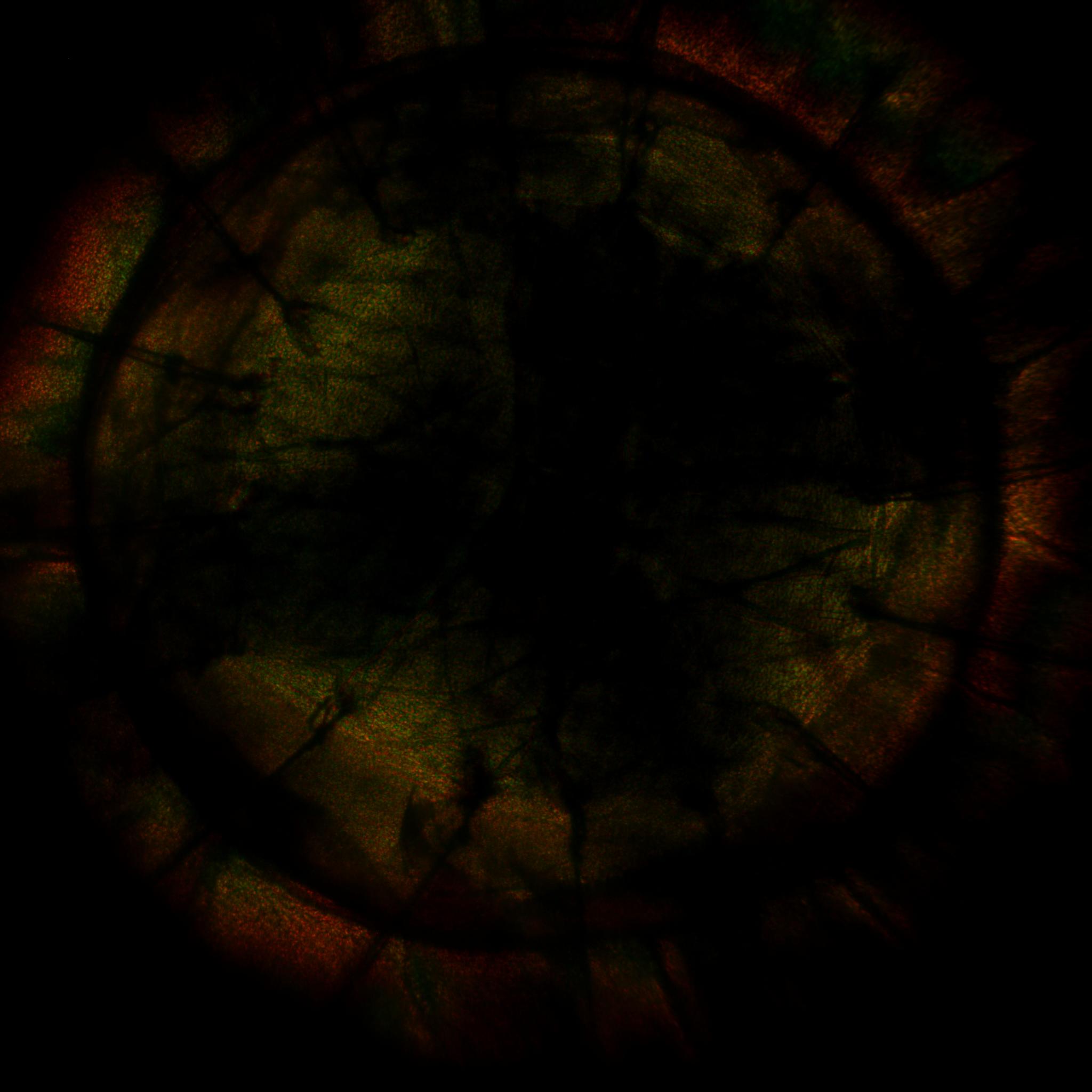

Supplement: S1 File — (ZIP) [file pone.0308204.s001.zip › S1 file. Birefringence Images/A-PK/60 degee/2693OS/IW2.jpg]

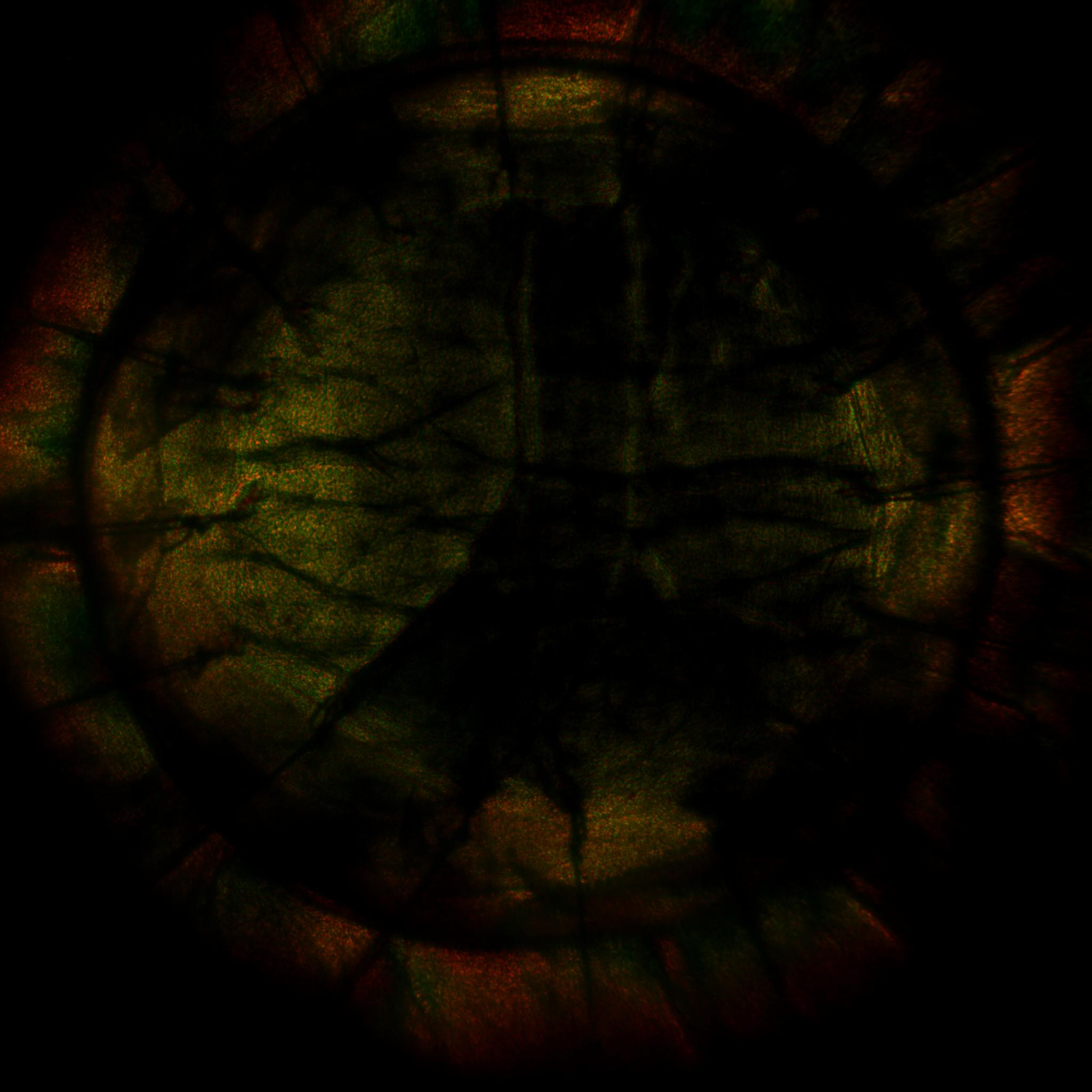

Supplement: S1 File — (ZIP) [file pone.0308204.s001.zip › S1 file. Birefringence Images/A-PK/60 degee/2693OS/IW3.jpg]

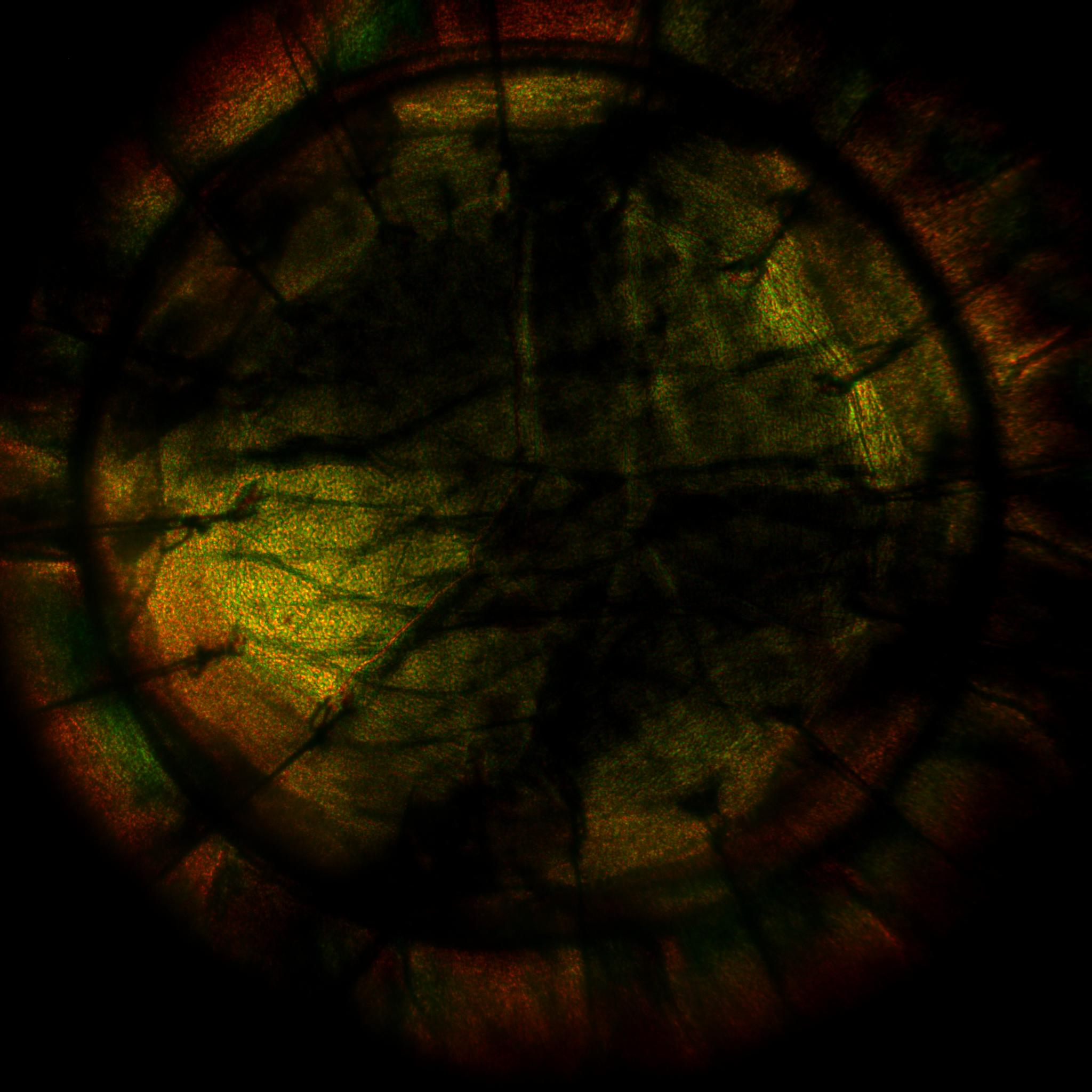

Supplement: S1 File — (ZIP) [file pone.0308204.s001.zip › S1 file. Birefringence Images/A-PK/60 degee/2693OS/IW4.jpg]

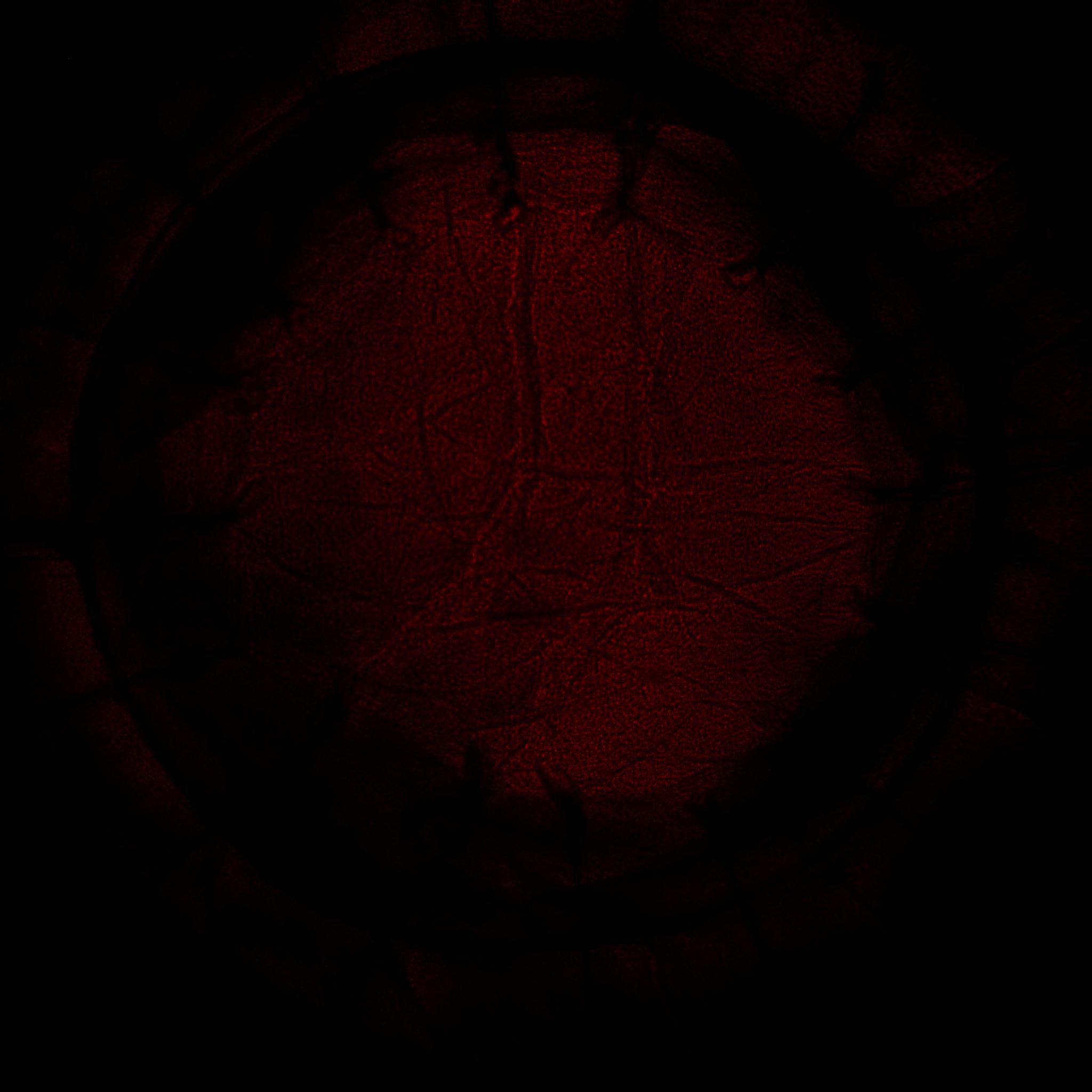

Supplement: S1 File — (ZIP) [file pone.0308204.s001.zip › S1 file. Birefringence Images/A-PK/60 degee/2693OS/IW5.jpg]

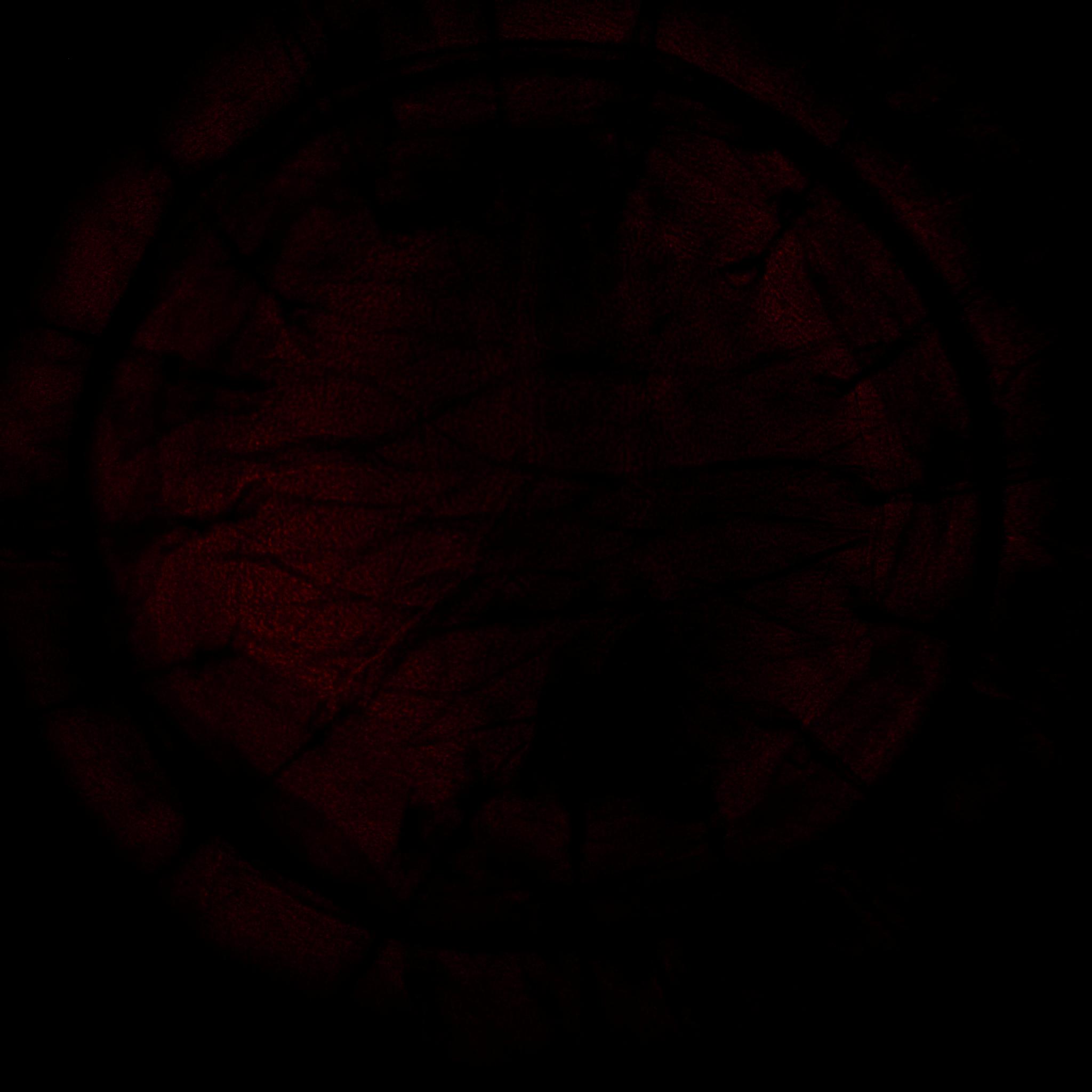

Supplement: S1 File — (ZIP) [file pone.0308204.s001.zip › S1 file. Birefringence Images/A-PK/60 degee/2693OS/IW6.jpg]

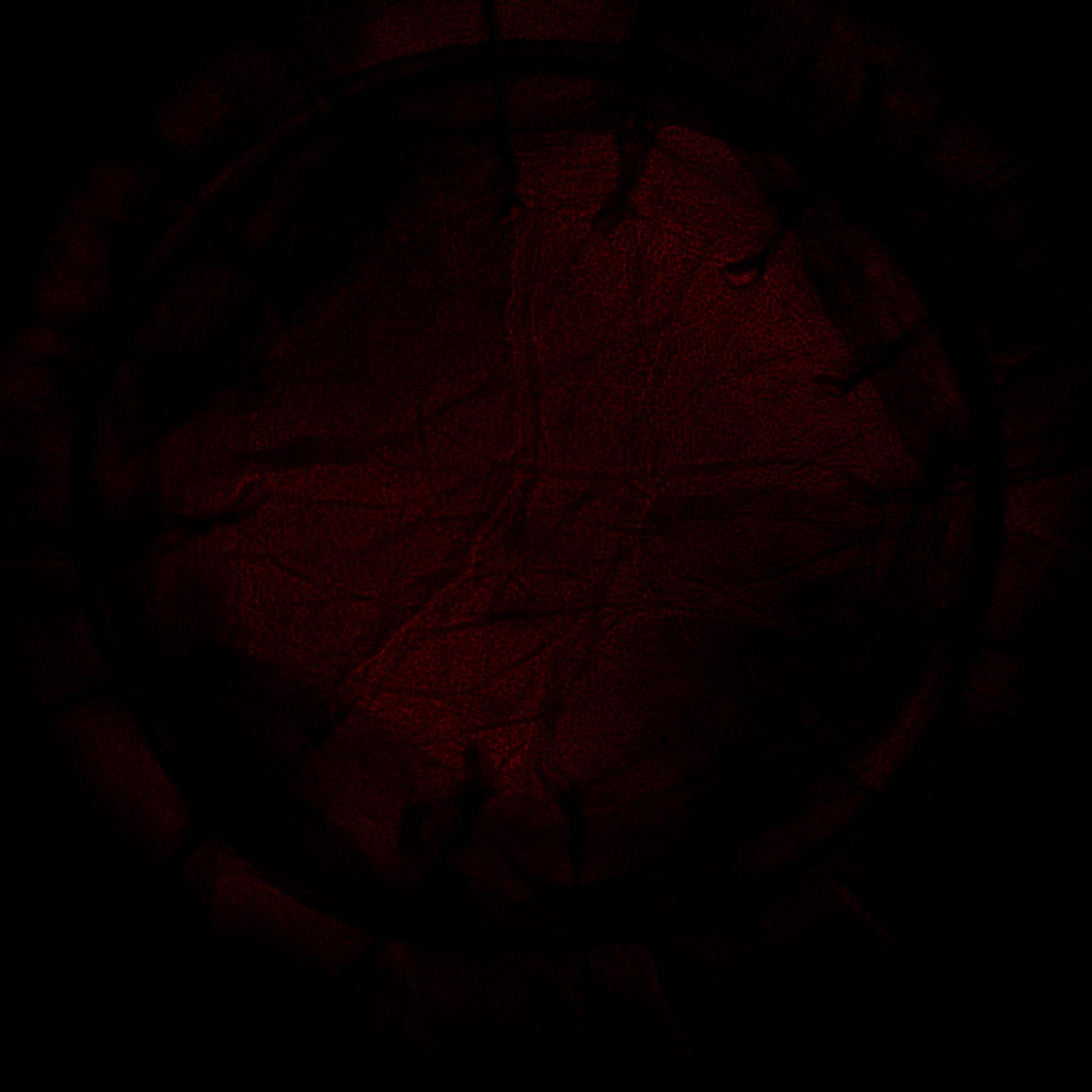

Supplement: S1 File — (ZIP) [file pone.0308204.s001.zip › S1 file. Birefringence Images/A-PK/60 degee/2693OS/IW7.jpg]

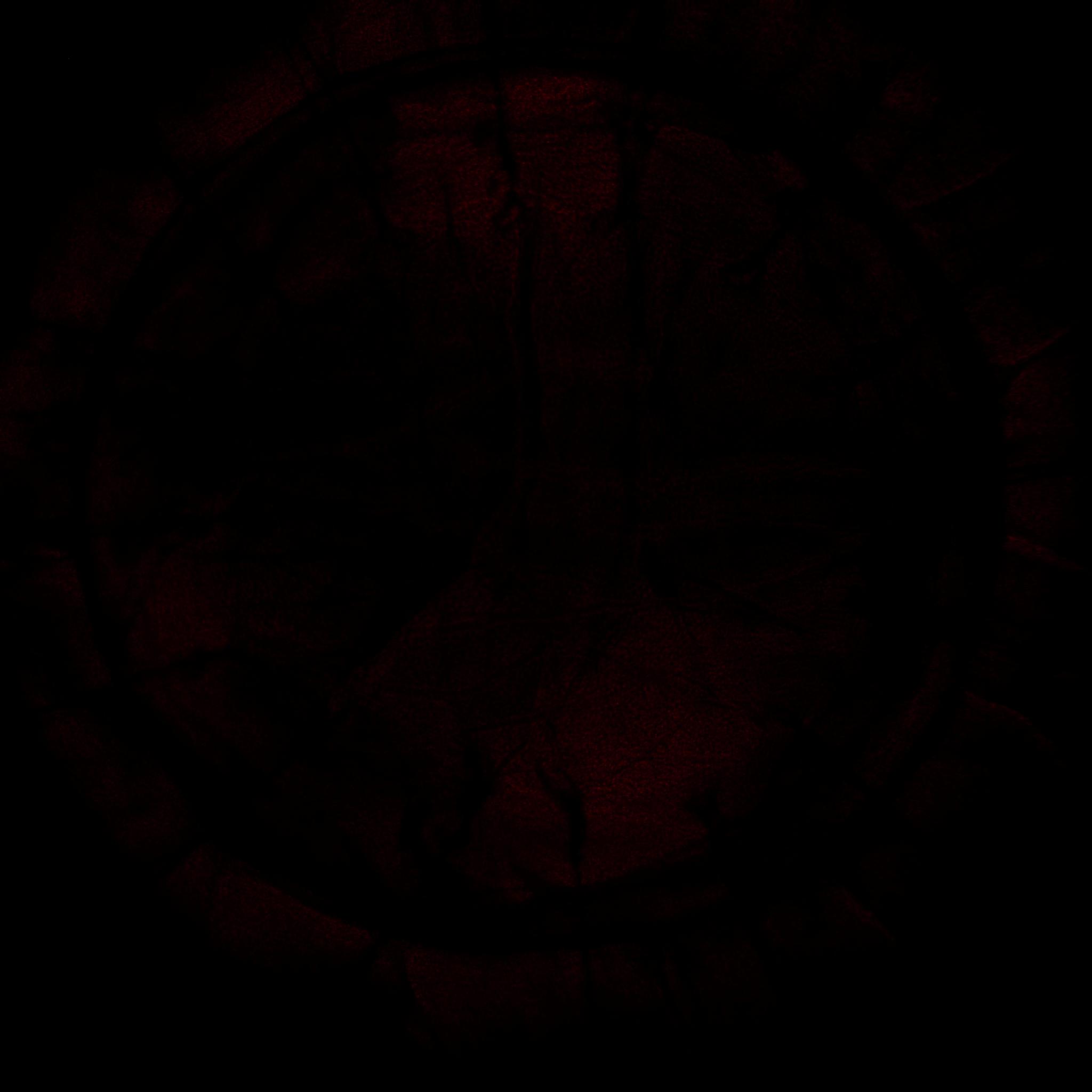

Supplement: S1 File — (ZIP) [file pone.0308204.s001.zip › S1 file. Birefringence Images/A-PK/60 degee/2693OS/IW8.jpg]

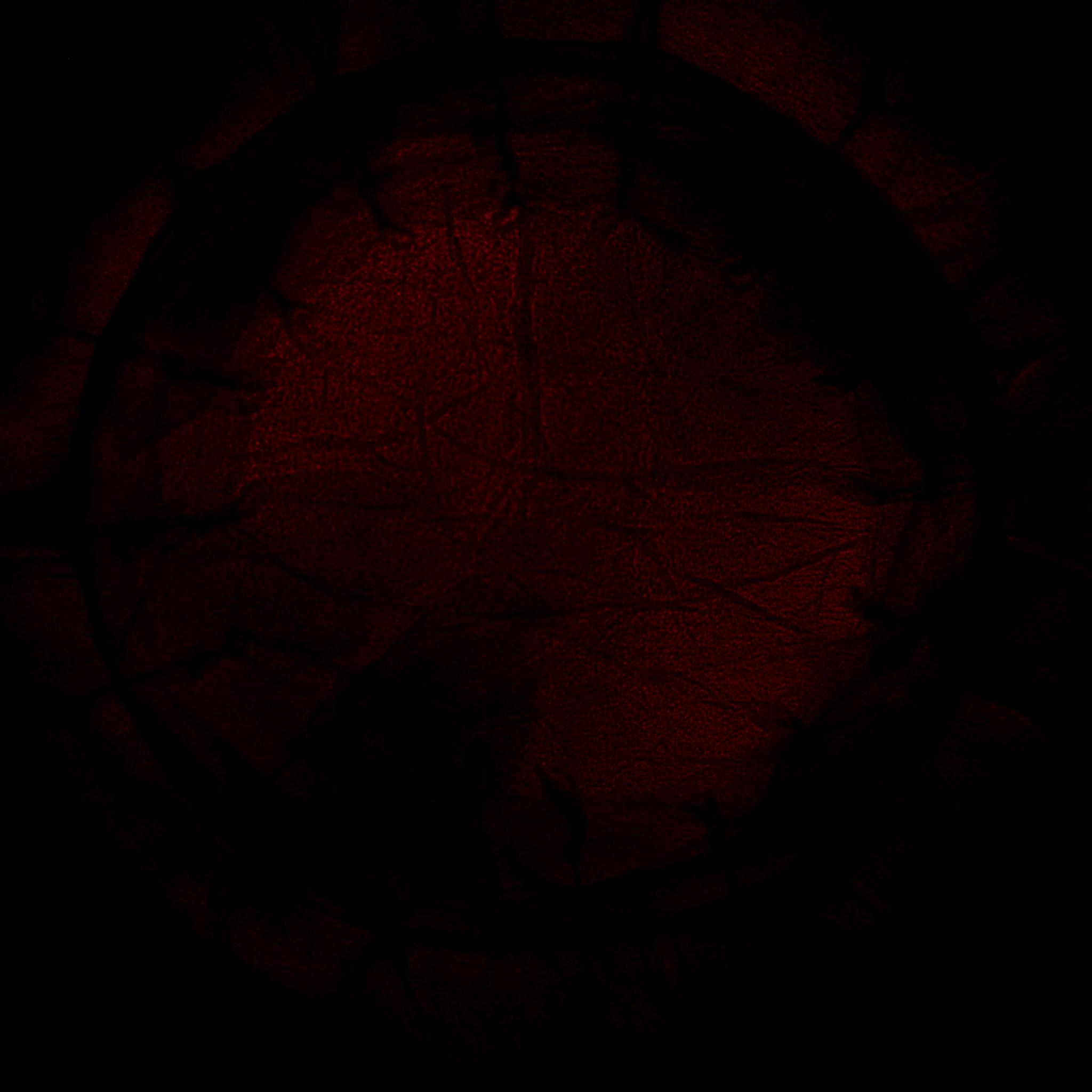

Supplement: S1 File — (ZIP) [file pone.0308204.s001.zip › S1 file. Birefringence Images/A-PK/60 degee/2693OS/IW9.jpg]

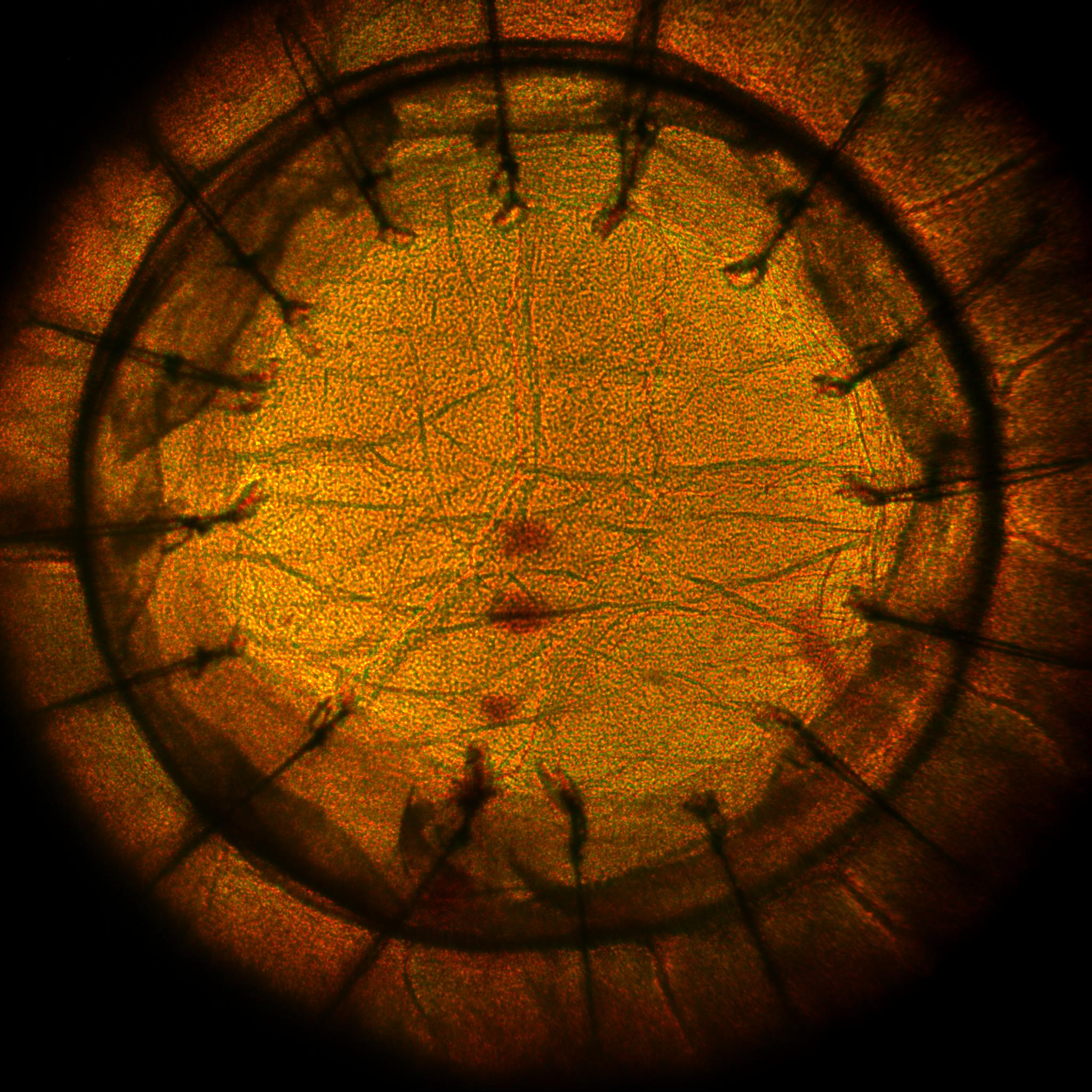

Supplement: S1 File — (ZIP) [file pone.0308204.s001.zip › S1 file. Birefringence Images/A-PK/60 degee/2693OS/sutures.jpg]

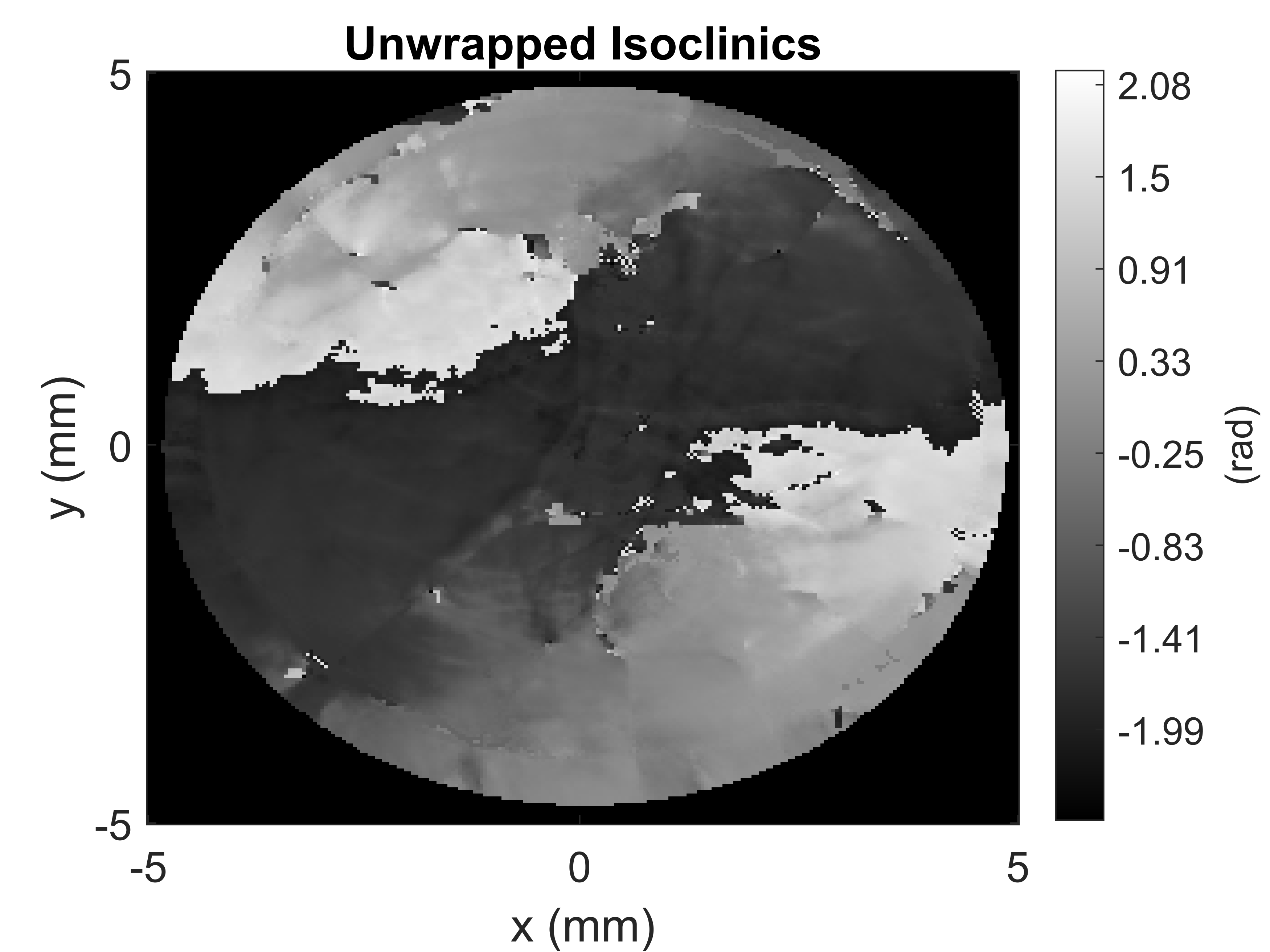

Supplement: S1 File — (ZIP) [file pone.0308204.s001.zip › S1 file. Birefringence Images/A-PK/60 degee/2693OS/unwrappedISOCHgray.tif]

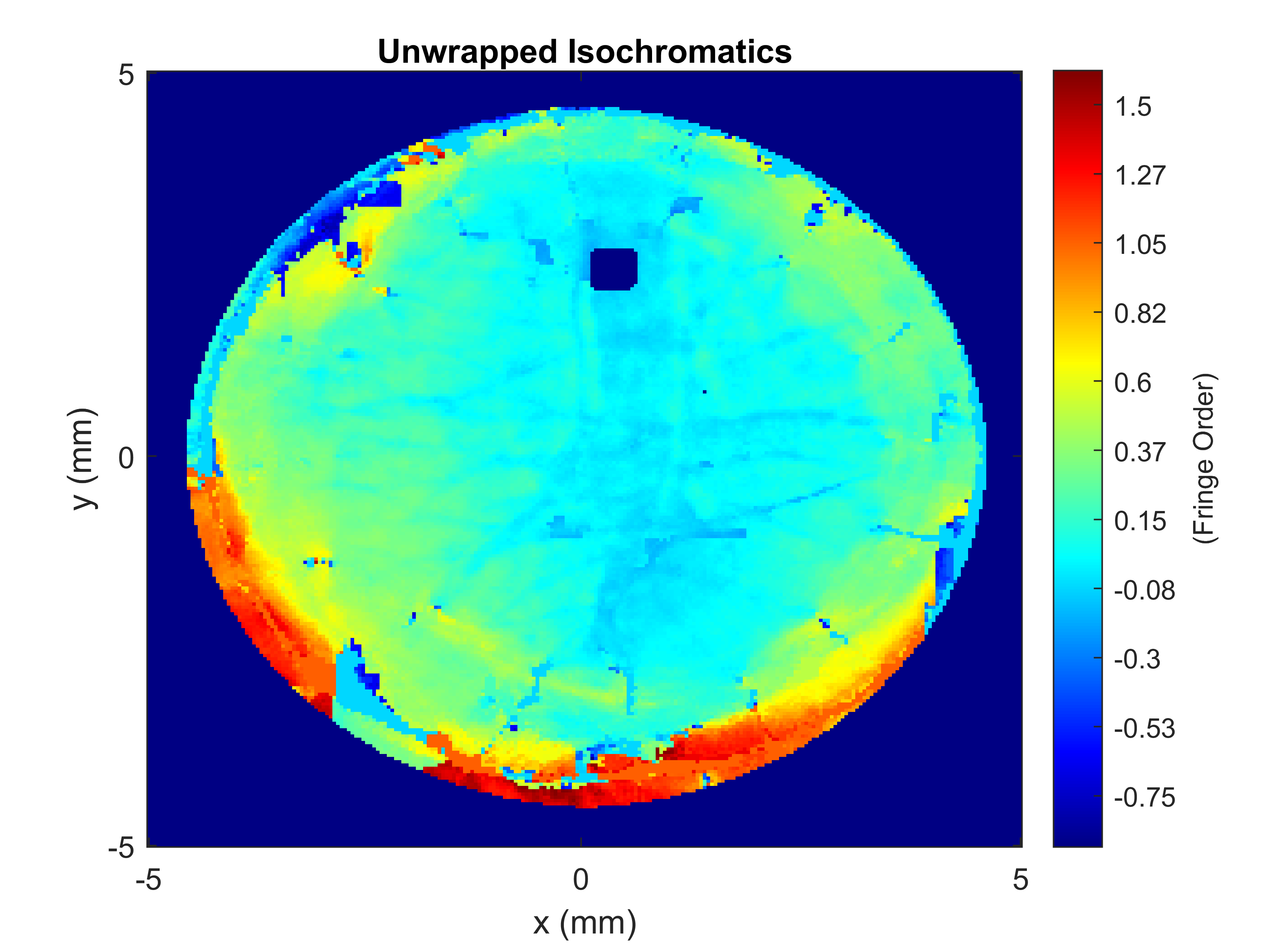

Supplement: S1 File — (ZIP) [file pone.0308204.s001.zip › S1 file. Birefringence Images/A-PK/60 degee/2693OS/unwrappedISOcolor.tif]

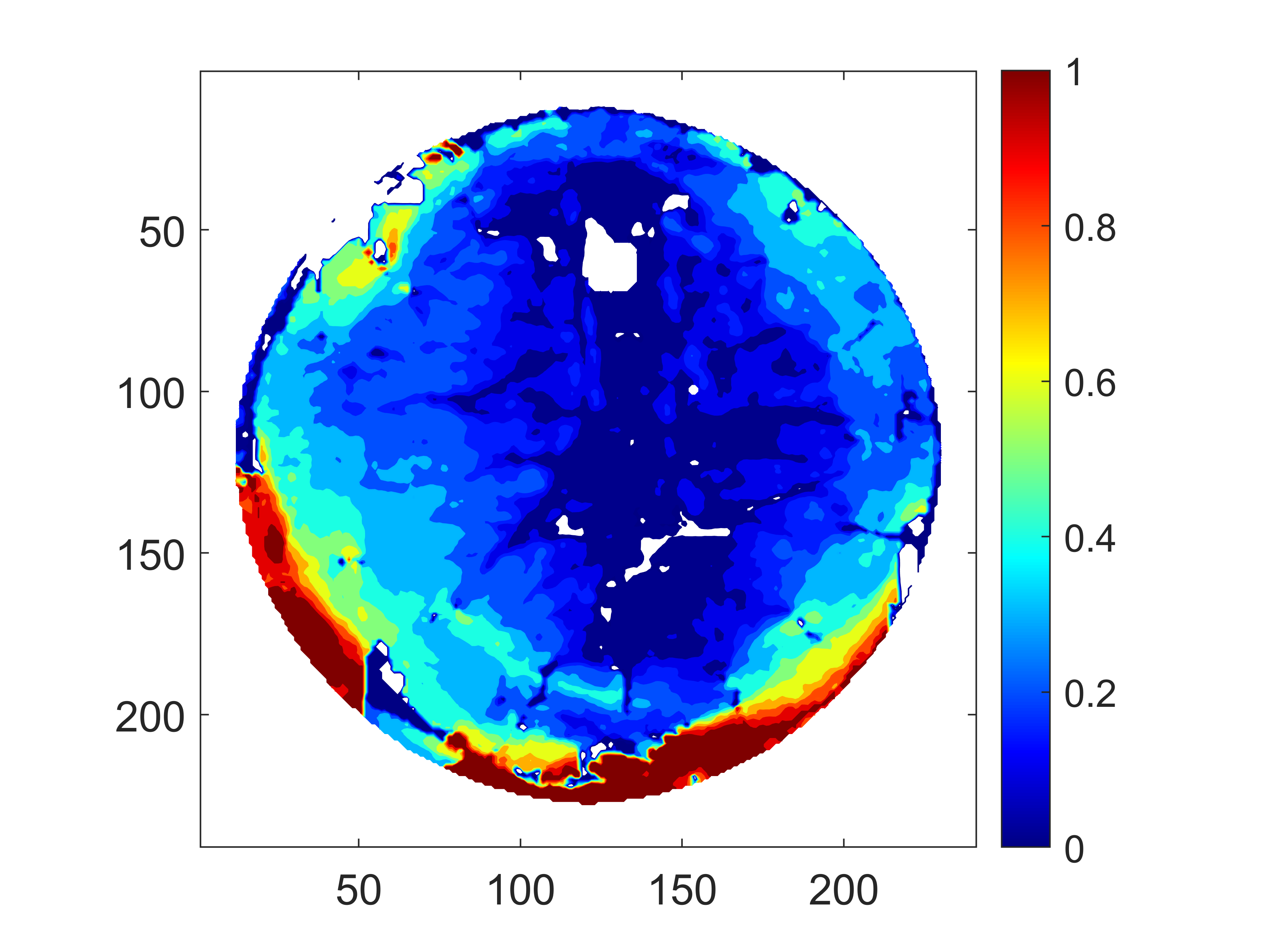

Supplement: S1 File — (ZIP) [file pone.0308204.s001.zip › S1 file. Birefringence Images/A-PK/60 degee/2693OS/unwrappedISOconour.tif]

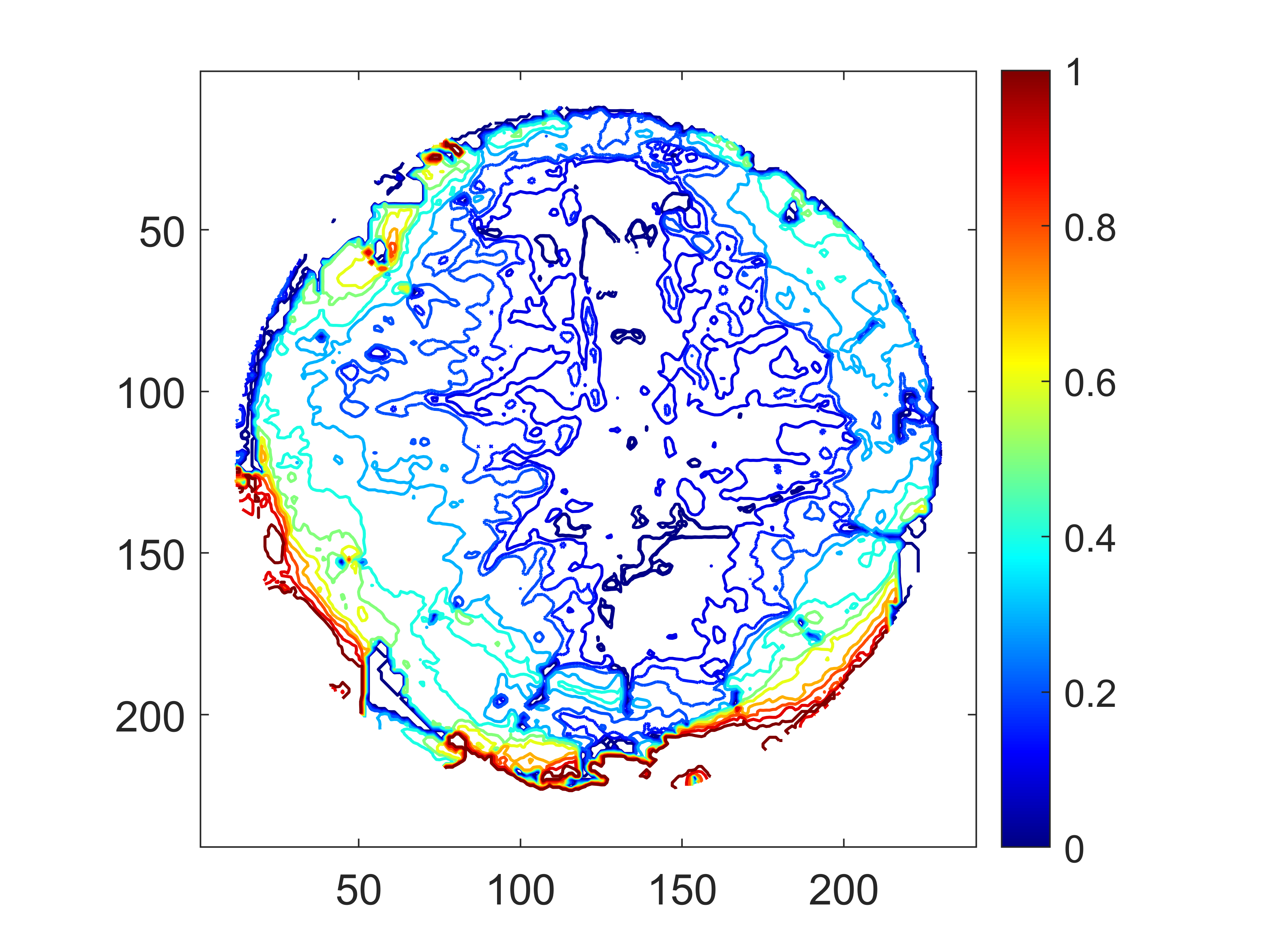

Supplement: S1 File — (ZIP) [file pone.0308204.s001.zip › S1 file. Birefringence Images/A-PK/60 degee/2693OS/unwrappedISOcons.tif]

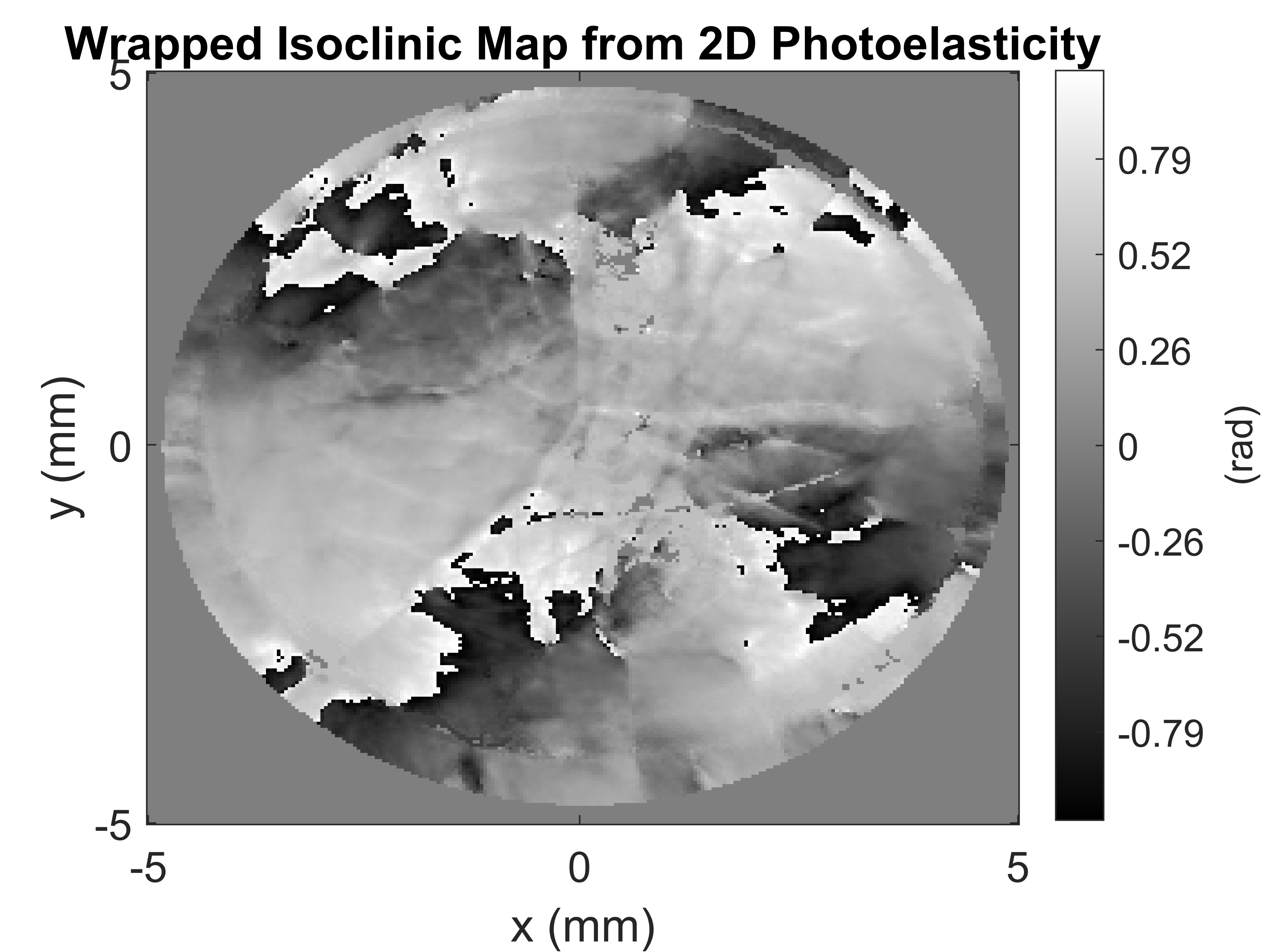

Supplement: S1 File — (ZIP) [file pone.0308204.s001.zip › S1 file. Birefringence Images/A-PK/60 degee/2693OS/wrappedISOgray.tif]

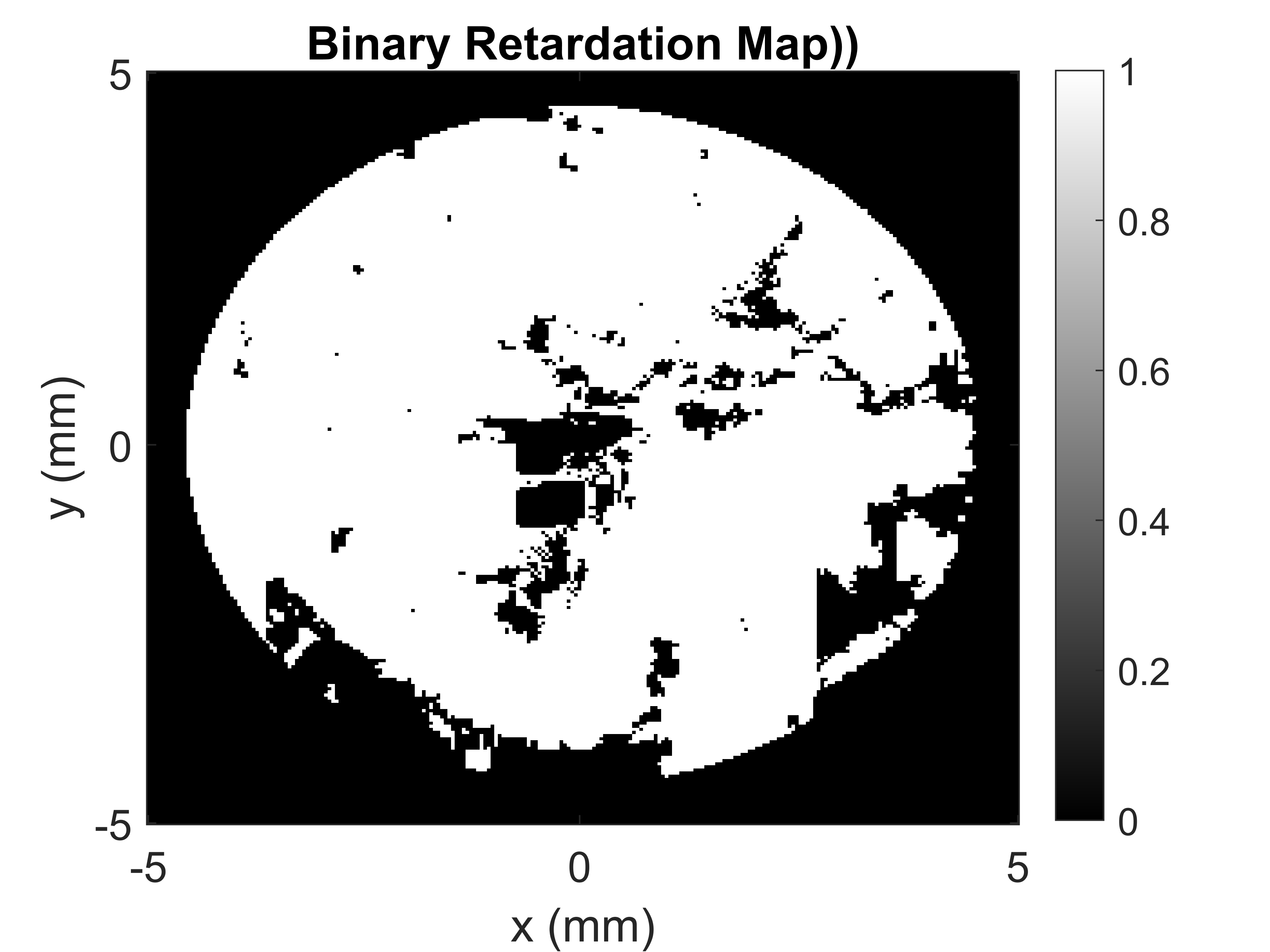

Supplement: S1 File — (ZIP) [file pone.0308204.s001.zip › S1 file. Birefringence Images/A-PK/90 degee/2845OD/isoopicpoins.tif]

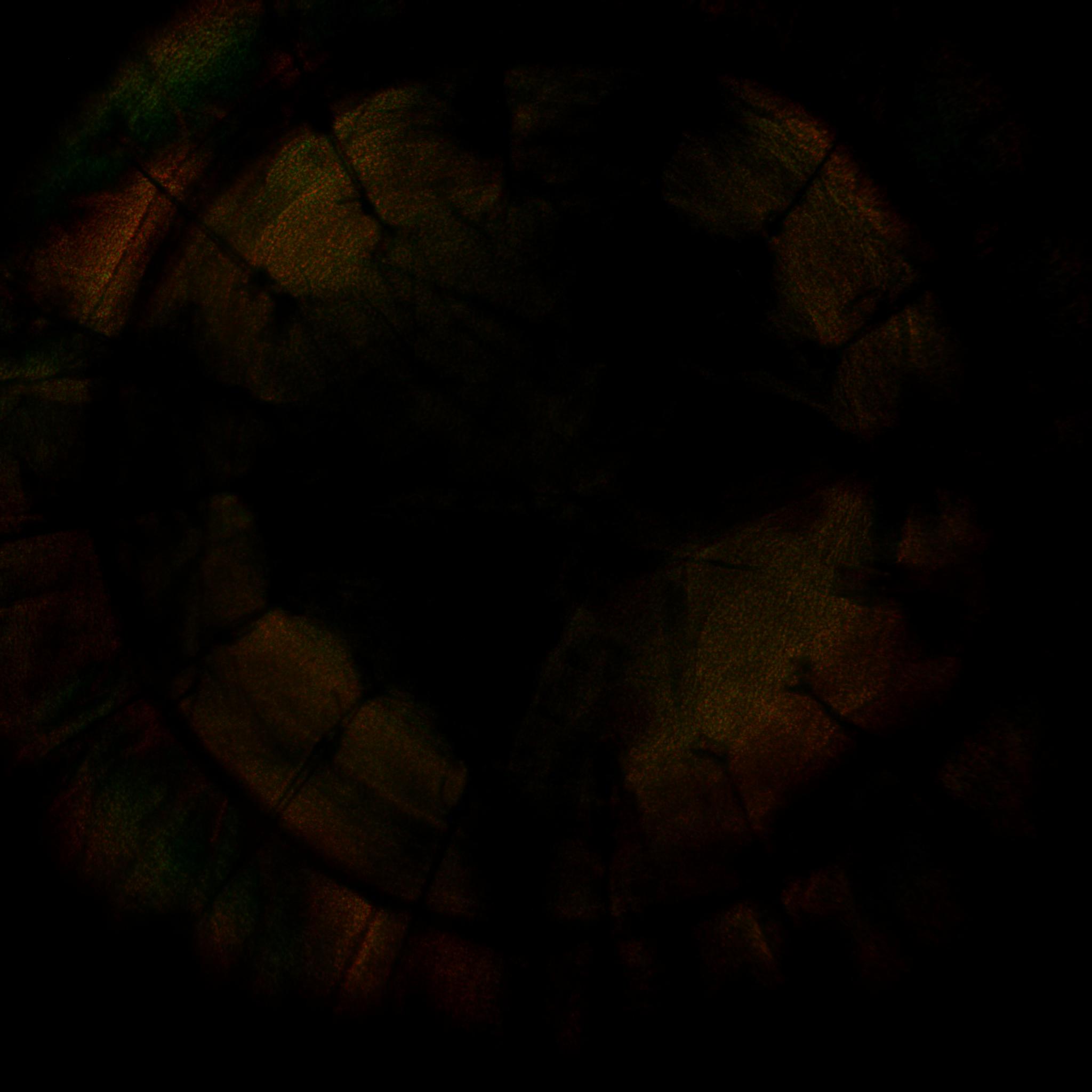

Supplement: S1 File — (ZIP) [file pone.0308204.s001.zip › S1 file. Birefringence Images/A-PK/90 degee/2845OD/IW1.jpg]

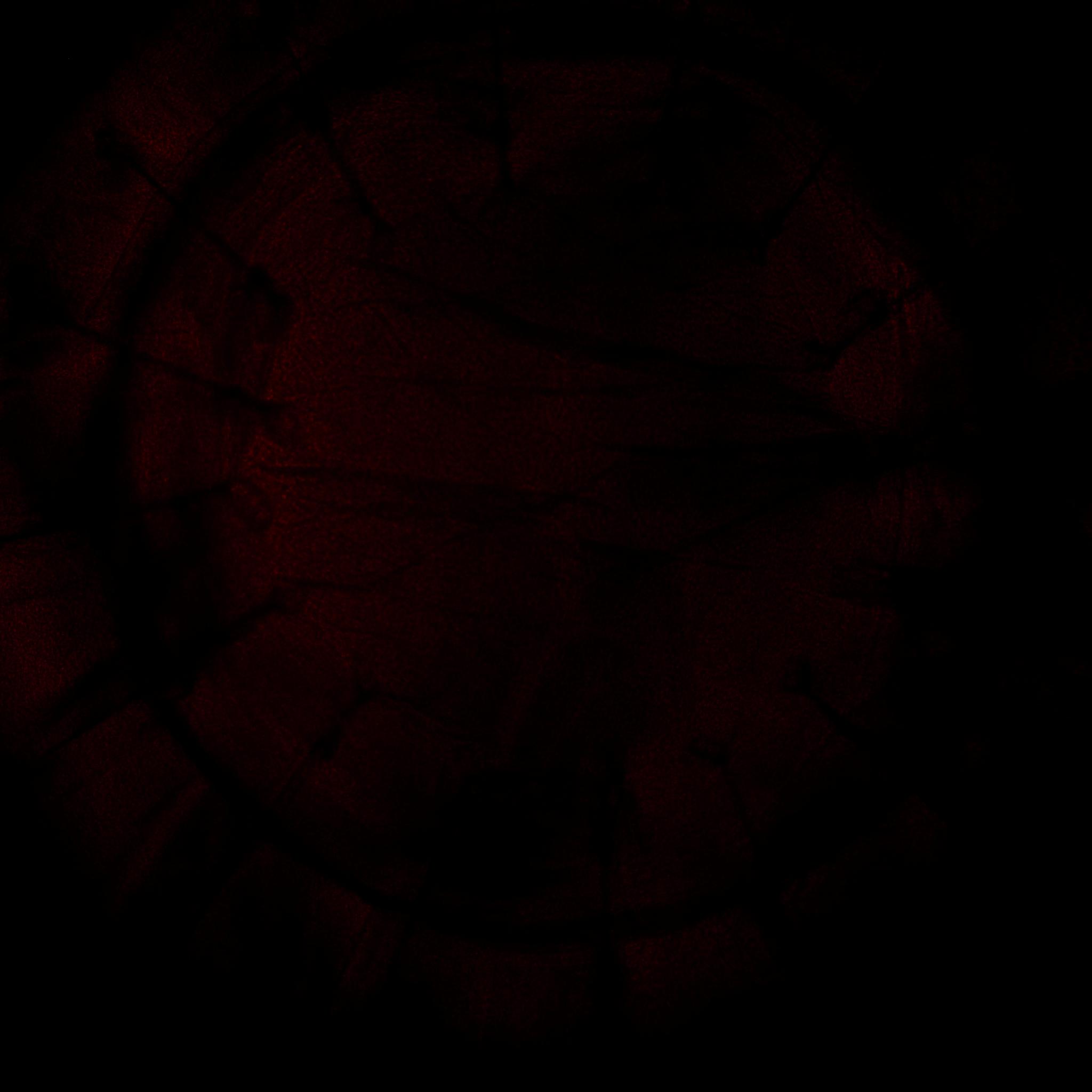

Supplement: S1 File — (ZIP) [file pone.0308204.s001.zip › S1 file. Birefringence Images/A-PK/90 degee/2845OD/IW10.jpg]

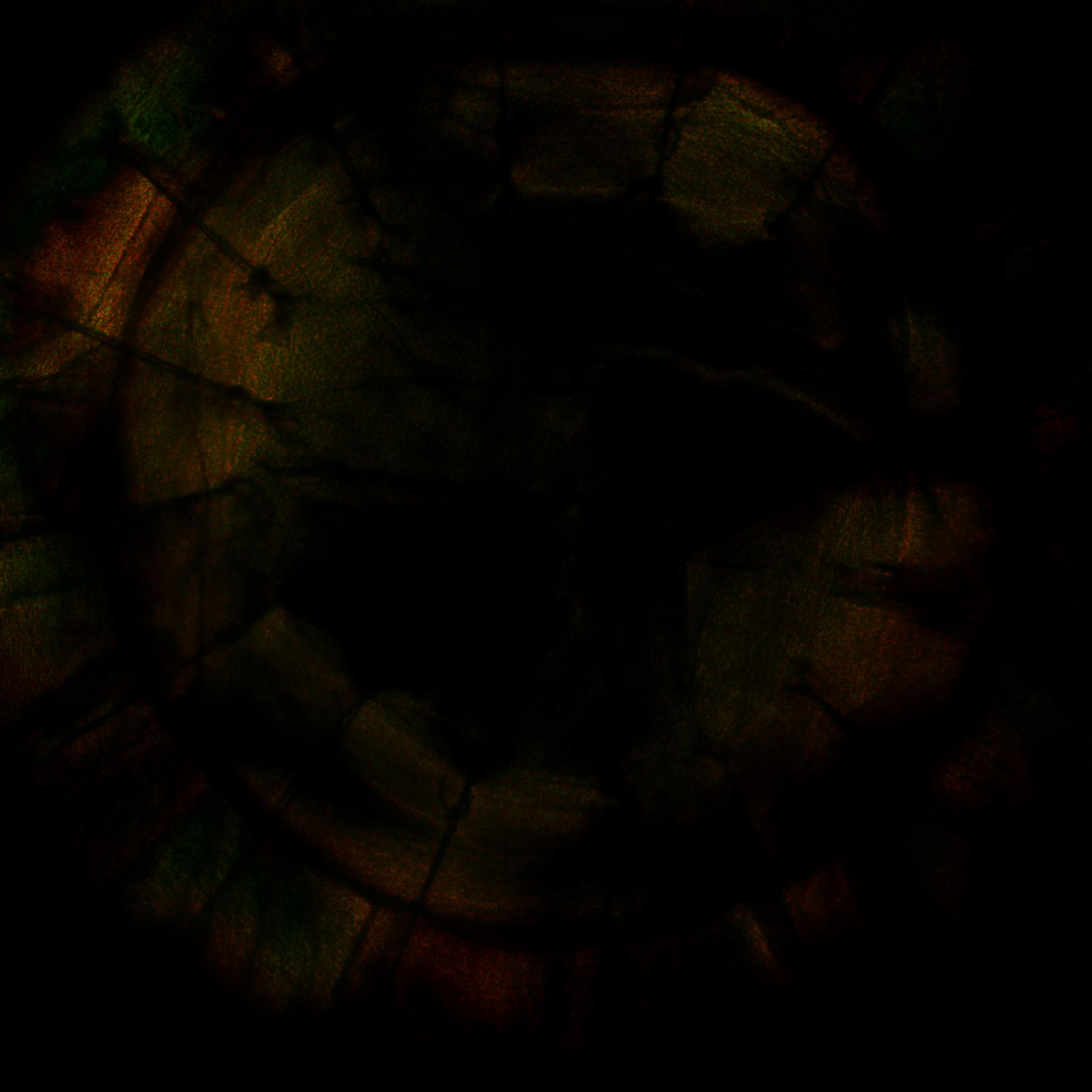

Supplement: S1 File — (ZIP) [file pone.0308204.s001.zip › S1 file. Birefringence Images/A-PK/90 degee/2845OD/IW2.jpg]

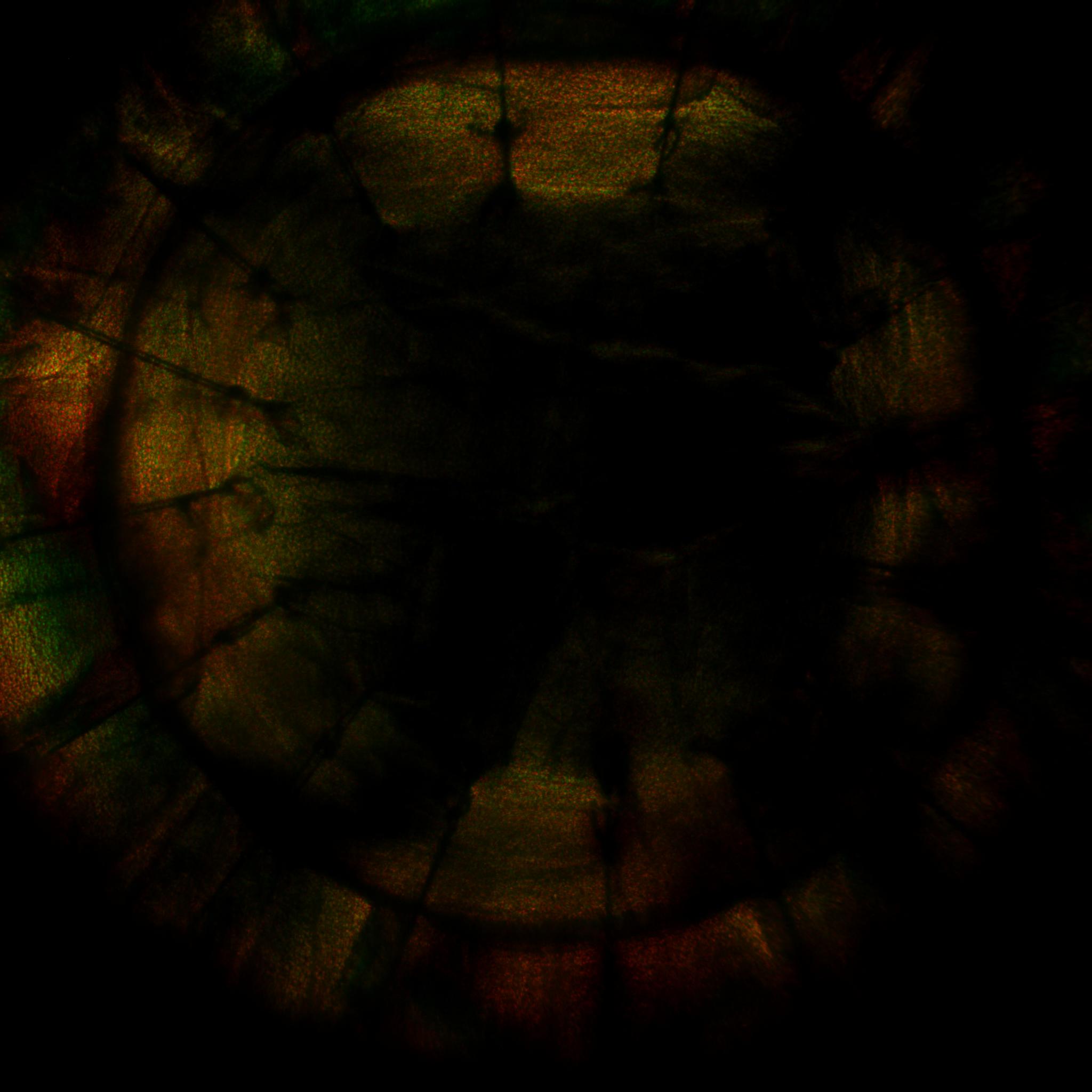

Supplement: S1 File — (ZIP) [file pone.0308204.s001.zip › S1 file. Birefringence Images/A-PK/90 degee/2845OD/IW3.jpg]

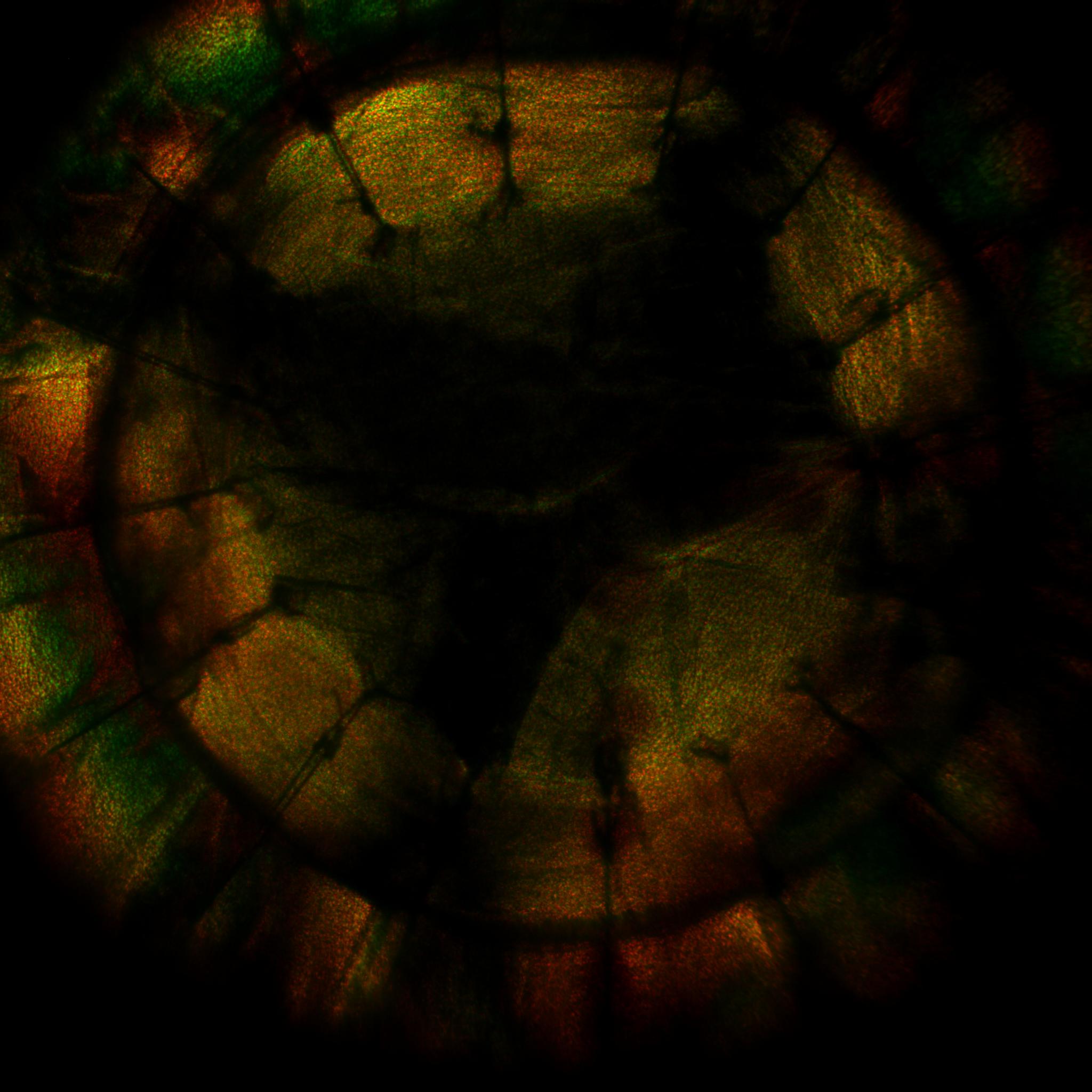

Supplement: S1 File — (ZIP) [file pone.0308204.s001.zip › S1 file. Birefringence Images/A-PK/90 degee/2845OD/IW4.jpg]

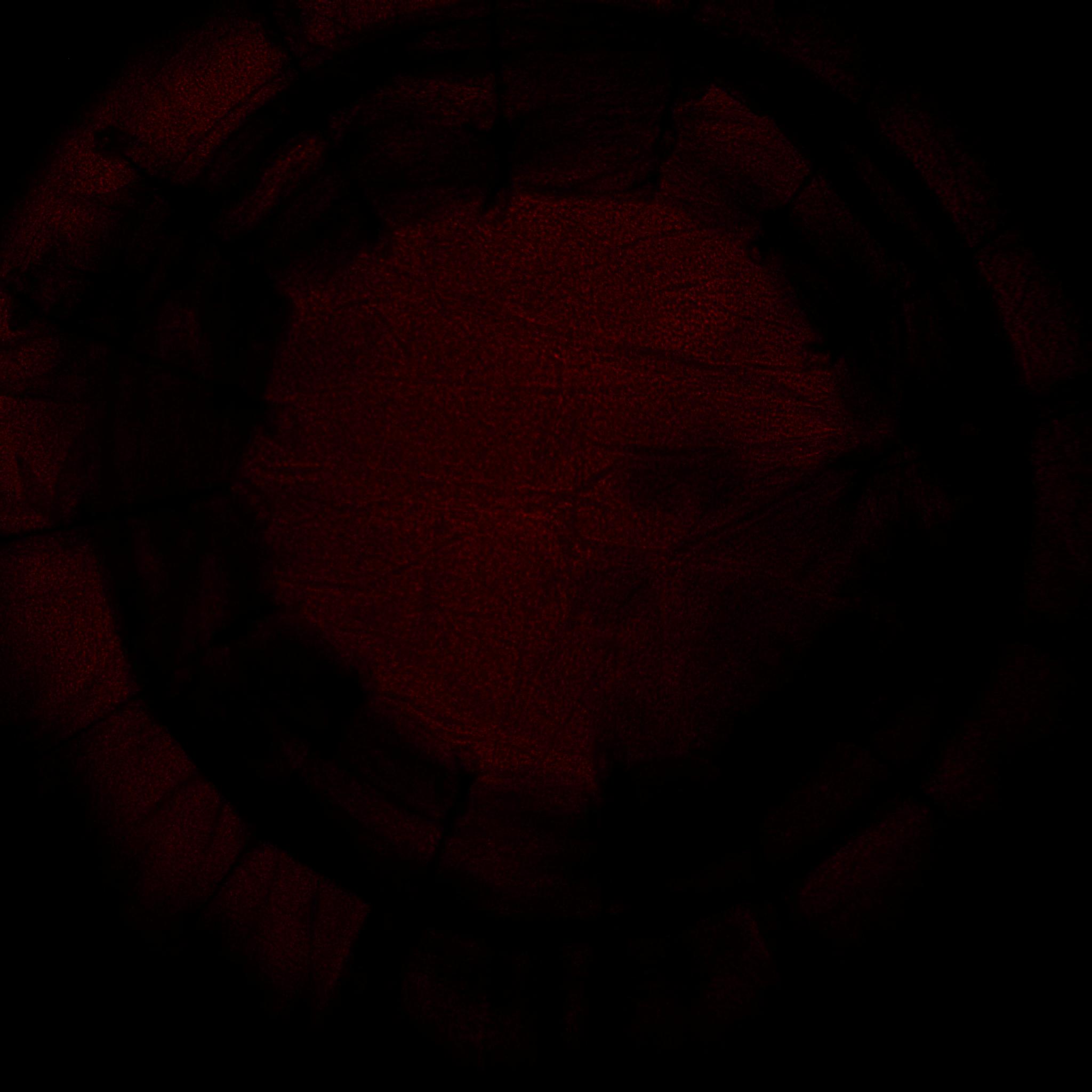

Supplement: S1 File — (ZIP) [file pone.0308204.s001.zip › S1 file. Birefringence Images/A-PK/90 degee/2845OD/IW5.jpg]

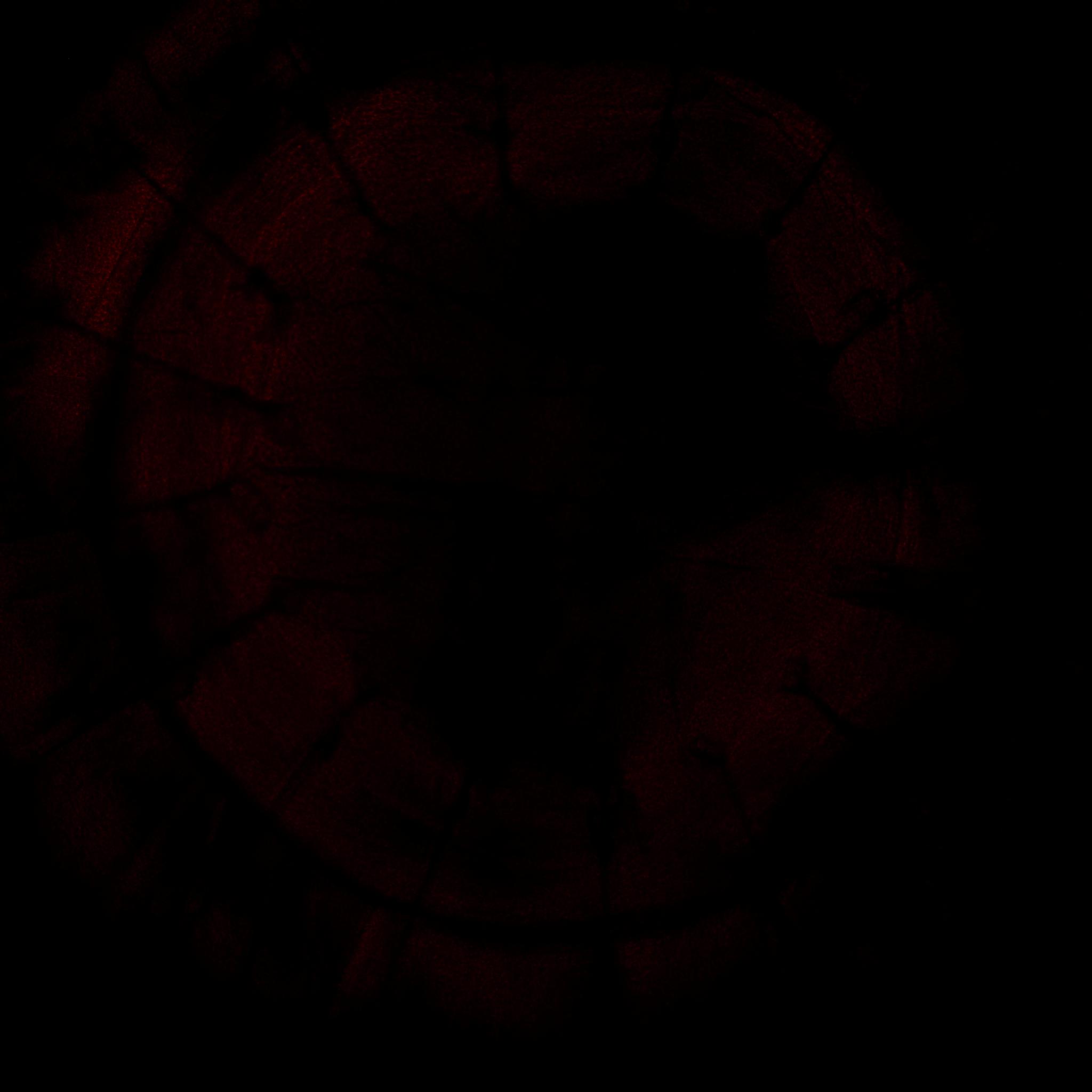

Supplement: S1 File — (ZIP) [file pone.0308204.s001.zip › S1 file. Birefringence Images/A-PK/90 degee/2845OD/IW6.jpg]

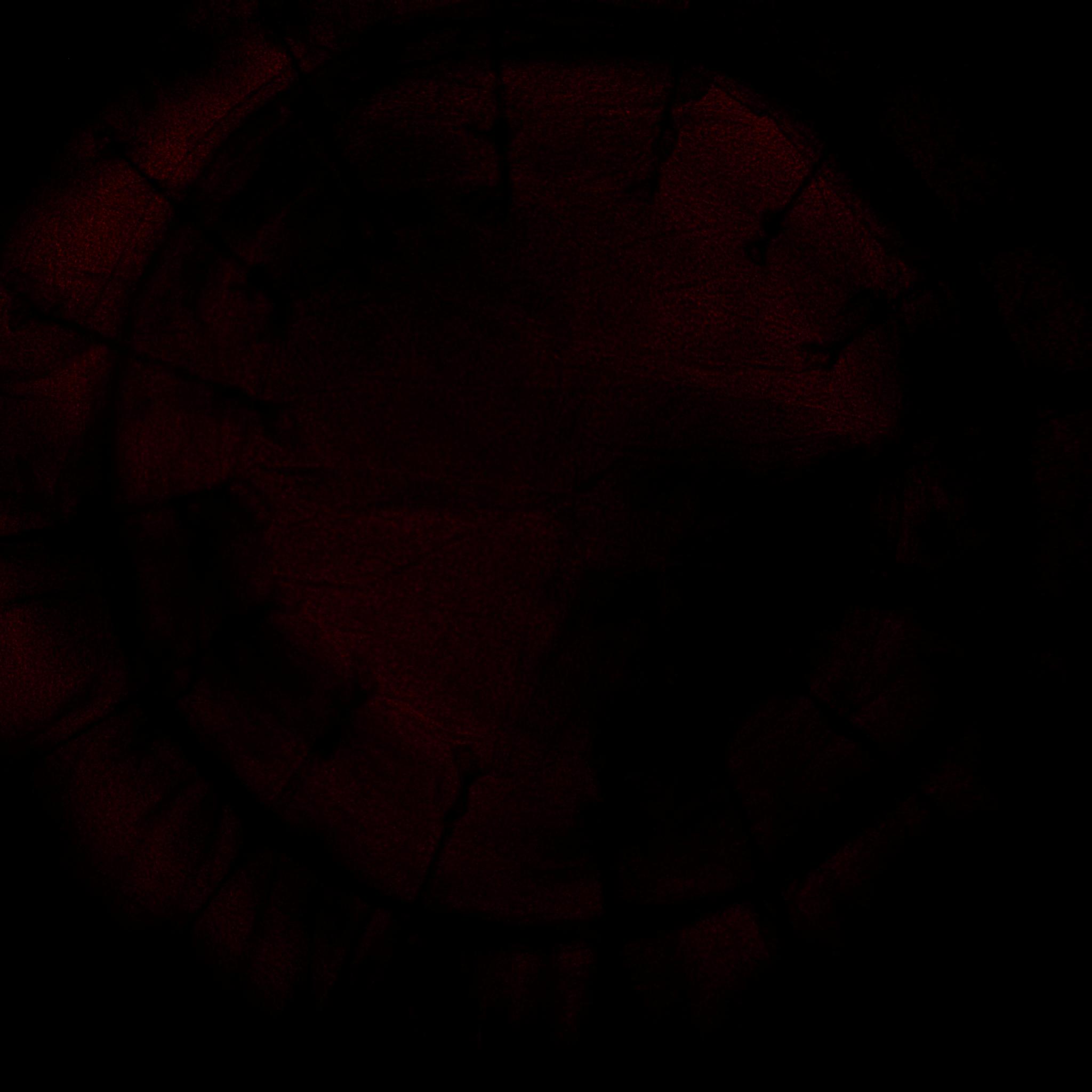

Supplement: S1 File — (ZIP) [file pone.0308204.s001.zip › S1 file. Birefringence Images/A-PK/90 degee/2845OD/IW7.jpg]

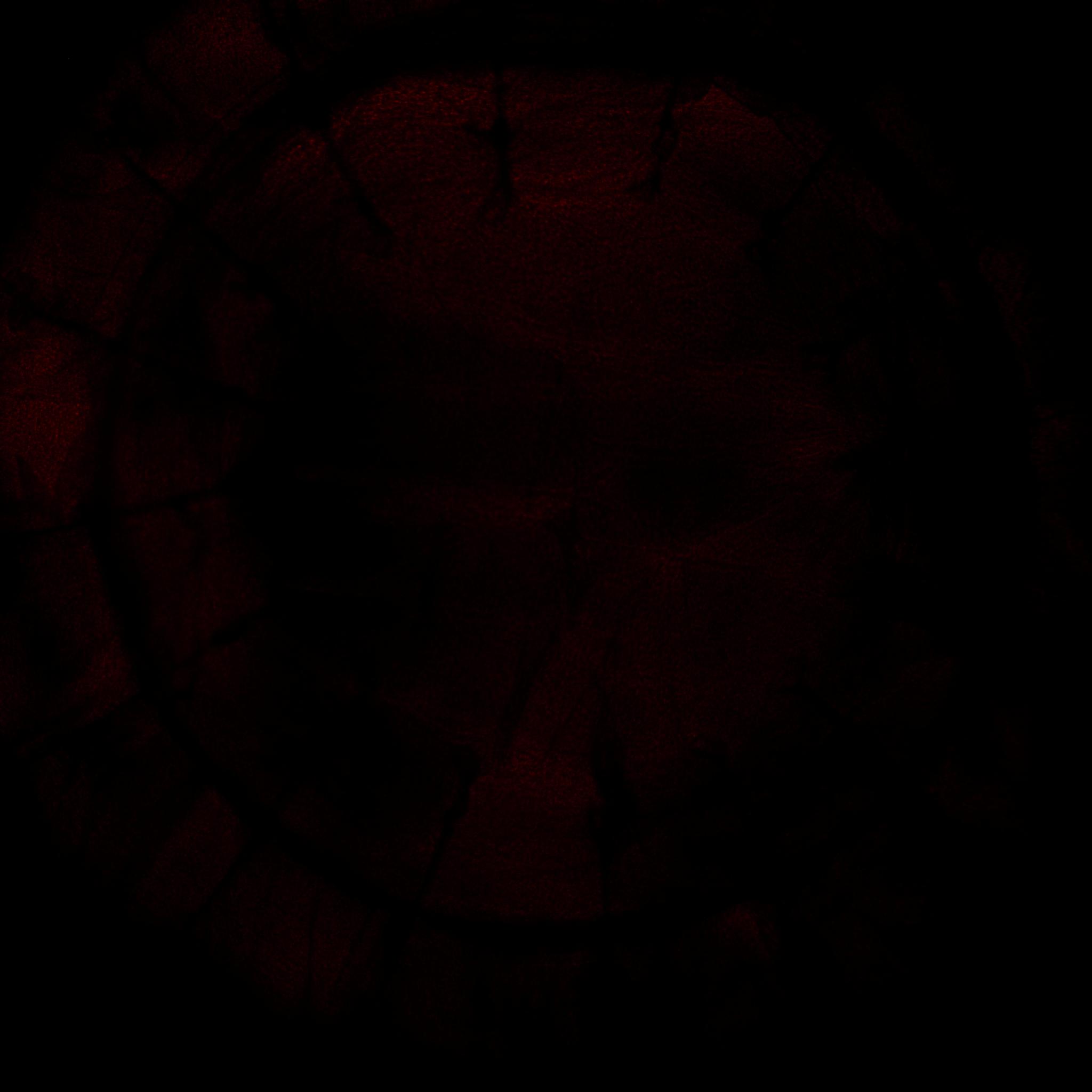

Supplement: S1 File — (ZIP) [file pone.0308204.s001.zip › S1 file. Birefringence Images/A-PK/90 degee/2845OD/IW8.jpg]

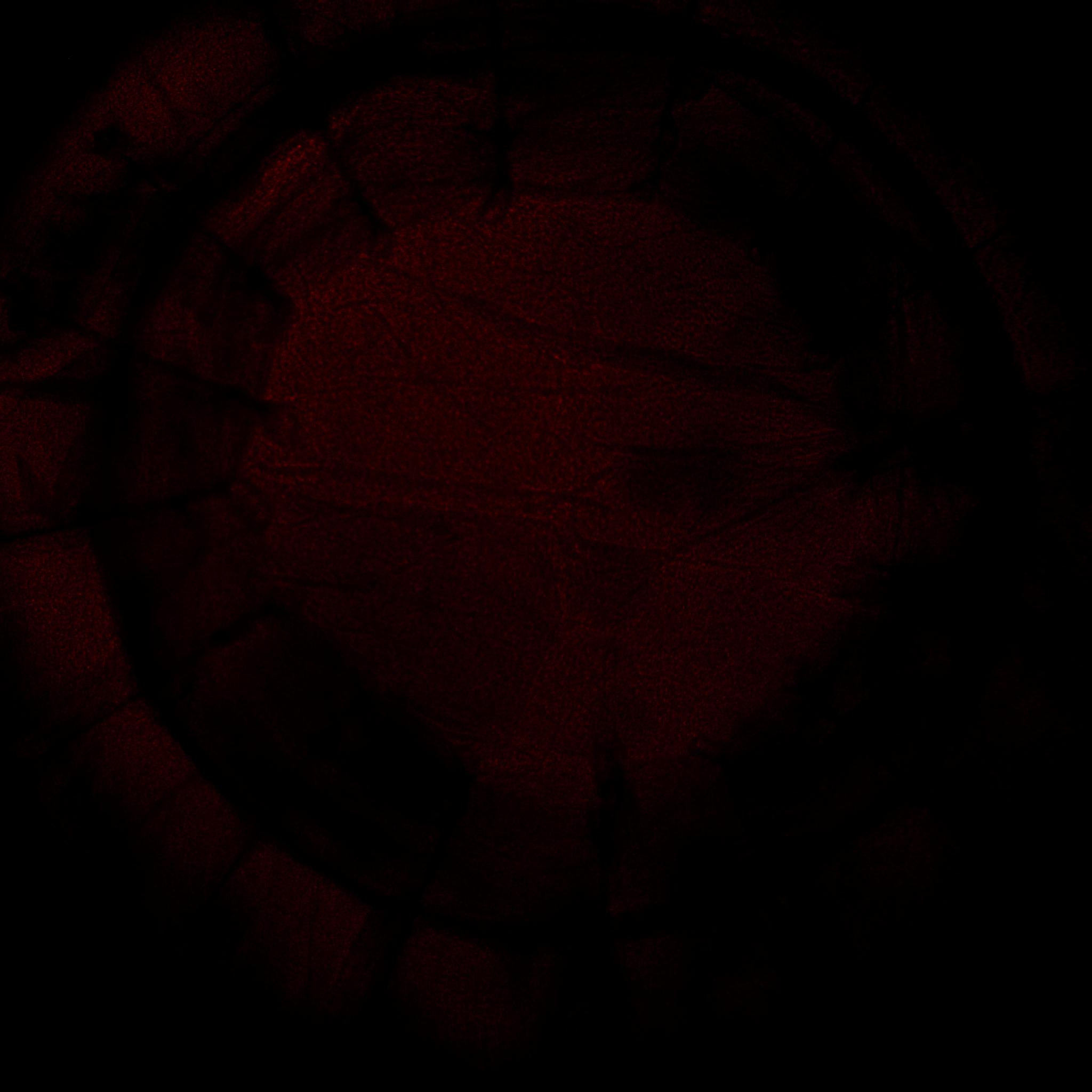

Supplement: S1 File — (ZIP) [file pone.0308204.s001.zip › S1 file. Birefringence Images/A-PK/90 degee/2845OD/IW9.jpg]

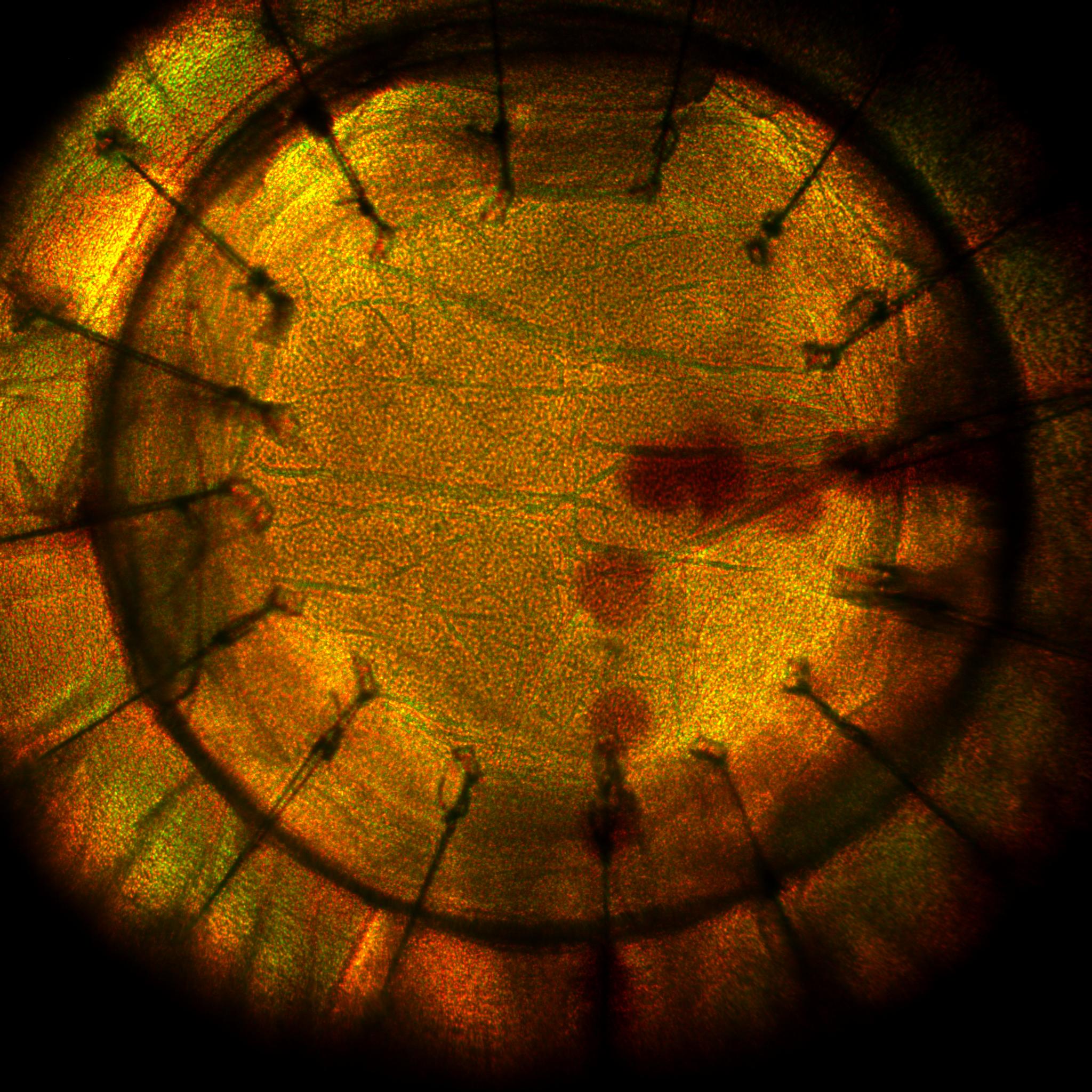

Supplement: S1 File — (ZIP) [file pone.0308204.s001.zip › S1 file. Birefringence Images/A-PK/90 degee/2845OD/suues.jpg]

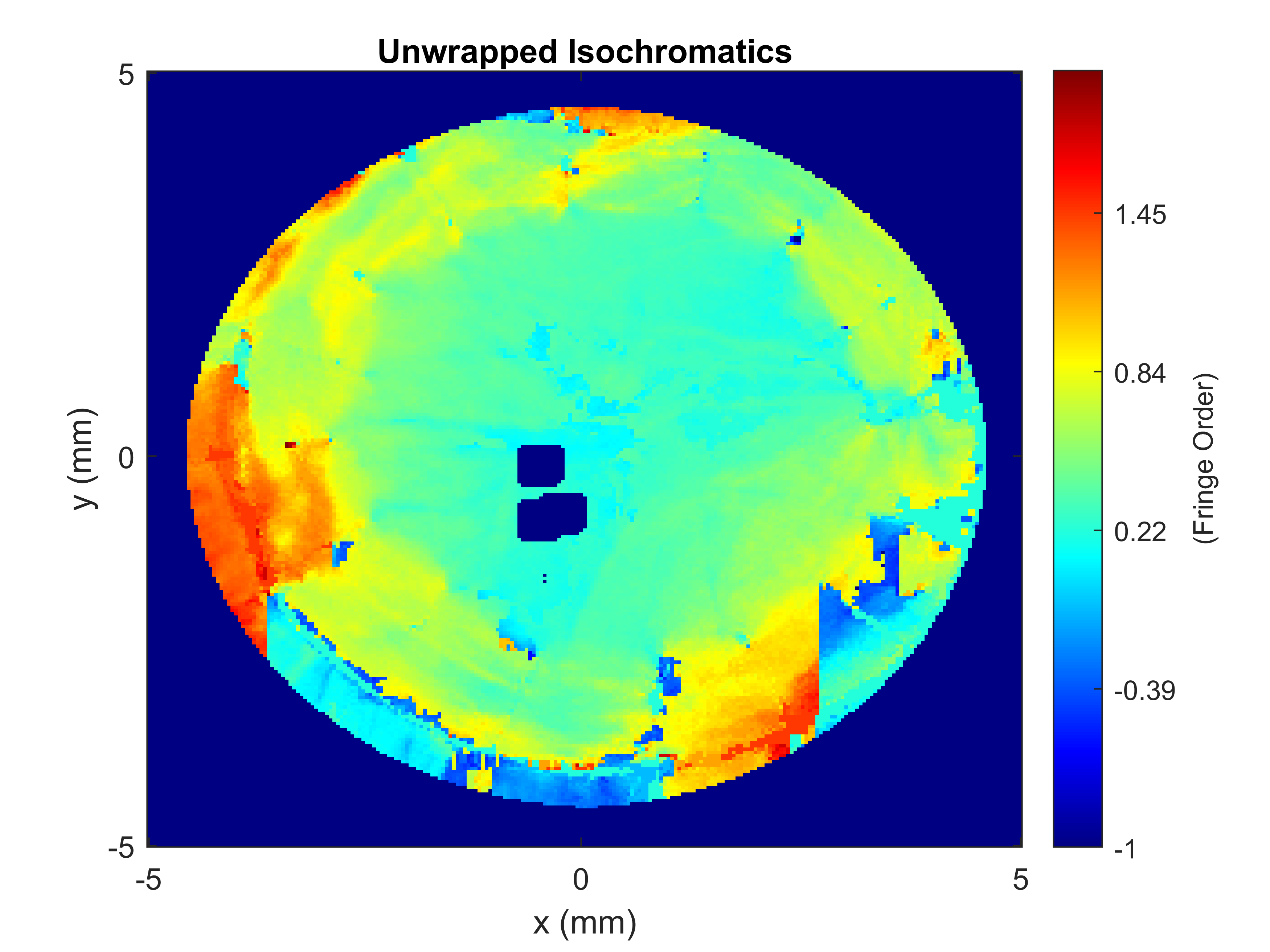

Supplement: S1 File — (ZIP) [file pone.0308204.s001.zip › S1 file. Birefringence Images/A-PK/90 degee/2845OD/unwappedISOCHcolo.tif]

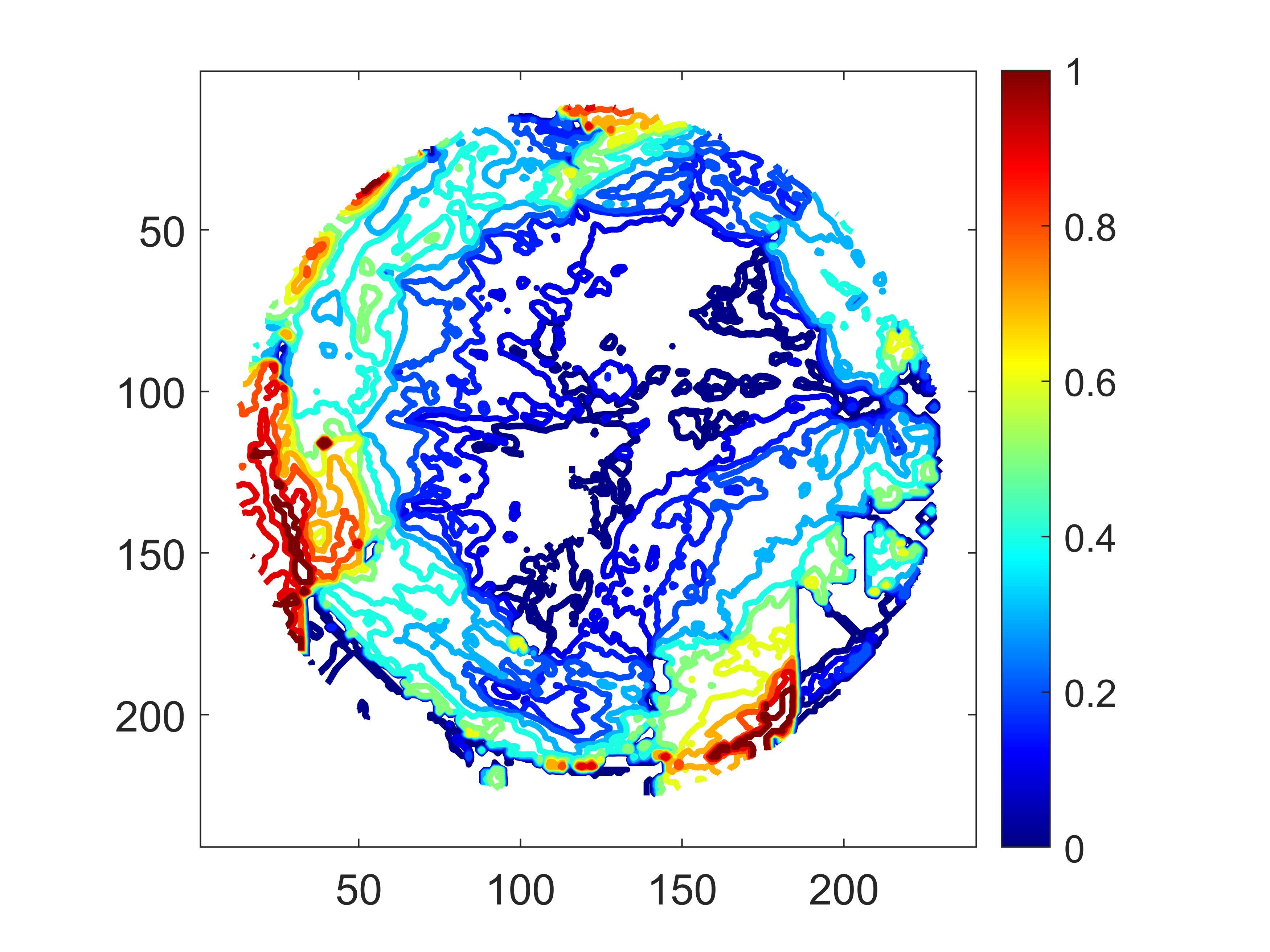

Supplement: S1 File — (ZIP) [file pone.0308204.s001.zip › S1 file. Birefringence Images/A-PK/90 degee/2845OD/unwappedISOCHconou.tif]

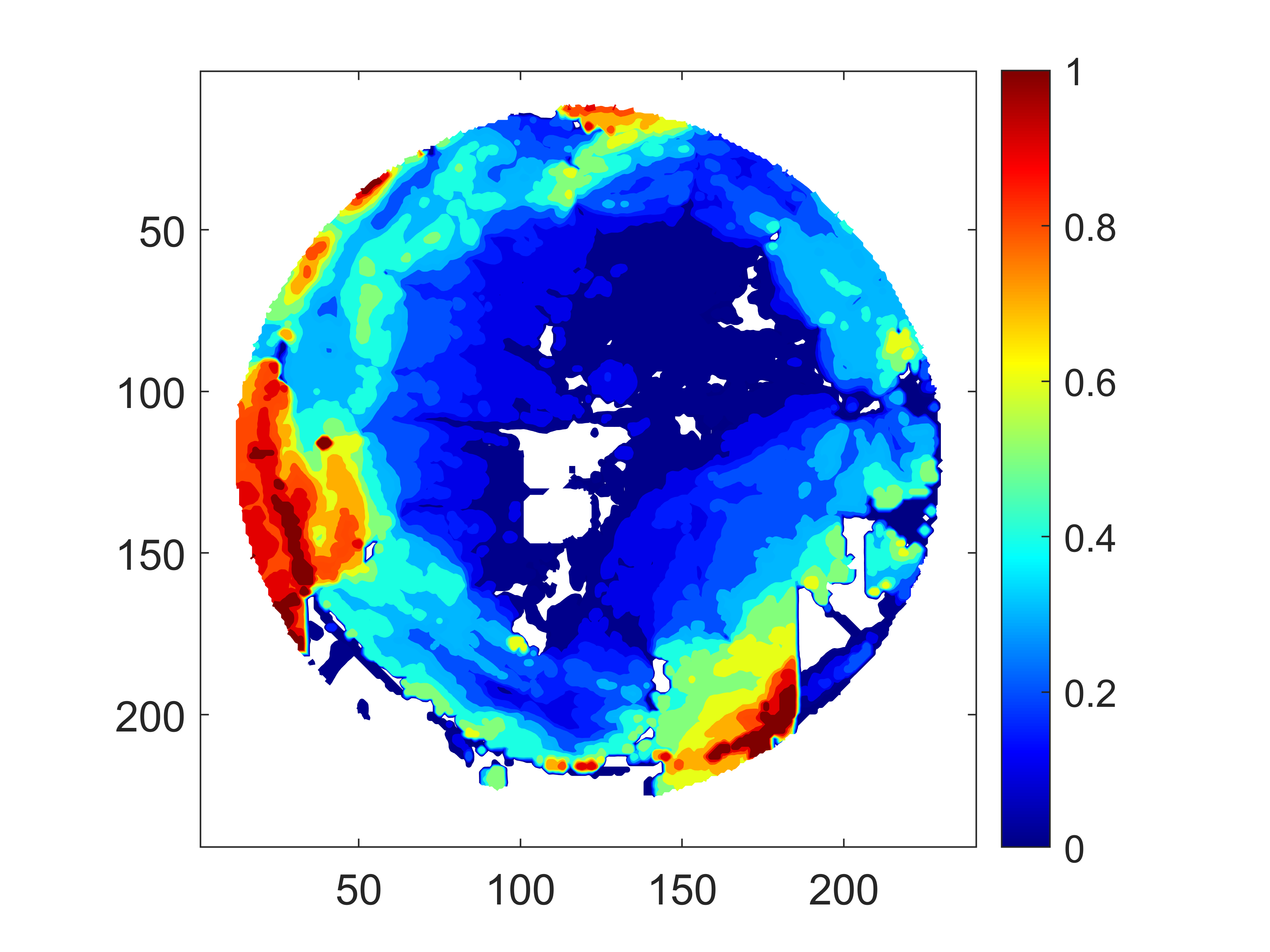

Supplement: S1 File — (ZIP) [file pone.0308204.s001.zip › S1 file. Birefringence Images/A-PK/90 degee/2845OD/unwappedISOCHfilled.tif]

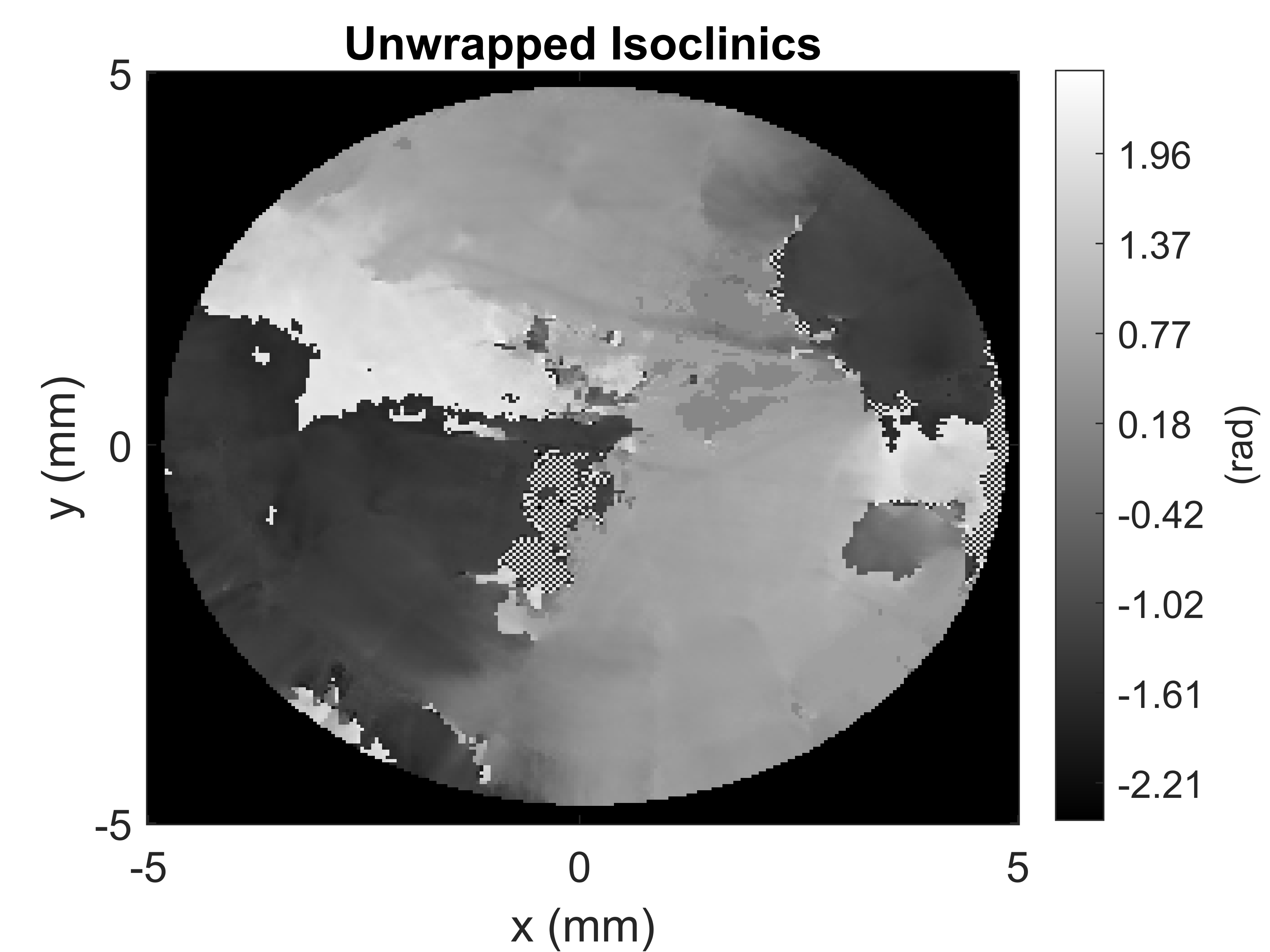

Supplement: S1 File — (ZIP) [file pone.0308204.s001.zip › S1 file. Birefringence Images/A-PK/90 degee/2845OD/unwappedISOCHgay.tif]

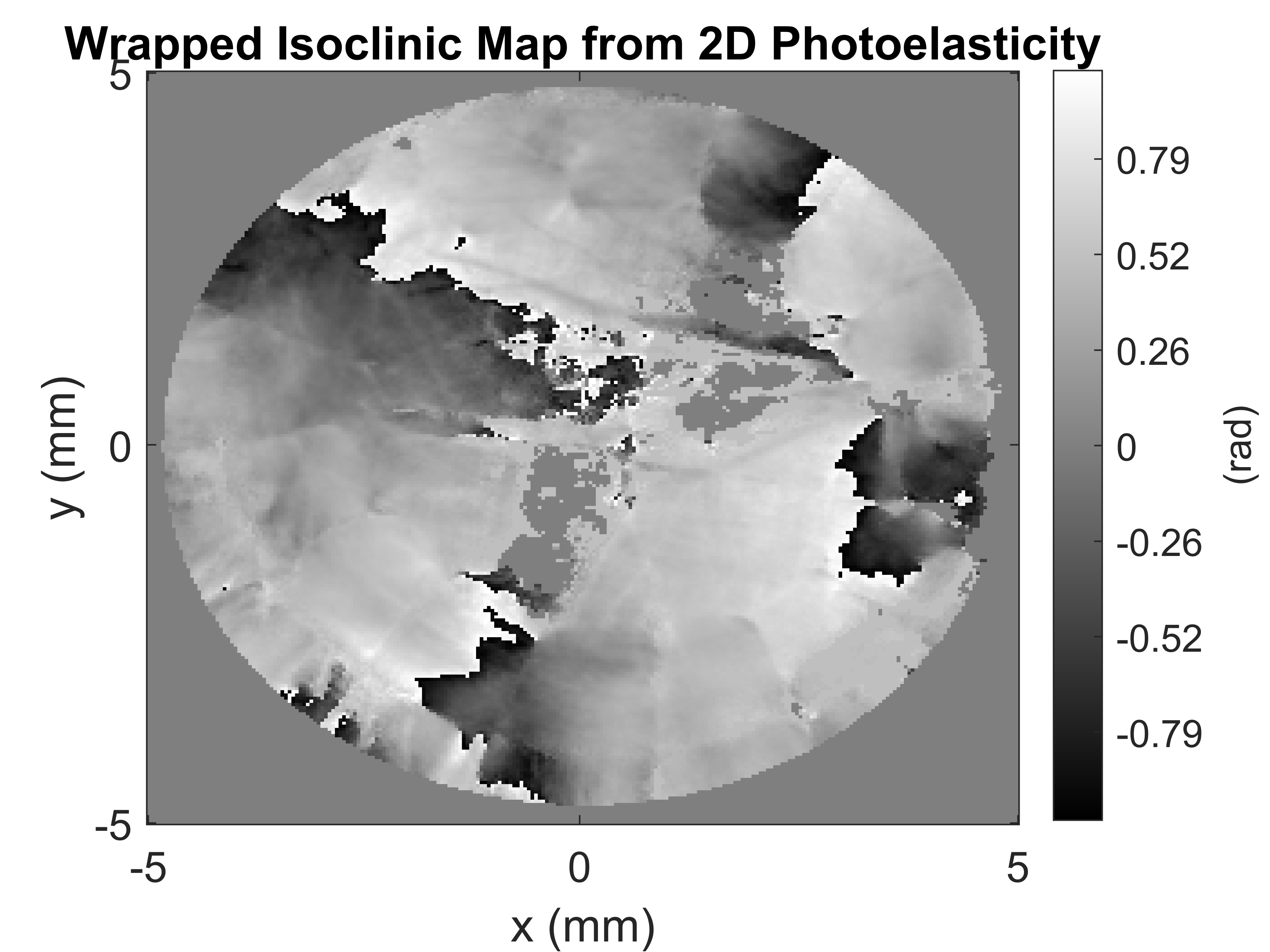

Supplement: S1 File — (ZIP) [file pone.0308204.s001.zip › S1 file. Birefringence Images/A-PK/90 degee/2845OD/wappedISOgay.tif]

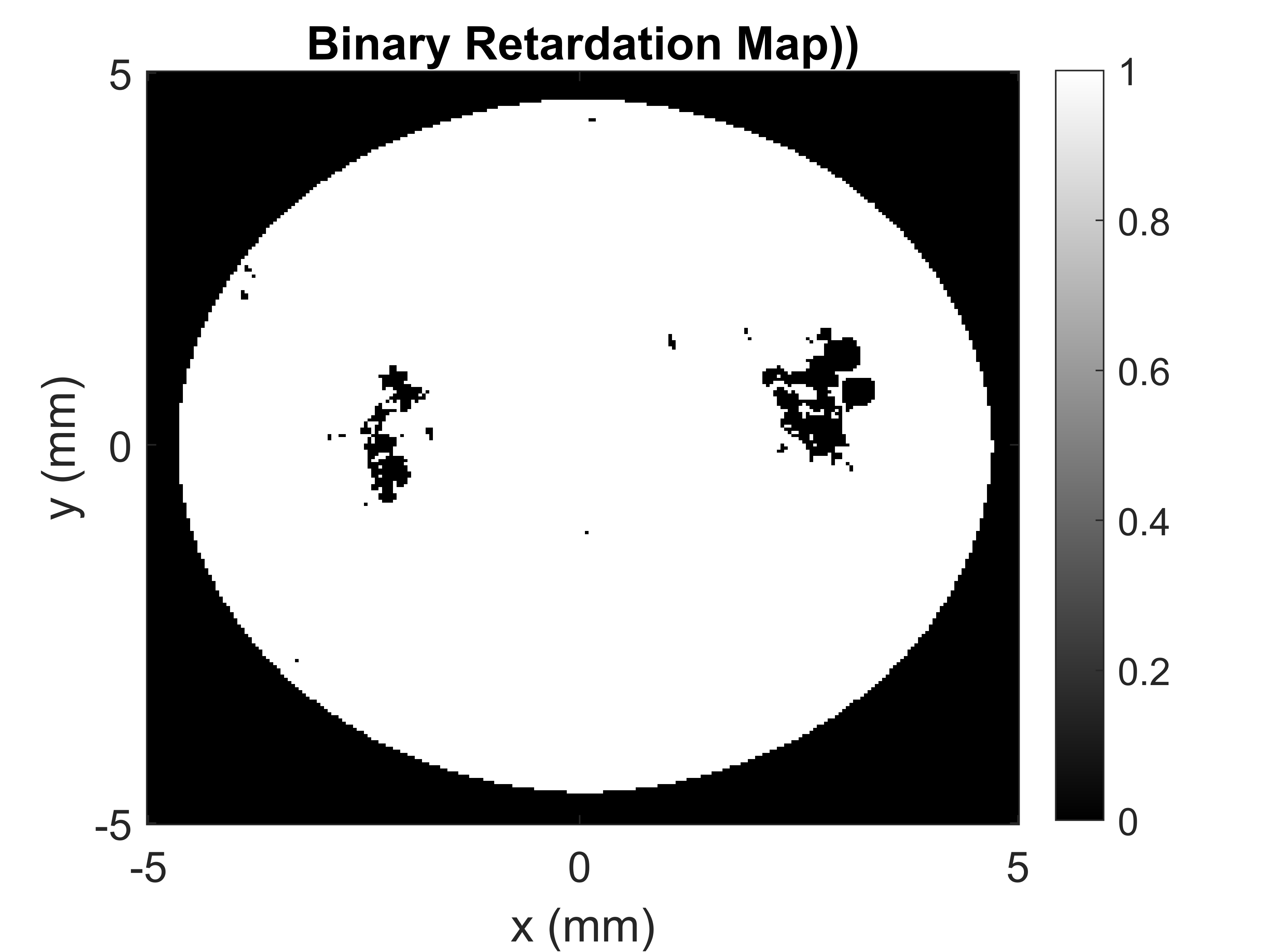

Supplement: S1 File — (ZIP) [file pone.0308204.s001.zip › S1 file. Birefringence Images/B-PK/0 degee/2349OD/ISOOPIC.tif]

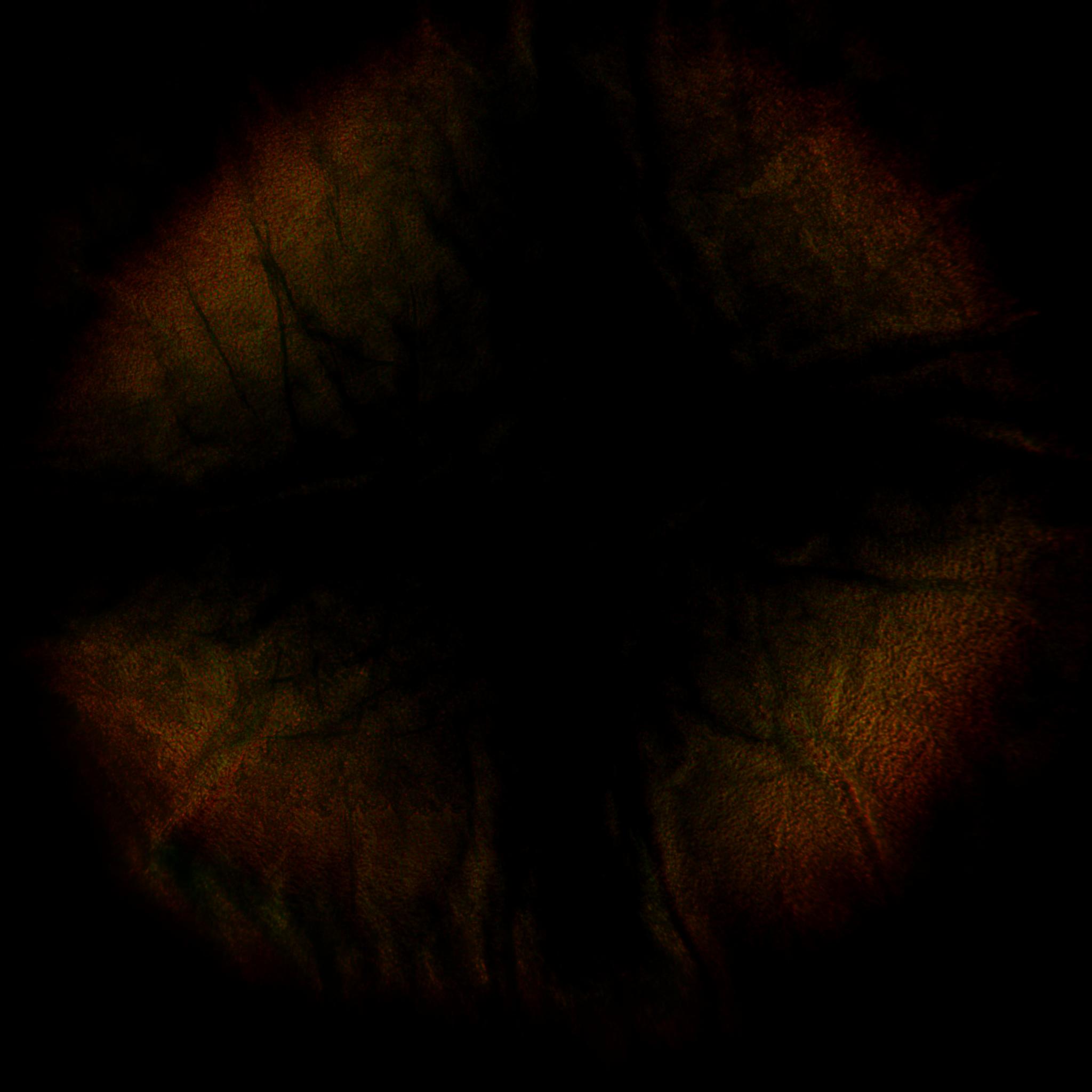

Supplement: S1 File — (ZIP) [file pone.0308204.s001.zip › S1 file. Birefringence Images/B-PK/0 degee/2349OD/IW1.jpg]

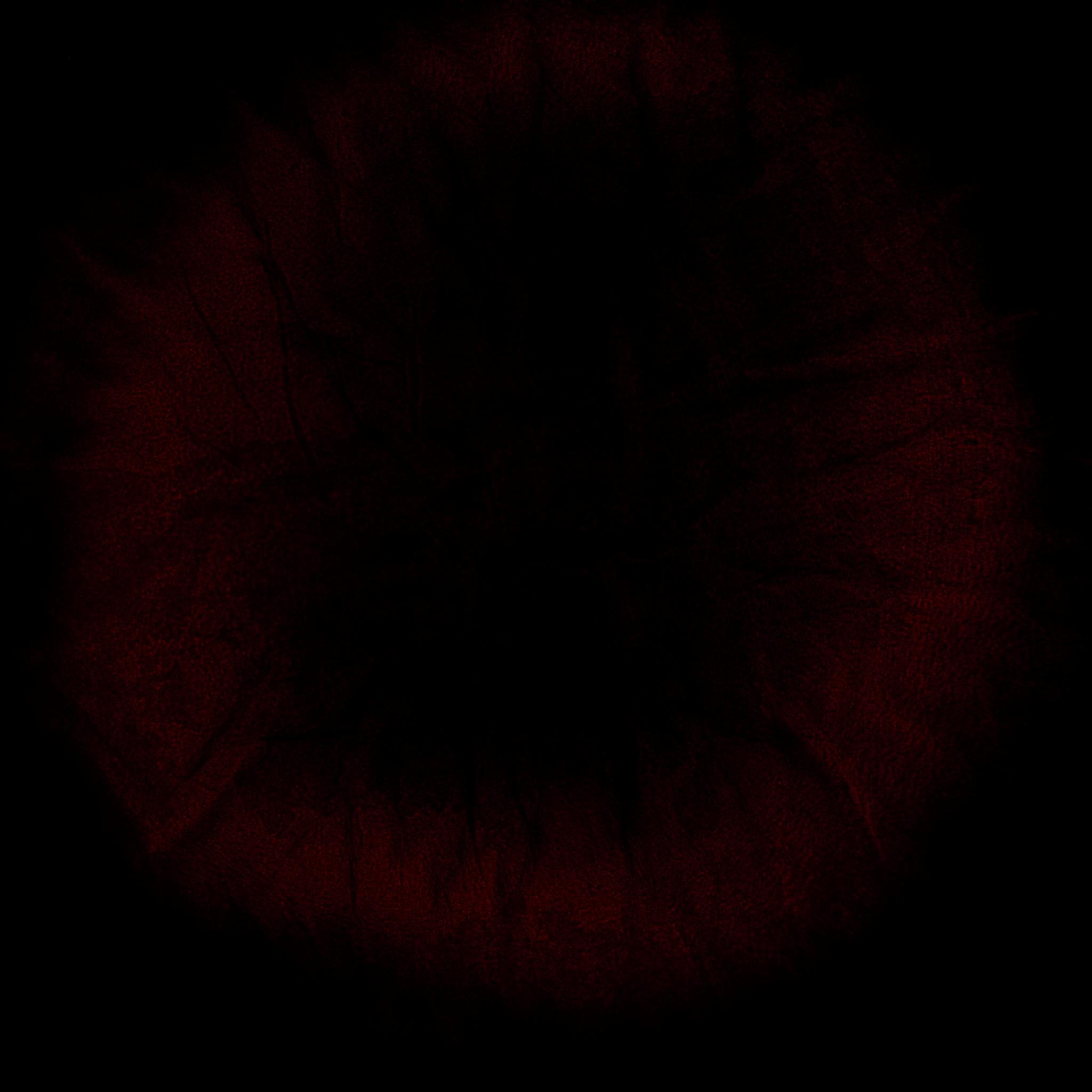

Supplement: S1 File — (ZIP) [file pone.0308204.s001.zip › S1 file. Birefringence Images/B-PK/0 degee/2349OD/IW10.jpg]

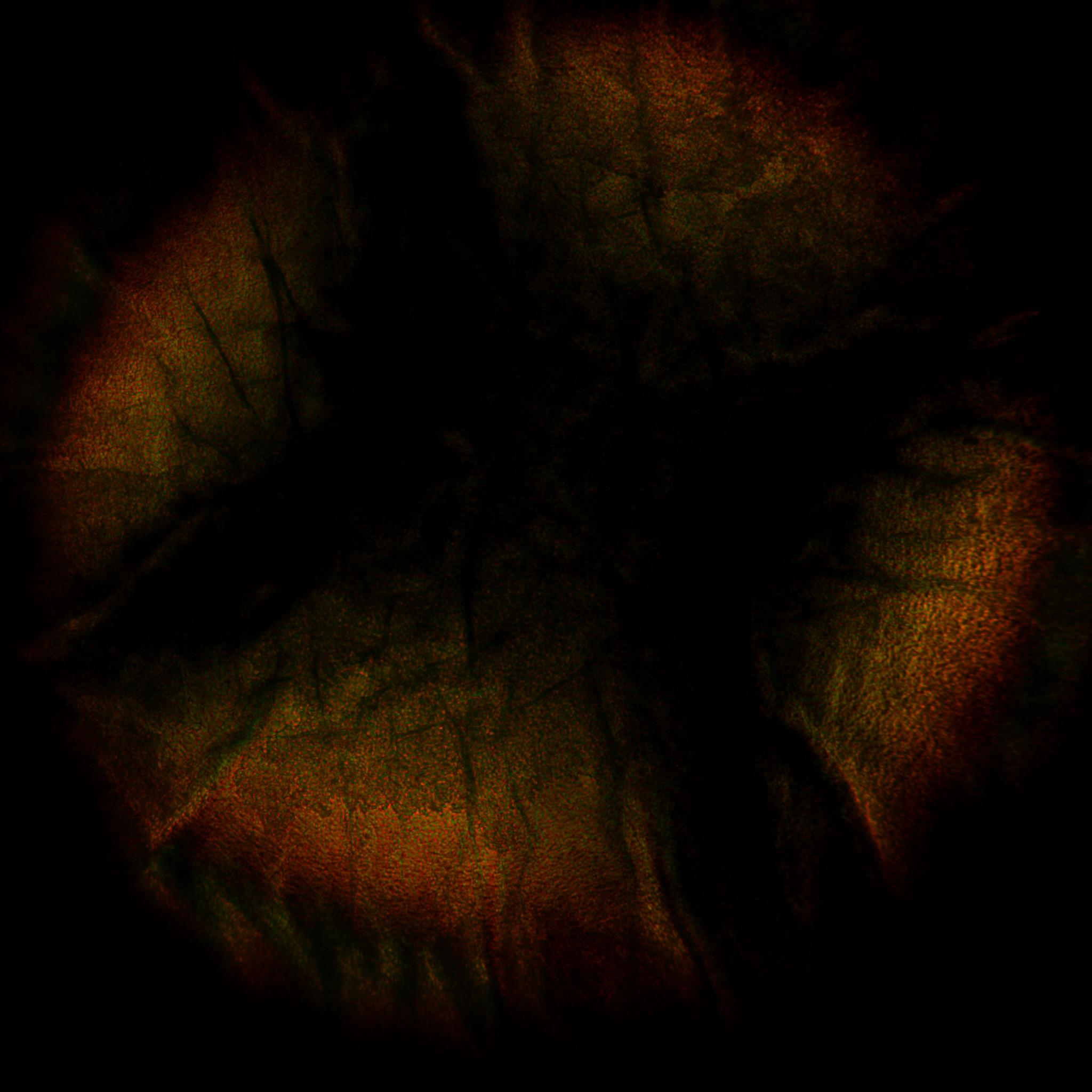

Supplement: S1 File — (ZIP) [file pone.0308204.s001.zip › S1 file. Birefringence Images/B-PK/0 degee/2349OD/IW2.jpg]

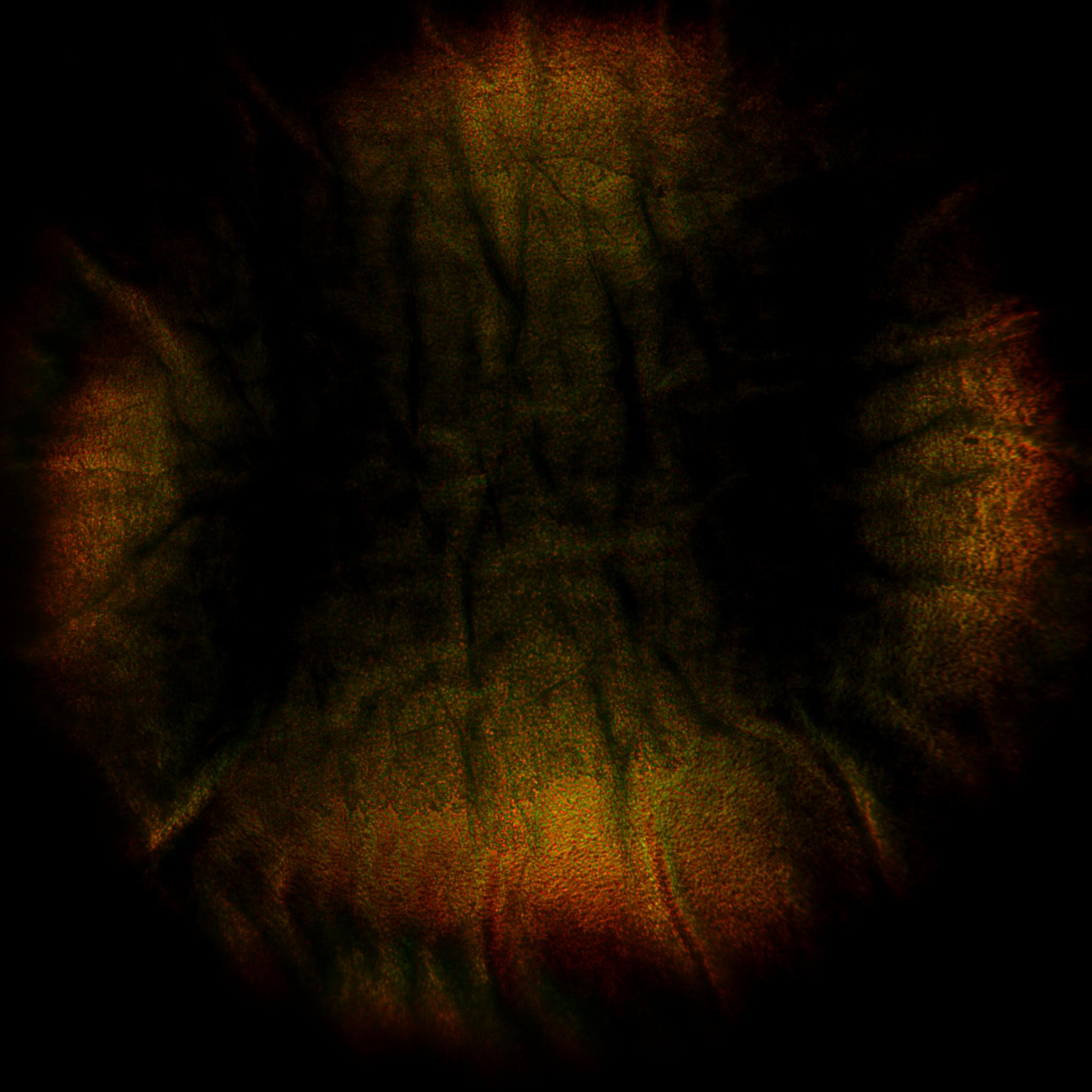

Supplement: S1 File — (ZIP) [file pone.0308204.s001.zip › S1 file. Birefringence Images/B-PK/0 degee/2349OD/IW3.jpg]

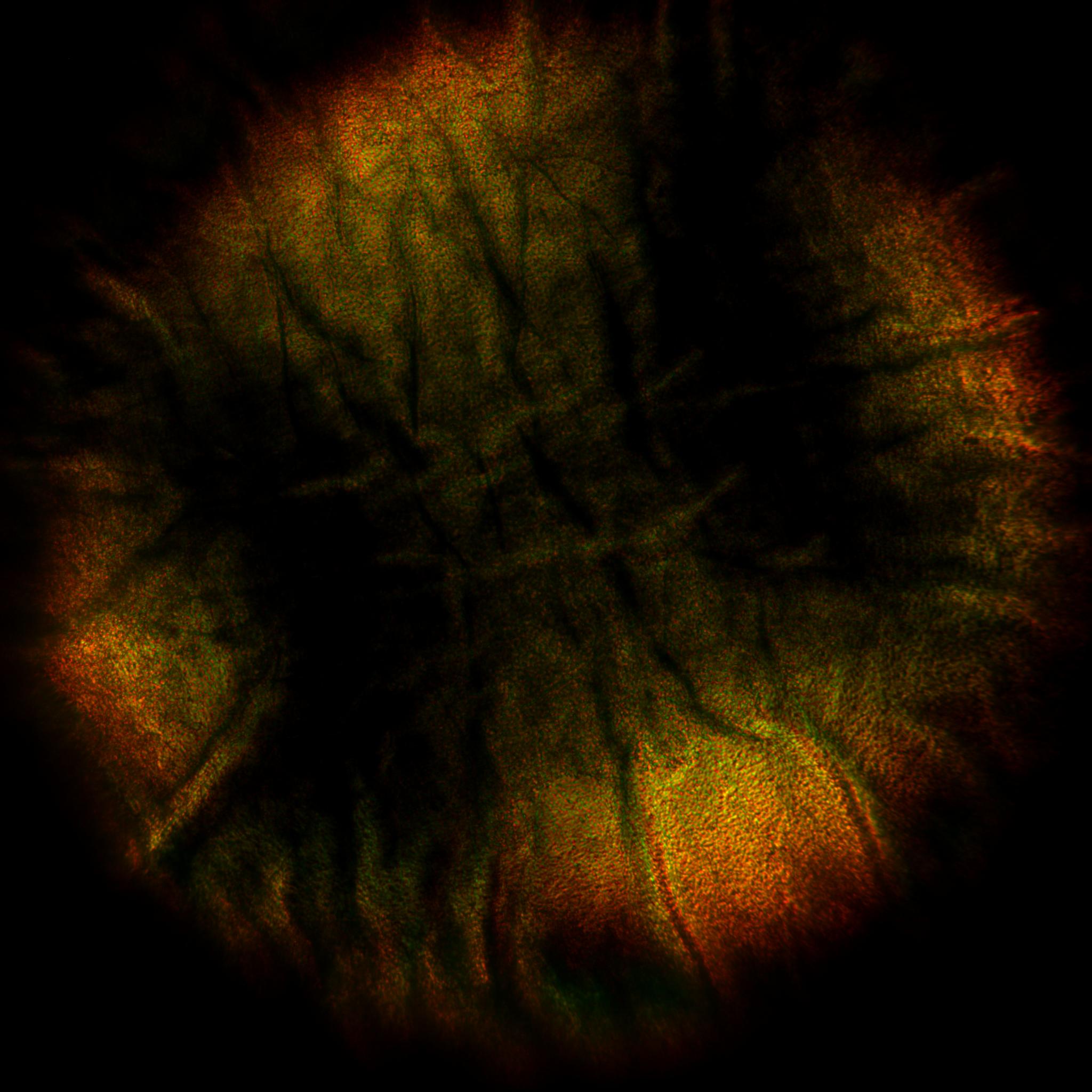

Supplement: S1 File — (ZIP) [file pone.0308204.s001.zip › S1 file. Birefringence Images/B-PK/0 degee/2349OD/IW4.jpg]

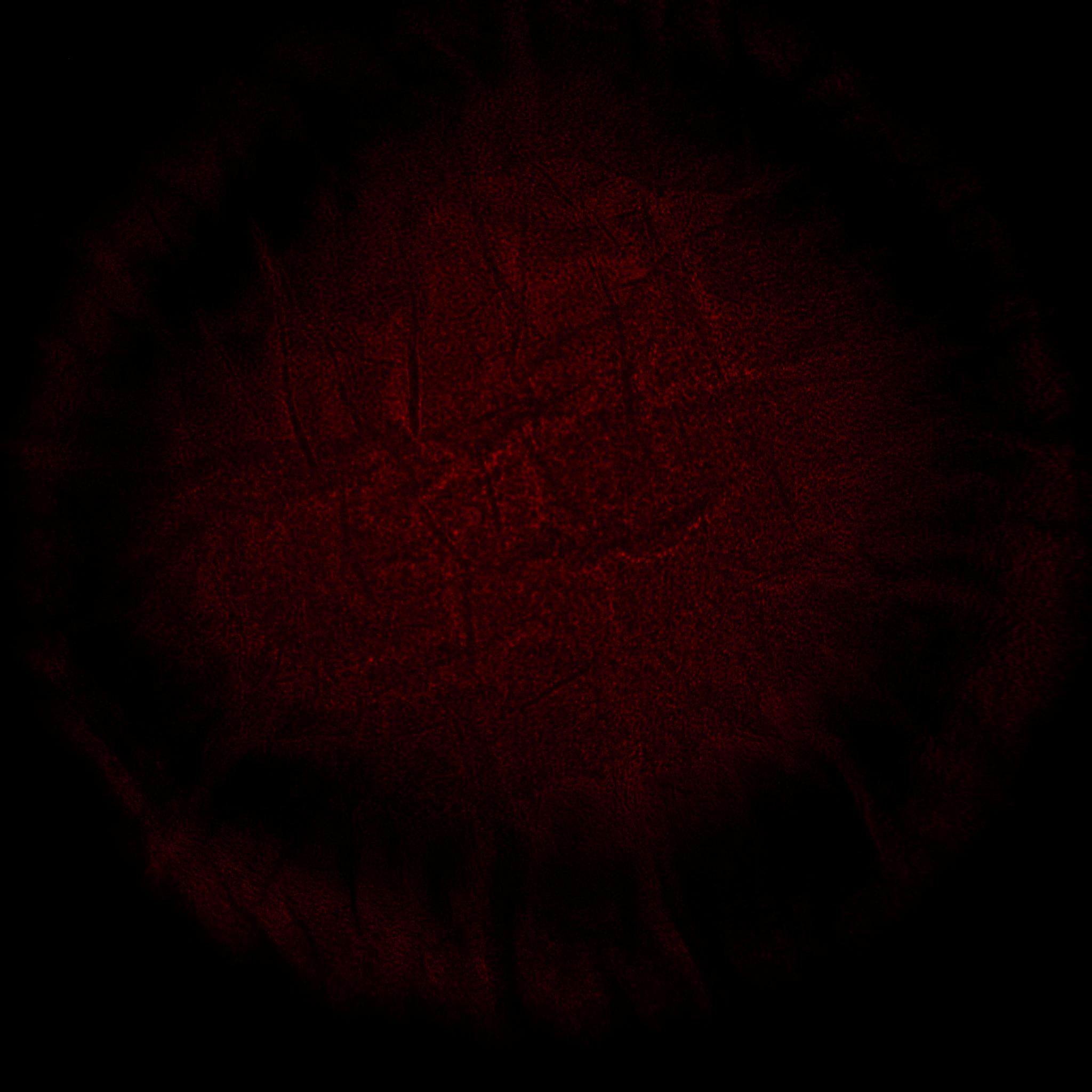

Supplement: S1 File — (ZIP) [file pone.0308204.s001.zip › S1 file. Birefringence Images/B-PK/0 degee/2349OD/IW5.jpg]
