# Supplementary material for: Collagen imaging reveals synergistic effects of sutures and host-donor misalignment on topographical irregularities in penetrating keratoplasty
Source: PLoS One. 2024 Aug 8;19(8):e0308204. doi: 10.1371/journal.pone.0308204 (PMC11309498; doi:10.1371/journal.pone.0308204)
Supplement: Attachment — Submitted filename: Response to Reviewers_Revision 3.docx [file pone.0308204.s003.docx]

Response to Reviewer-3

(Reviewer comments are shown in black-colored text, and responses to reviewer

comments are shown in blue-colored text)

**Reviewer #3**: **(1)** … birefringence analysis of all five corneas was done at once: This means, that the birefringence analysis was performed at different time points after keratoplasty rather than a two-day delay for all (first cornea 2-day delay, last cornea no-day delay?)? Please correct in manuscript and discuss how different timepoints could affect the measurement.

The authors thank the reviewer for raising doubt regarding the timespan of birefringence imaging of the PK corneas. This has helped the authors to add more information regarding the experimental methodology for better clarity for the readers.

i. As mentioned in the experimental methodology, the PK procedure was spread over two days, and then the birefringence data was taken for all the corneas. Meanwhile, the PK corneas were stored in the MK medium at 4ºC.
ii. Previous studies have shown that the cornea maintains its endothelial viability and ultrastructural integrity for up to 90 hours in the MK medium [1,2].
iii. The birefringence data of the control data was taken on the same day of procurement from the eye bank and followed by the PK procedure within two days.
iv. Before imaging, the visual inspection of the PK corneas showed no signs of deterioration in their optical quality.
v. Although imaged at different time points, it was ensured that all the corneas were imaged in the window of 90 Hours after the procurement to prevent the influence of tissue deterioration on the measurements.
vi. The above points have been incorporated in the revised manuscript, see p. 9.

**(2)** “The preoperative methods to mitigate PPKA include careful selection of the size of the donor tissue and using new techniques such as femtosecond laser-assisted keratoplasty (FLAK) [8,9] and deep anterior lamellar keratoplasty (DALK) [10,11] over conventional keratoplasty.”

Compared to PK DALK does not improve Astigmatism (according to your citations). Outcome of FLAK is related to the interface of the laser. Some have worse astigmatism in keratoconus patients.

The authors thank the reviewer in pointing out the deficiency in the introduction section considering the influence of DALK and FLAK procedures in post-PK astigmatism.

i. The preoperative factors that influence astigmatism are characteristics of the host and the donor tissues. These factors include the age difference between the host and the donor, unidentified preexisting conditions such as keratoconus and conditions such as keratoglobus and pellucid marginal degeneration.
ii. Likewise, the intraoperative component of post-PK astigmatism includes the factors that primarily affect the apposition of the host-donor junction. These factors include trephination-related issues such as eccentric trephination, trephination technique, sharpness of the wound edge, and shape and size discrepancy and suturing techniques.
iii. The astigmatism associated with manual trephination is typically dealt with techniques such as femtosecond laser-assisted keratoplasty (FLAK). The laser-assisted trephination results in better wound edge profile, thereby creating a better matched host-donor junction. Additionally, the technique such as deep anterior lamellar keratoplasty (DALK) has been shown to be equivalent of PK for the outcome of best corrected visual acuity (BCVA), but with no specific advantage for refractive outcome. However, DALK has been considered superior to PK considering the graft survival rate.
iv. The above information has been incorporated in the revised manuscript, see p. 3-4.

**(3)** Table 4: Bonferroni correction is missing.

The authors thank the reviewer for pointing out an important aspect of statistical analysis to incorporate the Bonferroni correction that helps in reducing the increased risk of Type I error when making multiple statistical tests.

v. The suggested changes regarding the Bonferroni corrected p-values have been incorporated in Table 4.
vi. It is interesting that the Bonferroni correction has eliminated the Type I error for the misalignment pairs 0˚ - 60˚ and 30˚ - 60˚. The authors have modified the manuscript based on the Bonferroni corrected p-values, see p.14-15.

**(4)** “Here, the greater spread of birefringence (towards the higher suture tension) “ and “The results on the birefringence behavior of the B-PK and A-PK corneas presented here reveal the synergistic effect of suture tension and host-donor misalignment to create topographic irregularities in PK”

Both are interpretations and not results and these are not supported by the experiments. A classification of the individual sutures to a certain level of tension (based e.g. on clinical features or on true tension measurements) would have been needed for such conclusions. Thus, no distinction can be made between the proportion of the tension and the proportion of misalignment for the measurement results.

The author appreciates the reviewer for their concern towards the conclusions made from the results of the present study.

i. From mechanics’ point of view, the suture tension creates a tension around the suture bite leading to increase in birefringence, which has been observed in the present investigation. Additionally, the erratic behavior of isotropic points (IPs) in misaligned corneas reveals the structural influence of the cornea on the tissue distribution despite the statistically similar increase in birefringence observed around the suture bites amongst all the misaligned corneas.
ii. The authors believe that this finding could be an important link that connects the structural aspects of cornea to the topographic changes in PK surgery.
iii. As rightly pointed out by the reviewer, quantifying the contribution of suture tension and misalignment is an important aspect that would shed more light into the synergistic effects of suture tension and misalignment between the host and donor cornea. This requires a method of measuring the suture tension during surgery.
iv. The findings of the present study are the insights from the indirect experiments (digital Photoelasticity) as no direct measurement techniques are available to measure the suture tension. To measure the suture tension, one needs to have a (1) strain gauge/ force transducer on the suture line or (2) use optical measurements based on image processing. First method will not ensure the grand challenge. And second method is an indirect method not yet established, and our method of indirect measurement is based on a proven experimental mechanics [3,4].
v. Nevertheless, the authors strongly believe that the findings of the experiments conducted in the present study aligns well with the fact that the corneal microstructure plays an important role in tissue distribution while suturing the donor cornea to the host.
vi. In the light of the previous response, the authors would like to reinstate the findings of a computational study [5], which revealed that the mismatch between the collagen architecture of the host and the donor cornea leads to a discrepancy between the actual tight suture and the clinical tight suture (suture corresponding to steep curvature around the suture bite). This could mislead the surgeon to remove the wrong suture instead of the actual tight suture.

References

1.	Aquavella J V., Van Horn DL, Haggerty CJ. Corneal preservation using M-K medium. American Journal of Ophthalmology. 1975;80: 791–799. doi:10.1016/0002-9394(75)90273-1

2.	Basu PK. A review of methods for storage of corneas for keratoplasty. Indian journal of ophthalmology. 1995;43: 55–58.

3.	Gururani H, Chittajallu SNSH, Ramji M, Basu S, Chinthapenta V. An In-Vitro Investigation on the Birefringence of the Human Cornea Using Digital Photoelasticity. Exp Mech. 2023;63: 205–219. doi:10.1007/s11340-022-00910-1

4.	Gururani H, Chittajallu SNSH, Doulatramani M, Manoharan R, Basu S, Chinthapenta V. Intraoperative collagen imaging of sutured cornea: A way towards managing post-penetrating keratoplasty astigmatism. Med Eng Phys. 2023; 104076. doi:10.1016/j.medengphy.2023.104076

5.	Richhariya A, Sangwan VS, Xu M, Punjabi S, Perucchio R, Yoon G. Multifactorial Analysis of Penetrating Keratoplasty. Invest Ophthalmol Vis Sci. 2015;56: 1558.
