# Supplementary material for: Collagen imaging reveals synergistic effects of sutures and host-donor misalignment on topographical irregularities in penetrating keratoplasty
Source: PLoS One. 2024 Aug 8;19(8):e0308204. doi: 10.1371/journal.pone.0308204 (PMC11309498; doi:10.1371/journal.pone.0308204)
Supplement: Attachment — Submitted filename: Changes suggestions.docx [file pone.0308204.s004.docx]

Changes suggestions:

In Line 66:

Original:

[13,14]. Further, the techniques such as SSR, sequential suture removal [15,16], intra-stromal ring (ICRS) insertion [17], intraocular lens (IOLs) [18,19], Photorefractive Keratectomy [20,21],laser in-situ keratomileusis (LASIK) [22,23] and repeat keratoplasty [24] are used post-operatively to manage PPKA.

Suggestion:

[13,14]. Further, the techniques such as sequential suture removal (SSR) [15,16], insertion of intra corneal ring segments (ICRS) [17], intraocular lens (IOLs) [18,19], Photorefractive Keratectomy (PRK) [20,21],laser in-situ keratomileusis (LASIK) [22,23] and repeat keratoplasty [24] are used post-operatively to manage PPKA.
